# Non-motor Clinical and Biomarker Predictors Enable High Cross-Validated Accuracy Detection of Early PD but Lesser Cross-Validated Accuracy Detection of Scans Without Evidence of Dopaminergic Deficit

**DOI:** 10.3389/fneur.2020.00364

**Published:** 2020-05-11

**Authors:** Charles Leger, Monique Herbert, Joseph F. X. DeSouza

**Affiliations:** ^1^Department of Psychology, York University, Toronto, ON, Canada; ^2^Neuroscience Diploma, York University, Toronto, ON, Canada; ^3^Centre for Vision Research, York University, Toronto, ON, Canada; ^4^Department of Biology, York University, Toronto, ON, Canada; ^5^Canadian Action and Perception Network (CAPnet), Vision: Science to Applications (VISTA), York University, Toronto, ON, Canada

**Keywords:** predicting Parkinson's, SWEDD, random forest, XGBoost, logistic regression

## Abstract

**Background:** Early stage (preclinical) detection of Parkinson's disease (PD) remains challenged yet is crucial to both differentiate it from other disorders and facilitate timely administration of neuroprotective treatment as it becomes available.

**Objective:** In a cross-validation paradigm, this work focused on two binary predictive probability analyses: classification of early PD vs. controls and classification of early PD vs. SWEDD (scans without evidence of dopamine deficit). It was hypothesized that five distinct model types using combined non-motor and biomarker features would distinguish early PD from controls with > 80% cross-validated (CV) accuracy, but that the diverse nature of the SWEDD category would reduce early PD vs. SWEDD CV classification accuracy and alter model-based feature selection.

**Methods:** Cross-sectional, baseline data was acquired from the Parkinson's Progressive Markers Initiative (PPMI). Logistic regression, general additive (GAM), decision tree, random forest and XGBoost models were fitted using non-motor clinical and biomarker features. Randomized train and test data partitions were created. Model classification CV performance was compared using the area under the curve (AUC), sensitivity, specificity and the Kappa statistic.

**Results:** All five models achieved >0.80 AUC CV accuracy to distinguish early PD from controls. The GAM (CV AUC 0.928, sensitivity 0.898, specificity 0.897) and XGBoost (CV AUC 0.923, sensitivity 0.875, specificity 0.897) models were the top classifiers. Performance across all models was consistently lower in the early PD/SWEDD analyses, where the highest performing models were XGBoost (CV AUC 0.863, sensitivity 0.905, specificity 0.748) and random forest (CV AUC 0.822, sensitivity 0.809, specificity 0.721). XGBoost detection of non-PD SWEDD matched 1–2 years curated diagnoses in 81.25% (13/16) cases. In both early PD/control and early PD/SWEDD analyses, and across all models, hyposmia was the single most important feature to classification; rapid eye movement behavior disorder (questionnaire) was the next most commonly high ranked feature. Alpha-synuclein was a feature of import to early PD/control but not early PD/SWEDD classification and the Epworth Sleepiness scale was antithetically important to the latter but not former.

**Interpretation:** Non-motor clinical and biomarker variables enable high CV discrimination of early PD vs. controls but are less effective discriminating early PD from SWEDD.

## Introduction

The second most common neurodegenerative disease (1), Parkinson's disease (PD) does not have a sudden onset but develops along a continuum. Indeed decades prior to emergence of cardinal motor dysfunction approximately one-third of substantia nigra pars compacta dopamine neurons may be lost (2–4). Further, non-human primate research has confirmed gross motor symptoms, such as bradykinesia, rigidity and resting tremor, occur subsequent to 70% loss of striatum dopaminergic synapses, verifying the development of pathology well in advance of PD motor symptom onset (5, 6). This potential for insidious development of PD pathology underlines the importance of early detection.

As concluded in commentary (7), there is overwhelming evidence of PD pathology incipience predating cardinal motor symptoms (resting tremor, postural instability, rigidity, and bradykinesia) likely stemming from early brainstem involvement and manifested in non-motor sense alteration such as olfaction and eye-movement behavior disruption. The olfactory system in particular is a candidate entry point for environmental insults that may be the inception point of pathological proteins (α-synuclein and other proteins such as ubiquitin and Tau) of which Lewy bodies are composed (8–11). Moreover, brainstem (e.g., the vagus nerve dorsal motor nucleus) and spinal cord Lewy body pathology could also explain preclinical PD occurrence of gastrointestinal complication and constipation (1, 12–14).

A prospective analysis demonstrated a 10% risk of conversion of PD-asymptomatic relatives of PD patients positive for hyposmia (olfactory deficit) to PD at 24 months from baseline (15). Single-photon emission computed tomography (SPECT) scans of 4 of 25 hyposmic relatives, asymptomatic for PD, indicated dopamine transporter (DAT) uptake reduction but at a subclinical level (16). Further, early stage PD hyposmia is associated with reduced striatal dopamine uptake (15, 17), and there is a particularly strong (positive) correlation between putamen DAT uptake and hyposmia (17). In addition, hyposmia has been reported associated with idiopathic rapid eye movement (REM) behavior disorder (RBD), where 29 of 30 RBD patients had RBD comorbid with hyposmia and 3 of 11 who underwent SPECT scans had indication of nigrostriatal dopamine neuron degeneration (18). RBD is a sleep disorder typified by vivid, action-filled dreams, but most uniquely by loss of REM-sleep muscle atonia with consequent dream enactment including violent behavior such as punching (19). A high association of hyposmia, RDB and beta amyloid 1–42 (Aβ_1−42_) with cognitive decline has predicted cognitive decline 24 months after assessment; male gender did not contribute to the predicted cognitive impairment (20).

Individuals without PD but with hyposmia or RDB have achieved a positive predictive value for PD of ≥ 40% (21–23), and in the majority of those with RBD a Lewy body disorder develops (24). But RBD has shown a particular sensitivity to α-synuclein pathology such as it occurs in PD (21, 25), and has a prevalence in PD of ~37–47% (26, 27) compared to the general population prevalence of 5% (28).

Cerebral spinal fluid (CSF) constituents, readily accessible and inexpensive to acquire, provide a window to central nervous system pathological states (29). Relative to controls, research on drug naïve early PD has demonstrated reduced levels of CSF biomarkers associated with early PD pathology. Specifically, lower CSF levels of phosphorylated tau_181_, (pTau), total tau (tTau), amyloid beta _1−42_ (Aβ_1−42_), and α-synuclein were found in early PD relative to controls (30); findings that were confirmed by the same research team in a subsequent study using a larger cohort (31), though Aβ_1−42_ in this study was not significantly lower in early PD relative to controls. In the first of the latter two studies reduced levels of pTau and Aβ_1−42_ were associated with PD diagnosis; reduced levels of α-synuclein and tTau were associated with heightened motor disruption (30).

Prior to emergence of cardinal motor symptoms, early phase PD diagnosis is further complicated by a category of pathology that fulfills PD clinical diagnostic criteria but without evidence of dopaminergic deficit- a PD lookalike. This particular pathology category designated scans without evidence of dopaminergic deficit (SWEDD) presents with some extent of parkinsonian motor symptoms but normal striatal dopamine neuron status and DAT uptake. A range of 1–15% of those diagnosed with PD demonstrate SWEDD group membership (32–34), and remain in the SWEDD category for at least 4 years after diagnosis (35). Accuracy of SPECT assessment in general, using visual or sem-quantitative methods is reduced by the potential PD-intermediate SWEDD condition (32, 35). SWEDD constitutes a heterogeneous category; a small portion may represent a subtype of PD, but most cases have other conditions (e.g., dystonia, essential tremor, fragile X permutation, etc.), and most of those categorized as SWEDD have been misdiagnosed (36).

There is an established early premotor-dysfunction phase of PD (37) to which non-motor clinical variables, notably olfactory acuity and RBD, as well CSF biomarkers (e.g., α-synuclein) have demonstrated early PD stage detection sensitivity (38–43). Hyposmia and RBD are not specific to PD, yet hyposmia in particular has been the predictor of greatest import in early PD predictive modeling (43), the dominant predictive model driver for those with genetic risk (41), and of secondary import only to imaging in other early PD predictive modeling research (42). Biomarkers, including CSF α-synuclein, pTau and tTau, can distinguish early PD from healthy controls but are inadequate for screening (38, 39) and have <80% diagnostic utility (29, 44, 45). Usage of both non-motor clinical and biomarker variables may lend clinical variables (notably hyposmia and RBDQ) enhanced PD-specific responsiveness, a heightened PD-specific responsiveness derived from biomarker (possibly alpha synuclein) putative PD-specific sensitivity (43). Accordingly, development of predictive models that combine non-motor clinical variables and biomarkers is a promising avenue of research. Moreover, although DAT scan imaging is arguably tantamount to a PD gold standard diagnostic measure it is quite expensive to acquire, and considerably less definitive distinguishing early PD from the SWEDD condition (35).

There is a scarcity of cross-validated classification research utilizing combined non-motor clinical and biomarker features in predictive models to distinguish early PD from healthy controls or from SWEDD. One such study (46) also included dopaminergic-imaging markers while other research (43) forwent imaging as a predictor and based model development only on combined non-motor clinical and biomarker features. The studies just referenced built models using data obtained from Parkinson's Progression Marker Initiative (PPMI), an invaluable resource of longitudinal PD-related data.

The current work had 3 main objectives. The first objective was to demonstrate a consistently high level of early PD/control (binary) cross-validated classification accuracy across 5 distinct models types utilizing non-motor clinical and biomarker data sourced from the PPMI. It was posited that the five algorithmically distinct models would classify idiopathic early PD relative to healthy controls with high cross-validation accuracy (i.e., AUC > 0.80) when applied to validation/test data unseen by the models. Although each of the differing model algorithms was not expected to perform identically, a close range of performance among models if achieved would provide a level of consistency further validating the early PD discriminatory usefulness of non-motor clinical and biomarker variables.

The second objective was to broaden understanding of the PD disease-predictor relationship by, in addition to the early PD/control classification analysis, conducting an early PD/SWEDD (binary) classification analysis. An early PD/SWEDD analysis was prompted by preliminary assessments suggesting predictor importance to model class prediction might differ for early PD/SWEDD relative to early PD/control. Moreover, because of the known diversity of the SWEDD category (36) early PD/SWEDD discrimination was expected to be less definitive and typified by lower AUC and other performance metric scores when applied to validation/test data unseen by the models. Of note, it was expected that modeling early PD vs. SWEDD would result in a classifier(s) advantageous for differentiation of SWEDD category patients without PD pathology from those with incipient PD pathology. Such a model could be used in clinical practice or research to reduce SWEDD category heterogeneity. The third objective was simply to report model selection and rank of features of import to early PD/control vs. early PD/SWEDD classification. Differing feature selection and rank of features by a given model between analyses has clinical diagnostic and research implications.

There is never a guarantee that one model will outperform another (47). Comparing performance of several models to reveal the highest performing classifier(s) is one means to potentially improve study caliber. The five distinct classifiers used were logistic regression, binary general additive (GAM) (48–51), decision tree (52, 53), random forest (54), and XGBoost (55). Model classification performance was compared using the receiver operator characteristic area under the curve (AUC), sensitivity, specificity, general accuracy and the Kappa statistic. Feature collinearity in all models was restricted (i.e., *r*_*s*_ < 0.75) and all models were tested on a validation partition unseen by models during training. To the best of our knowledge, unique to the current work was the set of five classifiers used and the dual early PD/control and early PD/SWEDD analyses approach adopted.

## Methods

### Procedures

**Figure 1:**
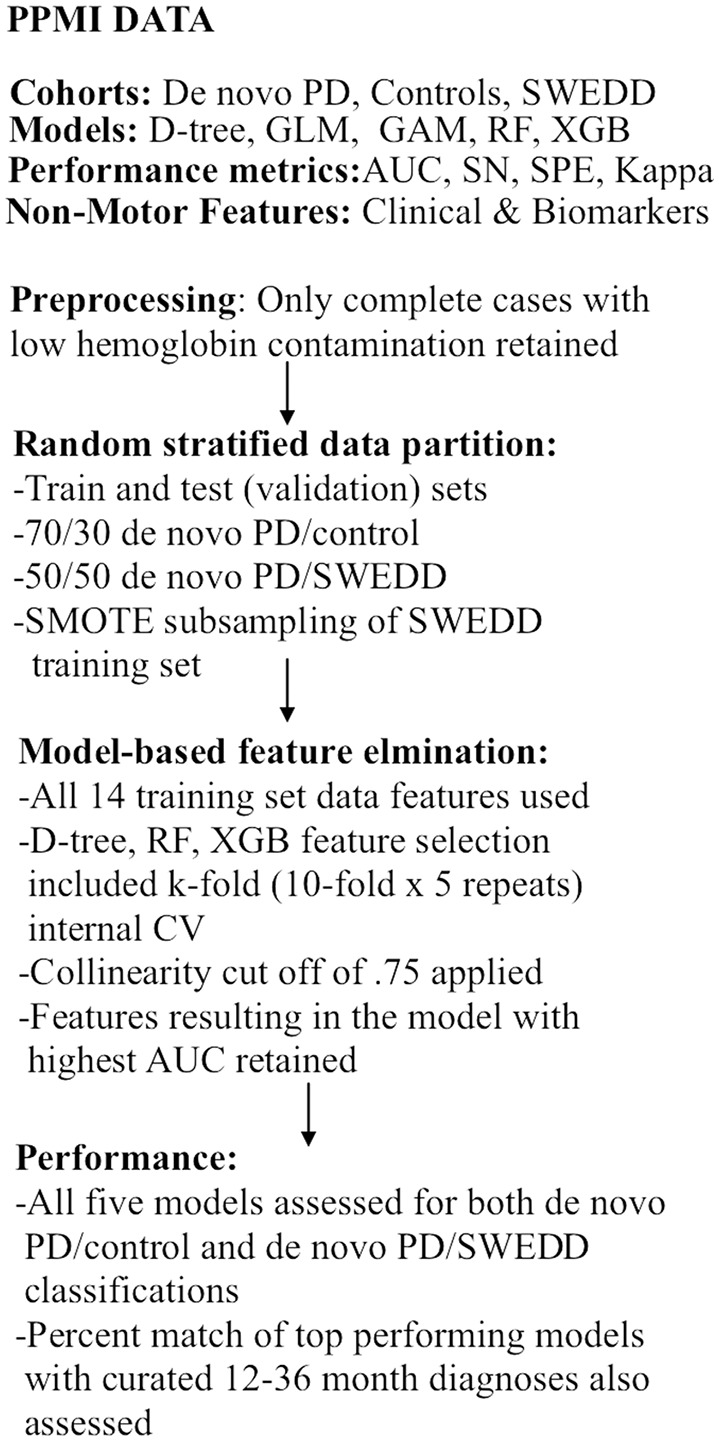
Analyses flow chart. CV, cross-validation; GAM, general additive model; GLM, logistic re-gression; AUC, receiver operator characteristic area under the curve; RF, random forest; SMOTE, synthetic minority oversampling technique; SN, sensitivity, SPE, specificity; SWEDD, scans without evidence of dopamine deficit.

As already stipulated, classification performance was compared for logistic regression, general additive (GAM) (48–51), decision tree (52, 53), random forest (54), and XGBoost (55) models in two separate analyses: early PD vs. control and early PD vs. SWEDD (scans without evidence of dopamine deficit). This amounted to building 10 (5 × 2) classifiers. The AUC was the main performance metric. Sensitivity, specificity, general accuracy and the Kappa static were also determined. The general sequence of data analysis steps is depicted in Figure 1. Also the two highest performing classifiers from the early PD vs. control classification analyses were applied to SWEDD test data to assess conversion of SWEDD to PD. The case-wise percentage of model predicted SWEDD to PD conversion that conformed to (available) longitudinal PPMI curated 12–36 months diagnosis was then assessed. Further, the case-wise percentage of early PD vs. SWEDD model sensitivity and specificity that conformed to (available) PPMI curated longitudinal 12–36 months diagnoses was also determined for the two highest performing early PD vs. SWEDD classifiers. Longitudinal 12–36 months DAT scan mean putamen values provided an imaging measure of disease.

After screening, the SWEDD minority class rate became 13% (43/338), and the random stratified training/validation data split further reduced the SWEDD training cohort of just 22 cases (and 148 early PD). To improve data symmetry early PD/SWEDD models were trained on SMOTE (synthetic minority oversampling technique) subsampled data.

It is underlined that to prevent leakage of test data information into training data, model features were selected only from training data; models were trained only on training data. This mitigated overly optimistic model performance estimates on the test data. To ensure reproducibility, one specific seed value was set prior to partitioning of data and model execution. All data used can be obtained from https://github.com or the corresponding author.

### Participant Data

Data used in the preparation of this article was obtained from the Parkinson's Progression Markers Initiative (PPMI) database (www.ppmi-info.org/data). For up-to-date information on the study, visit www.ppmi-info.org. The PPMI is a landmark, multicenter, longitudinal research effort mandated to identify PD markers (4). It is a public-private partnership funded by the Michael J. Fox Foundation for Parkinson's Research and the funding partners, include AbbVie, Allergan, Amathus Therapeutics, Avid Radiopharmaceudicals, Biogen, BioLengend, Bristol-Myers Squibb, Celgene, Denali Therapeutics, GE Healthcare, Genentech, GSK, Lilly, Lundbeck, Merck, Meso Scale Discovery, Pfizer, Piramal Imaging, Prevail Therapeutics, Roche, Sanofi Genzyme, Servier, Takeda, Teva, UBC, Verily, and Voyager.

Subject data was anonymized while also allowing individual subjects to be tracked across different studies. Acquired subject data (downloaded July 31, 2019) included three cohorts: 423 early (*de novo*) PD, 196 healthy controls (controls or HC), and 64 scans without evidence of dopamine deficiency (SWEDD). With respect to the models developed, only baseline data was used, and such baseline data originated within 2 years of PPMI project enrolment. It warrants mention that the PPMI early PD baseline cohort is drug naïve but symptomatic.

After screening (see *2.4 Screening*) and random stratified partitioning of data in to train and validation (test) data sets, train and validation data instances were as follows: the early PD/control training data set, had 207 early PD (133 male) vs. 91 controls (55 male); the validation set had 88 early PD (59 male); and 39 controls (26 male). The two top early PD/control models were also tested on all 43 SWEDD and the 39 controls from the early PD/control test set. For the early PD/SWEDD classification, a SMOTE-based training set of 44 early PD (30 male) and 44 SWEDD (30 male) was used for all early PD/SWEDD model training, except with respect to the decision tree. The early PD/SWEDD decision tree model used SMOTE data, but SMOTE data obtained during resampling, which had a higher AUC compared to the model based on standard SMOTE subsampling [See Supporting Information V Section Decision Tree Early PD vs. SWEDD Classification Results (SMOTE-Based Model)]. This resulted in a decision tree training set of 88 early PD (63 male) and 66 SWEDD (41 male). The validation or test set used for early PD/SWEDD (not altered or subsampled) consisted of 147 early PD (92 male) and 21 SWEDD (11 male).

### Feature Elimination and Hyper-Parameter Tuning

Final features (predictors), selected only from training set data, were determined by model-based feature elimination coupled with the AUC: models with features amounting to the highest model AUC constituted the final models applied to the test data sets. For tree-based models, caret package (56) internal cross-validation (10-fold, 5 repeats) resampling was used to tune hyper-parameters and arrive at the optimal feature set (see *Modeling and the caret package* Supporting information IV). Stepwise regression employing the Akaike information criterion (AIC) (57) was used for logistic regression feature elimination. GAM models used the same features as logistic regression. For the logistic regression GAM, the intent was to use the GAM to supplement and corroborate GLM results but also as a distinct classification model. The logistic regression GAM was executed using the same stepwise regression features selected for logistic regression. The caret package 0–100% ranking scale of feature importance to classification was used. Model built-in indices of feature rank is juxtaposed to carrot package feature ranking in Supporting Information V.

### Clinical Assessments and Cerebral Spinal Fluid Assays

Features (14 total) in prior research (see introduction) demonstrating promise discriminating early PD were assessed. Biological predictors, the biomarkers, were cerebral spinal fluid (CSF) levels of beta-amyloid 1-42 (Aβ_1−42_), α-synuclein, tau phosphorylated at threonine 181 (pTau), and total tau (tTau). With respect to biomarkers, because hemoglobin contamination can influence the biologic measures, exclusion of samples with > 200 ng/ml has been recommended (45); this screening recommendation was adopted in the current work. The non-motor clinical measures included anxiety, depression, cognition, constipation, daytime sleepiness, rapid eye movement sleep behavior disorder questionnaire (RBDQ) (58) and olfactory acuity (hyposmia) based on University of Pennsylvania Smell Identification Test (UPSIT) (59). The latter was reverse scaled here: higher is proportional to lesser olfactory acuity. The biomarkers are continuous variables, and the clinical variables are continuous or semi-continuous scales. Note, for all clinical measures, except cognition, higher scores are generally suggestive of pathology while the reverse typically holds for the biologics, where lower CSF biological values suggest pathology.

### Screening

The main screening criteria were complete records across all modeled variables for a given subject's data as well as low hemoglobin blood contamination [ <200 ng/mL (45)]. Complete imaging records (caudate and putamen SPECT DAT uptake) and MDS-UPDRS III, (60) scale data were also required. Strict adherence to the blood contamination criterion eliminated 131 cases, reducing the data-set to 151 controls, 328 early PD, and 47 SWEDD. Control group case number was further reduced by two missing UPDRS III scores, three missing anxiety scores, two missing MoCA scores, and 14 missing striatal DAT uptake values. For early PD, case number was further reduced by 15 cases of incomplete RDBQ scores, two incomplete instances of depression, one incomplete daytime sleepiness score, three incomplete MoCA records, and 12 incomplete dopamine transporter (DAT) uptake records. For SWEDD, there was one ESS missing record, one missing MoCA record and two missing olfaction records. Subsequent to this screening the final number of participants was 468 (130 controls; 295 early PD; 43 SWEDD).

### Imaging

Because dopamine active transporter (DAT) uptake and clinical motor (MDS-UPDRS III) status measures are virtually ever-present in PD assessments, they were included as background indices to help quantify extent of pathology. Single Photon Emission Computed Tomography (SPECT) dopamine transporter (DAT) uptake (i.e., striatal binding ratio) data was used as the striatal (dopamine) measure of neurodegenerative status. A complete technical specification and operations SPEC manual is provided by PPMI and is available at http://www.ppmi-info.org/wp-content/uploads/2017/06/PPMI-TOM-V8_09-March-2017.pdf.

### Statistical Analyses

The type I error rate was set at.05 (α = 0.05). Statistical analysis was conducted in *R* (61). The univariate distribution of all variables was initially examined for indications of relative data normality using descriptive statistics, density plots, and numeric (62) analyses. Gender proportion within groups was assessed with binomial tests; two-sample tests for equality of gender proportion were used to assess gender proportion between early PD and HC groups and early PD and SWEDD groups. Boxplots were used to show the range, or spread of variable data values for early PD, control and SWEDD groups. Bivariate variable relationships were assessed with correlation tests and scatterplots. The SPECT DAT values were included in these bivariate assessments to help link the broadly acknowledged disease indicator SPEC DAT with the non-motor clinical and biomarker predictors; imaging values then, provided an indication of disease-relation to predictors (but imaging was not included in the classification analyses). Because the data was generally non-normally distributed, robust *t*-tests (63) were used to compare variables between groups. Models initially included (controlled for) age, education and gender.

Collinearity can make logistic regression coefficients unstable, less precise (64, 65). It can result in GAM concurvity (a form of co-linearity where one smooth term approximates another) (66). For tree models, however, concern for collinearity of variables is controversial (67). But considering random forest, for example, one of two or more correlated features can be randomly selected without preference; impurity removed by the selected feature potentially masks additional impurity that could have been removed by the correlated features (68). Indeed, with correlated features, less relevant features can take the place of more importance features (69) and feature ranking can be inaccurate (70). Because collinearity is certainly problematic for logistic regression, can potentially bias feature selection, ranking and hence classification of GAM and tree models, the current work adopted a multicollinearity of cut off of *r*_*s*_ = 0.75, which is relatively sensitive to pairwise correlations (71). To prioritize unbiased feature selection and classification for all models, features exceeding the cutoff were not combined within the same model. Note however, that for all models, all 14 non-motor clinical and biomarker features were included in initial model-based feature elimination. The collinearity cut off was only applied to the final model feature set to increase reliability of feature importance ranking and classification.

The lone parametric model was logistic regression. Details on logistic regression assumption assessments (see Supporting information I) and the handling of assumption violation can be found in Supporting information V [see Sections Logistic Regression and General Additive Model Classification Analyses, Early PD vs. Controls and Logistic Regression and General Additive Classification Analysis, Early PD vs. SWEDD (SMOTE-Based Model)]. The logistic general additive model (GAM) was used to corroborate logistic regression model (GLM) results. MoCA in the early PD/control analysis and years of education in the early PD/ SWEDD analysis violated linearity of the logit [Box-Tidwell (72)]. Transforms were attempted (e.g., the square root, log, cube root) with only minor improvement. Consequently, the offending two variables were simply converted from continuous to categorical variables (quartiles) but at the likely cost of information loss, information retained by a GAM smoothing function applied to the same variables. Accordingly, logistic regression GAMs in addition to being distinct classification models, also added perspective to logistic GLM output.

The GAM model thin plate smoother function (the default smooth function in the mgcv package) (51) was the basis used. The restricted maximum likelihood (REML) function (with thin plate smoother as a random effects term) was chosen as the smoothness selection method governing the extent of wiggle in the wiggly parts of the thin plate smoother basis function. The REML method was used because it effectively penalizes overfitting (50). The degrees of freedom associated with a smoothed predictor, initially set by REML, were checked by ensuring the effective degrees of freedom (edf) of a given smoothed predictor was less than *k* (the upper limit on the degrees of freedom). The GAM parameter output and a diagnostic qqplot is provided in Supporting Information II. Deviance, pseudo *R*^2^ (73) and explained deviance values of the logistic GLM and GAM respective models are in Supporting Information V.

### Classification Performance Metrics

The AUC, rather than simple misclassification error, was used in the process of model-based feature elimination to select optimal features. The AUC was also employed to select tree-model optimal hyper-parameters settings using the caret package (56). Early PD was the predicted class in the early PD/control classification; SWEDD was the predicted class in the early PD/SWEDD classification. Model performance was based on model and cross-validated test set AUC, sensitivity, specificity, accuracy and Kappa values. The latter performance metrics are summarized in Section Model Classification Results. The confusion matrices for sensitivity, specificity, accuracy and Kappa are outlined in Supporting Information IV and further details are provided in Supporting information III.

At the default 0.50 cut-off classification threshold predictive classification probabilities > 0.50 are categorized as positive events: early PD rather than control; SWEDD rather than early PD. However, the default 0.50 cutoff often provides a less than ideal balance of confusion matrix performance metric values. Therefore, for each model, sensitivity, specificity, Kappa and accuracy metrics were reported at the optimized classification threshold (Supporting information V includes confusion matrix performance metrics at the 0.50 cutoff). The optimized model threshold was selected by the pROC package (74) utilizing a modified (75) version of the Youden Index (76). There were two exceptions where the optimal threshold point of balanced of sensitivity and specificity was point closest to the ROC curve top left. The AUC non-parametric method (77, 78) was used as implemented in pROC (74) because it has relaxed normality assumptions. In addition to AUC values and graphs for each model, a roc test for correlated (referring to the same response variable used by different models) ROC curves (74) was used to determine if the two highest performing models from each classification analyses significantly differed. Bonferroni family-wise error correction was used for AUC comparison between more than two models. In the current work, AUC ≥ 80% but <90% were regarded as indicative of good classification performance; values ≥90% are regarded as an excellent level of classification performance.

## Results

Following a descriptive statistics section, a summary of the main model classification outcomes (4.0 Model classification results) is provided. As specified in the methods section, only cases satisfying data requirements (i.e., low blood contamination with complete clinical and biologic data for all predictors) were retained. Also subsampling, not used in the early PD/control classification, was employed in the early PD/SWEDD classification in aid of addressing the SWEDD minority class rate of 13% (training set: 22/148).

### Descriptive Statistics and *t*-Tests

Tables 1, 2 provide variable descriptive statistics and pairwise (*t*-tests; Wilcox, 2005): Table 1 pertains to early PD vs. control groups; Table 2 pertains to early PD vs. SWEDD. Density plots in Supporting information V (see Figure SV-1) convey the largely non-normal distribution of the variables. Nine of eleven (82%) clinical and biologics in Table 1 significantly differed between early PD and control groups. By contrast, only 3/11 (27%) clinical and biologics significantly differed between early PD and SWEDD groups in Table 2, two of which, Epworth sleepiness scale (79) (ESS) and years of education, did not significantly differ between early PD and control groups (Table 1). The clinical variable University of Pennsylvania Smell Test (59), reverse-scaled in the current work (Upsit-rev), was significantly higher (higher reverse-scaled UPSIT is indicative of greater olfactory loss, more severe hyposmia) in early PD compared to controls as well as in early PD compared to SWEDD. The reverse-scaled UPSIT values are otherwise identical to standard (non-reverse scaled) UPSIT values. SPECT dopamine transporter uptake (DAT uptake) was also significantly different across both groups. Motor symptoms (MDS-UPDRS III) (60) significantly differed between early PD and controls but not between early PD and SWEDD. The number of years of education significantly differed between early PD and SWEDD but not between early PD and controls. With respect to gender proportions not tabulated, a binomial test for controls revealed a proportion of 0.27 females, which significantly differed from the expected proportion of 0.5 (50%), *p* < 0.001. Similarly, a binomial test for the early PD group indicated a proportion of 0.26 females, which significantly differed from the expected 50%, *p* < 0.001. Finally, a binomial test for the SWEDD group revealed a proportion of 0.30 females, which significantly differed from the expected 50%, *p* < 0.01. The proportion of male and female cases between early PD and control groups did not significantly differ, $\chi_{1}^{2}$ = 0.303, *p* = 0.582. Similarly, there was not a significant gender proportion difference between early PD and SWEDD groups, $\chi_{1}^{2}$ = 0.788, *p* = 0.375.

**Table 1:** Descriptive statistics and t-tests, early PD/controls.

***N*, 425**	**Early PD**, ***n*****, 295: 192 male, 103 female**						**HC**, ***n*****, 130: 81 male, 49 female**						
	***M***	***sd***	***Mdn***	***min***	***max***	***skew***	***M***	***sd***	***Mdn***	***min***	***max***	***skew***	***t-test^***a***^***
**CLINICAL**													
Anxiety	33	10	31	20	63	1	29	7	27	20	53	1	*p* < 0.001
CNST	0	1	0	0	3	2	0	0	0	0	3	4	*p* < 0.001
ESS	6	3	6	0	17	1	6	3	5	0	15	1	*p* = 0.269
GDS	5	1	5	1	11	1	5	1	5	1	15	3	*p* = 0.270
MoCA	27	2	28	17	30	−1	28	1	28	27	30	0	*p* = 0.007
RBDQ	5	3	4	0	13	1	3	2	2	0	11	1	*p* < 0.001
Upsit-rev	22	8	22	5	43	0	7	5	6	2	26	2	*p* < 0.001
**BIOLOGICS**													
AB _1−42_	885	379	835	239	2572	1	1043	526	941	239	3297	1	*p* = 0.017
CSF a-syn	1488	662	1374	472	5257	2	1698	756	1581	601	4271	1	*p* = 0.004
pTau	14	5	13	8	33	1	17	9	15	8	74	3	*p* = 0.001
tTau	164	56	154	79	345	1	192	81	170	79	581	1	*p* = 0.004
**SOCIODEM**													
Age	61	10	62	34	85	0	61	12	62	31	84	−1	*p* = 0.868
Yrs ed.	16	3	16	5	26	0	16	3	16	8	24	0	*p* = 0.167
**DAT UPTAKE**													
CaudL	2	1	2	0	4	0	3	1	3	1	5	0	*p* < 0.001
CaudR	2	1	2	0	4	0	3	1	3	1	5	0	*p* < 0.001
PutL	1	0	1	0	2	1	2	1	2	1	4	0	*p* < 0.001
PutR	1	0	1	0	3	1	2	1	2	1	4	0	*p* < 0.001
ave. Caud	2	1	2	0	4	0	3	1	3	1	5	0	*p* < 0.001
ave. Put	1	0	1	0	2	1	2	1	2	1	4	0	*p* < 0.001
**MOTOR**													
UPDRS III	22	10	21	5	62	1	1	2	0	0	10	2	*p* < 0.001

Anxiety, STAI trait subscale; CNST, MDS-UPDRS I NP1CNST: 0, none, 1, slight, 2, mild, 3, moderate, 4, severe; CaudL, left caudate; CaudR, right caudate; DAT, dopamine transporter; PutL, left putamen; PutR, right putamen; av. Caud = (left + right caudate)/ 2; av. Put, (left + right putamen)/2; UPDRS III, MDS-UPDRS III total; Upsit-rev (olfactory loss) = University of Pennsylvania Smell Identi-fication Test (a reverse scaled version); AB _1−42_, beta-amyloid _1−42_; CSF a-syn, cerebral spinal fluid α-synuclein; pTau, CSF phosphorylated Tau; tTau, CSF total tau; MoCA, Montreal Cognitive Assessment; Yrs. ed., years of education; PD, early Parkinson's patient data; HC, healthy control data; SocioDem, socio-demographic;

a *robust t-test based on Wilcox, 2005*.

**Table 2:** Descriptive statistics and t-tests, early PD/SWEDD.

***N*, 338**	**Early PD, n, 295: 192 male, 103 female**						**SWEDD, n, 43: 25 male, 18 female**						
	***M***	***sd***	***Mdn***	***min***	***max***	***skew***	***M***	***sd***	***Mdn***	***min***	***max***	***skew***	***t-test^***a***^***
**CLINICAL**													
Anxiety	33	10	31	20	63	1	36	10	32	22	59	1	*p* = 0.237
CNST	0	1	0	0	3	2	0	1	0	0	4	2	*p* = 0.956
ESS	6	3	6	0	17	1	8	5	8	0	19	0	*p* = 0.032
GDS	5	1	5	1	11	1	6	2	5	2	11	1	*p* = 0.299
MoCA	27	2	28	17	30	−1	27	3	27	18	30	−1	p = 0.563
RBDQ	5	3	4	0	13	1	6	3	5	0	13	0	*p* = 0.107
Upsit-rev	22	8	22	5	43	0	10	7	9	2	29	1	*p* < 0.001
**BIOLOGICS**													
AB 1-42	885	379	835	239	2572	1	960	338	982	374	1897	0	*p* = 0.044
CSF a-syn	1488	662	1374	472	5257	2	1654	716	1370	488	4041	1	*p* = 0.188
pTau	14	5	13	8	33	1	15	5	14	8	33	1	*p* = 0.157
tTau	164	56	154	79	345	1	177	56	170	79	344	1	*p* = 0.112
**SOCIODEM**													
Age	61	10	62	34	85	0	60	10	62	39	79	0	p = 0.573
Yrs ed.	16	3	16	5	26	0	15	4	15	8	24	0	p = 0.034
**DAT UPTAKE**													
CaudL	2	1	2	0	4	0	3	1	3	1	4	0	*p* < 0.001
CaudR	2	1	2	0	4	0	3	1	3	1	4	0	*p* < 0.001
PutL	1	0	1	0	2	1	2	1	2	1	3	0	*p* < 0.001
PutR	1	0	1	0	3	1	2	1	2	1	3	0	*p* < 0.001
ave. Caud	2	1	2	0	4	0	3	1	3	1	4	0	*p* < 0.001
ave. Put	1	0	1	0	2	1	2	1	2	1	3	0	*p* < 0.001
**MOTOR**													
UPDRS III	22	10	21	5	62	1	18	11	17	5	45	1	*p* = 0.007

Anxiety, trait subscale from the State-Trait Anxiety Inventory; CNST, constipation based on MDS-UPDRS I;CaudL, left caudate; CaudR, right caudate; PutL, left putamen; DAT, dopamine transporter; PutR, right putamen; av. Caud, (left + right caudate)/ 2; av. Put, (left + right putamen)/2; UPDRS III, MDS-UPDRS III total; Upsit-rev (olfactory loss), University of Pennsylvania Smell Identification Test (a reverse scaled version); AB 1-42, beta-amyloid1-421-421-42 1-42; CSF a-syn, cerebral spinal fluid α-synuclein; pTau, CSF phosphorylated Tau; tTau, CSF total tau; MoCA, Montreal Cognitive Assessment; Yrs. ed., years of education; PD, early Parkinson's patient data; HC, healthy control data; SocioDem, socio- demographic;

a *robust t-test based on Wilcox, 2005*.

**Figure 2:**
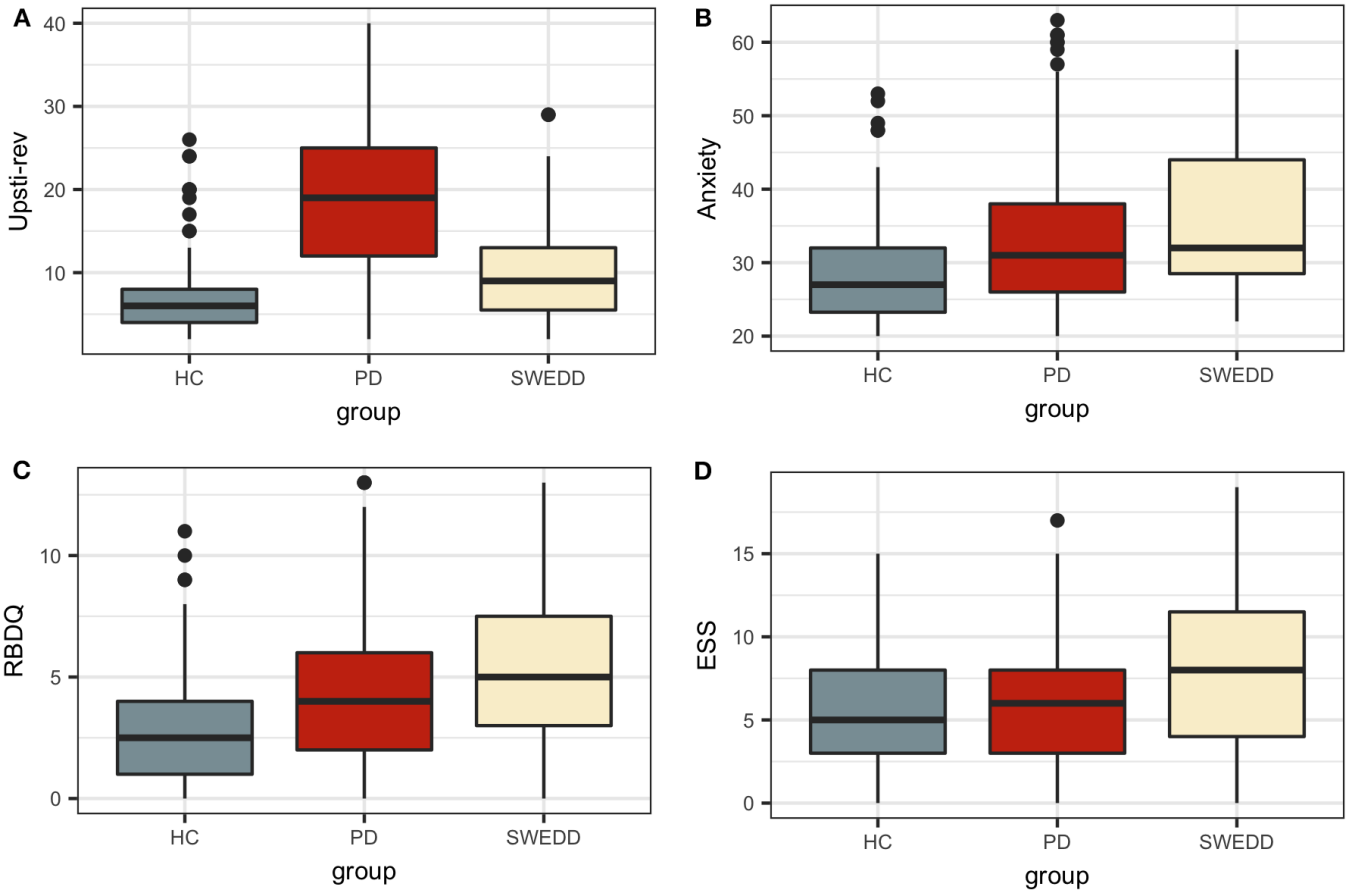
Clinical predictor boxplots. **(A)** Upsit-rev, University of Pennsylvania Smell Inventory Test score reverse-scaled; **(B)** anxiety, trait subscale from the State-Trait Anxiety Inventory; **(C)** RBDQ, rapid eye movement behavior disorder questionnaire; **(D)** ESS, Epworth Sleepiness Scale; HC, healthy controls; PD, early PD; SWEDD, scans without evidence of dopamine deficit.

**Figure 3:**
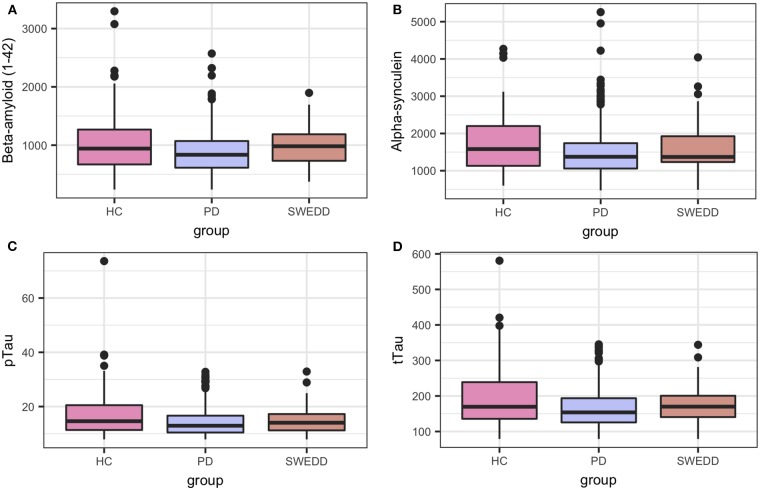
Biologics predictor boxplots. **(A)** beta amyloid Aβ _1−42_; **(B)** alpha-synuclein; **(C)** pTau; and **(D)** tTau. HC, healthy controls; PD, early PD; SWEDD, scans without evidence of dopamine deficit.

Boxplots in Figure 2 visually encapsulate properties (e.g., dispersion) of a few clinical predictors across groups. Hyposmia (Upsit-rev), rapid eye movement behavior disorder questionnaire (58) (RBDQ) and ESS proved to be important model variables. Similarly, the boxplots in Figure 3 characterized cerebral spinal fluid (CSF) biologic variables across groups.

### Bivariate Analyses

**Figure 4:**
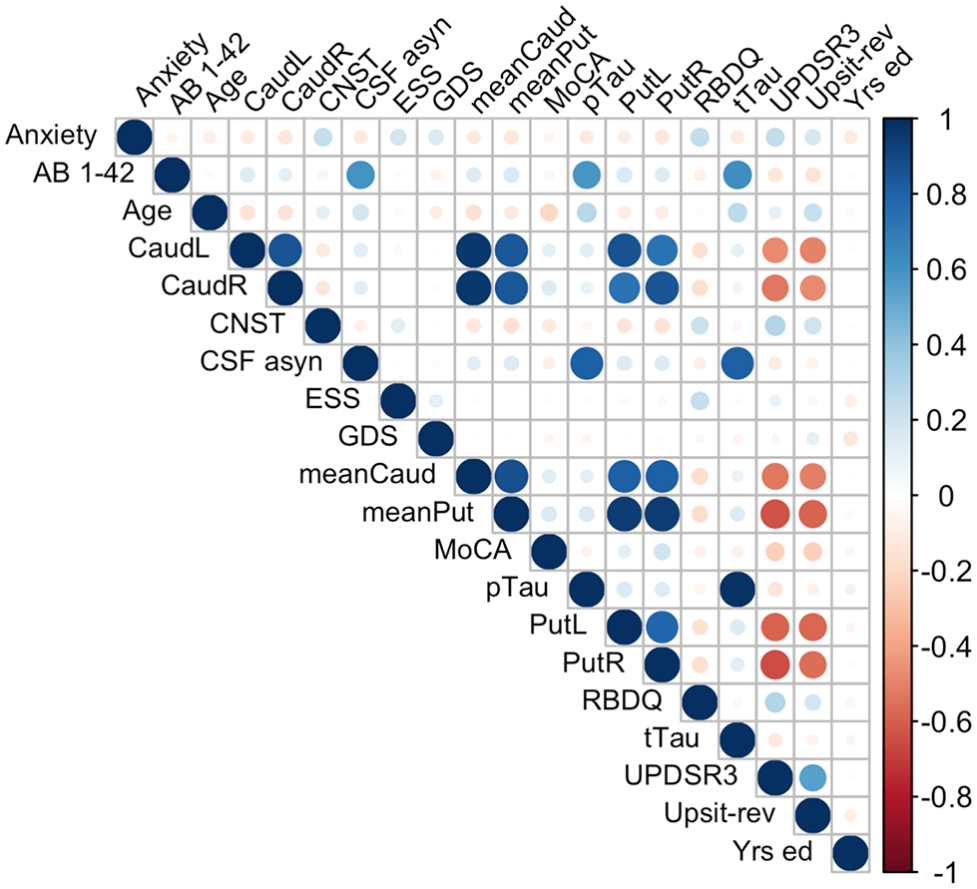
Predictor correlations (Spearman). Circle size is proportional to Spearman correlation; red indicates a negative correlation and blue a positive correlation; Anxiety, trait subscale from the State-Trait Anxiety Inventory; CNST, constipation based on MDS-UPDRS I; CaudL, left caudate; CaudR, right caudate; PutL, left putamen; PutR, right putamen; meanCaud, left + right caudate /2; meanPut, left + right putamen /2; UPDRS3, MDS-UPDRS III; Upsit-rev (hyposmia), University of Pennsylvania Smell Identification Test (a reverse scaled version); A*B* 1-42, beta-amyloid1-42; CSF a-syn, cerebral spinal fluid α-synuclein; pTau, CSF phosphorylated Tau; tTau, CSF total tau; MoCA, Montreal Cognitive Assessment; Yrs. ed., years of education. Note, CaudL, CaudR, PutL, PutR are dopamine transporter (DAT) DAT scan measures.

Because of the largely non-normal distribution of variables, non-parametric Spearman correlations were used rather than Pearson *r*. Figure 4 depicts variable correlations for all data ignoring groups. Circle size in Figure 4 is proportional to the Spearman correlation: larger circles reflect stronger correlations; correlations are color-coded, red indicating a negative correlation and blue indicating a positive correlation. For example, a strong negative association between hyposmia (reverse-scaled UPSIT: Upsit-rev) and DAT scan putamen values is evident; a strong negative association between hyposmia and DAT scan caudate values is also evident. Additionally, strong positive correlations exist among Aβ_1−42_, α-synuclein, p-Tau and t-Tau. A complete correlation table is available in Supporting information V (Table SV-20).

**Figure 5:**
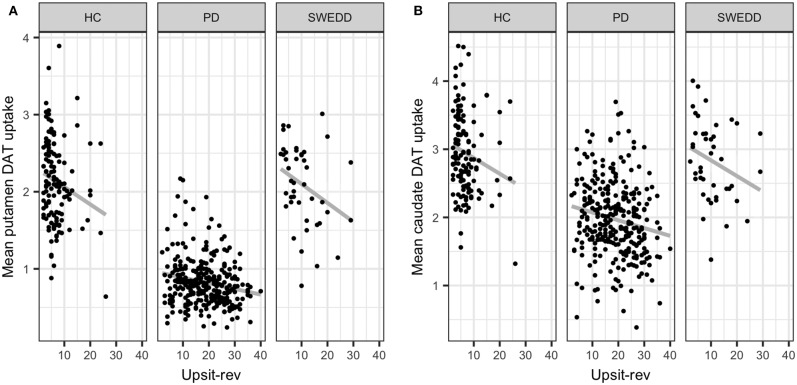
Mean putamen and caudate dopamine transporter (DAT) update against hyposmia. **(A)** mean putamen DAT uptake against Upsit-rev, University of Pennsylvania Smell Test, reverse-scaled; **(B)** mean caudate DAT uptake against Upsti-rev; HC, healthy controls; PD, early PD; SWEDD, scans without evidence of dopamine deficit.

Multicollinearity beyond the cutoff (0.75) was found for pairwise combinations of CSF pTau, tTau and α-synuclein, as such these features were not combined in the same model (see Methods, 2.6 regarding collinearity). The correlation between pTau and tTau was *r*_*s*_= 0.98. The correlations of α-synuclein and the tau proteins were *r*_*s*_= 0.82 for pTau and α-synuclein and *r*_*s*_= 0.81 for tTau and α-synuclein. While several other predictors demonstrated significant correlations (details available on request) these correlations did not exceed 0.75. Figure 4 conveys the finding that both Upsit-rev and MDS-UPDRS III exhibited by far the strongest associations (negative associations) with DAT uptake (bilateral caudate and putamen). DAT uptake and MDS-UPDRS III, not modeled as predictors, are used here only as indices of disease (see Section Statistical Analyses). Figure 4 also indicates linkage of hyposmia (Upsit-rev) and DAT putamen, caudate uptake values, as does Figure 5. Again, olfactory loss or hyposmia is based on reverse-scaled UPSIT scores (i.e., higher values reflect greater hyposmia).

## Model Classification Results

**Figure 6:**
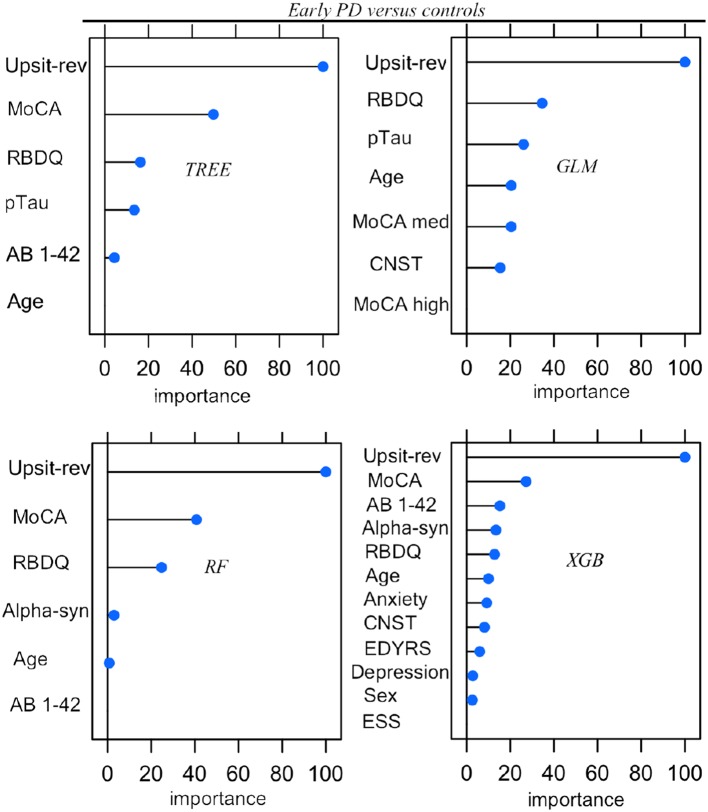
Predictor importance, Early PD vs. controls. *GLM*, logistic regression; *TREE*, decision tree; *RF*, random forest; *XGB*, Extreme gradient boosting; Anxiety, state trait anxiety; AB 1-42, beta amyloid _1−42_; Alpha-syn, CSF α-synuclein; CNST, constipation; ESS, Epworth sleepiness scale; Depression, Geriatric depression scale; MoCA, Montreal Cognitive Assessment; MoCA med, MoCA median; Upsit-rev, reverse scaled Upsit-rev, (UPSIT) University of Pennsylvania Smell Identification Test, reverse-scaled; pTau, CSF phosphorylated Tau; RBDQ, rapid eye movement behavior disorder questionnaire; Sex, gender. Predictors ranked on a 0 to 100% scale of importance.

**Figure 7:**
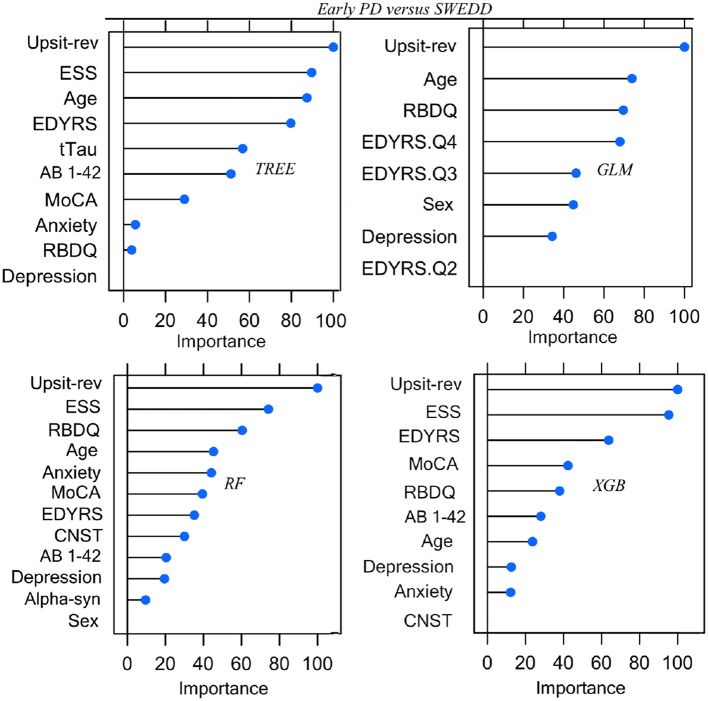
Predictor Early PD vs. SWEDD. *GLM*, logistic regression; *TREE*, decision tree; *RF*, random forest; XGB, Extreme gradient boosting; Anxiety, state trait anxiety; AB 1-42, beta amyloid _1−42_; Alpha-syn, CSF α-synuclein; CNST, constipation; Depression, Geriatric depression scale; EDYRS Q2, lower quartile for number of education years; EDYRS Q3, upper quartile for number of education years; EDYRS Q4, number of education years of those exceeding the upper quartile; ESS, Epworth sleepiness scale; GDS, Geriatric depression scale; MoCA, Montreal Cognitive Assessment; Upsit-rev, University of Penn-sylvania Smell Identification Test, reverse-scaled;RBDQ, rapid eye movement behavior disorder questionnaire; Predictors ranked on a 0 to100% scale of importance.

Parameters (for logistic regression) and hyper-parameter settings are detailed in Supporting information V. As outlined in Methods (Feature Elimination and Hyper-Parameter Tuning) features, selected only from training set data, were determined by model-specific (e.g., stepwise feature elimination using AIC in regression) or built-in (e.g., mean Gini decrease in random forest) feature elimination, with the final feature set determined by the combination of features resulting in the highest AUC. Summary graphs in Figures 6, 7 are based on caret generic feature of importance ranking (56): it conveniently ranks the import of predictors of the different model types on the same common 0–100% scale but utilizes model-specific information and can incorporate between predictor correlation into calculation of feature importance. Figure 6 pertains to the early PD/control classification ranking of features of importance; Figure 7 pertains to early PD/SWEDD classification ranking of feature of importance. The caret genetic ranking of features and the model-built-in ranking were identical for the top ranked feature (hyposmia: Upsit-rev), similar for the top 2nd and 3rd ranked features but ranking typically varied to some extent for lower ranked predictors. See the individual model feature importance tables in Supporting information V for details. The GLM ranking of features by coefficient z-scores was identical to the caret generic feature ranking. The GAM features of importance are not shown in Figures 6 and 7. The GAM model used the same predictors as the logistic regression model (see GLM in Figures 6, 7) but the rank of features to classification, with the exception of hyposmia, differed. In descending order of importance the rank of features to GAM early PD/control classification was hyposmia, RBDQ, age, pTau, constipation, and MoCA. In descending order of importance the rank of features to GAM early PD/SWEDD classification was Upsit-rev, RDBQ, age, years of education, gender, and depression. Overall, hyposmia was the top ranked predictor of importance and RBD was consistently of high rank for all models in both the early PD/control and early PD/SWEDD classification analyses. Otherwise there was variation in model feature selection and feature ranking between classification analyses, including variation within the same model types across early PD/control and early PD/SWEDD analyses.

The model performance results (from models applied to test validation data unseen by models during training) are summarized in Table 3. The AUC, accuracy, Kappa statistic, sensitivity and specificity outcomes are listed. Table 3 superscript notation reflects tree-model k-fold resampling of tuning parameters and if subsampling (i.e., synthetic minority oversampling technique [SMOTE]) was used. SMOTE was used only to augment the early PD/SWEDD training data.

**Table 3:** Performance summary.

**Models**	**Train**			**Test (cross-validation)**					
***Metric***	**AUC (95% CI)**	**SN**	**SP**	**Opt.Thr**	**AUC (95% CI)**	**ACC**	**Kappa**	**SN**	**SP**
**EARLY PD VS. HC**									
GLM	0.920 (0.888-0.953)	0.912	0.812	0.462	0.907 (0.849-0.964)	0.898	0.764	0.909	0.872
**GAM**	0.946 (0.922-0.970)	0.923	0.850	0.534	**0.928** (0.878-0.978)	0.898	0.768	0.898	0.897
Tree^a^	0.872 (0.831-0.913)	0.857	0.879	0.586	0.860 (0.799-0.922)	0.842	0.659	0.818	0.897
RF^a^	0.999 (0.999-1.00)	0.990	1.00	0.534	0.913 (0.858-0.968)	0.898	0.764	0.909	0.872
XGB^a^	0.958 (0.937-0.979)	0.898	0.901	0.660	0.923 (0.875-0.972)	0.882	0.736	0.875	0.897
**EARLY PD VS. SWEDD**									
GLM^b^	0.938 (0.863-0.972)	0.909	0.841	0.504	0.779 (0.677-0.880)	0.744	0.265	0.667	0.755
GAM^b^	0.955 (0.916-0.994)	0.886	0.909	0.437	0.787 (0.689-0.886)	0.756	0.299	0.714	0.762
Tree^a, b^	0.932 (0.894-0.971)	0.864	0.920	0.486	0.743 (0.617-0.869)	0.798	0.343	0.667	0.816
RF^a, b^	1.00 (1.00-1.00)	1.00	1.00	0.461	0.822 (0.746-0.899)	0.732	0.302	0.809	0.721
**XGB**^a, b^	0.997 (0.993-1.00)	0.977	0.954	0.542	**0.863** (0.777-0.948)	0.768	0.381	0.905	0.748

*Superscript a, 10-fold, 5 repeats resampling of the model tuning parameter(s), whereby the optimal hyper-parameter setting was determined by the AUC; ACC, accuracy; superscript b, synthetic minority oversampling technique (SMOTE); AUC, receiver operating characteristic area under the curve; CI, DeLong confidence interval; Kappa, Cohen's Kappa; SP, specificity; SN, sensitivity; GAM, general additive model; GLM, logistic regression generalized linear model; RF, random forest; Tree, decision tree; XGBoost, Extreme gradient boosting; thr, threshold; Bold model names, highest cross-validated AUC*.

**Figure 8:**
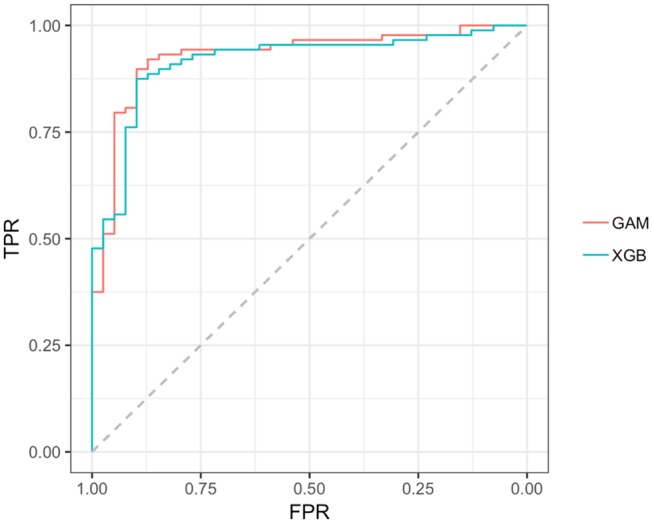
AUC, GAM, and XGBoost. GAM AUC =0.928; XGB AUC =0.916;GAM, general additive model; XGB, Extreme gradient boosting model; TPR, true positive rate (sensitivity); FPR, the False positive rate (1–specificity).

Reviewing the early PD/control results first, all models achieved an early PD/control classification AUC of >80%. Three pairwise AUC tests were run, which was sufficient to gain a comparative perspective on model early PD/control cross-validated (CV) AUC scores. Using Bonferroni correction for family-wise error, and rounding two figures, α was set at.02 (0.05/3 = 0.0167) to control for family-wise error. A modified (74) bootstrap (*n*, 2,000) test (80) was used for AUC pairwise comparisons of correlated ROC curves. All models except the GLM (CV AUC.907) had significantly higher AUC values (*p* < 0.01) relative to the decision tree model CV AUC (0.860), but there was not a significant AUC difference among the GAM, GLM, random forest and XGBoost models (*p* > 0.01). The GAM and XGBoost models were the highest performing early PD/control classifiers (see Table 3). The AUC of both models is graphed in Figure 8.

**Figure 9:**
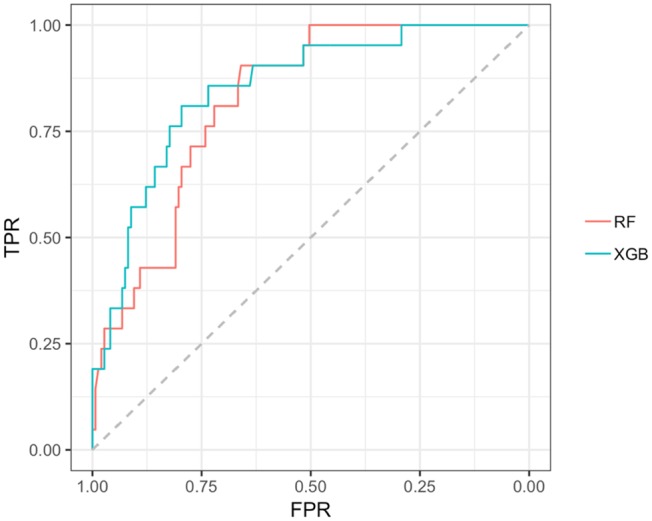
AUC, Random forest and XGBoost. RF AUC = 0.822. (sensitivity 0.809; Specificity 0.721); XGB AUC =0.863 (sensitivity 0.905; specificity 0.743); RF, random forest; XGB, Extreme gradient boosting model; TPR, true positive rate (sensitivity); FPR, the False positive rate (1–specificity).

In the early PD/SWEDD CV results, model classification performance metrics were lower relative to those in the early PD/control analysis. XGBoost and random forest were the most efficient early PD/SWEDD classifiers (see Table 3). In the comparison of correlated ROC curves (74), the XGBoost AUC (0.863), the highest CV AUC outcome in the early PD/SWEDD analysis, was not significantly different from the lowest CV AUC from the decision tree model (0.743), *D* = 1.89, *p* = 0.06. Other models were not significantly different from either the decision tree or XGBoost CV AUC outcomes (*p* > 0.01). The early PD/SWEDD CV AUC of the random forest and XGBoost models is provided in Figure 9.

### Model Prediction and Long-Term Diagnosis

The GAM and XGBoost models were the best performing (highest AUCs) early PD/control classifiers (see Table 3 and Figure 8). These models were applied to the SWEDD/control validation data (SWEDD: *n*= 43, 25 male; controls: *n* = 39 controls, 26 male) to assess usefulness of early PD/control models to predict SWEDD to PD conversion. The control data was the same controls test set used for early PD/control model cross-validation. The GAM model achieved an AUC of 0.863 (optimal cut-off 0.389: sensitivity =0.814; specificity =0.846) distinguishing SWEDD from controls. In the available longitudinal PPMI curated 12–24 months data, 12/38 (32%) GAM model SWEDD cases predicted to be PD-like were diagnosed as idiopathic PD. However, the majority of GAM model PD-like cases, 26/38 (68%), were not diagnosed as idiopathic PD longitudinally but rather were a mix of almost a dozen non-PD pathologies (e.g., essential tremor, psychogenic illness, etc.) and apparently normal cases. Four of those predicted by the model to convert to idiopathic PD (4/12) and re-diagnosed by 24 months as PD had DAT scan evidence of likely dopaminergic dysfunction as suggested by relatively low mean putamen DAT scan values (*M* = 1.24, *SD* = 0.73). The mean putamen value of 26/38 predicted by the model to convert from SWEDD to PD but that converted to status other than PD was 2.01 (*SD* = 0.47), and the mean putamen value of the model predicted control cohort was 2.14 (*SD* = 0.57). The 24-months time point diagnosis data also indicated that none of the 33 model predicted controls converted to PD.

The XGBoost model applied to SWEDD/control validation data achieved an AUC of 0.831 (optimal cut-off 0.378: sensitivity = 0.837; specificity = 0.769) distinguishing SWEDD from controls. In the available longitudinal PPMI curated 12–24 months data, 13/43 (30%) SWEDD predicted by the XGBoost model to be PD-like had converted from SWEDD to idiopathic PD. But, as with the GAM model, the majority of XGBoost model PD-like data instances, 34/43 (79%), were not classified in the 12–36 months longitudinal data as idiopathic PD but diagnosed as a collective of diverse disorders similar to those noted for the GAM model. The XGBoost model predicted conversions also included the same four SWEDD subjects found by the GAM model with lower DAT scan values suggestive of dopaminergic dysfunction. The mean putamen value of the remaining 20/34 (59%) cases predicted by the model to convert to PD but that converted to status other than control or PD was 2.05 (*SD* = 0.49); the mean putamen value of the controls was 2.09 (*SD* = 0.57). As with the GAM model, none of the model predicted controls was diagnosed at 12–24 months as PD.

Longitudinal curated diagnoses available for the two top performing early PD/SWEDD classifiers, random forest and XGBoost (see Table 3 and Figure 9), demonstrated again the largely non-PD diversity of pathologies that constitute the SWEDD category noted in the preceding paragraph. Here, however, the main interest was estimating long-term model accuracy or fidelity in terms of estimated model long-term sensitivity (percentage of model classification non-PD SWEDD matching curated long-term diagnosis) and specificity (percentage model classification of PD matching curated long-term diagnosis). At its optimal cutoff (0.461), random forest long-term sensitivity amounted to 12/16 (75%) correctly predicted non-PD SWEDD cases that matched the 12–24 months curated diagnoses records available. The mean putamen DAT scan value averaged across all (non-PD) SWEDD at 12–36 months was 2.06 (*SD* = 0.50). Random forest model-approximated long-term specificity amounted to 92/128 (71.87%) cases (true negatives) correctly classified by the model as PD at 12–24 months. The mean putamen DAT scan value of these PD confirmed cases, averaged across all PD cases at 12–36 months, was 0.69 (*SD* = 0.27). For XGBoost, and at its optimal cutoff (0.542), long-term model sensitivity to non-PD SWEDD amounted to 13/16 (81.25%) class predictions that correctly matched 12–24 months curated diagnoses. The mean putamen DAT scan value averaged across all (non-PD) SWEDD at 12–36 months was 2.0 (*SD* = 0.50). XGBoost long-term specificity amounted to 97/128 (75.78%) cases (model true negatives) correctly classified by as PD. The mean putamen DAT scan value of the PD classified segment, averaged across all PD diagnosed cases at 12–36 months, was 0.70 (*SD* = 0.31).

## Discussion

Unique to the current work was the particular set of five classifiers used and the dual early PD/control and early PD/SWEDD analyses approach adopted. There is never a guarantee that one model type will outperform another (47). By comparing several classifiers, here five, we were able to determine the optimal model for the data, and the optimal model differed for the early PD/control relative to the early PD/SWEDD classification analyses. The GAM was top performing early PD/control classifier, and the XGBoost model was the top performing early PD/SWEDD classifier. Overall, the XGBoost model had the most consistent classification performance, achieving the second highest performance in the early PD/control analysis and the highest early PD/SWEDD outcome (see Table 3 for details). Moreover, as made apparent in Figures 6, 7, conducting the dual classification analysis revealed differential importance of certain features to early PD/SWEDD vs. early PD/control discrimination. Notably, Epworth sleepiness scale (ESS) and years of education figured as prominent features of import to early PD/SWEDD classification but were of little to no consequence to early PD/control classification. In both classification analyses hyposmia (based on the University of Pennsylvania Smell Test—reverse scaled) was inevitably the single most important feature to model classification. Rapid eye-movement behavior disorder questionnaire (RBDQ) was the next most common feature of relatively high rank importance to classification for all models in both analyses (MoCA was also quite consistently important to classification). Biomarkers CSF α-synuclein and pTau were features of greater importance to early PD/control classification than to early PD/SWEDD classification, and age assumed greater importance in early PD/SWEDD classification.

Predictive model results from recent studies using Parkinson's Progressive Markers Initiative (PPMI) data incorporating either clinical and genetic risk (41) or clinical variables and biologics (43) achieved high early PD vs. control AUC scores without including an imaging (DAT scan) predictor: 0.923 (sensitivity 83.4%; specificity 90.3%) (41); 0.927 (sensitivity 89.7%, specificity 80.4 %) (43). Predictive models in the current work, also incorporating PPMI clinical and biologics (not genetic risk) data, achieved similarly high AUC scores discriminating early PD vs. control. The two top performing models, as already noted, were the GAM and XGBoost classifiers. The GAM model had an AUC of 0.946 (sensitivity 91.3%; specificity 80.7%) and the XGBoost model an AUC of 0.958 (sensitivity 93.7%, specificity 83.5%). The Nalls et al. (41) and Yu et al. (43) studies both used logistic regression. Comparing apples to apples, our logistic regression model had an AUC of 0.920 (sensitivity 91.2%, specificity 81.2%). The marginally lower logistic regression model AUC we obtained was due in part to a smaller training set: we divided the PPMI data, using random stratification, into train and test sets while the above referenced studies used all the early PD/control PPMI data to train models and validated models in different cohort data sets. In addition, our stringent data filtering for only complete cases across 14 variables resulted in further data instance reduction. Moreover, while hyposmia (based on the UPSIT scale) and age were common features selected by the logistic regression stepwise process among the Nalls et al., Yu et al. and the current work, feature elimination in our study otherwise resulted in a different final set of predictors. The Nalls study, which included genetic risk, not part of our study, used five features: hyposmia, genetic risk, family history, age and gender. The Yu et al. study used hyposmia, age, CSF α-synuclein and gender. The logistic model stepwise (AIC) feature elimination procedure in our study determined hyposmia, rapid eye-movement behavior disorder questionnaire (RBDQ), pTau, age, MoCA and constipation as the most important features to early PD/control classification. With respect to gender in our study, and SWEDD test data in particular, the random stratified split of SWEDD data into train and test sets left gender under represented. However, in the early PD/SWEDD as well as the early PD/control classification gender was a feature of low or no importance. Further, Yu et al. commented that their model's outcome was similar whether or not gender was included.

Note, to avoid collinearity exceeding *r*_*s*_ *0*.75 neither pTau jointly with tTau, nor α-synuclein and either pTau or tTau were used concurrently in the same model. The correlations of α-synuclein and pTau and α-synuclein and tTau were *r*_*s*_= 0.82 and *r*_*s*_= 0.81, respectively. The correlation between pTau and tTau was *r*_*s*_= 0.98 (see Section Bivariate analyses). We adopted the relatively low collinearity cut off of 0.75, sensitive to pairwise correlations (71), to prioritize unbiased feature selection and classification consistency for all models (69, 70) (see Statistical analyses).

Another recent study also using PPMI data reported features important to early PD/control classification including hyposmia, RBDQ, CSF α-synuclein, pTau, tTau, and notably DAT scan values (46). The DAT scan values (striatal binding ratios for the left and right caudate and putamen) made the greatest contribution to model performance and further heightened AUC scores to >0.98 for all five models in the latter study. However, like the Nalls and Yu analyses we did not include SPECT DAT scan values as predictors. SPECT imaging is not always accessible and a single scan can be costly (typically over $1,800 in the US).

Importantly, our early PD vs. control classification models applied to validation data, unseen by models during training (the cross-validated [CV] outcome), achieved high classification accuracy. The highest performing model, the GAM, had a CV AUC of 0.928 (at the optimal threshold of 0.534, sensitivity 89.9%, specificity 89.7%). The second highest CV AUC from the XGBoost model was 0.923 (at the optimal threshold of 0.660, sensitivity 87.5%, specificity 89.7%). Overall, and as hypothesized, the non-motor clinical and biologic features used achieved >0.80% AUC classification accuracy across all models (decision tree, logistic regression, general linear, random forest, and XGBoost), a level of consistency supporting the validity and reliability of these features to differentiate early stage PD pathology from age-matched normal healthy subjects with relatively high classification accuracy. This consistency, across all models adds to the growing body of research (30, 40, 41, 43, 46) demonstrating the usefulness of non-motor clinical and biomarker features in early stage PD discrimination. In addition, the AUC of all models with the exception of decision tree were very similar. The decision tree model had a significantly lower AUC of 0.860 (at the optimal threshold of 0.586, sensitivity 81.8%, specificity 89.7%) compared to the other four model types. The logistic regression model (GLM) offered, arguably, the best blend of simplicity, parsimony of predictors and performance. In addition, as a parametric model, it had the benefit of quantifying predictor contribution to the model (e.g., see model coefficients in Supporting information V). But with a non-linear feature–logit relation, exemplified by the MoCA feature in the early PD/control classification, the GAM, random forest or XGBoost models may be more appropriate.

We had also posited that outcome of the second classification analysis involving early PD vs. SWEDD discrimination would be less definitive and typified by lower AUC results for all models. This also proved true. Results for both early PD/control and early PD/SWEDD classification analyses are provided in Table 3. The discrepancy of model performance between early PD/control and early PD/SWEDD classification is, at least in part, due to the wide range of disorders encompassed by the SWEDD category. The diversity of clinical entities within the SWEDD category, reported in other research (35, 36, 81, 82), was evident in current study longitudinal findings, where SWEDD proved to be largely a mix of almost a dozen clinical entities (Alzheimer's disease case, polyneuropathy, lateral sclerosis, essential tremor, psychogenic illness, apparently normal etc.). The heterogeneity of the SWEDD category, in general adds complexity and confusion to PD pathology differentiation. Indeed, removal of the term or category SWEDD, as currently conceptualized, has been recommended (35, 36).

Developing a model (s) to disentangle non-PD SWEDD cases from actual cases of early PD pathology was one objective of our study. The two top performing early PD/SWEDD classifiers, XGBoost and random forest, were able to discriminate non-PD pathology SWEDD from early PD with moderate sensitivity to detect non-PD SWEDD cases. From random forest results 12/16 (75%) SWEDD non-PD predicted cases matched the SWEDD non-PD case diagnoses in PPMI curated 12–24 months (available) records. From XGBoost results 13/16 (81.25%) SWEDD non-PD predicted cases matched the SWEDD non-PD case diagnoses in the 12–24 months records. The random forest long-term specificity (to early PD) amounted to 92/128 (71.87%) cases matching the 12–24 months available diagnoses; XGBoost long-term specificity amounted to 97/128 (75.78%) cases matching the 12–24 months available diagnoses. These results suggest that either model could be useful to help differentiate non-PD SWEDD category patients from those with actual incipient PD pathology.

In a brief review of descriptive statistics, including the UPDRS III and DAT scan putamen and caudate values not used in models, compared to healthy controls we observed more severe hyposmia, rapid eye-movement behavior disorder (questionnaire-based [RBDQ]), anxiety traits, and constipation in early PD compared to healthy controls. Montreal cognitive assessment (MoCA) scores were also lower for the early PD cohort as were caudate and putamen DAT scans from the early PD cohort compared to controls. As might be expected UPDRS III scores were also much higher, typical of PD, and DAT scan caudate and putamen values lower in early PD compared to controls. Comparing SWEDD to early PD, hyposmia was more severe for early PD, Epworth sleepiness scale (ESS) was higher (worse) for SWEDD, and there were fewer years of education for SWEDD. T-tests (63) demonstrated significant early PD/control and early PD/SWEDD differences for all of these variables (see Tables 1, 2), findings consistent with prior research (40). Also in agreement with other research (31, 40), we observed significantly reduced cerebral spinal fluid biomarker values of Aβ_1−42_, α-synuclein, pTau and tTau in early PD compared to healthy controls. In addition, we found significantly increased Aβ_1−42_ in SWEDD compared to early PD, and while this agreed with findings from Marek et al. (40), contrary to the latter study we did not find significantly differing α-synuclein between early PD and SWEDD (see Tables 1, 2). Finally, in agreement with other research (30, 83), moderate to high correlations (*r*_*s*_ > 0.75) were found among CSF α-synuclein, pTau and Ttau.

The median age in the PPMI data used in the current work was 62, which along with other PPMI demographic data (education, ethnicity, and gender) is consistent with clinical trial demographics (84–86). Age, though, poses the single highest risk factor for neurodegenerative diseases such as idiopathic PD (87). Further, as there is an age related increase in hyposmia (88) for instance, age is a variable with increasing confounding potential in more elderly cohorts (e.g., 85+). We found age was positively correlated with hyposmia (higher age was associated with more severe hyposmia), though the correlation was well under the 0.75 limitation set (*r*_*s*_ =0.22, *p* < 0.001). Also, including age in all our models controlled for this variable. However, while age in age-matched groups can be controlled for in the statistical sense, classifiers trained on younger cohorts would, in general, help to isolate the importance of features related to diagnosis of PD neurodegeneration independent of age.

As reported in the first paragraph of this discussion, Epworth sleepiness scale (ESS) in particular but also years of education were important features to early PD/SWEDD but not to early PD/control discrimination (see Figures 6, 7). Both features also significantly differed between early PD and SWEDD but not early PD and controls (see Tables 1, 2). These findings, in concert with other PPMI data research (40, 89), warrant further investigation. Is the difference in years of education, fewer years of education in SWEDD, just specific to the particular SWEDD cohort used? If not, how does more extensive education relate to PD pathology? With respect ESS, an even more important early PD/SWEDD group differentiator, a question to be probed is how does dozing-off in certain situations (ESS measures dozing-off rather than fatigue) relate differently to the non-PD clinical entities of SWEDD compared to early PD?

It warrants note that hyposmia, the main model driver here as in other research (41, 43) and of secondary import only to DAT scan imaging in yet another study (46), is not specific to PD pathology (59, 90–93). It has been suggested that CSF α-synuclein, which is synucleinopathy-specific, may increase specificity for PD-type pathology when combined with other features (e.g., hyposmia) in a model (43). But if so, it is critical to first determine the species of α-synuclein specific to PD pathology. While variations of glia-to-glia, glia-to-neuron and neuron-to-neuron spread of α-synuclein are likely (94–96), the form of this toxic misfolded protein to be targeted for diagnostic and prognostic purposes remains to be established: α-synuclein monomers, oligomers or the misfolded fibril? A recent study demonstrated that α-synuclein fibrils injected into the mouse brain acted as agents recruiting monomeric endogenous α-synuclein and induced PD indicators including loss of substantia nigra pars compacta and striatal dopamine terminals as well as dysfunctional motor behavior (97). However, the root cause may involve an oligomer pre-fibril state. For reviews on this subject see Mead et al. (98) and Xu and Pu (99).

## Conclusion

We undertook dual early PD/control, early PD/SWEDD classification analyses to broaden understanding of non-motor clinical and biomarker feature utility to discriminate preclinical, early PD. In agreement with other research, hyposmia, RBD, and CSF biomarkers distinguished early PD vs. controls with high classification performance. Indeed, as a testament to the classification efficacy of features used, we demonstrated that five different models could achieve >0.80% AUC cross-validated classification accuracy without imaging or motor predictors. Most distinctive in the current work however, was the dual binomial classification approach. Relative to early PD/control results, early PD/SWEDD model classification performance was lower (for all models), the optimally performing model-type differed, and, with the exception of hyposmia, there was variation in feature selection or rank of features by models for early PD/control compared to early PD/SWEDD analyses- informative findings that justified the dual analysis approach. Moreover, data at 12–36 months from baseline indicated longitudinal model sensitivity of up to about 81% to distinguish non-PD SWEDD cases from PD pathology. The model may be useful to screen SWEDD category patients with actual incipient PD pathology from those non-PD SWEDD category patients. Without such screening, the heterogeneity in the SWEDD category will diminish the capacity of future models to link to and discriminate PD pathology.

## Limitations

After filtering for only completed cases, and only cases meeting our screening criteria (e.g., exclusion of samples with >200 ng/ml hemoglobin levels), data sets were quite small, particularly the SWEDD validation data-set. However, with respect to PD/SWEDD data, SMOTE subsampling augmented training data instances while also balancing groups. It should be mentioned that ratios of biomarkers or (biomarkers and clinical variables) were not included in the current work, and would have added more depth to evaluations. In addition, a multinomial rather than binomial approach could have been used. However, in respect to the latter, most current classification research has used the binomial approach, which facilitates comparison among study outcomes.

## Data Availability Statement

All datasets used are available on-line (https://www.frontiersin.org/articles/10.3389/fneur.2020.00364/full#supplementary-material) or from the corresponding author (cslfalcon@gmail.com; or charlie9@yorku.ca)

## Ethics Statement

PPMI subjects provided written, informed consent to participate and all PPMI study aspects were in keeping with the Helsinki accord (https://www.ncbi.nlm.nih.gov/pmc/articles/PMC6292383/).

## Author Contributions

The authors (CL, MH, and JD) contributed to differing research related tasks. For example, models initially executed by CL where all independently re-run and vetted by MH ensuring complete reproducibility. JD monitored the overall adherence to original aims and scope from initial to the final paper version. All authors contributed to final proofing of this work.

## Conflict of Interest

The authors declare that the research was conducted in the absence of any commercial or financial relationships that could be construed as a potential conflict of interest.

## Supplementary Material

All data used is available from https://github.com or the corresponding author: charlie9@yorku.ca.

Data Sheet 1 Supporting Information I-V.

Data Sheet 2 Data sets.

Data Sheet 3 R-code.

## Abbreviations

Aβ_1−42_: Amyloid beta _1−42_
CSF: Cerebral spinal fluid
DAT: Dopamine transporter
ESS: Epworth sleepiness scale
GAM: General additive model
MoCA: Montreal cognitive assessment
PD: Parkinson's disease
pTau: Phosphorylated tau_181_
RDB: Rapid eye-movement behavior disorder
RBDQ: Rapid eye-movement behavior disorder questionnaire
REML: Restricted maximum likelihood
PPMI: Parkinson's Progression Marker Initiative
SMOTE: Synthetic minority oversampling technique
SPECT: Single-photon emission computed tomography
SWEDD: Scans without evidence of dopamine deficit
Tp: thin plate smoother function
Tree: Decision tree model
tTau: Total tau
XGBoost: Extreme gradient boosting model.
