# Supplementary material for: Non-motor Clinical and Biomarker Predictors Enable High Cross-Validated Accuracy Detection of Early PD but Lesser Cross-Validated Accuracy Detection of Scans Without Evidence of Dopaminergic Deficit
Source: Front Neurol. 2020 May 11;11:364. doi: 10.3389/fneur.2020.00364 (PMC7232850; doi:10.3389/fneur.2020.00364)
Supplement: Data Sheet 1 — Supporting Information I-V. [file Data_Sheet_1.ZIP › Appendices/Supplorting Information III.docx]

*Supporting Information III: confusion matrices*

| Confusion Matrix and Statistics: **decision Tree**, early PD/control | Confusion Matrix and Statistics: **random forest**, early PD/control |
| --- | --- |
|  |  |
| Reference | Reference |
| Prediction HC PD | Prediction HC PD |
| HC 35 16 | HC 34 8 |
| PD 4 72 | PD 5 80 |
|  |  |
| Accuracy : 0.8425 | Accuracy : 0.8976 |
| 95% CI : (0.7673, 0.9011) | 95% CI : (0.8313, 0.9444) |
| No Information Rate : 0.6929 | No Information Rate : 0.6929 |
| P-Value [Acc > NIR] : 8.426e-05 | P-Value [Acc > NIR] : 3.758e-08 |
|  |  |
| Kappa : 0.6592 | Kappa : 0.7645 |
| Mcnemar's Test P-Value : 0.01391 | Mcnemar's Test P-Value : 0.5791 |
|  |  |
| Sensitivity : 0.8182 | Sensitivity : 0.9091 |
| Specificity : 0.8974 | Specificity : 0.8718 |
| Pos Pred Value : 0.9474 | Pos Pred Value : 0.9412 |
| Neg Pred Value : 0.6863 | Neg Pred Value : 0.8095 |
| Prevalence : 0.6929 | Prevalence : 0.6929 |
| Detection Rate : 0.5669 | Detection Rate : 0.6299 |
| Detection Prevalence : 0.5984 | Detection Prevalence : 0.6693 |
| Balanced Accuracy : 0.8578 | Balanced Accuracy : 0.8904 |
|  |  |
| 'Positive' Class : PD | 'Positive' Class : PD |

| Confusion Matrix and Statistics: **GLM** early PD/controls | Confusion Matrix and Statistics: **GAM**  early PD/controls |
| --- | --- |
| Reference | Reference |
| Prediction HC PD | Prediction HC PD |
| HC 34 8 | HC 35 9 |
| PD 5 80 | PD 4 79 |
|  |  |
| Accuracy : 0.8976 | Accuracy : 0.8976 |
| 95% CI : (0.8313, 0.9444) | 95% CI : (0.8313, 0.9444) |
| No Information Rate : 0.6929 | No Information Rate : 0.6929 |
| P-Value [Acc > NIR] : 3.758e-08 | P-Value [Acc > NIR] : 3.758e-08 |
|  |  |
| Kappa : 0.7645 | Kappa : 0.7678 |
| Mcnemar's Test P-Value : 0.5791 | Mcnemar's Test P-Value : 0.2673 |
|  |  |
| Sensitivity : 0.9091 | Sensitivity : 0.8977 |
| Specificity : 0.8718 | Specificity : 0.8974 |
| Pos Pred Value : 0.9412 | Pos Pred Value : 0.9518 |
| Neg Pred Value : 0.8095 | Neg Pred Value : 0.7955 |
| Prevalence : 0.6929 | Prevalence : 0.6929 |
| Detection Rate : 0.6299 | Detection Rate : 0.6220 |
| Detection Prevalence : 0.6693 | Detection Prevalence : 0.6535 |
| Balanced Accuracy : 0.8904 | Balanced Accuracy : 0.8976 |
|  |  |
| 'Positive' Class : PD | 'Positive' Class : PD |
|  |  |

| Confusion Matrix and Statistics: **XGBoost,**  early PD/controls |
| --- |
|  |
| Reference |
| Prediction HC PD |
| HC 35 11 |
| PD 4 77 |
|  |
| Accuracy : 0.8819 |
| 95% CI : (0.8127, 0.9324) |
| No Information Rate : 0.6929 |
| P-Value [Acc > NIR] : 4.799e-07 |
|  |
| Kappa : 0.7357 |
| Mcnemar's Test P-Value : 0.1213 |
|  |
| Sensitivity : 0.8750 |
| Specificity : 0.8974 |
| Pos Pred Value : 0.9506 |
| Neg Pred Value : 0.7609 |
| Prevalence : 0.6929 |
| Detection Rate : 0.6063 |
| Detection Prevalence : 0.6378 |
| Balanced Accuracy : 0.8862 |
|  |
| 'Positive' Class : PD |

***PD/Control model applied to SWEDD patients***

| Confusion Matrix and Statistics | Confusion Matrix and Statistics |
| --- | --- |
| **GAM model** applied to SWEDD | XGBoost model applied to SWEDD |
| Reference | Reference |
| Prediction HC SWEDD | Prediction HC SWEDD |
| HC 33 8 | HC 30 7 |
| SWEDD 6 35 | SWEDD 9 36 |
|  |  |
| Accuracy : 0.8293 | Accuracy : 0.8049 |
| 95% CI : (0.7302, 0.9034) | 95% CI : (0.7026, 0.8842) |
| No Information Rate : 0.5244 | No Information Rate : 0.5244 |
| P-Value [Acc > NIR] : 7.367e-09 | P-Value [Acc > NIR] : 1.209e-07 |
|  |  |
| Kappa : 0.6585 | Kappa : 0.6079 |
| Mcnemar's Test P-Value : 0.7893 | Mcnemar's Test P-Value : 0.8026 |
|  |  |
| Sensitivity : 0.8140 | Sensitivity : 0.8372 |
| Specificity : 0.8462 | Specificity : 0.7692 |
| Pos Pred Value : 0.8537 | Pos Pred Value : 0.8000 |
| Neg Pred Value : 0.8049 | Neg Pred Value : 0.8108 |
| Prevalence : 0.5244 | Prevalence : 0.5244 |
| Detection Rate : 0.4268 | Detection Rate : 0.4390 |
| Detection Prevalence : 0.5000 | Detection Prevalence : 0.5488 |
| Balanced Accuracy : 0.8301 | Balanced Accuracy : 0.8032 |
|  |  |
| 'Positive' Class : SWEDD | 'Positive' Class : SWEDD |

| Confusion Matrix and Statistics | Confusion Matrix and Statistics |
| --- | --- |
| GAM model applied to SWEDD |  |
| Reference | Reference |
| Prediction SWEDD PD | Prediction HC PD |
| SWEDD 21 73 | SWEDD 28 15 |
| PD 22 15 | PD 15 73 |
|  |  |
| Accuracy : 0.2748 | Accuracy : 0.771 |
| 95% CI : (0.2005, 0.3596) | 95% CI : (0.6895, 0.8398) |
| No Information Rate : 0.6718 | No Information Rate : 0.6718 |
| P-Value [Acc > NIR] : 1 | P-Value [Acc > NIR] : 0.008557 |
|  |  |
| Kappa : -0.2618 | Kappa : 0.4807 |
| Mcnemar's Test P-Value : 2.899e-07 | Mcnemar's Test P-Value : 1.000000 |
|  |  |
| Sensitivity : 0.4884 | Sensitivity : 0.6512 |
| Specificity : 0.1705 | Specificity : 0.8295 |
| Pos Pred Value : 0.2234 | Pos Pred Value : 0.6512 |
| Neg Pred Value : 0.4054 | Neg Pred Value : 0.8295 |
| Prevalence : 0.3282 | Prevalence : 0.3282 |
| Detection Rate : 0.1603 | Detection Rate : 0.2137 |
| Detection Prevalence : 0.7176 | Detection Prevalence : 0.3282 |
| Balanced Accuracy : 0.3294 | Balanced Accuracy : 0.7404 |
|  |  |
| 'Positive' Class : SWEDD | 'Positive' Class : SWEDD |

***SMOTE based results***

| Confusion Matrix and Statistics: **GLM** early PD/SWEDD (SMOTE-based training data) | Confusion Matrix and Statistics: **GAM**, early PD/SWEDD  (SMOTE-based training data) |
| --- | --- |
| Reference | Reference |
| Prediction PD SWEDD | Prediction PD SWEDD |
| PD 111 7 | PD 112 6 |
| SWEDD 36 14 | SWEDD 35 15 |
|  |  |
| Accuracy : 0.744 | Accuracy : 0.756 |
| 95% CI : (0.6711, 0.8082) | 95% CI : (0.6838, 0.8188) |
| No Information Rate : 0.875 | No Information Rate : 0.875 |
| P-Value [Acc > NIR] : 1 | P-Value [Acc > NIR] : 1 |
|  |  |
| Kappa : 0.265 | Kappa : 0.2991 |
| Mcnemar's Test P-Value : 1.955e-05 | Mcnemar's Test P-Value : 1.226e-05 |
|  |  |
| Sensitivity : 0.66667 | Sensitivity : 0.71429 |
| Specificity : 0.75510 | Specificity : 0.76190 |
| Pos Pred Value : 0.28000 | Pos Pred Value : 0.30000 |
| Neg Pred Value : 0.94068 | Neg Pred Value : 0.94915 |
| Prevalence : 0.12500 | Prevalence : 0.12500 |
| Detection Rate : 0.08333 | Detection Rate : 0.08929 |
| Detection Prevalence : 0.29762 | Detection Prevalence : 0.29762 |
| Balanced Accuracy : 0.71088 | Balanced Accuracy : 0.73810 |
|  |  |
| 'Positive' Class : SWEDD | 'Positive' Class : SWEDD |

| Confusion Matrix and Statistics: decision tree, early PD/SWEDD (SMOTE-based training data) | Confusion Matrix and Statistics: random forest, early PD/SWEDD (SMOTE-based training data) |
| --- | --- |
| Reference | Reference |
| Prediction PD SWEDD | Prediction PD SWEDD |
| PD 120 7 | PD 106 4 |
| SWEDD 27 14 | SWEDD 41 17 |
|  |  |
| Accuracy : 0.7976 | Accuracy : 0.7321 |
| 95% CI : (0.7288, 0.8556) | 95% CI : (0.6585, 0.7974) |
| No Information Rate : 0.875 | No Information Rate : 0.875 |
| P-Value [Acc > NIR] : 0.99840 | P-Value [Acc > NIR] : 1 |
|  |  |
| Kappa : 0.343 | Kappa : 0.3023 |
| Mcnemar's Test P-Value : 0.00112 | Mcnemar's Test P-Value : 8.025e-08 |
|  |  |
| Sensitivity : 0.66667 | Sensitivity : 0.8095 |
| Specificity : 0.81633 | Specificity : 0.7211 |
| Pos Pred Value : 0.34146 | Pos Pred Value : 0.2931 |
| Neg Pred Value : 0.94488 | Neg Pred Value : 0.9636 |
| Prevalence : 0.12500 | Prevalence : 0.1250 |
| Detection Rate : 0.08333 | Detection Rate : 0.1012 |
| Detection Prevalence : 0.24405 | Detection Prevalence : 0.3452 |
| Balanced Accuracy : 0.74150 | Balanced Accuracy : 0.7653 |
|  |  |
| 'Positive' Class : SWEDD | 'Positive' Class : SWEDD |

| Confusion Matrix and Statistics: XGBoost |
| --- |
| Early PD/SWEDD (SMOTE-based training data) |
| Reference |
| Prediction PD SWEDD |
| PD 117 4 |
| SWEDD 30 17 |
|  |
| Accuracy : 0.7976 |
| 95% CI : (0.7288, 0.8556) |
| No Information Rate : 0.875 |
| P-Value [Acc > NIR] : 0.9984 |
|  |
| Kappa : 0.3956 |
| Mcnemar's Test P-Value : 1.807e-05 |
|  |
| Sensitivity : 0.8095 |
| Specificity : 0.7959 |
| Pos Pred Value : 0.3617 |
| Neg Pred Value : 0.9669 |
| Prevalence : 0.1250 |
| Detection Rate : 0.1012 |
| Detection Prevalence : 0.2798 |
| Balanced Accuracy : 0.8027 |
|  |
| 'Positive' Class : SWEDD |
