# Supplementary material for: Non-motor Clinical and Biomarker Predictors Enable High Cross-Validated Accuracy Detection of Early PD but Lesser Cross-Validated Accuracy Detection of Scans Without Evidence of Dopaminergic Deficit
Source: Front Neurol. 2020 May 11;11:364. doi: 10.3389/fneur.2020.00364 (PMC7232850; doi:10.3389/fneur.2020.00364)
Supplement: Data Sheet 1 — Supporting Information I-V. [file Data_Sheet_1.ZIP › Appendices/Supporting Information II.docx]

*Supporting Information II* (S2)

Figure legends

Table S2-1: Early PD/controls logistic general additive model (GAM). Diagnostics used were gam.check and qq.gam from the mgcv package (Wood et al,. 2018).

Table S2-2: Early PD/SWEDD logistic general additive model (GAM). Diagnostics used were gam.check and qq.gam from the mgcv package (Wood et al,. 2018).

FIG S2-1: Early PD and controls qq.gam plot

FIG S2-2: Early PD and controls scatterplot, logistic GAM

*Early PD/SWEDD: one data-set (internal evaluation using k-fold resampling)*

Table S2-2: Early PD and SWEDD logistic general additive model (GAM). Diagnostics used were gam.check and qq.gam from the mgcv package (Wood et al,. 2018).

FIG S2-3: Early PD and SWEDD qq.gam plot

FIG S2-4: Early PD SWEDD scatterplot. Logistic GAM

**TABLE S2-1**

Family: binomial

Link function: logit

Formula:

gam(ENROLL_CAT~ s(age, bs="tp", k=3) + s(RevUpsit.sum, bs="tp", k=6) +

s(rbdSum, bs="tp", k=4) + s(MCATOT, bs="tp", k=5) +

s(NP1CNST, bs= "tp", k=4) + s(pTau, bs= "tp",k=3),

data= LR1_rs1,

method = "REML",

family= binomial(link= 'logit'))

Parametric coefficients:

Estimate Std. Error z value Pr(>|z|)

(Intercept) 6.287 3.214 1.956 0.0504 .

Approximate significance of smooth terms:

edf Ref.df Chi.sq p-value

s(age) 1.000 1.000 4.339 0.0372 *

s(RevUpsit.sum) 2.160 2.669 53.537 1.42e-11 ***

s(rbdSum) 1.000 1.000 4.703 0.0301 *

s(MCATOT) 2.739 2.945 3.577 0.2875

s(NP1CNST) 1.763 2.097 5.394 0.0749 .

s(pTau) 1.000 1.000 3.825 0.0505 .

---

Signif. codes: 0 ‘***’ 0.001 ‘**’ 0.01 ‘*’ 0.05 ‘.’ 0.1 ‘ ’ 1

R-sq.(adj) = 0.585 Deviance explained = 55.9%

-REML = 90.787 Scale est. = 1 n = 298

**
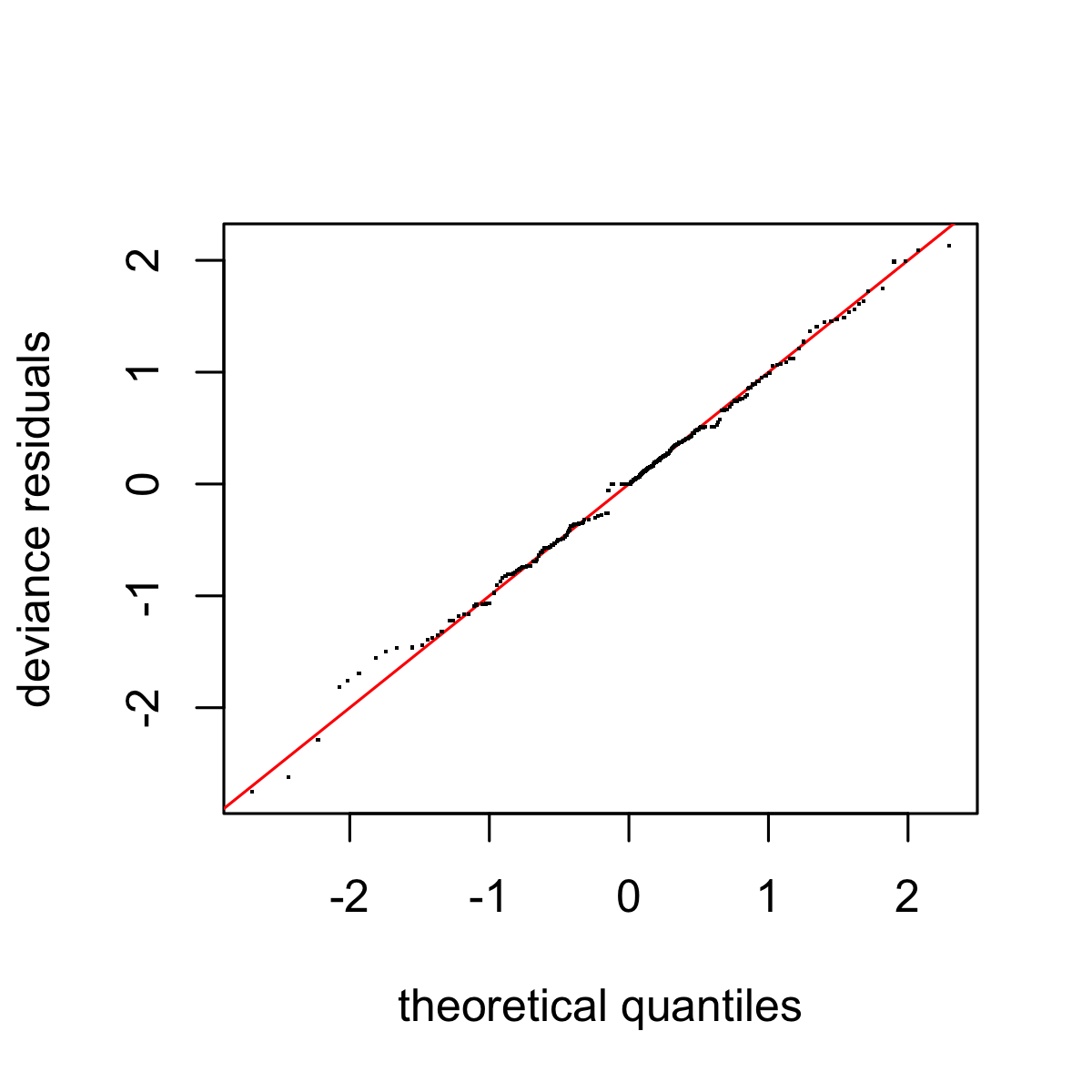
**

**FIG S2-1: Early PD and controls, qqplot (qq.gam)**

**
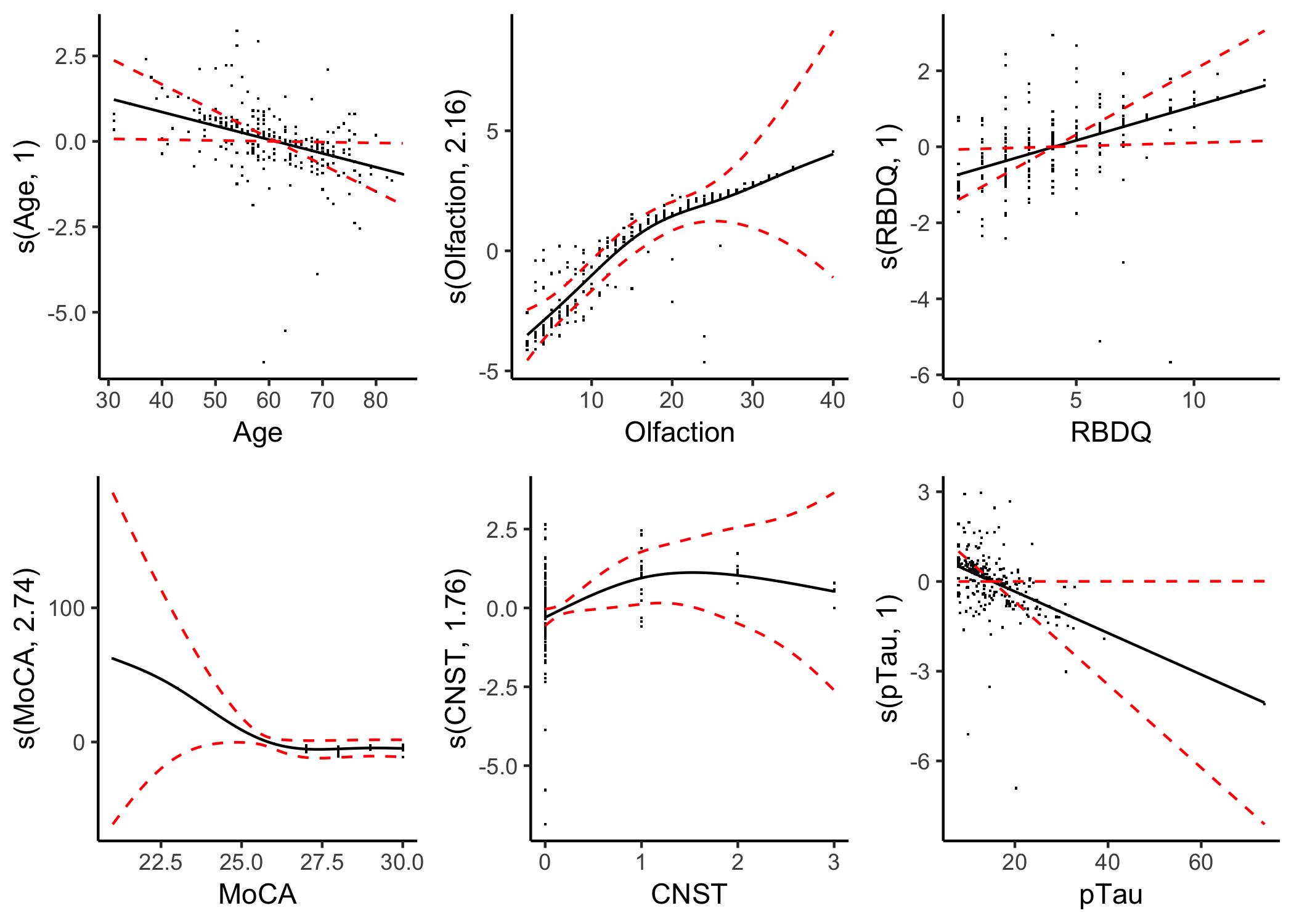
**

**FIG S2-2 Early PD/controls logistic GAM scatterplots:** The solid line is the predicted value of the dependent variable as a function of the x axis, where s(x) is fit as the outcome as a smooth of x. The dashed lines are plus-or-minus two standard errors. The y-axis is in the linear units, here logits, centered on 0 (50/50 odds), and included both positive and negative values (for graph function details see <https://cran.r-project.org/web/packages/mgcViz/vignettes/mgcviz.html>)

**TABLE S2-2:** Early PD and SWEDD, logistic general additive model (GAM)

Family: binomial

Link function: logit

Formula:

ENROLL_CAT ~ age + RevUpsit.sum + rbdSum + s(EDUCYRS, bs = "tp", k = 7) + gend + gdsSum

Parametric coefficients:

Estimate Std. Error z value Pr(>|z|)

(Intercept) -10.20406 3.43193 -2.973 0.002946 **

age 0.13050 0.04095 3.187 0.001438 **

RevUpsit.sum -0.40482 0.09974 -4.059 4.94e-05 ***

rbdSum 0.57431 0.16794 3.420 0.000627 ***

gend 2.63400 1.13550 2.320 0.020358 *

gdsSum 0.59875 0.31026 1.930 0.053623 .

---

Signif. codes: 0 ‘***’ 0.001 ‘**’ 0.01 ‘*’ 0.05 ‘.’ 0.1 ‘ ’ 1

Approximate significance of smooth terms:

edf Ref.df Chi.sq p-value

s(EDUCYRS) 4.119 4.912 14.39 0.0126 *

---

Signif. codes: 0 ‘***’ 0.001 ‘**’ 0.01 ‘*’ 0.05 ‘.’ 0.1 ‘ ’ 1

R-sq.(adj) = 0.635 Deviance explained = 62.3%

-REML = 33.054 Scale est. = 1 n = 88


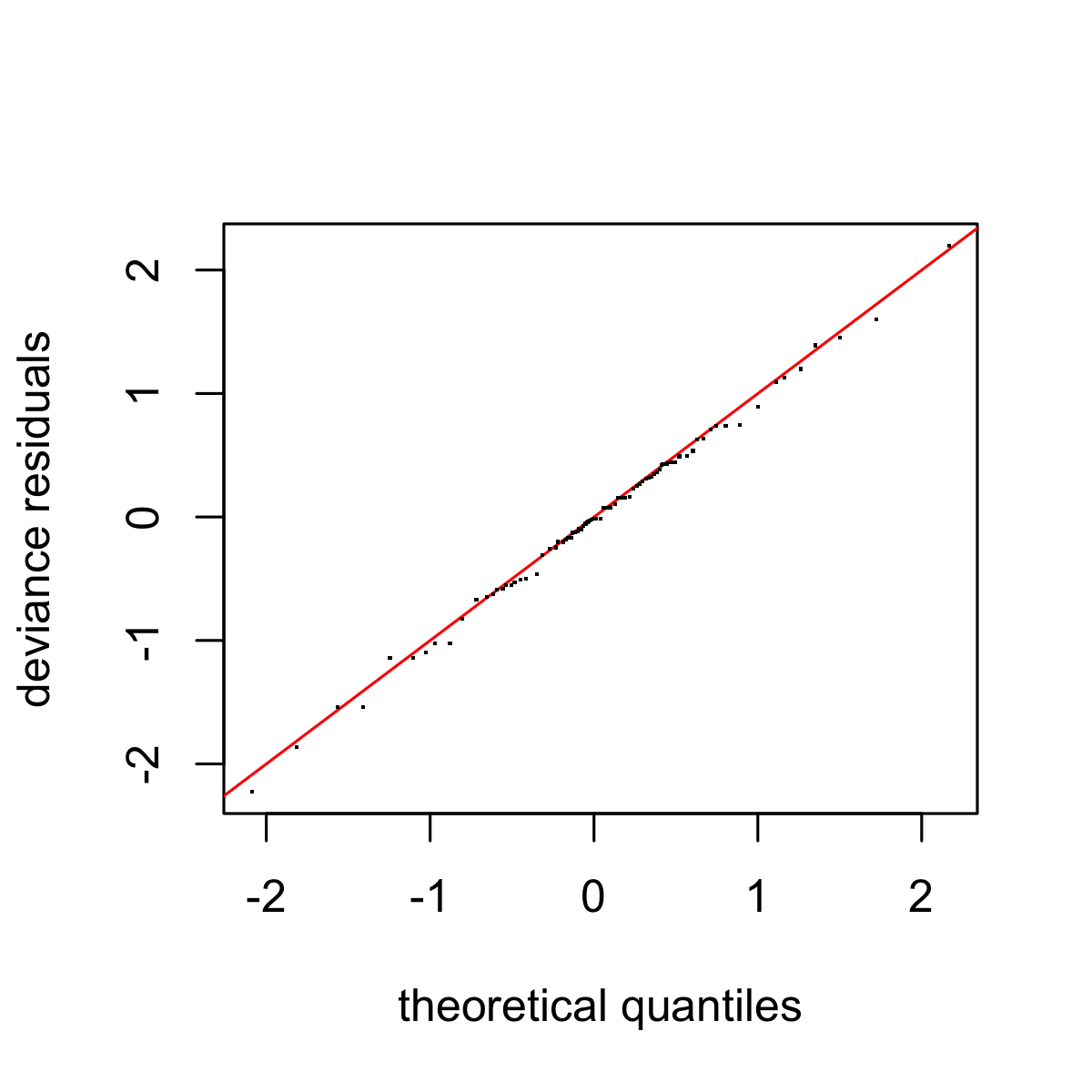


**FIG S2-3 Early PD and SWEDD, qqplot (qq.gam)**


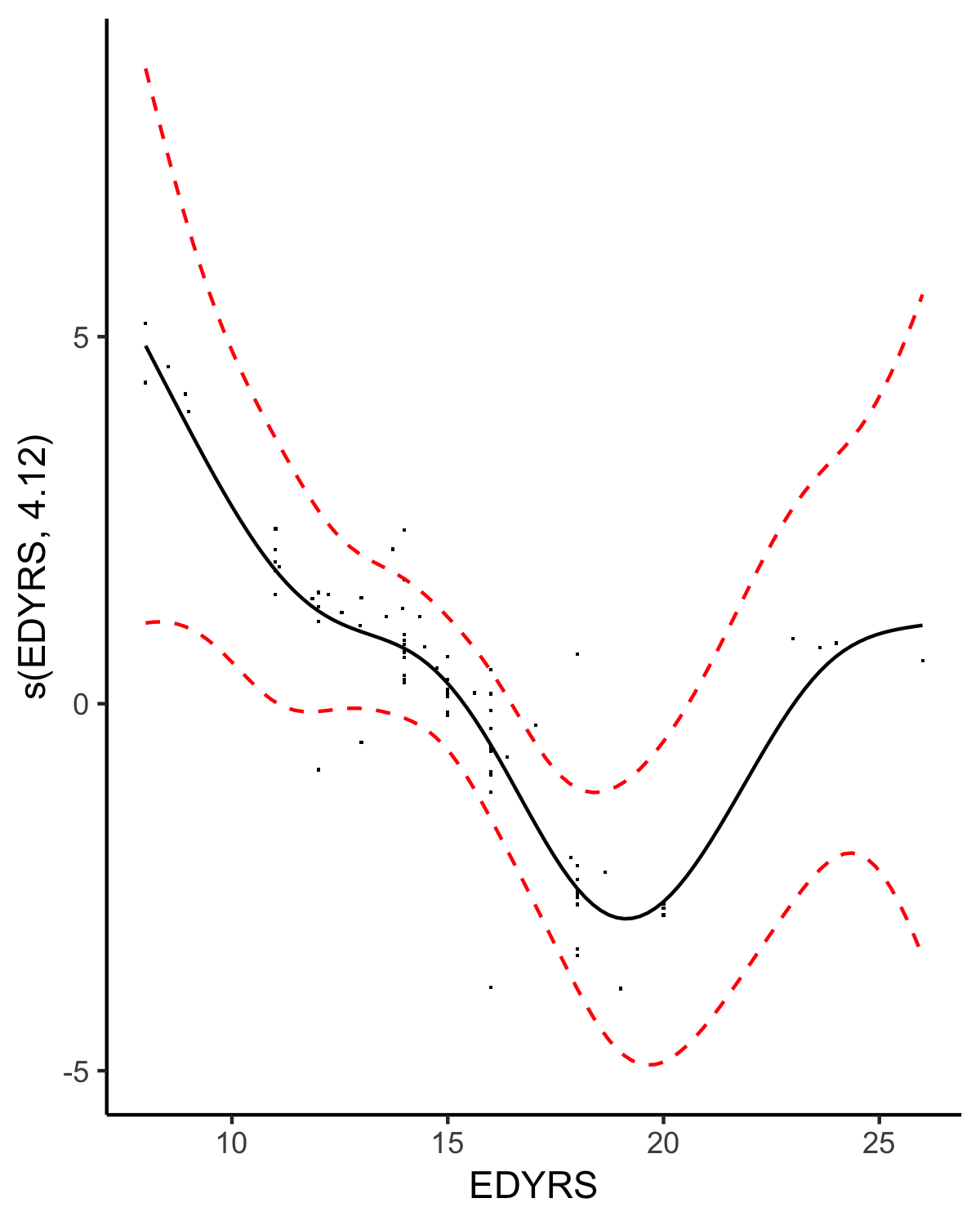


**FIG S2-4 Early PD/controls logistic GAM scatterplot (years of education**): EDYRS = years of education**.** The solid line is the predicted value of the dependent variable as a function of the x axis, where s(x) is fit as the outcome as a smooth of x. The dashed lines are plus-or-minus two standard errors. The y-axis is in the linear units, here logits, centered on 0 (50/50 odds), and included both positive and negative values (for graph function details see <https://cran.r-project.org/web/packages/mgcViz/vignettes/mgcviz.html>)
