# Supplementary material for: Non-motor Clinical and Biomarker Predictors Enable High Cross-Validated Accuracy Detection of Early PD but Lesser Cross-Validated Accuracy Detection of Scans Without Evidence of Dopaminergic Deficit
Source: Front Neurol. 2020 May 11;11:364. doi: 10.3389/fneur.2020.00364 (PMC7232850; doi:10.3389/fneur.2020.00364)
Supplement: Data Sheet 1 — Supporting Information I-V. [file Data_Sheet_1.ZIP › Appendices/Supporting information IV.docx]

*Supporting information IV*

*Predictive classification model evaluation metrics*

Error in context of predictive classification models is not expressed as MSE, RMSE or *R^2^*; such measures are appropriate for regression but not classification models ^(1)^. In classification problems, error is generally the proportion or fraction of misclassifications that can be expressed in the form

$$\frac{1}{n} \sum_{i=1}^{n} I(yi \neq\hat{y}i) ,$$

where $\hat{y}i$ refers to the label associated with the predicted class (e.g. Class 1 vs. Class 2) for the *i*th observation, and $I(yi \neq\hat{y}i)$ is an indictor equal to zero if $yi= \hat{y}i$ (correct classification) but equal to 1 if $yi \neq\hat{y}i$ . In the event $I\left( yi \neq\hat{y}i \right)= 0$the model correctly classified the *i*th observation, else the *i*th observation was misclassified ^(1, 2)^. Ultimately, minimized misclassification error is equivalent to maximized accuracy. More specific indices of classification error are typically used in analyses aimed at predicting pathology.

With a binary response variable (e.g. with or without disease) a predictive model outputs continuous probabilities of presence/positive (P) or absence/negative of an event, such as disease. The probabilities indicate the extent of predicted case membership in either the presence of disease (P) group or absence (1-P) of disease group. A threshold is set, typically 0.50 (a 50% cutoff to discriminate the predicted class); statistical software normally defaults to a 0.50 threshold. Below this threshold cases are predicted as members of the disease absence group; cases above the threshold are predicted as positive, and hence members of the group with disease. The continuous probabilities (or, alternatively, discrete predicted class labels) produced by a model’s binary classification allows calculation of four different fractions in a 2 x 2 confusion matrix$\begin{matrix} TN & FN \\ FP & TP \end{matrix}$: the true positive (TP) fraction is the proportion of cases correctly predicted as with disease; the true negative (TN) fraction is the proportion of cases correctly predicted without disease; the false positive fraction (FP: equivalent to Type I error or commission error) is the proportion of cases incorrectly predicted as diseased; and the false negative (FN: equivalent to Type II error or omission error) fraction is the proportion of cases with disease incorrectly predicted as healthy or without disease. If a model threshold other than 0.50 is selected it will alter confusion matrix values.

Binomial classification model performance is often assessed with the receiver operating characteristic (ROC) area under the curve (AUC). Other often-used performance measures are accuracy, Cohen’s Kappa, sensitivity, and specificity. Binary transformed probabilities from TP, TN, FP, FN fractions are used to derive performance measures including accuracy, Cohen’s Kappa, sensitivity, and specificity. Cohen’s Kappa ^(3)^, or simply the Kappa statistic, was originally used to compare agreement between experiment raters, but it also measures the performance of a model, its accuracy, relative to random chance. It can be expressed as $K= \frac{O-E}{1-E}$, where *O* is observed accuracy and *E* expected accuracy, the latter referring to random chance. In general a higher Kappa is better: 0.41-0.60 is rated as moderate and a Kappa >.75 as excellent ^(4)^, though there is currently not a universally accepted scale for interpretation of the Kappa statistic. Despite wide spread usage, the Kappa statistic has earned sharp criticism from more than a few sources. Reported problems include, but are not restricted to, redundancy (due to high correlation) with the general accuracy measure, a basis on random chance probabilities that are not random, and underestimation of the probability of correct classification ^(5-8)^.

Accuracy is often confused with the ROC AUC statistic (often referred to a simply the AUC), but the two measures differ. Accuracy, otherwise known as diagnostic accuracy, is simply the number of correct predictions a model or classifier makes expressed as the proportion of correct predictions over all predictions. To elaborate, accuracy considers the predicted true negative (TN), true positive (TP), false negative (FN) and false positive (FP) values that result from an algorithm. In the context of binary classification, accuracy (*A*) can be derived from the 2 x 2 confusion matrix illustrated above and can be expressed as $A= \frac{TP+TN}{\sum(TP, TN, FP, FN)}$. In short, diagnostic accuracy is the proportion of correct predictions made by the classifier, and it imposes a cutoff threshold of > 0.50 ^(9)^. It warrants note, that accuracy is not reliable when classes are severely imbalanced. For example 96 instances of pathology and 4 instances of healthy controls has a minority class rate of 4% (4/4+96). In such a circumstance, accuracy would be 96%, and controls would have a nil recognition rate, meaning accuracy is just reflecting the underlying class imbalance. Moreover, accuracy, as well as Cohen’s Kappa, sensitivity, and specificity depend on the class discrimination threshold.

The AUC statistic, by contrast, is not a function of a given threshold setting but evaluates a classifier’s accuracy to discriminate between two classes across all possible threshold cut-offs. Further, a model that achieves perfect class separation would fall at point (0,1) on a ROC curve; the format of a ROC curve is provided in Graphic 1. In a model with poor accuracy the ROC curve may fall on the diagonal, which reflects only a 50% chance of correct classification. The AUC is a performance summary measure that is based on evaluation of continuous class probabilities, were .50 is equivalent to chance. ROC curves falling in closer proximity to the upper left hand corner reflect more accurate models: AUC values of .60 - .70 reflect relatively poor models while AUC values of .90 and up indicate high model classification accuracy. A measure of .90 indicates there is a 90% probability that a randomly chosen individual with presence of disease has a higher AUC value than a randomly chosen individual without disease ^(10)^. In general, the AUC is the probability or chance that a randomly selected subject with disease is rated more likely to have disease than a randomly selected subject without disease ^(11)^.

The AUC combines both sensitivity and specificity, where each paired instance of sensitivity and specificity corresponds to a ROC space single point ^(12)^. The paired points of sensitivity, plotted on the y-axis and specificity, plotted on the x-axis, occur across all cutoff thresholds. Sensitivity (also known as recall) is the true positive rate (TPR), also referred to as the true positive fraction (TPF): the percentage of events/cases with pathology correctly identified as positive for the presence of pathology. Specificity is the false positive rate (FPR), also referred to as the false positive fraction (FPF); the percentage of non-events/non-pathology cases correctly identified as negative and without pathology. In a ROC curve, there is a trade-off between the sensitivity and specificity. If sensitivity is high (perhaps set by a predetermined threshold) specificity will lower; if specificity is high sensitivity will be lower. Each point in ROC space then, is a function of a given threshold or cutoff. Importantly, the ROC AUC statistic is invariant to threshold change (and class prevalence); different thresholds can change sensitivity and specificity but will not alter the AUC measure of model performance. Specifically, a different classification threshold (e.g. .40 rather than .50) alters confusion matrix$\begin{matrix} TN & FN \\ FP & TP \end{matrix}$ values but not the AUC.

As distinct metrics, sensitivity and specificity offer insight to model classification error. Again, specificity is the model’s percentage of correctly identified negatives (non-events; typically healthy controls). A model with high specificity has a lower type I error (or false positive rate). Specificity (*SPE*) can be expressed as $SPE= \frac{TN}{TN+FP}=1-FPR$, where *FP* is the number of false positives and *TN* is the number of true negatives. *SPE* is equivalent to 1- false positive rate (FPR). Sensitivity, as just defined, is the true positive rate (TPR), the model’s percentage of correctly identified positives (events, or those with disease). Sensitivity can be expressed as $SN= \frac{TP}{TP+FN}=1-FNR$, where *SN* is sensitivity, *TP* the number of true positives, and *FN* is the number of false negatives. Given the true positive, false negative, false positive and false negative measures from which sensitivity and specificity are derived, it is not surprisingly that error, in the context of a confusion matrix, can be couched in terms of sensitivity and specificity ^(1)^.

Critical to the sensitivity and specificity indices is selection of the appropriate cut-off. Often the .50 classification cut-off has insufficient sensitivity to detect an event or case of pathology. Or, antithetically, specificity may be poor resulting in a low correct identification percentage of those without pathology. Of course, to eliminate unnecessary diagnostic procedures, a diagnostic test that is both highly specific (to rule out disease) and sensitive (to identify disease) is ideal. While achieving this may often not be possible, estimated threshold cutoffs based on maximal sensitivity and specificity are in aid of such predictive disease screening. One approach is to calculate a balanced threshold; a threshold that maximizes both sensitivity and specificity across all classification cut-off points to arrive at an optimal cut-off threshold. This is known as the Youden’s index ^(13)^. The Youden Index Youden’s *J* index can be expressed as: *J* = *SN+ SPE* – 1. Various software offerings provide a Youden Index option for ROC AUC analysis. Another method of finding an appropriate cut-off value is to use the base rate of a given disease or event’s prevalence in the population ^(14)^. Should there be a need to prioritize identifying disease, a cut-off with relatively high sensitivity, say > .80 to lower the false negative rate, would be effective for screening disease as true positive cases are less likely to be missed. However, prioritizing sensitivity comes at the expense of specificity ^(14)^. Of note, deriving a cutoff threshold from a training or validation data-set post hoc will can result in an overly optimistic model performance estimate ^(15)^. Ideally, the cutoff threshold should be estimated from an evaluation data-set not used in training or testing the model ^(1)^, though often alterative data sources for this approach are not available.

In a clinical context a test that detects 85% of patients with disease while not detecting the remaining 15% with disease: this test has 85% sensitivity (true positive rate) to correctly detect disease but a false negative rate of 15%; the presence of disease was not detected in 15% of those with disease. If a test has 85% specificity, it correctly identifies 85% of patients without disease (true negatives) but 15% of patients are incorrectly classified as with disease (false positive). A suggested method to more effectively rule out disease (correct true negative classification), is for patients with a high sensitivity outcome but low specificity to undergo a second test but one with antithetically low sensitivity but high specificity. This would facilitate identification of those false positive and without disease ^(16)^.

Imbalance between classes adds additional complexity to cut-off selection and methods for resolving class imbalance should be considered prior to selection of a cut-off. Moreover, any metric (e.g. accuracy) based on values in both columns of a confusion matrix $\left( \begin{matrix} TN & FN \\ FP & TP \end{matrix} \right)$ will be sensitive to imbalance or class proportion disparity. Antithetically, ROC curves are based on the TP and FP rates, which are ratios independent of class prevalence ^(12)^. It is important to underline that accuracy – a much used performance measure- is dependent not only on the classification threshold but on the frequency of each class. By contrast the ROC AUC statistic is threshold-independent and unaffected by imbalanced data ^(12, 17)^. Consequently, and especially in the context of imbalanced data, the AUC statistic is regarded as the preferred indicator of performance ^(1)^ and an index of model *global* accuracy.

Accuracy, as already noted, is sensitive to differential class distribution where the number of positive/negative instances in a class differs, particularly for large case instance discrepancy between classes. Sensitivity and specificity (integral to the ROC AUC), are not sensitive to class imbalance. For example, consider a circumstance with 200 positive observations (case instances) and 200 negative observations (non-case or controls). Accuracy is given the general accuracy expression $A= \frac{TP+TN}{\sum(TP, TN, FP, FN)}$, and TP = 140, TN = 160, FP = 40 and FN = 60; accuracy or A =.75 (75%.) Given sensitivity (SN) is defined as $SN= \frac{TP}{TP+FN}=1-FNR$ and specificity (SPE) is defined as $SPE= \frac{TN}{TN+FP}=1-FPR$, in this example SN = .70 and SPE = .80. If the negative samples increased by a factor of 10 to 2000 and resulted in new TN and FP rates of 1600 and 400 respectively, accuracy is altered to .79, but sensitivity and specificity remain unchanged. Clearly, this class imbalance altered the accuracy metric but not sensitivity and specificity. However, if the threshold at which class labels are predicted is altered then accuracy as well as both sensitivity and specificity will be impacted ^(18, 19)^.

Consider a ROC curve used to determine the predictive classification accuracy of a trained model on validation or test set data. While the ROC AUC is threshold-invariant, changes in threshold can be readily demonstrated to, in turn, change sensitivity (the number of positive cases correctly identified) and specificity (the number of negative cases correctly identified). Typically, a decrease in threshold increases sensitivity and returns more positives, while an increase in threshold heightens specificity and returns more negatives. In short, specificity and sensitivity are inversely proportional. This is illustrated ROC AUC Graphic 1 below.


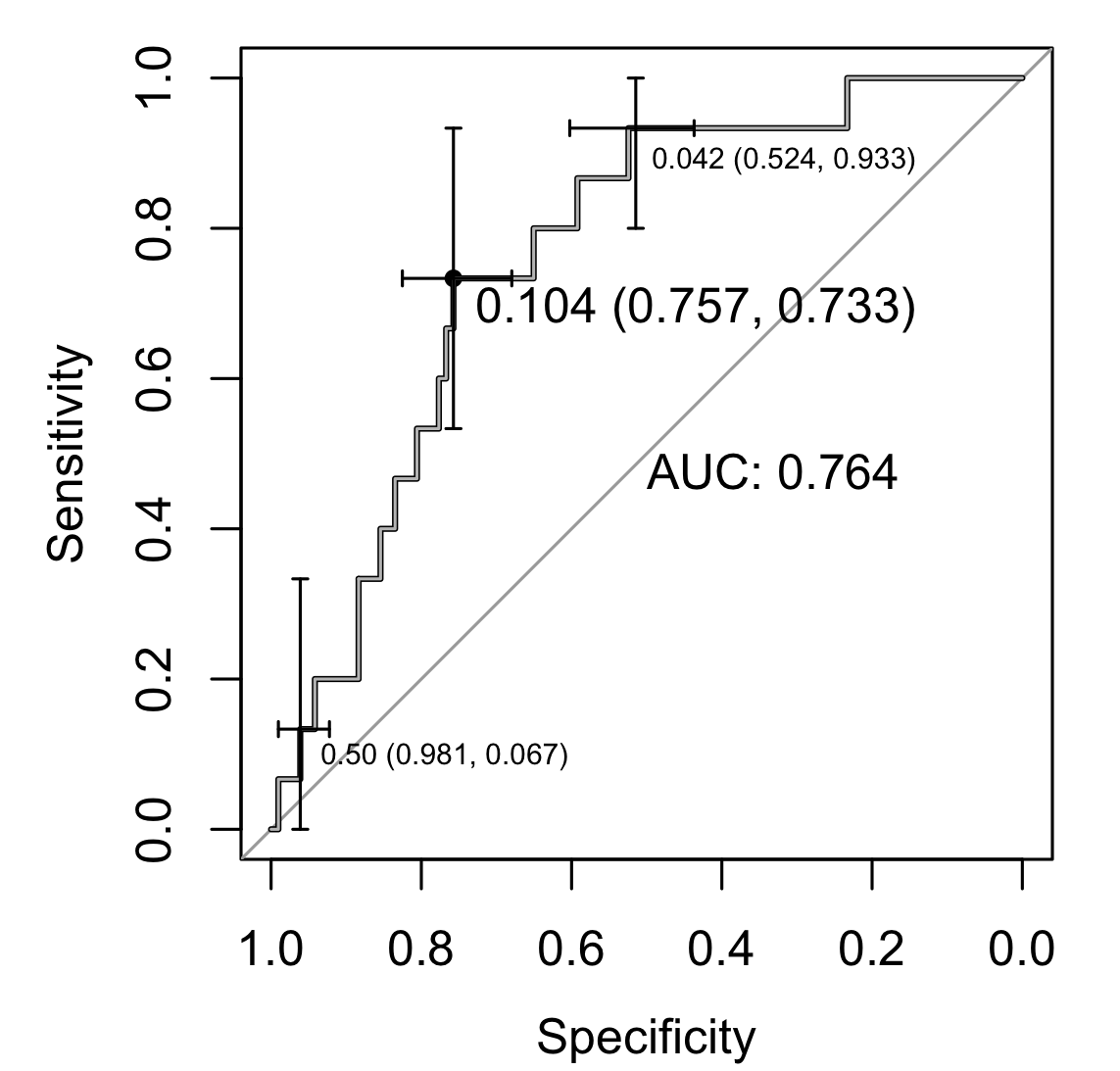


**GRAPHIC 1: The ROC curve**

The point (0,0), in the lower left corner, corresponds to a threshold at which all cases are classified as negative; point (1,1), in the upper left corner, corresponds to the threshold at which at all cases are positive. At the default 0.50 threshold, (error bars) near the lower left corner, sensitivity is only about 7% but specificity is 98%. By contrast, at the threshold of less than 5% (.042), the upper-most set of numbers, sensitivity is 93% but specificity is reduced to 52%. The best balance of sensitivity and specificity with this data occurs at the threshold of .104, which is a Youden Index ^(13)^ optimized point that minimizes the mean positive and negative error rates while maximizing the sum of specificity and sensitivity. At a threshold of .104, sensitivity is 73% and specificity is 76%. The error bars in the above graph reflect variation in specificity and sensitivity.

*Cross-validation*

The litmus test of a trustworthy model’s accuracy is performance on previously unused data, data on which the existing model was not trained – a cross-validation paradigm. Comparable results on training and evaluation data-sets indicate the model will generalize well ^(20)^. Ideally, model accuracy should be tested on a new data-set (from the same population), though availability of such entirely new samples is quite infrequent. Alternatively, a single data-set can be partitioned (prior to pre-processing and feature selection), split into separate train and test/validation set portions. In this quite common circumstance, the model is trained on a train partition subset, typically 60-80% of the data, and then the model is tested on the remaining test/validation partition “unseen” by the model.

One method of splitting the data into training and test or validation subsets is simple random sampling. Another approach is stratified random sampling, which in the context of a classification analysis preserves proportions within classes by sampling within classes (e.g. as achieved by the “createDataPartition” function in the caret package function) ^(21)^. The training error rate (see *Evaluation metrics* above) and performance metrics (e.g. AUC, specificity, and sensitivity) are computed on the training data but of greatest concern is the performance outcome when the model is applied in cross-validation fashion to the unseen partition or new data. External and internal types of cross-validation can be distinguished. External cross-validation is the validation process described so far. Internal validation refers to cross-validation within the model itself using only a portion of the data allocated to train the model. The rpart decision tree and and XGBoost model types are examples of algorithms with built-in, internal cross-validation whereby multiple models are internally tested on (hold-out) data not used to train a given model. Further, a model is often then externally cross-validated; tested on an unseen data partition or new data set. Internal cross-validation improves the models ability to generalize through a resampling method.

*Resampling and internal validation*

In general, resampling provides internal cross-validation by fitting the model on a subset of the data allocated to train the model. A portion of the training data is held-out for model performance testing. Such data resampling is an iterative process but how data subsets are selected differentiates resampling methods. K-fold and the bootstrap are commonly adopted resampling methods for cross-validation. In the k-fold resampling method data is partitioned into approximately equal sized k-subsamples; one of the subsamples (the first fold) is held out for validation testing while the other k-1 subsamples are used to train or fit the model. It warrants emphasizing that class predictions are made on the validation or held-out data not on the data used to train a model. For example, if *k*=10, *k*-1 or 9 samples are used to train a model, 1 sample is held-out for validation on which predictions are computed; 90% of the data is therefore allocated to training and 10% is allocated to validation. Again, this is an iterative process, with many models being built allowing optimal hyper-parameters/tuning parameters to be selected. With each k-repetition a different segment of the observations is treated as the validation test data-set (then returned to the training data-set), which results in k-test error estimates of model predicative performance. A k-fold cross-validated (CV) estimate is the average of the k-test error estimates ^(2)^.

The bootstrap differs. The model is built on random samples taken from the original data-set with replacement ^(22)^, meaning that the same cases can be repeatedly used to build the model which can result in bias; greater that 63% of the same cases has been reported ^(1)^. In addition some observations may not be allocated to the validation test data-set at all. By contrast, in the k-fold approach every observation will be used for both training and validation and each observation is allocated for validation not more than once. Moreover, though it is a point of debate ^(23)^, it has been contended that the k-fold technique (10-20 folds) reduces variance between (training) models relative to a method such as leave one out cross-validation ^(24)^. Leave-one-out CV is a particular application of k-fold CV where *k* is replaced by *n* (*n*= number of samples) and the model is fit on *n*-1 training data observations. With sufficient k-fold repetitions (e.g. k=10), similar variance and hence results have been reported for both k-fold and leave-one out methods ^(25)^. While a single CV method will not be ideal for all data-sets, for smaller sample sizes acceptable bias and variance outcomes can be achieved using the 10-fold CV approach ^(1)^.

*Sampling methods*

Sampling (also referred to as subsampling) techniques include down-sampling, up-sampling as well as hybrid approaches. In a very cursory description of subsampling methods, down-sampling data randomly samples classes and matches class proportions to the class with the lowest frequency of observations, though this could lead to loss of important majority class information. Up-sampling, in a largely antithetical procedure, randomly samples the minority or rare event class with replacement such that it is the same frequency as the class with the greater frequency of observations. A drawback is potential overfitting from replication of trends in the original dataset observations, meaning predictive performance on a separate validation dataset may be poor because the (trained) model adheres too closely to patterns in the training data (see bias-variance trade-off in *Factors impacting predictive modelling*) to adapt to new patterns in validation data. Random over sampling examples (ROSE) ^(26)^, and synthetic minority over-sampling (SMOTE) ^(27)^ are hybrid techniques that simulate new minority class data points while also down-sampling the majority class. Both are over-sampling methods that create artificial samples but they have differing sampling methods. ROSE generates artificial samples from the predictor (feature) space of the minority class using a bootstrapping approach. SMOTE, in a k-nearest neighbours approach, synthesizes new minority instance samples somewhere along a line between an existing randomly selected minority instance and that instance’s nearest (existing) minority instance neighbour. A number of studies have reported such subsampling methods allay data imbalance related issues ^(28-31)^, though mixed results have been reported elsewhere ^(1)^. Combined use of the both the ROC AUC performance metric and subsampling have been recommended to counter issues stemming from imbalanced data^(32)^.

*Collinearity and feature elimination*

Models are impacted by the correlation structure among predictors and with the response variable. Models built using predictors with high collinearity (e.g. > *r* .75 between predictors) can obscure interpretation of results. Two highly correlated predictors may each have similar important relationships with the response variable. In regression (linear or logistic) the coefficients of highly correlated predictors have sizable overlap in contribution to the response variable; these coefficients are therefore not unique and difficult to interpret. Moreover, in models based on algorithms without collinearity issues (e.g. tree-based models), the importance of one predictor can be masked by another predictor with which it is highly correlated, which can lead to an inaccurate conclusion that one of these predictors is unimportant ^(1, 9, 33)^.

Under the rubric of feature (predictor) elimination however, high correlation among predictors is not a concern; the objective is not interpretation of the data but simply to reduce irrelevant or redundant predictors. Feature elimination can remove predictors that similarly impact the response variable. Eliminating such redundant parameters mitigates overfitting ^(34)^. Feature elimination is often undertaken to arrive at the most potent group of predictors. For logistic regression, a stepwise approach automates sequential addition of a candidate predictor to a model, then evaluates it for elimination by refitting the model ^(35, 36)^. In this commonly used method, the Akaike information criterion (AIC) is increasingly used as the criterion for predictor elimination. AIC is a measure of model fit that imposes a penalty for each added predictor; a lower AIC value is better. A preferred variant of stepwise regression is backwards stepwise regression ^(9)^, where all predictors are initially incorporated in the model, then iteratively removed or retained based on the change in model AIC. This is a recursive feature selection technique. A stepwise approach is useful in narrowing a set of variables predetermined by research as relevant, but without prior screening to include only subject-relevant predictors at the outset, it is possible that even noise can be selected by this method as a significant predictor ^(37, 38)^.

Many statistical algorithms have built-in mechanisms to reveal predictors of greatest import to a model. Regression models automatically indicate the importance of predictors: for example the logistic regression predictor coefficient z-scores and odds ratios convey predictor contribution to a model. Decision tree models ^(39)^ have a summary measure, Goodness of Split, indicating contribution of each predictor to splits in a tree resulting in classification. In random forest ^(40)^ a measure called Mean Decrease in Gini also measures the importance of a predictor, but as random forest is an ensemble of trees, this measure is across all random forest trees. In XGBoost ^(41)^ a measure called Gain is typically used to determine feature importance, and it too calculates the contribution of each predictor across all trees in the model. For more information see the section *The models: logisitic regression, decision tree, random forest and XGBoost.*

Alternatively, generic or unifying predictor of importance estimators are available that can be applied to several model types. One such offering is the varImp function ^(21)^ for ranking the importance of predictors used in a model. If a modelling algorithm has a built-in calculation of predictor importance the varImp function calls the built-in importance function (but see ^(42)^. The varImp function can scale predictor contribution to a model between 0 and 100, with the predictor of lowest import given a value of zero. How the varImp function works depends on the model type. The documentation, including online sources ^(43, 44)^ stipulates scaling may be able to incorporate model-based inter-predictor correlations ^(1)^.

A review of classification-related literature determined 64% of studies adopted inappropriate feature selection validation, which resulted in overfitted models that had pessimistic results on test data ^(45)^. This emphasizes the importance of removing non-informative variables. Validating the feature set has been recommended as a solution, whereby the training data-set is used for predictor selection and feature selection is conducted within and encapsulated by cross-validation (e.g. 10-fold cross-validation) resampling of hyper-parameters ^(1)^. This allows feature selection to be applied to the held-out samples, which approximates testing on an independent data-set.

*Modeling and the caret package*

Model building typically involves a repetitive process. Relevant variables are used often in several trials in pursuit of the optimal inferential or predictive variable combination. The classification and regression training (caret) package ^(21)^ expedites the iterative model development process. Resampling (bootstrapping, leave-one-out cross-validation or k-fold cross-validation) methods are used to arrive at estimates of model performance. An objective of caret resampling is to find optimal model tuning parameters automatically while greatly facilitating comparisons of multiple model types. The resampling “injects” variation into the modeling process to aid in generalization on future samples and automates selection of the most representative model tuning parameters. The caret package automates model tuning currently for 237 model types (e.g. CART, fuzzy rules, glmnet, knn, random forest, XGBoost, etc.) and also simplifies random stratified data partition.

Tuning parameters of model types relevant to the current work are the decision tree complexity parameter (determines the “price” of misclassification and tree depth) the random forest mtry (the number of randomly sampled variables used to spit data) and in XGBoost there are multiple tuning parameters (for details see *The models: logistic regression, decision tree, random forest, and XGBoost)*. Caret resampling can expedite the process of finding optimal hyper-parameter settings, and offers the latitude to use a measure other than just misclassification error to select the optimal tuning parameter settings. Specifically, Kappa and general accuracy, or the ROC AUC can be adopted as criteria to find optimal tuning parameter settings. For imbalanced data, employing the ROC AUC in this regard has the advantage of bias-free analysis. Borrowing from the online caret reference (https://topepo.github.io/caret/model-training-and-tuning.html), the procedure can be conveyed in a simple algorithm, here incorporating 10-fold resampling and the ROC AUC performance measure:

| ***for*** a given tuning parameter do | | |
| --- | --- | --- |
|  | ***for*** each resampling iteration do | |
|  |  | Hold-out specific samples (10% of the samples) |
|  |  | Optionally pre-process the data |
|  |  | Fit the model on the remainder (90% of the samples) |
|  |  | Make predictions on the hold-out samples using ROC AUC |
|  | **end** | |
|  | Calculate average performance across hold-out predictions | |
|  |  |  |
| **end** | | |
| Determine the optimal tuning parameter(s) | | |
| Fit the final model to all of the training data using the optimal tuning parameter(s) | | |

The caret train module schematic above runs 10 times, using a range of tuning parameter settings, and with each iteration the module trains a model on 90% of the data but tests a given (tuning) parameter on 10% of hold-out data to which the training module was not exposed. Considering a tree-based model, trees are built using 9 samples and the first fold is held-out. The testing or evaluation criterion (the ROC AUC here) results across predictions on hold-out models are averaged, the optimal tuning parameter (the one with the highest ROC AUC) is found, and the optimal parameter(s) is then used to fit a final model to all of the training data. The final model, benefiting from variation lent by the resampling process, will have improved generalization relative to a model without such resampling. The measures (ROC AUC, and associated sensitivity and specificity or metrics Kappa and accuracy) used to evaluate tuning parameters also provide an estimate of model future performance. It is noteworthy, that by repeating 10-fold cross-validation to some extent (e.g. 10 fold repeated 3 times or 10 fold repeated 5 times) variance across held-out data samples is further reduced ^(1)^.

Caret can work in conjunction with built-in resampling methods. Using random forest out-of-bag (OOB) sampling as an example, the OOB sampling (a bootstrap with replacement) rate is calculated while the model is being built. By contrast, caret cross-validation, such as k-fold, makes predictions after the random forest model OOB rate has been computed, and the caret predictions are made on hold-out samples (without replacement).

It is important to note, that, the general linear model (glm) does not have tuning parameters, and caret will not tune logistic regression coefficients. Consequently, a logistic regression model’s coefficients are unaltered by caret resampling. Moreover, it warrants adding that the caret package also includes functions for random stratified data splitting, feature selection as well as pre-processing. Pre-processing, not discussed in the introduction, often takes the form of centering and scaling. Centering subtracts an average predictor value from that predictor’s full set of values (e.g. x – mean(x)). Scaling simply refers to conversion of predictor values to z-scores. Centering and scaling can therefore remove the influence of original scale values that may detract from a model’s ability to find relationships in the data. When centering is applied to all predictors, all will have a common mean of zero, though predictors do not necessarily need to be centered on their mean. It is important to note, that if zero constitutes a meaningful value in predictors, centering is not generally recommended ^(46)^.

*Factors impacting predictive modelling*

Many researchers favour a particular model type, yet it has been convincingly argued in the so-called “no free lunch” theorem that in the absence of substantive insight regarding a particular analysis issue, modeling algorithm (A) will not necessarily perform better than modelling algorithm (B) ^(47, 48)^, and consequently assessing several different model types can be informative.

The utility of logistic regression for classification has been previously verified ^(49)^. Its high usage over the past century to the present day underlines the overall across-discipline high regard for this algorithm. Decision tree ^(39)^models offer instant model visualization and are not limited by assumptions that can restrict the use of logistic regression. Random forest ^(40)^ uses decision tree ensembles but adds overfitting mitigation. XGBoost has properties that both control overfitting and advance the search for predictive relationships in a model ^(41)^.

The development of logistic regression and XGBoost occurred at opposite ends of a historical timeline. Logistic regression appeared on the scene in the mid 1800s ^(48)^; a stable release of XGBoost ^(41)^ appeared on the scene in 2017. Distanced by time, the tree-based algorithms, which include XGBoost, also differ widely from logistic regression in the bias-variance trade-off and sensitivity to variable variance. Data set size also differentially impacts the model types. The tree-based models are also distinguished from logistic regression by two additional related properties: tree models are non-parametric and have hyper-parameters while logistic regression is parametric and has parameters but not hyper/tuning parameters. As a prelude to a largely non-mathematical differentiation of logistic regression, decision tree, random forest, and XGBoost, the effect of data set size and variance on performance of these model types will be initially reviewed. Subsequently, the bias-variance trade-off will be outlined followed by differentiation of parameters vs. hyper-parameters.

Logistic regression has outperformed tree-based analysis across 32 smaller data-sets (*N* < 1000 observations) ^(49)^. Logistic regression and decision tree ^(39)^ have achieved a stable ROC AUC utilizing far fewer instances per variable relative to Random forest ^(50)^. By contrast, and in larger data-sets, random forest, has demonstrated higher performance ^(51)^. Similar outcomes were found for smaller versus larger data-sets, particularly data with higher levels of variance or noise: logistic regression performs best in smaller data-sets and data-sets distinguished by higher variability while random forest has superior performance in larger data-sets characterized by relatively high signal-to-noise ratio or low variability ^(52, 53)^. Relative to logistic regression, Random forest was found to attain a higher true positive rate (or sensitivity) but also a false positive rate that increased as variability in data increased ^(53)^. Narrowing discussion to data set size and performance, XGBoost (ROC AUC .860) has out performed logistic regression (ROC AUC .728) in a sample of *N* = 6682 ^(54)^, but in a sample of *N* = 551 logistic regression (ROC AUC .873) out performed random forest (ROC AUC .854) and XGBoost (ROC AUC .868) ^(55)^. Moreover, while logistic regression and XGBoost have shown comparable performance ^(56, 57)^ and logistic regression and random forest have modestly out performed XGBoost ^(58)^, a search of available online research (e.g. PubMed) ranks XGBoost quite consistently as the highest performing model, especially with larger data sets ^(59-62)^. Further, without doubt, the XGBoost algorithm ^(41)^ has become the performance front-runner in machine learning challenges ^(63)^. Yet, as articulated in a vetted on-line forum ^(64)^, it is important to stress that despite the excellent track record of XGBoost, it is not guaranteed to always be the best model type in all settings.

In a cursory outline of the bias-variance trade-off, bias refers to an over simplified analysis of a complex problem. A linear regression model assumes a linear Y and X_1_, X_2_, … X_n_ relationship (see Graph 1 A), but the relationship may not be linear and important patterns in the data may be unaccounted for by the model. In general, parametric models, including linear and logistic regression as well as discriminant model types, have higher bias relative to more flexible models including tree-based algorithms; the latter tend to have higher variance. Variance refers to the extent to which a model estimate $\hat{f}$will change when the data used to train the model is altered. A model with high variance closely adheres to the pattern of the observations and is not constrained by any assumption of linear data relationships (as in Graphic 1 B).


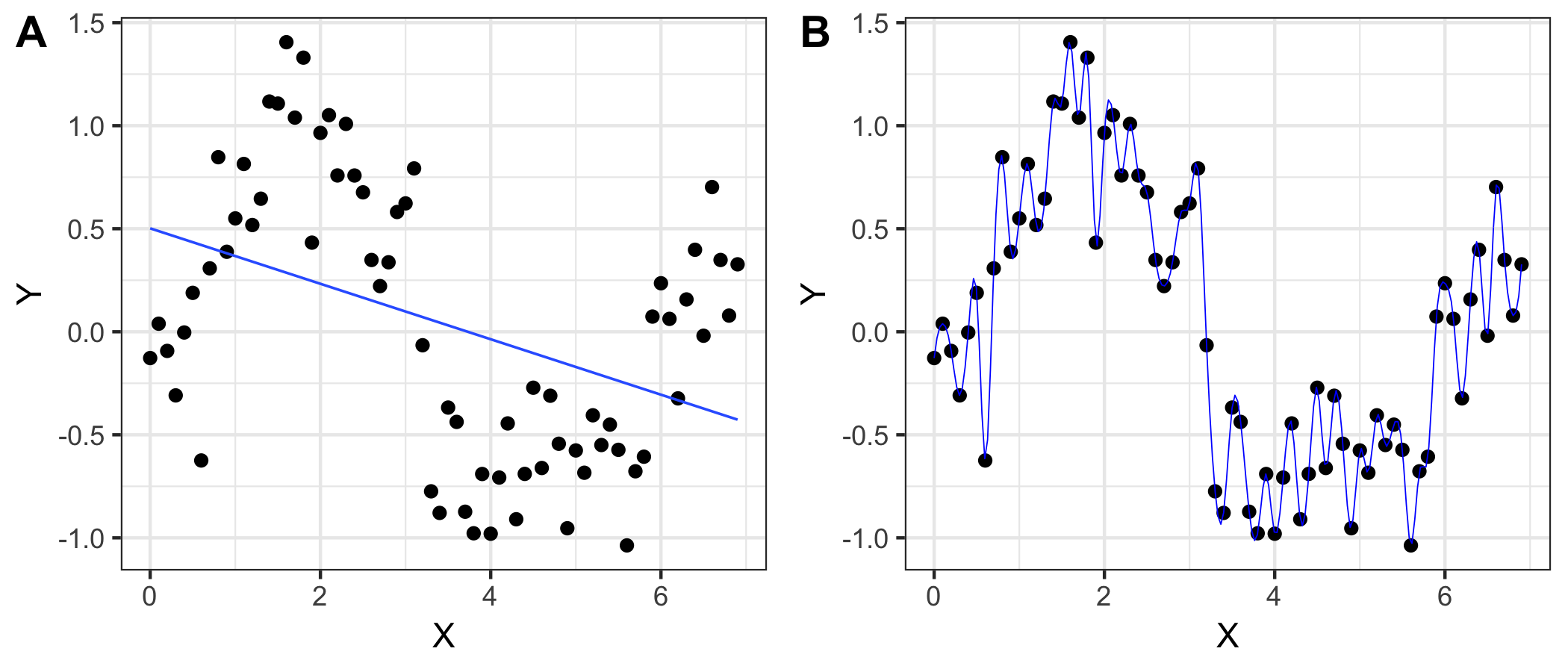


**GRAPHIC 2: Bias (A) vs. variance (B)**

Accordingly, a high bias but low variance model will fit a straight line to the data as in Graphic 2 A, while a high variance but low bias model may fit a curve passing through all points, as in Graphic 2 B. But the high variance model can be overly sensitive; so sensitive that it can even learn noise in the training data, which will likely result it having poor accuracy when applied to another data sample from the same population, given the new sample with not have identical noise to the training data on which the model was built. Ideally, the aim of a model is to have both low bias and low variance, and models pursue a bias-variance trade-off that both efficiently learns the patterns in a training data sample while also generalizing well. The bias-variance trade-off is often referred in terms of an underfitted-overfitted trade-off, and again the challenge is to find the balance between over and under fitting that captures key data patterns in the training data while also generalizing with high accuracy ^(2)^.

Parameters are derived or learned from the data and most often are not set manually. Parameters define a quantifiable contribution to the model. For example, logistic regression coefficients are parameters (as are support vector machine support vectors and neural network weights). Hyper-parameters, also referred to as tuning parameters, are not derived from the data by means of some analytical formula. Typically they can be entered manually and can be improved or tuned by changing their values manually or programmatically. For a given analysis, the optimal tuning parameter value is unknown ^(1)^. The decision tree complexity-parameter (a hyper-parameter) the Random forest mtry hyper-parameter and the XGBoost ^(41)^ eta hyper-parameter are examples. The complexity parameter (a hyper-parameter) in a decision tree ^(39)^ determines how many times the data splits, in the process of predicting class labels, into branches, which controls the size of the tree. The complexity parameter value is available in a table that is created when a tree model is executed. Is it common practice to select the complexity parameter value associated with the lowest cross-validation error corresponding to the lowest number of splits. The XGBoost eta hyper-parameter controls the rate at which new trees correct errors from a prior sequence of trees. The random forest ^(40)^ mtry parameter, as previously mentioned, determines how many predictors are used to split the data at given point. The default is the square root (rounded) of the number of predictors, but this can be manually changed. Further details on hyper-parameters are outlined under *The models:* *Logistic regression, decision tree, random forest, and XGBoost*. Tuning parameter values that generalize optimally can be determined by validating a model on data separate from that on which the model was trained; specifically by applying the trained model on out-of-sample data. This can be efficiently accomplished with resampling (see *Resampling*).

*The models:* *Logistic regression, general additive, decision tree, random forest and XGBoost*

*Logistic regression*

In binary logistic regression, as with other binary classification models, the response variable takes a binary form 0 or1. In this dummy coding, 0 = negative, which is synonymous with non-event, non-case; 1 = positive, which is synonymous with event occurrence or case (e.g. a case of pathology). As already outlined (see *Predictive classification model evaluation metrics*), with a binary response variable the predictive model output is continuous probabilities of presence/positive (P) or absence/negative of an event, such as disease. The probabilities indicate the extent of predicted case membership in either the presence of disease (P) group or absence (1-P) of disease group. Probability expresses the chance, quantitatively, that an event will happen; the number of times some event occurred divided by the total possible number of times the event could have occurred. Logistic regression is a departure from model types such as tree-based models in that it provides quantification of individual predictor and outcome relationships in the form of coefficients, the Wald statistic, and the odds ratios.

Logistic regression uses maximum likelihood (ML) to fit a model, where ML is based on the parameter (coefficient) estimates that maximize the likelihood of the *Ŷ* values closest to the actual *Y* observations ^(9)^. A logistic regression model predicts the probability of case group membership, the probability predicted from the known observation values of a predictor(s). This is depicted on y-axis in Graphic 3, which is logistic regression probability plot. The s-shaped pattern of the Graphic 3 conveys a non-linear and dichotomous relationship between predictor *X* and response variable coded 0 for controls and 1 for early PD. The probability of early PD relative to controls is on the y-axis (ranging from 0 to 1) and this is a function of (*X*) the caudate striatal binding (SBR) ratio on the x-axis. Quite simply, the predicted probabilities - the quantitative expression defining the chance that early PD will occur - are graphed against observed caudate SBR values. Caudate SBR refers to the average caudate DAT uptake (left + right caudate/2). Specifically, the predicted probability $\hat{pr}_{i}$ of being a case (of early PD) for the *i*th individual increases as (*X*) caudate SBR DAT uptake diminishes on the x-axis. Moving left to right along the x-axis the .50 or 50% cutoff occurs at a little < 3, after which the $\hat{pr}_{i}$ (of being a case) is > .50. As caudate SBR values get progressively smaller (moving left to right) the $\hat{pr}_{i}$ of being a case of early PD increases sharply as caudate SBR approaches a value of 2.


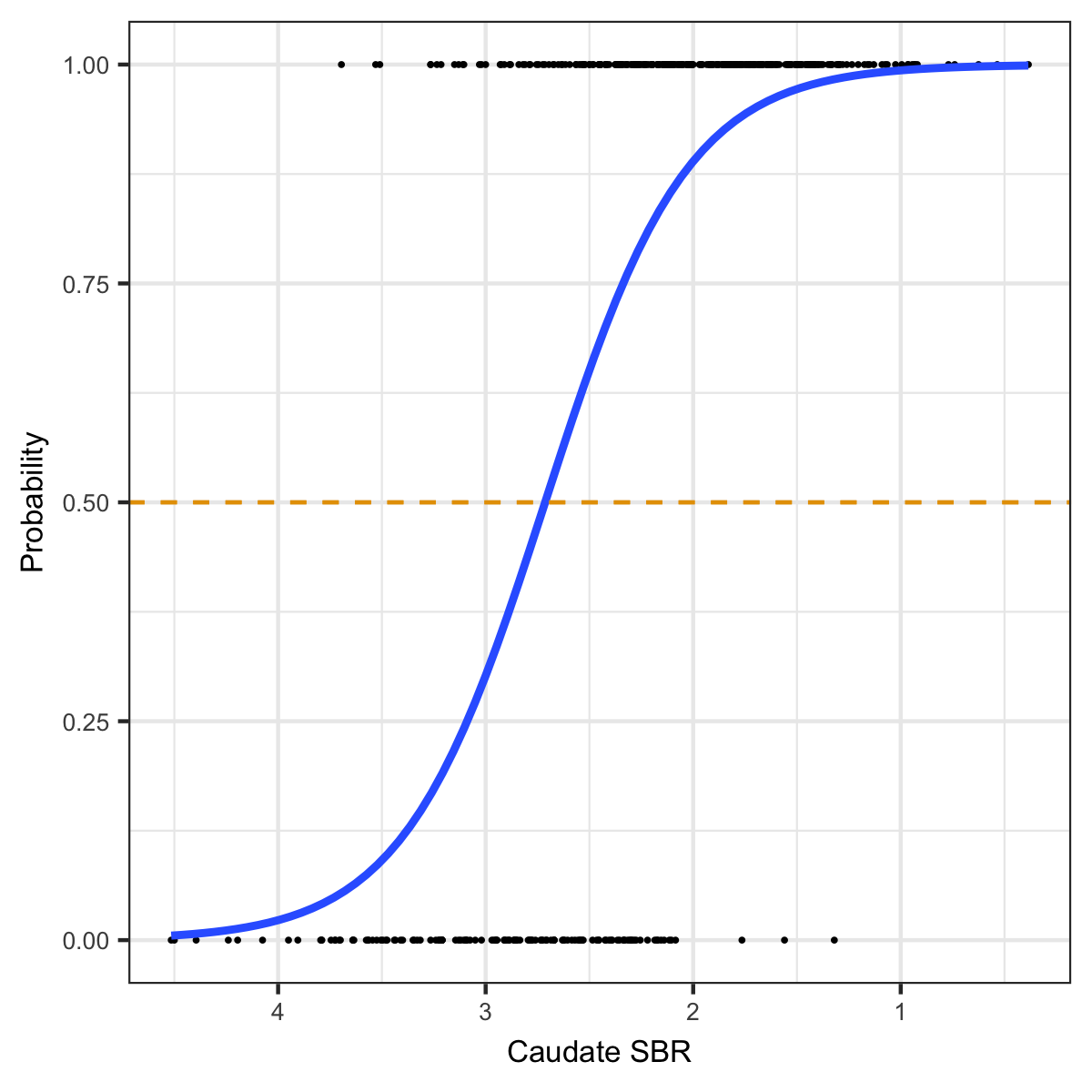


**GRAPHIC 3: Early PD vs. control caudate SBR probability plot**

SBR = SPECT striatal binding ratio: left + right caudate/2

Logistic regression is often expressed as the natural logarithm. In the fraction on the left side of the expression below the numerator is the predicted probability *Ŷ* =1 ($\hat{pr}_{i}$), and the denominator amounts to the predicted probability *Ŷ* = 0.

$$\ln\left( \frac{\hat{pr}_{i}}{1- \hat{pr}_{i}} \right)= \beta_{0 +}\beta_{1}X_{1i},$$

The *ln* on the left side of the expression is the base of the natural logarithm (~2.72); it transforms the left side of the above equation to a logit, more commonly referred to as the log-odds. This transformation expresses the distinctly non-linear relationship (a binary or categorical response variable is non linear) being modelled in a linear form ^(9)^. The right side of the above equation indicates the logit of *Y* follows a linear form; *β*_0_ is the intercept and *β*_1_ is the coefficient for predictor *X_1._* The intercept *β*_0_ is also referred to as the constant, which in the Graphic 3 example would be the predicted log-odds of caudate SBR for controls. Interpretatively, for every unit increment in predictor *X_1_* there will be a *β*_1_ increment in the log-odds of outcome (a *β*_1_ increment in the predicted probability of a case = 1, here early PD). With multiple predictors, the logistic regression predicted probability of being a case for *i*th participant can be represented in the expression$\hat{pr}_{i}= \frac{1}{1+ e^{{(\beta}_{0}+\beta_{1}X_{1i}+\beta_{2}X_{2i}+\ldots\beta_{k}X_{ki})}}$, where *e* is also the natural logarithm and the bracketed part of the denominator represents a multiple linear equation. Each regression coefficient is, as in multiple linear regression, a partial regression coefficient: the effect of *β*_1_ is interpreted controlling for other coefficients (*β*_1,_ *β*_2,_ … *β*_k_) which are held constant ^(46)^.

A predictor coefficient’s interpretation is enhanced when it is converted to an odds ratio. In most software packages this is easily accomplished with an exponential function (exp). Predictor odds ratios can be expressed as *exp*(*β*_0,_ *β*_1_*X_1i_* + *β*_2_*X_2i_+*_…_ *β_k_*X*_ki_+ β*_0_) , where exp is the exponential function, or equivalently $e^{{(\beta}_{0}+\beta_{1}X_{1i}+\beta_{2}X_{2i}+\ldots\beta_{k}X_{ki})}= \frac{\hat{{pr}_{i}}}{1- \hat{{pr}_{i}}},$ where the odds ratio is the predicted probability $\hat{pr}_{i}$ (ranging from 0 to 1) of having case status to the predicted probability of not having $({1-\hat{pr}}_{i})$case status. Interpretation of the odds ratio (OR) is straightforward: an OR = 1 indicates the absence of a predictor relationship with *Y* and all participants have equal odds of case group membership; an OR < 1 indicates that increments in a predictor reduce the odds of the case group membership; OR > 1 indicates that as the predictor increases so do the odds of case group membership. Further, a unit increase in a predictor is associated with the multiplicative amount by which the odds of case group membership (*Y*=1) is changed. For example, if predictor *X_1_* had an OR of 2.58, this would indicate that a unit increment in *X_1_* multiplies the odds of being a case by 2.58.

Measures of the how well a model fits the data include the deviance statistic and the related likelihood-ratio, the Hosmer-Lemeshow goodness of fit test ^(35)^, the Akaike information criterion (AIC) ^(65)^, and pseudo *R^2^* ^(66)^. The logistic regression example (model 1) output in Table (A) serves as reference facilitating the explanation of these model goodness of fit (GOF) measures. Table (A) also assists in differentiating logistic regression given it provides quantification of individual predictor and outcome relationships (coefficients, the Wald statistic, and the odds ratios) not readily available in the tree-based models. The Table (A) data from which the simple example logistic regression model was built used the Pima Indians data included with the mlbench package ^(67)^. For consistency, this same data set, consisting of the binary outcome diabetes positive (*Y*= 1) or negative (*Y*= 0) and continuous predictors, was used to demonstrate the decision tree classification visual (see Graphic 4). All the data was used in the decision tree example. For the regression model, and in aid of brevity, four predictors were used.

The deviance statistic is directly related to log-likelihood. The latter can be expressed as

$${\sum_{i=1}^{N} [Y}_{i}ln\left( \hat{pr}\left( Ŷ_{i} \right) \right)+\left( 1-Y_{i} \right)ln(1-\hat{pr}\left( Ŷ_{i} \right))],$$

where $\hat{pr}$ is the predicted probability that sums the probabilities associated with both the actual and predicted outcomes ^(20)^ and is grossly analogous to the SS residual (residual sum of squares) to the extent that it provides the amount of unexplained variation remaining subsequent to the fitting of the model; a poorly fitted logistic model will have a larger log-likelihood ^(9, 46)^. The deviance statistic equals negative 2 times the log-likelihood (*LL*): -2 ×*LL* = *– 2LL*.It is often used in place of the *LL*, largely because it has a chi-square distribution permitting simpler significance value calculation and comparison of logistic models, or comparison of the baseline model that represents only the constant without any predictors to a model including various predictors. New model deviance subtracted from baseline model deviance results in a difference called the likelihood ratio ^(9)^. The likelihood ratio (which actually is subtraction not a ratio) has a chi-square distribution, where the degrees of freedom equal the number of parameters (i.e. the number of predictors plus 1 for the constant) in the new model minus the number of parameters in the baseline model: χ^2^= (-*2LL*(new)) – (*-2LL*(baseline) ^(9)^. If the baseline model is the null model (with only the constant and no predictors) the constant is the only parameter, meaning it has 1 degree of freedom.

| **TABLE A: model 1, logistic regression** | | |  |  |  |  |
| --- | --- | --- | --- | --- | --- | --- |
| Variable | $\hat{\boldsymbol{\beta}}\boldsymbol{(SE)}$ | *z* | ***p*** | ***95% CI*** | ***Odds ratio*** | ***95% CI (Odds) ratio)*** |
| Intercept | -0.77 (.62) | -0.49 |  |  |  |  |
| BMI | 0.08 (.013) | 5.47 | < .0001*** | (05, .11) | 1.08 | (1.05, 1.11) |
| Pedigree | 0.83 (.29) | 2.90 | = .00412** | (.27, 1.40) | 2.29 | (1.30, 4.04) |
| Glucose | 0.03 (003) | 10.53 | < .0001*** | (.03, .04) | 1.03 | (1.03, 1.04) |
| Age | .03 (.007) | 3.94 | < .0001*** | (.02, .05) | 1.03 | (1.02, 1.05) |
|  | Null deviance: | 993.48 | on 767 df |  |  |  |
|  | Residual deviance: | 747.23 | on 763 df |  |  |  |
|  | AIC: | 757.23 |  |  |  |  |

Note: BMI = body mass index; Pedigree = diabetes insulin function

Comparing the fit of the logistic regression model 1 (see output Table A above) to the null model (with only the constant) a likelihood ratio test (model 1 deviance – null model deviance) indicated the model with predictors had a significantly better fit, *χ*^2^(4) = 246.25, *p* < .0001. The Hosmer-Lemeshow GOF test ^(35)^, which determines if predicted classification probabilities are similar to observed proportions (a Pearson Chi-squared statistic), indicated the model was a good fit to the data, *χ*^2^(2) = 2.65, *p* = .27. Note, that in the Hosmer-Lemeshow GOF test, a small chi-square value and large p-value suggest a good model fit to the data. The AIC ^(65)^ statistic, another measure of fit when comparing two models, can be expressed as AIC = -*2LL* + 2*k*, where k is the number of predictors included and *-2LL* is the deviance statistic. A smaller AIC is better reflecting that AIC (via + 2*k* in the expression) penalizes a model with more predictors. The smallest AIC occurs in models that combine the best fit and parsimonious use of predictors ^(46)^. AIC is often used as the predictor selection (or feature elimination) criterion in stepwise regression and application of AIC in a stepwise procedure has been well documented ^(35, 36)^. For example, adding the additional predictor triceps (a skin fold thickness measure) from Pima Indians data to the predictors already included in model 1 to create a new model (model 2), we find that AIC becomes a little higher (758.24). In a stepwise regression using AIC ^(68)^ (MASS package) for predictor selection, triceps was removed from the regression model. However, a different predictor may have both improved model fit while also maintaining a low AIC, in which case the stepwise procedure would have retained the newly added variable in a five variable model. The final model GOF measure considered is the McFadden pseudo *R^2^* ^(66)^ (see Cohen et al. 2003 for a discussion of linear regression variance vs. pseudo variance of logistic regression). Pseudo *R^2^* (*pR^2^*) can be expressed as ${pR}^{2}=1-\left[ \frac{\ln\left( LLM \right)}{\ln\left( LL0 \right)} \right],$where $ln\left( LLM \right)$ is the fitted model log likelihood and $ln\left( LL0 \right)$ is the null model (just the constant) log likelihood. McFadden pseudo *R^2^* ranges between 0 and 1; a value > .2 is considered satisfactory while a value < .2 is inadequate to explain the target outcome. The original four predictor model in Table A has a *pR^2^* = .248, while the five predictor model (not shown), where the triceps measure was added, has a *pR^2^ =* .249. So will the AIC criterion did not retain the triceps variable it inclusion in the model marginally increase model explanation of outcome. Where parsimonious models are preferred, the four-predictor model would likely be used given the additional predictor in five-predictor model only marginally increased the pseudo *R^2^* value. With respect to the coefficients, and interpreting just the predictor pedigree (a diabetes insulin function), a unit difference in pedigree, holding other predictors constant, is associated with .83 increase in the log-odds of diabetes; an effect that significantly differs from zero, z = 2.90, p = .004. A unit difference in pedigree multiplies the odds of diabetes 2.29 (95% CI 1.30, 4.04). It warrants note that the z-score, often referred to as the Wald (*W*) statistic is $W= \frac{\hat{\beta}}{\hat{se}(\hat{\beta)}}$, where the coefficient $\hat{\beta}$ is divided by the coefficient standard error.

Also, and with respect to coefficients, is consideration of coefficient standardization and scaling. Continuous or semi-continuous (integer rating scales with response scored over a range, e.g. 0 to 10) predictors can be converted to z-scores and used in a logistic model ^(69)^. While this will not alter model goodness of fit measures (e.g. deviance), these predictors are all now measured commonly in standard deviations; i.e. a one standard deviation change in a predictor corresponds to the change in number of standard deviations in outcome. However, a binary (0, 1) predictor (such as female and male) converted to a z-score understandably loses its original interpretive clarity. In regards to scaling, semi-continuous integer rating scales coving a large range (e.g. 0 to 40) have smaller coefficient estimates relative to binary range variables like gender (0, 1) or a predictor with a 1-5 range. On a 0 to 40 scale 1-unit change covers just .025 of the scale, while a 1-unit change in 1-5 range predictor covers a considerably greater extent of the scale: 0.2 or 1/5^th^ of the scale. Consequently both coefficients and odds ratios of the predictor with the small-scale range will be more impressive. To facilitate more appropriate interpretation, semi-continuous rating scale predictors can be rescaled, whereby a given predictor is rescaled to have a binary (0 to 1) range by dividing each score by the range maximum. All semi-continuous scaled predictors will then have a 0 to 1 range with coefficients and odds ratios based on the same scale ^(46)^. In the Table A model 1, there were no semi-continuous rating scales nor were standardized versions of the predictors provided.

As already noted and demonstrated, unlike the tree-based models (decision tree, random forest, and XGBoost), logistic regression parameters (coefficients) have readily available Wald (z-scores) and p-values and odds ratios that quantify the relation between each predictor and outcome. Such per predictor quantification can be of great value but comes at the price of a few assumptions that must be met for results to be valid. These assumptions, again in aid of brevity, are not tested for the example model using the Pima Indians data. However, the general assumption and diagnostic guidelines for logistic regression warrant brief outline.

The observations must be independent, such that there must be a lack of autocorrelation and hence independence of errors (residuals are not correlated); the same participants can not be measured across two time points. This can be assessed with the Durbin-Watson test ^(70)^ adapted for logistic regression ^(71, 72)^. There is also a requirement that allows for only a small amount or no multicollinearity. Highly correlated (e.g. *r* >= .75) predictors should normally be removed at the outset ^(1)^. The extent of multicollinearity can also be assessed with a logistic regression variation of the variable inflation factor (VIF)^(71)^. A VIF of > 5 associated with any predictor indicates problematic amount of multicollinearity. Logistic regression is also sensitive to outliers and influential cases.

Recommended logistic regression case-wise diagnostics to examine the effect of outliers include assessment of discrepancy, leverage, and influence measures. Specifically, case discrepancy is determined using the (externally) studentized residual, which measures the extent of discrepancy between observed and predicted *Ŷ* values. A commonly adopted threshold cut-off value is ± 2 (other cut-off points have also been recommended; see Cohen et al., 2003 ^(46)^). For Cook’s distance, which is a measure of given case’s global influence on the model, the widely used threshold value of 1 is typically adopted. With respect to leverage, a measure of how far case *i* observed is from the mean of the predictor(s), a recommended cut-off value can be calculated with $3M_{h}=3(k+1)/n$ ^(73)^, where $M_{h}$ is mean leverage for the predictor, *k* is the number of predictors and *n* is the number of cases. In addition, leverage in logistic model, should be restricted to the predicted probability of cases between .10 and .90 ^(35)^.

Finally, with logistic regression there is an assumption of linearity of the logit, which means there must be a linear relationship between the logit of outcome and continuous predictors. This can be assessed graphically ^(74)^ but has traditionally been tested using the Box-Tidwell transformation ^(75)^. The Box-Tidwell test amounts to incorporating in the model predictor interaction terms that are the cross-product of a given predictor and its natural logarithm (e.g. *X_1_** log(*X_1_*)). Any predictors that show significant (*p* < .05) interaction with their natural log are regarded by the Box-Tidwell test as violating linearity of the logit. In the event a predictor violates this assumption, it is often converted from a continuous to percentile-based categorical variable such as a quintiles or quantiles. This is a practice common in epidemiological research that also allows convenient framing of the relation between binary outcome and low-medium-high variable levels ^(76-79)^. However, the choice of categorizations is arbitrary, may miss portions and hence characteristics in the data, and may fail to produce sufficiently sensitive models ^(80-82)^.

The general additive model (GAM) is, like tree-based models, a non-parametric alterative to logistic regression that does not make any assumption of linearity between a predictor and the response variable^(83)^. Uniquely, while a GAM is nonparametric it combines both linear and non-linear generalized linear model link structures (lm, binomial, poisson etc.). As such, GAM models could be characterized as a bridge between parametric and non-parametric models ^(84)^. This is achieved by capturing the impact of a predictor variable with non-linear properties via a nonparametric smoothing function. The smoothing function can take the form of several basis functions – known transformation functions such splines (e.g. cubic splines, smoother splines, thin plate splines, etc.).

In regression, a predictor is multiplied by its regression coefficient. This also occurs in a GAM, but any predictor can alternatively be multiplied by a smoothing function, such as a spline; the smoother spline replaces the predictor coefficient ^(85)^. A GAM could be represented as$g\left( \mu_{i} \right)= \beta_{0}+ s_{1}\left( {X_{i}}_{1} \right)+\ldots s_{p}\left( X_{ip} \right)+ \varepsilon_{i}$. The variable *g* represents the transformation of *y* to linear terms, as occurs for example in logistic regression where a categorical response variable is linearized by transformation to logit form. The *g* variable then is analogous to the link function in the generalized linear model. The $\beta_{0}$ is the intercept, $\varepsilon_{i}$ is the error, but $s_{1}\left( {X_{i}}_{1} \right)+\ldots s_{p}\left( X_{ip} \right)$ are nonparametric smoothing functions that lend GAMs their distinctive capacity. An assumption of a GAM is that a smoother function can be estimated in a scatter-plot smoother ^(83)^.

Consider a logistic regression classification GAM to predict (the probability) of pathology, *P* (*y*=1|*X*) vs. *P* (*y*=0|*X*), which includes predictors ${X_{i}}_{1}$ and ${X_{i}}_{2}$. If ${X_{i}}_{1}$ violated the assumption of linearity of the logit it could be wrapped in a smoothing function. And, if ${X_{i}}_{2}$ did not violate this assumption it could be run in the same model but without the need to have a nonparametric smoothing function applied to it. Such a model could be expressed as $\log\left( \frac{p\left( X \right)}{1-p\left( X \right)} \right)= \beta_{0}+ s_{1}\left( {X_{i}}_{1} \right)+ \beta_{2}({X_{i}}_{2})+ \varepsilon_{i}$, where ${X_{i}}_{1}$ is wrapped in a nonparametric smoothing function, such as a form of spline, but ${X_{i}}_{2}$ is not and remains parametric. Also, in a GAM, smoothing occurs with respect to partial residuals; the smoothing fit is found that most suitably represents the relationship between a given predictor and the partial residuals controlling for other predictors.

A smoothing function typically retains more information than percentile-based variables such as quantiles. While this does likely increase sensitivity of the model it can also lead to a tendency of overfitting ^(86, 87)^. The more closely a smoother function follows the pattern of data the more degrees of freedom are used up (more degrees of freedom are added to the model). Figuratively, this can be conveyed by a smoother in the form of a wiggly line passing through points in a scatterplot, as in Graphic 4 (B). In a GAM, the segments forming the curve in such a scatterplot are the predictors separately fitted to polynomial functions, and knots delimit these predictor/segments. In the case of a cubic spline for example, *k* knots uses 4 + *k* degrees of freedom. The more points in a scatterplot the wiggly line hits, the higher the degrees of freedom contributed to the model and the greater flexibility the model has, but such flexibility incurs a propensity to overfit. The lines in Graphic 4 plot the same simulated data, but the GAM spline curve in Graph 4 B conforms much more closely to the shape of the data than the linear method in Graphic 4 A, and the gam spline curve in B hits may more data points. A curve passing through more points (as in Graphic 2 B) is certain to capture more variation in the training data but too close adherence to training data variation may result in a model that generalizes poorly.


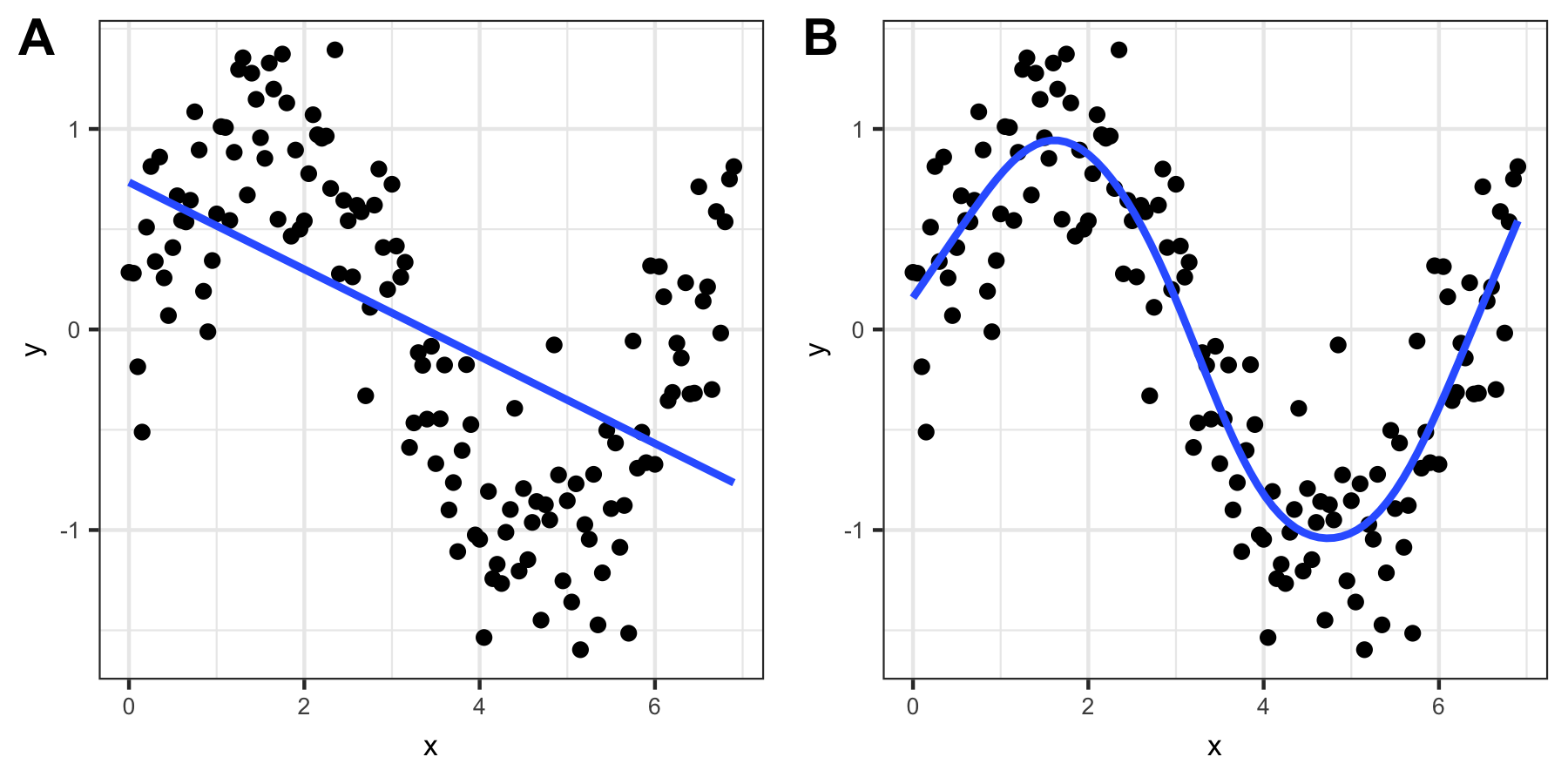


**GRAPHIC 4:** A = linear method line; B= gam spline curve

In short, overfitting occurs when a model learns trends specific to the training data set and will not generalize well to new, unseen samples. While this can occur in almost any model, here with respect to the smoothing function, to counter this propensity the smoothness level of a smoothing function can be adjusted by a smoothing parameter *λ*, $s_{\lambda}\left( X \right)$, where $s_{\lambda}$ represents a smoothing function and *λ* denotes the smoothing parameter that exerts control over the extent of smoothing and hence the degrees of freedom. The degrees of freedom used approximate the number of points a curve hits. Consider Hastie and Tibshirani’s ^(85)^ expression of a cubic spline function summarizing the dependence of y on x,

$$\sum{(yi-f\left( x_{i} \right))}^{2}+ \lambda\int f^{'}'({x)}^{2}dx.$$

Employing only the first part of the latter in a curve to minimize the difference between *y_i_* and *f(x_i_),* specifically using only $\sum{(yi-f\left( x_{i} \right))}^{2}$, the curve could take the excessive wiggly form of Graphic 1 (B), which has no smoothing, hits all points, and the points hit would approximate the considerable degrees of freedom used. The $\lambda$, however, penalizes “wiggliness” ($\int f^{'}'({x)}^{2}$ measures the extent of wiggle). There is, as might be expected, a trade-off in this adjustment between “wiggliness”and smoothness of a curve: when *λ* = 0 “wiggliness” of the curve is maximized as is the fit and as *λ* is increased the curve becomes smoother (and degrees of freedom decrease as the smoothing parameter increases). In short, a smoothing function’s coefficients are penalized to ideally limit the extent of curve wiggle in order to satisfy the dual GAM objective of minimizing the degrees of freedom while also maximizing goodness of fit.

In the mgcv package^(88)^, smoothing parameters can be estimated from the data by generalized cross-validation (GCV), maximum likelihood, or restricted maximum likelihood (REML). The GCV is a variation of leave-one-out cross-validation (LOOCV). Normally, in LOOCV models are trained the same number of times as there are data points; *N* data points means there will be *N* separate training instances on the entire data-set except for one sample- the leave-one-out sample. Prediction is made on that left out sample and an average across the *N* is computed. However, GCV is an approximation of actual LOOCV that amounts to a linear transform of the Akaike information criterion (AIC). For details see Wood^(87)^. It warrants note, that a binomial logistic GAM fitted with GVC smoothness selection will include an Un-biased Risk Estimator (UBRE) score in the output, which is in essence the GVC score provided when the *Y* is known as is the case when it is binomial ^(89)^. Alternatively, smoothing parameter selection with REML involves treating wiggly spline parts as random effects terms within the likelihood framework of random effects ^(87)^. The REML fitting method while having greater computational expense is more robust to under-smoothing compared to the CGV method (Wood, 2006 *Generalized Additive Models: An Introduction with R*. Chapman and Hall/CRC). Smaller values of UBRE or REML indicate better fitting models.

A GAM typically has distinct inner and outer loop iterative operations. While the outer loop functions to maximize the overall model fit selecting parameters making the data most probable (as in maximum likelihood), the inner loop is involved in smoothing of individual predictors using a smoothing function $s_{\lambda}\left( X \right)$ that optimizes the fit for a given smoothed predictor relative to the partial residuals (i.e. the fit to the data of ${X_{i}}_{1}$ controlling for other predictors).

Procedurally, recommended steps to diagnose a GAM include initially fitting the model, extracting the deviance residuals, and then checking that a smoothed term’s degrees of freedom are appropriate ^(90)^. In the mgcv package ^(88)^ the degrees of freedom for a basis function smoothing term, such as a spline, defaults to 10 (*k*-1= 9 estimated degrees of freedom). Also, a smoothed term’s degrees of freedom (*k*) is automatically governed by choice of smoother penalization. For example, in the latter mentioned package if the GCV smoother constraint is selected it automatically selects a smooth term’s degrees of freedom and in addition determines the number of “knots” which delimit predictors in a GAM. The estimated degrees of freedom, however, should be checked. Where *k* represents the upper limit on a smoother term’s estimated degrees of freedom, an estimated degrees of freedom approaching *k*-1 warrants checking if altering *k* provides more explanatory power ^(91)^. It warrants mention that the caret package (Kuhn,et al., 2019), while providing a general wrapper for the mgcv GAM does not currently allow specification of GAM formula, k and the smoothed versus parametric terms are automatically determined and can not be modified.

The logistic regression GAM model produces a term referred to as deviance explained, which is a pseudo *R^2^* goodness of fit measure. This is the same as McFadden pseudo *R^2^* ^(66)^. Greater wiggle in a GAM equates to greater reduction in deviance and improved model fit. Again, however, there is the trade-off between extent of model fit and generalizability of the model. In addition, using residual deviance a logistic regression GAM can be directly compared to a logistic regression GLM model (using anova with a Chi-squared test). Further, AIC values of each model type can also be compared. Of course, performance metrics on validation data such as ROC AUC, accuracy, sensitivity and specificity can also be assessed.

The increased sensitivity of GAM should be weighed against heightened interpretation difficultly. A smoothed variable in a GAM, while provided with a p-value, does not retain the regression parameters- the coefficients. This complicates interpretation. Also, the implementation of a GAM is more complex than logistic regression. Moreover, where the outcome of a GAM constitutes only a small improvement relative to a simpler model logistic regression model, the latter is preferable ^(85)^. Finally, assessment of variables as percentile-based and as smoothed functions can be useful ^(80)^. The former provides a preliminary perspective and simpler interpretation of the data while the latter provides a more comprehensive examination of the data.

*Decision tree*

Decision tree regression and classifier types exist. Accordingly, decision trees are also called classification and regression trees (CART). The open source implementation of CART in *R* is provided in the “*rpart*” package.

An example of an *rpart* ^(39, 92)^ classification tree is shown below. The model was constructed in *rpart* using the readily available Pima Indians data set included with the mlbench package ^(67)^. Those negative for diabetes are labeled “neg” and those in the positive class are labeled “pos”.


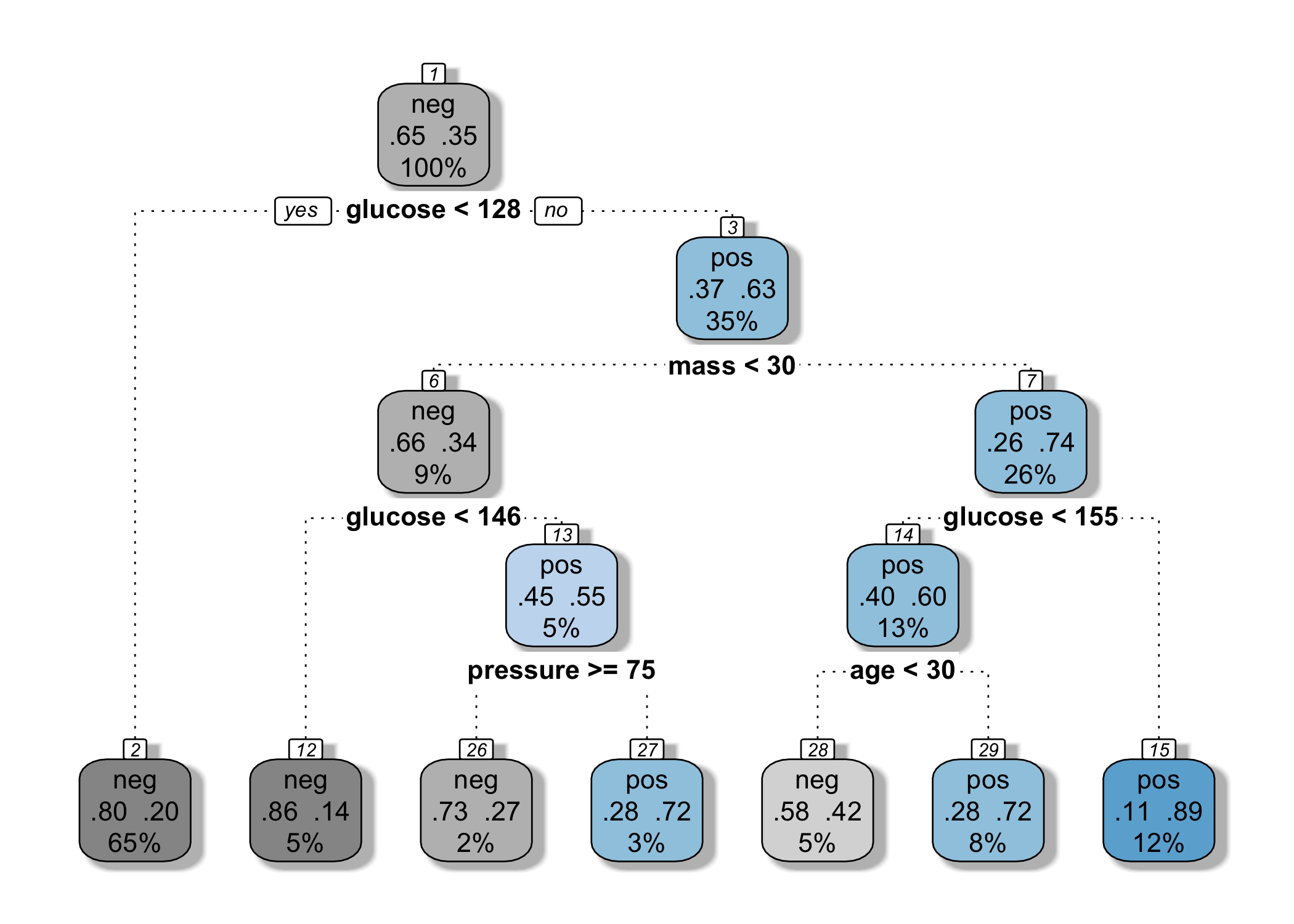


The root node (rectangle) at the top of the tree represents the entire data set sample or population. The top of each node displays the classification, the mid-node shows the probability of the class at that node, and the lower node value is the percentage of observations at the node. Starting at root node at the top, 65% do not have diabetes (neg) and 35% are diabetic. The most important predictor discriminating diabetes, and hence the neg or pos classification label, is glucose. Each split follows the same pattern: the data split is based on the variable that makes the largest contribution to reducing heterogeneity of a node. Here, glucose is the most important classification criterion. The second most important criterion is body mass index. Out of 65% of observations, those with a glucose level of < 128 have an 80% probability of not having diabetes (bottom grey leaf on the left). In general, nodes other than the root node are termed decision nodes, where a decision to spit data occurs. The terminal nodes or leaves at the bottom represent the final outcome. Returning to the tree above, those whose glucose exceeds 128 have a 63% probability of being positive for diabetes, and those whose body mass index exceeds 30 have a 74% chance of being positive for diabetes. Nodes, as is evident, carry the predictor names, and branches or arcs extending from nodes include class labels (e.g. case or non-case, positive or negative).

In a method called recursive partitioning, the *rpart* algorithm repeatedly segments or splits data into multiple sub-partitions (sub-nodes) resulting in homologous sub-partitions. Two new nodes are created after each split and each new node is made up of a subset of the observations. With default settings, *rpart* will split the data as many times as possible and splitting continues until a given stopping criterion is satisfied, which shortens or “prunes” the tree.

As already noted, decision tree spits in the data are made based on the predictor that makes the greatest contribution to reducing outcome variable *Y* (e.g. case vs. non-case, or positive vs. negative) heterogeneity. A “majority vote” for each predictor results in a prediction-based split. The rules that govern data splitting are underpinned by the objective to achieve purity of a node to arrive ideally at a leaf that is a single, homogenous class. Such a node has an impurity equal to zero; impurity ranges from 0 to 1, which is from lowest impurity to highest impurity. Impurity is typically quantified by cross-entropy, the Gini index (also referred to as Gini impurity) and classification error. The latter is perhaps intuitively the simplest to understand and represents the proportion of misclassified training data-set observations. The classification error is expressed by
$E=1- \max_{k} (\hat{p}_{mk}$), where $\hat{p}_{mk}$ is the proportion of misclassified *k*th class training observations from the *m*th region. However, the Gini index and entropy are more sensitive measures ^(2)^. The Gini index can be expressed as

$$Geni=\sum_{k=1}^{k} \hat{p}_{mk}(1- \hat{p}_{mk})$$

; cross-entropy can be expressed as

$$D= -\sum_{k=1}^{k} \hat{p}_{mk}\log\hat{p}_{mk}$$

A small Gini index value for a given node results from $\hat{p}_{mk}$ values all close to 0 and indicates relative purity of the node; that the node has largely observations from one class and therefore a low misclassification rate. Similarly, with respect to cross-entropy, and since 0 ≤ $\hat{p}_{mk}$ ≤ 1 and therefore 0 ≤ - $\hat{p}_{mk}\log\hat{p}_{mk}$, a small cross-entropy (a value close to 0) indicates relative purity of the *m*th node. Although the Gini index or cross-entropy indices are more sensitive measures of tree node purity, the classification error rate is the preferred prediction accuracy measure of the pruned final tree (see *Classification error*).

The *rpart* algorithm allows for either the Gini index or entropy based rule methods; which is preferable is debatable but it is generally agreed that this depends on the data and research objective. Splitting in r*par*t employs the Gini-based impurity index by default to select splits. A limitation of *rpart* is that, irrespective of the splitting rule, the algorithm does not necessarily find the optimal global tree because the split rule is applied locally at each split using a local stage predictor without consideration of subsequent splits and their associated predictors ^(92)^.

In general, a model using variables that too closely conform to the peculiarities of a given sample learns patterns (including noise) the can be overly ingrained in the model. When this same classifier is then applied to unseen data it typically has poor predictive accuracy because it has not learned to generalize beyond training data patterns. Any model could be susceptible to overfitting in this regard (See bias-variance trade-off in *Factors impacting predictive modelling*). An overfitted decision tree may develop relatively rigid rules from too close adherence to training data specifics, which can result in branches with sparse data not part of an unseen validation data-set. The *rpart* decision tree method of helping control overfitting is pruning (stopping or reversing growth), which reduces a tree to a smaller or sub-tree. Tree pruning is controlled through specific hyper-parameters monitored by *rpart* that can be tuned or adjusted, such as the minimum number of observations required to attempt a split at a given node (minsplit = 20, by default) and the minimum number of observations required in a terminal node (minbucket = 1/3^rd^ of minisplit by default), and the complexity parameter (*cp*), which is a cost function; it determines the “price” of misclassification and provides an indication of error.

The *cp* could be viewed as roughly analogous to the residual sum of squares in multiple linear regression and the deviance statistic in logistic regression in so far as all these metrics provide an indication of model error. In *rpart* the *cp* parameter is the essential tree pruning control and it has a default of *cp* = .01. The *cp* parameter penalizes the tree for each additional split and arrives at the optimal tree depth. Briefly elaborating, let *α* represent the *cp* parameter as part of a cost function C*α(T),* where *T* is the number of terminal nodes (leaf nodes). The cost function C*α(T)* combines instances that are misclassified (error) with the number of tree leaf nodes. When *α =* 0 an un-pruned and fully-grown tree occurs (typically a very overfitted model). An increment in *α* (*cp* parameter) incurs a cost or penalty proportional to the number of leaf nodes in the tree ^(92)^.

The default processing in *rpart* maximizes the number of splits and hence tree growth, then subsequently prunes the tree using (internal) cross-validation (default is 10-fold). Each tree spit and hence each node has a *cp* coefficient and an associated cross-validated error. The *cp* coefficient with the lowest cross-validated prediction error should be selected. This will likely prune off a given split that does not improve the model fit and serve to reduce overfitting. A generally agreed optimal *cp*-value can be arrived at using the earliest split with the lowest cross-validated error (xerror). The appropriate *cp* value can be extracted programmatically, or simply selected from a table of model associated *cp* values, or selected automatically by the *caret* package ^(1)^. In practice, the *cp* value with the lowest split number and xerror is then used within a control in *rpart* to produce the final pruned tree. Initially it is typical to execute an un-pruned tree, or a tree with relaxed parameter settings. For example: model 1 <- rpart(class~., data = data-set, control = rpart.control(cp =.001, minsplit = 10), method = “class”). This can be plotted to view the classification scheme and the cross-validated *cp* can be selected from the model 1 results formatted in a table, the cptable. Again, the *cp* value with the lowest split number and xerror would be used in final pruned tree model.

The importance of a predictor to decision tree class discrimination is measured by *rpart’s* built-in goodness of split metric. The goodness of split is the sum of a given predictor’s usage as the primary basis of a split in all nodes, which is also the sum of the predictor’s contribution to decrease in impurity. A predictor’s usage includes its use as a surrogate (i.e. when it is substituted for another variable’s missing data-point). This sum is transformed in to a percentage score (100% being the maximum). Details of the *rpart* algorithm have been well documented and provide added insight to this model type ^(39, 92)^.

Trees are overly sensitive to minor alterations in the training data. This can be addressed by ensemble methods that allow splitting decisions to be averaged over multiple tree constructions. The prediction accuracy of a decision tree model can be enhanced by ensemble methods that utilize trees as building blocks (e.g. bagging, random forest, XGBoost). These tree enhancement methods are outlined the subsequent Random forest and XGBoost sections.

*Random forest*

A decision tree model typically has higher variance (and hence higher error on unseen data) but lower bias relative to a logistic regression model. Bias in a logistic regression model, as already discussed, stems from its assumptions, which are that there is a linear relationship between continuous predictors and the logit outcome, independence of errors holds, and predictor correlation is not high. A decision tree makes no such assumptions and has consequently low bias. However, decision trees generally have higher variance than logistic models; tree growth is highly sensitive to alterations in the training data, and as already noted in the *rpart* overview, for *rpart* and decision tress in general, a split rule is applied locally at each split using a local stage predictor without consideration of subsequent splits and their associated predictors ^(2, 92)^. Model instability and diminished predictive performance can result ^(1)^. There are ensemble algorithms that combine predictions from multiple models to reduce variance and improve predictive accuracy. Ensemble methods include bootstrap aggregation (bagging) random forest and XGBoost.

The bagging algorithm ^(93)^ takes multiple *B* bootstrap samples from the training data with replacement, trains the algorithm on each *bth* bootstrapped sample, and then arrives at an aggregated average prediction ^(1, 2)^. The aggregation results in an averaging of model predictions, with each model having the same weight in determining into which group or class the sample is classified. This averaging process reduces variance across individual model predictions and typically improves accuracy. In addition, in the process of bootstrap sampling, and with each model built in the collective or ensemble, some samples (approximately 1/3^rd^) are left out and are referred to as out-of-bag samples. Models for each bagged tree are fit to approximately two-thirds of observations. Because the out-of-bag samples are not used to fit a given model, they are used to test an individual model’s predictive accuracy (or test error), and an average out-of-bag (OOB) based metric (the out-of-bag estimate) provides a measure of performance accuracy for the entire aggregated ensemble that is typically similar to that derived from a cross-validation method. The OOB error then, is a bootstrap estimate of the aggregated or mean model prediction error on a given sample *x_i_* only utilizing trees without *x_i_* in their bootstrapped sample ^(2)^. It is a form of internal cross-validation to mitigate overfitting. While bagging may improve prediction accuracy, interpretation of bagged trees is, understandably, complicated relative to a single decision tree. Variables of importance in a solitary decision tree are evident in numeric and graphic output but such is not the case with bagging a large number of trees (e.g. 50 iterations or more). But given all predictors are assessed for consideration at all splits of every singly tree, it follows that the structure of bagged trees can be similar. As a consequence high tree correlation can result and inhibit variance reduction of predictions ^(1, 2)^. By contrast, in random forest there is a random selection of predictors that avoids the bias that a decision tree or simple bagging could introduce.

The original the random forest algorithm was unveiled at a conference in 1995 ^(94)^, further developed subsequently ^(95, 96)^, with the appearance of the best known version in 2001 ^(40)^. Each tree in a random forest results from a randomly selected subset of predictors (as opposed to all predictors considered for all splits of every tree in bagging), which introduces randomness mitigating tree correlation. Typically, random forest has reduced OOB prediction and test error relative to bagging ^(1, 40)^. Moreover, as in a decision tree classification analysis, a tree has a “vote” and the class with the most votes is selected as the final outcome. In sequential steps, the number of trees in random forest ensemble is chosen (e.g. 500 is the default, but a range of 500 to 3000 is optional); for every split in the data, train a tree (a bagged tree in the ensemble) on a randomly selected bootstrap subset sample *m* of predictors *p*; the best among *p* predictors is chosen to split the data; there is a vote for the class from each tree; the predicted class outcome with the most votes is the final prediction. The randomly selected subset *m* or fraction of the number of predictors *p* is a referred to as the forest tuning parameter $m_{try}$. The default $m_{try}$ setting for a classification analysis is the square root of the number of predictors (i.e. $m_{try}= \sqrt{p}$).

A random forest does not avoid overfitting by pruning; decision trees, as already outlined, do adopt pruning via selection of sub-trees with the lowest cross-validated error. Random forest has been demonstrated as protected from overfitting ^(40)^, and in application this is achieved by optimizing the tuning parameters, notably the $m_{try}$ parameter. Certainly, the default for $m_{try}$ and other parameters work remarkably well “out of the box” despite the apparent lack of theoretical support for the default settings ^(97)^. Optional parameter alterations have been recommended to optimize performance: assessing five different *m_try_* evenly spaced values ranging from 2 to the number of predictors *p*; experimenting with the number of trees (ntree) grown by starting at 1000 and increasing the ntree number until performance no longer improves ^(1)^.

The trees in a random forest are small ‘weak’ trees. Multiple small weak decision trees can be combined in parallel and averaged (analogous to reaching a majority decision or vote) to form one strong learner. A weak learner ( a bagged tree in the ensemble) is one with accuracy that is marginally greater than chance (just > 50%); selection of a weak learner with low bias reduces variance ^(40)^. Multiple small weak decision trees can be combined in parallel (analogous to reaching a majority decision or vote) to form one strong learner.

A random forest model has heighted complexity of interpretation, though this is largely overcome by a built-in function that estimates variable importance to the model. However, the estimated importance of a variable may be unreliable with multicollinearity (Kuhn 2013; <https://stats.stackexchange.com/questions/59124/random-forest-assumptions>). Two measures of feature importance in random forest are Mean Decreased Accuracy and Mean Decreased Gini values ^(40, 98)^. Reduced model accuracy based on out-of-bag samples when a predictor’s values are randomly permuted (i.e. altering values but not removing the predictor) is the Mean Decreased Accuracy. The Mean Decreased Gini impurity (here not to be confused with model performance) for a given predictor over all trees is an indicator of that predictor’s importance. Larger values associated with a predictors Mean Decreased Accuracy measure and a predictors Mean Decreased Gini indicate greater predictor relevance to model classification. The two measures of variable importance are highly correlated and it has been demonstrated that both measures have a bias favouring categorical predictors with more categories ^(99)^ . Overall, random forest is one of the highest performing analysis methods ^(100-102)^, and an undeniable appeal of this algorithm is that it often performs well “out of the box” requiring little in the way of model tuning expertise.

*XGBoost*

The extreme gradient boosting (XGBoost) package ^(41)^ is a variant of gradient tree boosting ^(103)^. Garnering a plethora of accolades in recent years, winning solutions employing XGBoost have proven dominant in machine learning challenges such as Kaggle, the KDD Cup, and in the Higgs machine learning challenge ^(104-106)^. In general, XGBoost is known for it speed and accuracy.

As with Random Forest, gradient boosting combines and averages multiple weak individual tree models in an ensemble to produce an improved learner. However, unlike Random Forest, models are not made from entirely random subsets of features and data. Rather, model building is sequential. Initially, model prediction errors for every observation are determined. A model is then built predicting the errors and this process is reiterated adding such error finding models to an ensemble to reduce misclassification rate. It is also worth distinguishing that while random forest mitigates overfitting largely by training models on randomly sampled data, XGBoost includes penalization of complex models (regularization) to control overfitting.

Data for XGBoost must be in numeric format. Prior to model implementation, categorical variables (predictors/features) need to be transformed to binary (0 1) form using one-hot encoding. Further, data should conform to a matrix format, and the dependent or outcome variable is simply a vector of labels. Importantly, XGBoost only does regression, and regression trees rather than decision trees are used in classification models. This means that each regression tree leaf has a continuous (rather than dichotomous) score. As such, in binary classification, if the probability of some observation (label) datum is < 0.5 it is classified as 0 but if it is > 0.5 it is classified as 1 ^(107)^. Drawing a parallel to random forest, XGBoost also uses tree ensembles, however, and as just noted, the trees used in XGBoost are regression rather than the decision trees used in random forest.

XGBoost makes use of both loss and regularization terms to find optimal model parameters, which are the coefficients *θ* that provide the best fit to outcome *y_i_* (actually a vector of labels) and (a matrix) of training data *x_i_*. A so-called objective function is defined to measure the fit of the model to the training data. The objective function includes *L* loss and Ω regularization terms. In a binary classification analysis, log loss can be used to penalize group membership misclassifications. Log loss *L* (negative log loss) can be generally expressed as

$$L = -\frac{1}{N} \sum_{i=1}^{N} [y_{i} \cdot log(p\left( y_{i} \right)) +\left( 1- y_{i} \right)\cdot log (1- p(y_{i}))]$$

, where N is the number of total instances, $y_{i}$ is the *i*-th instance outcome label, and *p* is the predicted probability. Assume, say, a green group membership label has a value of 0 and a red group membership label has an outcome value of 1. Given $y_{i}=1 (red label)$, then ${1- y}_{i}=0$ (green label),$p$is the predicted probability that the *i*-th instance is red, which is 1, over all N instances. Following the equation verbatim, for every red label instance where $y_{i}=1,$*log p* is added to the loss which here is the log probability of the instance being red. Antithetically, the equation adds $log (1- p(y_{i}))$, which the log probability of an instance being green, for every green point ($y_{i}=0$). XGBoost uses a Taylor expansion version of log loss that includes first and second order derivatives ^(41)^.

Regularization, or a regularization term, controls model complexity and so helps to mitigate overfitting. To elaborate, consider a model $\hat{y}_{i}= \sum_{k=1}^{K} f_{k}\left( x_{i} \right),f_{k}\inϜ,$ where F is a set of all classification and regression trees (CARTs), *K* represents the number of trees, and *f* is a function in space F. The objective function finding the optimal parameters (optimal functions here) could be generally expressed as

$$objfunc\left( \theta\right)= \sum_{i}^{n} l\left( y_{i},\hat{y}_{i} \right)+ \sum_{k=1}^{K} \Omega(f_{k})$$

 ,where $l\left( y_{i},\hat{y}_{i} \right)$ is an abbreviated loss function measuring the predicted $\hat{y}_{i}$ and target $y_{i}$ difference , and as already defined, Ω is a regularization term controlling complexity of the trees^(107)^. In order to arrive at the model with optimized functions, the objective function is minimized. This can be expressed as

$$\mathcal{L}\left( \phi\right)= \sum_{i} l\left( \hat{y}_{i},y_{i} \right)+\sum_{k} \Omega(f_{k})$$

, where $\sum_{k} \Omega(f_{k})= \gamma T+ \frac{1}{2} \lambda{|\left| \omega\right||}^{2}$ ^(41)^. The term *ω* is a vector of leaf scores and *T* is the number of leaves and each leaf has weights (coefficients) and a penalty *λ* for this weight. Again, the $\sum_{k} \Omega(f_{k})$ term is the penalty sum of tree attributes over all trees built. This added regulation term aids in avoiding overfitting; it includes a smoothing function analogous L1 in Lasso regression and L2 Ridge regression (L1 and L2 limit the size of coefficients). The gamma in $\gamma T$is some number that can range from zero to infinitely and will depend on the loss function used (log loss in classification and residuals in regression) and the number of leaves (number of leaves in turn depends on the maximum depth of a tree). Additional parameters to guard against overfitting include the gamma parameter, maximum tree depth, and a term called minimum child weight. Gamma is the loss required to further split a tree leaf node. The function of maximum tree depth setting conveyed by its name, controls tree depth and greater depth increases the chance of overfitting. Minimum child weight acts to stop additional node splitting after a particular degree of purity is attained ^(108)^. Internal cross-validation (default is 10-fold) should be used to find the appropriate gamma, maximum tree depth and minimum child weight. Cross-validation should also be used to find nrounds (number of iterations), which is another setting parameter, and it is similar to the number of trees in random forest. Other parameters important to grasp are eta, subsample, and column sample by tree. The eta parameter (also known as a shrinkage parameter) controls learning rate and should be supported by nrounds. If nrounds is a range between 100 and 500 (100 to 1000 is a common range) then eta could fall within some sequence ranging from 2 to 10 divided by nrounds [(2:10)/nrounds] ^(109)^. The default eta is .3, the possible values range between 0 and 1, and setting the eta value higher slows down the learning rate of the model. New trees correct errors from a prior sequence of trees; the trees learn quickly which results in a tendency to overfit the training data. However, higher values of eta result in fewer corrections per every tree added to the model and so reduce likelihood of overfitting, but may also result in underfitting. Hence, to find the appropriate eta value may require assessing a range of values. Alternatively, eta, like most XGBoost parameters, can also be tuned in caret ^(44)^.

Cover, weight and gain are available to measure feature importance. As with all model types, certain features improve model accuracy while others have an antithetical effect and simply add to the error. Cover refers to the number of times a feature is used to partition data over all trees, and the total number of data instances passing through the same partitions weights it. Weight simply refers to the number of times a given feature is utilized to split data across all the trees. Gain is the feature contribution to the model determined by calculating the contribution of each feature to each tree in the model. Gain is generally regarded as the most appropriate measure of feature importance ^(107)^.A higher gain feature value indicates the feature is more important relative to other features with lower gain values. Finally, it warrants note that XGBoost implementation is a departure from the considerably simpler implementation of other models outlined in the present work. This can be attributed mainly to the added number of tuning parameters that need to be configured but also to the required data format.

References

1. Kuhn M. *Applied Predictive Modeling*. In: Johnson K, editor.: Springer; 2013.

2. Gareth J, Witten, D., Hastie T., Tibshirani, rR. An Introduction to Statisical Learning Springer Texts in Statistics; 2013.

3. Cohen J. A Coefficient of Agreement for Nominal Data. Educational and Psychological Measurement. 1960;20:37-46.

4. Landis JR, Koch GG. MEASUREMENT OF OBSERVER AGREEMENT FOR CATEGORICAL DATA. Biometrics. 1977;33(1):159-74.

5. Foody GM, Campbell NA, Trodd NM, Wood TF. DERIVATION AND APPLICATIONS OF PROBABILISTIC MEASURES OF CLASS MEMBERSHIP FROM THE MAXIMUM-LIKELIHOOD CLASSIFICATION. Photogrammetric Engineering and Remote Sensing. 1992;58(9):1335-41.

6. Liu CR, Frazier P, Kumar L. Comparative assessment of the measures of thematic classification accuracy. Remote Sensing of Environment. 2007;107(4):606-16.

7. Olofsson P, Foody GM, Herold M, Stehman SV, Woodcock CE, Wulder MA. Good practices for estimating area and assessing accuracy of land change. Remote Sensing of Environment. 2014;148:42-57.

8. Pontius RG, Millones M. Death to Kappa: birth of quantity disagreement and allocation disagreement for accuracy assessment. International Journal of Remote Sensing. 2011;32(15):4407-29.

9. Field AP. *Discovering statistics using R*. Miles J, & Field, Z., editor. London: Sage; 2012.

10. Altman DG, Bland JM. DIAGNOSTIC-TESTS-3 - RECEIVER OPERATING CHARACTERISTIC PLOTS .7. British Medical Journal. 1994;309(6948):188-.

11. Hanley JA, McNeil BJ. THE MEANING AND USE OF THE AREA UNDER A RECEIVER OPERATING CHARACTERISTIC (ROC) CURVE. Radiology. 1982;143(1):29-36.

12. Fawcett T. An introduction to ROC analysis. Pattern Recognition Letters. 2006;27(8):861-74.

13. Youden WJ. INDEX FOR RATING DIAGNOSTIC TESTS. Cancer. 1950;3(1):32-5.

14. Habibzadeh F, Habibzadeh P, Yadollahie M. On determining the most appropriate test cut-off value: the case of tests with continuous results. Biochemia Medica. 2016;26(3):297-307.

15. Ewald B. Post hoc choice of cut points introduced bias to diagnostic research. Journal of Clinical Epidemiology. 2006;59(8):798-801.

16. Lalkhen MBM, A. Clinical tests: sensitivity and specificity. Continuing Education in Anaesthesia, Critical Care & Pain. 2008;8(6).

17. Provost FF, T.; Kohavi, R., editor The case against accuracy estimation for comparing induction algorithms. International Conference on Machine Learning; 1998.

18. Freeman EA, Moisen GG. A comparison of the performance of threshold criteria for binary classification in terms of predicted prevalence and kappa. Ecological Modelling. 2008;217(1-2):48-58.

19. Tharwat A. Classification assessment methods. Applied Computing and Informatics. 2018.

20. Tabachnick BGF, L. S. Using multivariate statistics. 5th ed. Boston: Allyn & Bacon; 2007.

21. Package Tc. Classification and regression training 2019, March, 27 [Available from: <https://topepo.github.io/caret/>.

22. Efron BT, R. Bootstrap methods for standard errors, confidence intervals, and other measures of statistical accuracy. Statistical Science. 1986:54-75.

23. Bengio Y, Grandvalet Y. No unbiased estimator of the variance of K-fold cross-validation. Journal of Machine Learning Research. 2004;5:1089-105.

24. Zhang Y, Yang Y. Cross-validation for selecting a model selection procedure. Journal of Econometrics. 2015;187(1):95-112.

25. Molinaro AM, Lostritto K, van der Laan M. partDSA: deletion/substitution/addition algorithm for partitioning the covariate space in prediction. Bioinformatics. 2010;26(10):1357-63.

26. Menardi GT, N. Training and assessing classification rules with unbalanced data. Data Min Knowl Disc. 2014;28(92).

27. Chawla NV, Bowyer KW, Hall LO, Kegelmeyer WP. SMOTE: Synthetic minority over-sampling technique. Journal of Artificial Intelligence Research. 2002;16:321-57.

28. Batista GP, R.; Monard, M.;. A Study of the Behavior of Several Methods for Balancing Machine Learning Training Data. ACM SIGKDD Explorations Newsletter,. 2004;6(1):20-9.

29. Burez JVdP, D. Handling Class Imbalance In Customer Churn Prediction. Expert Systems with Applications. 2009;36(3):4626-36.

30. Jeatrakul PW, K.; Fung, C. Classification of Imbalanced Data By Combining the Complementary Neural Network and SMOTE Algorithm. Neural Information Processing; Models and Applications. 2010:152-9.

31. Van Hulse JK, T.; Napolitano, A., editor Experimental Perspectives On Learning From Imbalanced Data. Proceedings of the 24th International Conference On Machine learning; 2007.

32. Brownlee J. 8 tactics to combat imbalanced classes in your machine learning dataset 2015, August 19 [Available from: <https://machinelearningmastery.com/tactics-to-combat-imbalanced-classes-in-your-machine-learning-dataset/>.

33. Exchange S. Highly-correlated variables in random forest 2018, August 3 [Available from: <https://stats.stackexchange.com/questions/141619/wont-highly-correlated-variables-in-random-forest-distort-accuracy-and-feature>.

34. Everitt B, S.; Skrondal, A. Cambridge Dictionary of Statistics: Cambridge University Press; 2010.

35. Hosmer D, W., Jr.;, Lemeshow, S.; Sturdivant, R., X. Applied Logistic Regression. Hoboken: John Wiley & Sons, Inc; 2013.

36. Harrell F, E. Regression modeling strategies: with applications to linear models, logistic regression, and survival analysis. New York: Springer-Verlag 2013.

37. Flack VF, Chang PC. FREQUENCY OF SELECTING NOISE VARIABLES IN SUBSET REGRESSION-ANALYSIS - A SIMULATION STUDY. American Statistician. 1987;41(1):84-6.

38. Freedman DA. A NOTE ON SCREENING REGRESSION EQUATIONS. American Statistician. 1983;37(2):152-5.

39. Breiman L, Freidman, J., Olshen, R., & Stone, C. Classification and regression trees. Belmont: Wadsworth; 1984.

40. Breiman L. Random forests. Machine Learning. 2001;45(1):5-32.

41. Chen T, Guestrin, C. XGBoost: A Scalable Tree Boosting System. SIGKDD. 2016:785-94.

42. Overflow S. Difference between varimp (caret) and importance (randomforest) for random forest 2016, October 5 [Available from: <https://stackoverflow.com/questions/37888619/difference-between-varimp-caret-and-importance-randomforest-for-random-fores?rq=1>.

43. <https://topepo.github.io/>. Variable Importance 2019, March, 3 [Available from: <http://topepo.github.io/caret/variable-importance.html>.

44. Kuhn M. Package caret. In: Kuhn M, editor. Classification and regression training. CRAN2019, March, 3.

45. Castaldi PJ, Dahabreh IJ, Ioannidis JPA. An empirical assessment of validation practices for molecular classifiers. Briefings in Bioinformatics. 2011;12(3):189-202.

46. Cohen J, Cohen P, West SG, Aiken LS. Applied Multiple Regression Correlation Analysis for the Behavioral Sciences. 3r ed. ed. New Jersey: Lawrence Erlbaum Associates, Inc.; 2003.

47. Wolpert DH. The lack of A priori distinctions between learning algorithms. Neural Computation. 1996;8(7):1341-90.

48. Cramer JS. The origins of logistic regression. Tinbergen Institute. 2002:167-78.

49. Lim TS, Loh WY, Shih YS. A comparison of prediction accuracy, complexity, and training time of thirty-three old and new classification algorithms. Machine Learning. 2000;40(3):203-28.

50. van der Ploeg T, Austin PC, Steyerberg EW. Modern modelling techniques are data hungry: a simulation study for predicting dichotomous endpoints. Bmc Medical Research Methodology. 2014;14.

51. Guo FT, Wang GY, Su ZW, Liang HL, Wang WH, Lin FF, et al. What drives forest fire in Fujian, China? Evidence from logistic regression and Random Forests. International Journal of Wildland Fire. 2016;25(5):505-19.

52. Perlich C, Provost F, Simonoff JS. Tree induction vs. logistic regression: A learning-curve analysis. Journal of Machine Learning Research. 2004;4(2):211-55.

53. Kirasich KS, T.; Sadler, B. Random forest vs logistic regression: binary classification for heterogenous datasets. SMU Data Science Review. 2018;1:3.

54. Zhang Z, Ho KM, Hong Y. Machine learning for the prediction of volume responsiveness in patients with oliguric acute kidney injury in critical care. Crit Care. 2019;23(1):112.

55. Xiao J, Ding R, Xu X, Guan H, Feng X, Sun T, et al. Comparison and development of machine learning tools in the prediction of chronic kidney disease progression. Journal of translational medicine. 2019;17(1):119-.

56. Chen VC, Lin TY, Yeh DC, Chai JW, Weng JC. Predicting chemo-brain in breast cancer survivors using multiple MRI features and machine-learning. Magn Reson Med. 2019;81(5):3304-13.

57. Hong WS, Haimovich AD, Taylor RA. Predicting hospital admission at emergency department triage using machine learning. Plos One. 2018;13(7).

58. Gao C, Sun H, Wang T, Tang M, Bohnen NI, Müller MLTM, et al. Model-based and Model-free Machine Learning Techniques for Diagnostic Prediction and Classification of Clinical Outcomes in Parkinson's Disease. Sci Rep. 2018;8(1):7129.

59. Hernesniemi JA, Mahdiani S, Tynkkynen JA, Lyytikäinen LP, Mishra PP, Lehtimäki T, et al. Extensive phenotype data and machine learning in prediction of mortality in acute coronary syndrome - the MADDEC study. Ann Med. 2019:1-8.

60. Luo L, Li J, Liu C, Shen W. Using machine-learning methods to support health-care professionals in making admission decisions. Int J Health Plann Manage. 2019.

61. Shimoda A, Ichikawa D, Oyama H. Using machine-learning approaches to predict non-participation in a nationwide general health check-up scheme. Comput Methods Programs Biomed. 2018;163:39-46.

62. Tang CQ, Li JQ, Xu DY, Liu XB, Hou WJ, Lyu KY, et al. [Comparison of machine learning method and logistic regression model in prediction of acute kidney injury in severely burned patients]. Zhonghua Shao Shang Za Zhi. 2018;34(6):343-8.

63. Kaggle. XGBoost 2018 [Available from: <https://www.kaggle.com/dansbecker/xgboost>.

64. Exchange S. Classification XGBoost vs Logistic Regression 2019, March 1 [Available from: <https://stats.stackexchange.com/questions/394705/classification-xgboost-vs-logistic-regression>.

65. Akaike H. NEW LOOK AT STATISTICAL-MODEL IDENTIFICATION. Ieee Transactions on Automatic Control. 1974;AC19(6):716-23.

66. McFadden D. Conditional logit analysis of qualitative choice behavior. Frontiers in Econometrics. 1974:105-42.

67. Newman DJH, S. ; Blake, C. L.; Merz, C. J. UCI Repository of machine learning databases. 1998.

68. Venables WNR, B. D. Modern Applied Statistics with S. . New York: Spriinger; 2002.

69. Pampel FC. Logistic regression: a primer. Thousand Oaks, CA: Sage; 2000.

70. Durbin J, Watson GS. TESTING FOR SERIAL CORRELATION IN LEAST SQUARES REGRESSION .2. Biometrika. 1951;38(1-2):159-78.

71. Fox JW, S. . An {R} Companion to Applied Regression. Thousand Oaks, CA: Sage; 2011.

72. Group SC. Logistic Regression (R): San Diego State University; 2013, November 14 [Available from: <http://scg.sdsu.edu/logit_r/>.

73. Belsely DA, Kuh E, Welsch RE. Regression diagnostics. Wiley and Sons, New York, USA. 1980.

74. Zhang Z. Model building strategy for logistic regression: purposeful selection. Annals of Translational Medicine. 2016;4(6).

75. Box GEP, Tidwell PW. Transformation of the Independent Variables. <http://www.jstor.org/stable/1266288>: Technometrics; 1962. p. 531-50.

76. Thiebaut ACM, Kipnis V, Chang S-C, Subar AF, Thompson FE, Rosenberg PS, et al. Dietary fat and postmenopausal invasive breast cancer in the National Institutes of Health-AARP Diet and Health Study cohort. Jnci-Journal of the National Cancer Institute. 2007;99(6):451-62.

77. Tu JV, Austin PC, Chan BTB. Relationship between annual volume of patients treated by admitting physician and mortality after acute myocardial infarction. Jama-Journal of the American Medical Association. 2001;285(24):3116-22.

78. Vickers AJ, Bianco FJ, Serio AM, Eastham JA, Schrag D, Klein EA, et al. The surgical learning curve for prostate cancer control after radical prostatectomy. Journal of the National Cancer Institute. 2007;99(15):1171-7.

79. Vickers AJ, Savage CJ, Hruza M, Tuerk I, Koenig P, Martinez-Pineiro L, et al. The surgical learning curve for laparoscopic radical prostatectomy: a retrospective cohort study. Lancet Oncology. 2009;10(5):475-80.

80. Bennette C, Vickers A. Against quantiles: categorization of continuous variables in epidemiologic research, and its discontents. Bmc Medical Research Methodology. 2012;12.

81. Greenland S. DOSE-RESPONSE AND TREND ANALYSIS IN EPIDEMIOLOGY - ALTERNATIVES TO CATEGORICAL ANALYSIS. Epidemiology. 1995;6(4):356-65.

82. Royston P. A strategy for modelling the effect of a continuous covariate in medicine and epidemiology. Statistics in Medicine. 2000;19(14):1831-47.

83. Jones K, Wrigley N. GENERALIZED ADDITIVE-MODELS, GRAPHICAL DIAGNOSTICS, AND LOGISTIC-REGRESSION. Geographical Analysis. 1995;27(1):1-21.

84. Wegman EJ, Wright IW. SPLINES IN STATISTICS. Journal of the American Statistical Association. 1983;78(382):351-65.

85. Hastie T, Tibshirani R. Generalized additive models for medical research. Stat Methods Med Res. 1995;4(3):187-96.

86. Weinberg CR. HOW BAD IS CATEGORIZATION. Epidemiology. 1995;6(4):345-7.

87. Wood SN. Fast stable direct fitting and smoothness selection for generalized additive models. Journal of the Royal Statistical Society Series B-Statistical Methodology. 2008;70:495-518.

88. Wood SN. Package ‘mgcv'. **Mixed GAM Computation Vehicle with Automatic Smoothness**

**Estimation**2019, March 21.

89. Kim L. The Predictive Modeling Silver Bullet <https://multithreaded.stitchfix.com/2015>, July 30 [Available from: <https://multithreaded.stitchfix.com/blog/2015/07/30/gam/>.

90. Wood SN. Stable and efficient multiple smoothing parameter estimation for generalized additive models. Journal of the American Statistical Association. 2004;99(467):673-86.

91. Wood SN. Generalized additive models with integrated smoothness estimation 2019 [Available from: <https://astrostatistics.psu.edu/su07/R/library/mgcv/html/gam.html>.

92. Therneau TA, B.; Ripley, B. Recursive Partitioning and Regression Trees. CRAN; 2018.

93. Breiman L. Bagging predictors. Machine Learning. 1996;24(2):123-40.

94. Ho TK. Random decsion forests. Proceedings of the 3rd International Conference on Document Analysis and Recognition Montreal, QC1995. p. 278-82.

95. Amit Y, Geman D. Shape quantization and recognition with randomized trees. Neural Computation. 1997;9(7):1545-88.

96. Ho TK. The random subspace method for constructing decision forests. Ieee Transactions on Pattern Analysis and Machine Intelligence. 1998;20(8):832-44.

97. Scornet E. Tuning parameters in random forests. Proceedings and Surveys. 2018;60:144-62.

98. Liaw AW, M. Package random forest. CRAN2018, March 25.

99. Strobl C, Boulesteix AL, Zeileis A, Hothorn T. Bias in random forest variable importance measures: Illustrations, sources and a solution. Bmc Bioinformatics. 2007;8.

100. Collins L, Griffioen P, Newell G, Mellor A. The utility of Random Forests for wildfire severity mapping. Remote Sensing of Environment. 2018;216:374-84.

101. Pal M. Random forest classifier for remote sensing classification. International Journal of Remote Sensing. 2005;26(1):217-22.

102. Rodriguez-Galiano VF, Ghimire B, Rogan J, Chica-Olmo M, Rigol-Sanchez JP. An assessment of the effectiveness of a random forest classifier for land-cover classification. Isprs Journal of Photogrammetry and Remote Sensing. 2012;67:93-104.

103. Friedman JH. Greedy function approximation: A gradient boosting machine. Annals of Statistics. 2001;29(5):1189-232.

104. Kaggle. What is XGBoost 2018 [Available from: <https://www.kaggle.com/dansbecker/xgboost>.

105. KDnuggetts. XGBoost 2017 [Available from: <https://www.kdnuggets.com/?s=XGBoost>.

106. ATLAS. Machine Learning Wins the Higgs Challenge 2014 [Available from: <https://atlas.cern/updates/atlas-news/machine-learning-wins-higgs-challenge>.

107. XGBoost. XGBoost R tutorial 2016 [Available from: <https://xgboost.readthedocs.io/en/latest/R-package/xgboostPresentation.html>.

108. Chen T, Guestrin, C. Extreme gradient boosting. CRAN2019, March 11.

109. Zhang O. Winning data science competitions 2016, July 16 [Available from: <https://www.slideshare.net/ShangxuanZhang/winning-data-science-competitions-presented-by-owen-zhang>.
