# Supplementary material for: Non-motor Clinical and Biomarker Predictors Enable High Cross-Validated Accuracy Detection of Early PD but Lesser Cross-Validated Accuracy Detection of Scans Without Evidence of Dopaminergic Deficit
Source: Front Neurol. 2020 May 11;11:364. doi: 10.3389/fneur.2020.00364 (PMC7232850; doi:10.3389/fneur.2020.00364)
Supplement: Data Sheet 3 — R-code. [file Data_Sheet_1.PDF]

```
# Model R code for research paper entitled:
# Non-motor Clinical and Biomarker Predictors Enable High Cross-validated
# Accuracy Detection of Early PD but Lesser Cross-validated Accuracy Detection of
# Scans Without Evidence of Dopaminergic Deficit

# Please copy this code, and paste it into an R script file.
# To run these models first set a working directory to where you have stored the
data: e.g. setwd('~/Documents/my_data')

# libraries
library(car); library(DMwR); library(grid); library(psych); library(QuantPsyc);
library(corrplot)
library(tidyr); library(MASS); library(pscl); library(ROSE); library(rpart)
library(dplyr); library(ggplot2); library(effects); library(randomForest)
library(caret); library(rpart.plot);  library(rattle); library(pROC); library(xgboost)

# The models were built using a 64-bit Mac OS cpu; processor 3.2, 4-cores.
# Consistent seeds were set prior to partitioning (random stratified) data
# and model exection. As such all model outcomes are 100% reproducible. Note,
# however, that we found caret package XGBoost model tuning did require at least a
4-core
# or equivalent system.

# The caret package (citation(package= "caret")) was used for random stratified
# partitioning of the data and to find optimal tree-model (decision-tree, random
forest,
# and XGBoost) tuning hyper-parameters using k-fold cross-validation:
# specifically, 10-fold cross validation repeated 5 times (50 models created).
# The main model performance criterion was the AUC metric: the model
# with the highest AUC was selected. Note the GLM (logistic regression)
# parameters (coefficients) are not tuned by the caret package. In addition,
# at the time of this writing, the caret package did not permit specification
# of GAM formula particulars deemed important to model optimization.
# For this reason the caret package was not used to tune GAM model parameters.

# Features were selected initially by model-based feature selection, and
# models that had the highest AUC.

# Note, to ensure models did not model unique case identifiers
# data sets used did not include unique row (ie.subject) indentifers. When
# reading data from csv files this results in an added index file labeled
# "X". This "X" column should be removed (as it is in the code) prior to running any
model.
# If you want to add a unique identifying row, this syntax can be used:
# data_set$id<- seq.int(nrow(data_set)). The data set can then
# be saved as a csv file and called avoiding the added "X" column.
```

```
# Alternatively, once a csv file is read into R, it can be saved in typical fashion:
# save(data_set,file="data_set.Rda");
# load("datPN.Rda")

# Data sets should be loaded prior to executing models. Models can be located
# by searching for the following:
# early PD/control classification:GLM_1; GAM_1; tree_1 (Decion tree); rf_1 (random
forest); xgb_1
# early PD/SWEDD classification starts line 1265: glm_2;GAM_2, tree_2; rf_2

# Descriptive data set data (includes all data, and all three groups: early PD,
controls, SWEDD)

# Final data after filtering for only cases with complete data
# for all features and low hemoglobin contamination.
# Includes all three groups. Note, striatal SPECT imaging and UPDRS III data were
also
# included for desciptive statistics but were not modelled.
datfabr<-read.csv('gp3DatNumFactcorrecteAug4.csv', header=T, na.strings = c(""))
datfabr$X<- NULL
colnames(datfabr) # PATNO is cell id and removed so it will not be modeled
table(datfabr$ENROLL_CAT)
# HC    PD    SWEDD
# 130   295    43

# Same as datfabr but PATNO retained for tracking predicted SWEDD vs PD
# in curated data (12-36 months)
# post baseline
datFinalpatno<- read.csv("datfinalpatnoMay3checkrevUp.csv", header=T)
datPN <- datFinalpatno
colnames(datPN)
datPN$X<-NULL
sapply(datPN, function(x) sum(is.na(x)))
head(datPN$ENROLL_CAT)

table(datPN$ENROLL_CAT)

# Modelled Features
# library(dplyr)
#  features<- datfabr %>%
#    select(ENROLL_CAT, age, gend, EDUCYRS, RevUpsit.sum,
# Abeta1_42,CSFasyn, pTau,tTau, NP1CNST,SumTrait,
# rbdSum, gdsSum, epwSum, MCATOT)
#  head(features)
# # colnames(features)
# # # "ENROLL_CAT"   "age"        "gend"        "EDUCYRS"     "RevUpsit.sum"
```

```
# # # "Abeta1_42"    "CSFasyn"      "pTau"        "tTau"        "NP1CNST"
# # # "SumTrait"     "rbdSum"      "gdsSum"      "epwSum"      "MCATOT"
#  ncol(features[, -1]) #14 featurs


# HC vs PD
# 1_hcpd:
hcpd.dat<- read.csv("hcpd_dat.csv", header= T, na.strings = c(""))
hcpd.dat$X<- NULL
head(hcpd.dat)


# random stratified split of data for healthy controls (HC) vs early PD patients (PD)
library(caret)
set.seed(9876)
index.70<- createDataPartition(y = hcpd.dat$ENROLL_CAT,
                        p = 0.70, # 70 of data allowcated to training the model
                        times = 1, #just 1 split
                        list = FALSE)
train1 <- hcpd.dat[index.70,]
test1 <- hcpd.dat[-index.70,]

# optionally read-in previously saved and partitioned data
#train1<- read.csv("train1.csv", header=T)
#test1<- read.csv("test1.csv", header=T)

# data instance numbers
table(train1$ENROLL_CAT)
# HC  PD
# 91 207
table(test1$ENROLL_CAT)
# HC PD
# 39 88

# data proportions all the same
round(prop.table(table(hcpd.dat$ENROLL_CAT)), 2)
#   HC   PD
# 0.31 0.69
round(prop.table(table(train1$ENROLL_CAT)), 2)
# HC   PD
# 0.31 0.69
round(prop.table(table(test1$ENROLL_CAT)), 2)
# HC   PD
# 0.31 0.69
```

```
# PD vs. Scans without evidence of dopamine deficit (SWEDD)
# 2_pdsw:
pdsw2<- read.csv("pdsw2.csv", header = T, na.strings = c(""))
pdsw2$X<-NULL
colnames(pdsw2)
ncol(pdsw2[, -1]) # note 15 features here as PATNO left in for
# longitudinal analyses but not modelled
table(pdsw2$ENROLL_CAT)
# PD SWEDD
# 295   43

# random stratified split of data for PD and SWEDD
library(caret)
set.seed(9876)
indexPDSW1<- createDataPartition(y = pdsw2$ENROLL_CAT,
                          p = 0.50, # 50%
                          times = 1, #just 1 split
                          list = FALSE)
train50a <- pdsw2[indexPDSW1,]
test50a <- pdsw2[-indexPDSW1,]

# optionally read-in previously saved and partitioned data
#train50a<- read.csv(""train50a.csv, header=T)
#test50a<-  read.csv("test50a.csv", header=T)


# data instance numbers
table(train50a$ENROLL_CAT)
# PD SWEDD
# 148   22

round(22/(22+148),2) #0.13 SWEDD minority rate

table(test50a$ENROLL_CAT)
# PD  SWEDD
# 147   21

# all have similar proportions
round(prop.table(table(pdsw2$ENROLL_CAT)),2)
# PD SWEDD
# 0.87  0.13
round(prop.table(table(train50a$ENROLL_CAT)),2)
# PD SWEDD
# 0.87  0.13
round(prop.table(table(test50a$ENROLL_CAT)), 2)
# PD SWEDD
```

```
# 0.88  0.12


#GLM_1
# features extracted from Training data (train1);
# features determined by stepwise regression using AIC
# and AUC (features resulting in the highest AUC used in model)

colnames(top6LR<- train1[, c(1,2,5,8,10,12,15)])
# 1] "ENROLL_CAT"  "age" "RevUpsit.sum" "pTau"   "NP1CNST"
# [6] "rbdSum" "MCATOT"

#Linarity of the logit
# diag_ Box-Tidwell
# lin log test pred * log of itself : MoCA was non-linear
LL1<- top6LR
colnames(LL1)
LL1$ageInt<- (LL1$age * log(LL1$age))
LL1$revInt<- (LL1$RevUpsit.sum* log(LL1$RevUpsit.sum))
LL1$pTauInt<- (LL1$pTau * log(LL1$pTau))
LL1$CNST_Int<- (LL1$NP1CNST * log(LL1$NP1CNST))
LL1$RBD_int<- (LL1$rbdSum* log(LL1$rbdSum))
LL1$Moca_int<- (LL1$MCATOT* log(LL1$MCATOT))

# can not run all variables at once: will cause perfect separation
set.seed(3450) # CNST not in this mod but next
summary(LLtest<- glm(ENROLL_CAT~ RevUpsit.sum + revInt +
            rbdSum + RBD_int +
            pTau + pTauInt  +
            age + ageInt +
            MCATOT + Moca_int,
           family = binomial(link = 'logit'),
           data = LL1))
# MoCA violated linearity of the logit
# MCATOT      -88.8130    31.2351  -2.843  0.00446 **
# Moca_int     20.4733     7.2044   2.842  0.00449 **

# Graphing linear relationship between the continuous features and logit of
outcome
library(dplyr)
# Fit the logistic regression model
summary(mod_LLa <- glm(ENROLL_CAT ~., data = top6LR,
            family = binomial(link= 'logit')))
pr1 <- predict(mod_LLa, newdata= NULL, type = "response")
head(pr1)
predClasses <- ifelse(pr1 > 0.5, "PD", "HC")
```

```
head(predClasses)

library(dplyr)
top6<- top6LR %>%
  dplyr::select_if(is.numeric)
head(top6) # correct all but DV
preds1<- colnames(top6)# get colnames

# Bind the logit, tidying and convert to long format
top6<- top6 %>%
  mutate(logit = log(pr1/ (1-pr1))) %>%
  gather(key = "preds1", value= "predictor.value", -logit) # converts to long format;  -
logit » option will simply ignore the « logit » column when gathering the predictors.
head(top6) # long format

ggplot(top6, aes(logit, predictor.value))+
  geom_point(size = 0.5, alpha = 0.5) +
  geom_smooth(method = "loess") +
  theme_bw() +
  facet_wrap(~preds1, scales = "free_y")

# GLM_1 data with MoCA as quartiles and also has rescaled versions of
# constipation and rbd which included zero values
# CNST and rbdSum variables have meaningful 0 value So recaled to start a 1 not
zero
# this allows option to scale and center for standardization of predictors
# (see line 338 HCPD.R)

# MoCA was converted to quartiles (e.g. https://rpubs.com/kaz_yos/logistic-
linearity)
# Note, there were only 3 cases equal to the base (Q1) score of 21 but there were 71
cases (all early PD)
# less than 27, which was the score marking the first quartile (.25).
# To reasonably represent the lower scores, the first two quantiles, Q1 and Q2, were
merged,
# leaving a single low range for all instances with a score < 27.
# This resulted in a 3-level factor representing the MoCA low (21-26),
# midrange or median (28) and upper level scores (29-30). See Supporting
# information V for details.

# GLM and GAM data sets
LR1_rs1<- read.csv("LR1_rsCNSTrbdMocaQ3.csv", header = T)
LR1_rs1$X<-NULL
head(LR1_rs1) # includes centered vars and mocha.ch1 helmert
# and importantly new factor MocaQ3
```

```
# Includes MoCA in dummy format; k-1
LR1_rsDumMo<- read.csv("LR1_rsMocaDummies.csv", header=T, na.strings= c(""))
LR1_rsDumMo$X<- NULL

# GLM test data: given same names as LR1_rs1 and LR1_rsDumMo
test1a<- read.csv("test1MocaQ3dum.csv", header = T)
test1a$X<- NULL

# Note, in the LR1_rs1 data set,the variables constipation and RDBQ were
# are also provide in scaled (to start from 1 rather than zero) formate.
# This was done for potential future use but the models
# can be executed with the orignial rbdSum and NP1CNST varables (as below),
# which include base minimal zero values for constipation and RDBQ or
# using the scaled version of these variables; the outcome will be indentical.

# GLM_mod_1
set.seed(3450)#
summary(modLR_1<- glm(ENROLL_CAT~ age + RevUpsit.sum +
                    rbdSum +MoMidrange+ MoHigh+
                    NP1CNST + pTau,
                  data= LR1_rsDumMo,
                  family = binomial(link = 'logit')))

#              Estimate Std. Error z value Pr(>|z|)
# (Intercept)   0.27499    1.05961   0.260  0.79523
# age          -0.03493    0.01786  -1.956  0.05047 .
# RevUpsit.sum  0.27043    0.03558   7.600 2.97e-14 ***
#   rbdSum       0.23452    0.07926   2.959  0.00309 **
#   MoMidrange   -0.92691    0.47399  -1.956  0.05052 .
# MoHigh       -0.21589    0.42578  -0.507  0.61212
# NP1CNST       0.58949    0.36864   1.599  0.10980
# pTau         -0.07942    0.03370  -2.357  0.01842 *

# Null deviance: 366.75  on 297  degrees of freedom
# Residual deviance: 194.42  on 290  degrees of freedom
# AIC: 210.42

library(pscl)
pR2(modLR_1) #0.4698650

# With conversion of Moca to quantiles fit slightly better;
# some data lost, hence need for the GAM
# here is the model without MoCA (MCATOT) converted to quartiles
summary(modglm_hcpd6<- glm(ENROLL_CAT~.,
                    data= train1[, c(1,2,5,8,10,12,15)],
                    family = binomial(link = 'logit')))
```

```
# Null deviance: 366.75  on 297  degrees of freedom
# Residual deviance: 191.49  on 291  degrees of freedom
# AIC: 205.49

# other assumption tests see lines 733 -907 HCPD.R available on request

# GLM1_pred

# Mod AUC
predMod<- predict(modLR_1, newdata = NULL, type = "response")
head(predMod)
library(ROSE)
roc.curve(LR1_rsDumMo$ENROLL_CAT,predMod, main= "" )
#Area under the curve (AUC): 0.920

thr<-.5
predGLMmodlab<- factor(ifelse(predMod > thr, "PD", "HC"))
head(predGLMmodlab)
confusionMatrix(predGLMmodlab, LR1_rs1$ENROLL_CAT, positive = "PD")
library(pROC)
(rocLG_mod<-roc(LR1_rsDumMo$ENROLL_CAT,predMod, # same as ROSE, as
expected
            # using group of interest
            levels = levels(test1$ENROLL_CAT)))
auc(rocLG_mod)# Area under the curve: 0.9205
ci.auc(rocLG_mod) #95% CI: 0.8877-0.9533 (DeLong)
coords(rocLG_mod, x = "best", best.method="youden")
coords(rocLG_mod, x = "best", best.method="topleft") # better balance here

# threshold specificity sensitivity
# 0.7079819   0.9120879   0.8115942

thr<-0.7079819
predGLMmodlab2<- factor(ifelse(predMod > thr, "PD", "HC"))
confusionMatrix(predGLMmodlab2, LR1_rs1$ENROLL_CAT, positive = "PD")
# Sensitivity : 0.8116
# Specificity : 0.9121


# validation/test set
colnames(test1a[, c(2,5, 12,20,21,10,8)])
pred.LR.test <- predict(modLR_1, newdat= subset(test1a, select= c(2,5,
12,20,21,10,8)),
                  type = "response")

head(pred.LR.test) #vector of class probabilities
```

```
#  0.9981938 0.2162027 0.3831425 ...

thr<- .50
predLabGLM1<- factor(ifelse(pred.LR.test > thr, "PD", "HC"))
head(predLabGLM1)
library(caret)
confusionMatrix(predLabGLM1, test1a$ENROLL_CAT, positive = "PD")
# Reference
# Prediction HC PD
# HC 34  9
# PD  5 79
# Accuracy :    0.8898
# Kappa :       0.7482
# Sensitivity : 0.8977
# Specificity : 0.8718


library(pROC)
(rocLG2<-roc(test1a$ENROLL_CAT, pred.LR.test,
        levels = levels(test1$ENROLL_CAT)))

ci.auc(rocLG2)
auc(rocLG2) #Area under the curve: 0.9068

# optimized coords
coords(rocLG2, x = "best", best.method = "youden")
# threshold specificity sensitivity
# 0.4620365   0.8717949   0.9090909

thr<- 0.4620365 # lowering thrs (e.g. .40) will improve sens at cost of lower spec
# thr<- .2 # lowering thrs (e.g. .40) will improve sens at cost of lower spec
predbestLR1<- factor(ifelse(pred.LR.test > thr, "PD", "HC"))
head(predbestLR1)
confusionMatrix(predbestLR1, test1$ENROLL_CAT, positive = "PD")
# Sensitivity:  0.9091
# Specificity : 0.8718

rocLG_1<- plot.roc(test1$ENROLL_CAT,pred.LR.test,
            levels = levels(test1$ENROLL_CAT),
            ci= TRUE, of = "thresholds", # CI for threshold
            thresholds = "best", #
            print.thres = "best",
            print.auc= TRUE,
            legacy.axes =TRUE,# highlight threshold in plot
            #print.auc= TRUE,
```

```
        #print.thres.pattern = "%.3f (Spec = %.2f, Sens = %.2f)", # % special
character. .2f just print to 2 floating point decimal places
        print.thres.cex = .8,
        cex.axis= .6,
        cex.lab=.8)
dev.off()



# GAM_1; used the same features as GLM
library(nlme)
library(mgcv)
colnames(LR1_rs1)

set.seed(3450)# k= -1 is default of 10-9 = 9
modgam1d<- gam(ENROLL_CAT~ s(age, bs="tp", k=3) + s(RevUpsit.sum, bs="tp",
k=6) +
       s(rbdSum, bs="tp", k=4) + s(MCATOT, bs="tp", k=5) +
       s(NP1CNST, bs= "tp", k=4) + s(pTau, bs= "tp",k=4),
      data= LR1_rs1,
      method = "REML",
      family= binomial(link= 'logit'))
summary(modgam1d)
# edf Ref.df Chi.sq  p-value
# s(age)         1.000  1.000  4.339   0.0372 *
# s(RevUpsit.sum) 2.160  2.669 53.537 1.42e-11 ***
#s(rbdSum)      1.000  1.000  4.703   0.0301 *
#s(MCATOT)      2.739  2.945  3.577   0.2875
#s(NP1CNST)     1.763  2.097  5.394   0.0749 .
#s(pTau)        1.000  1.000  3.825   0.0505 .

# R-sq.(adj) =  0.585   Deviance explained = 55.9%
# -REML = 90.787  Scale est. = 1        n = 298

gam.check(modgam1d) # edf did not closely approch k-1 for any predictors.
# Respose vs Fitted graph is not useful here
# The defualt k= 10 (k-1= 9 maximum) produced
# good results but k values in modgam1d above
# had best AUC


anova(modLR_1,modgam1d, test= "Chi" )# GAM sig better
# Resid. Df Resid. Dev     Df    Deviance  Pr(>Chi)
# 1    290.00     194.43
# 2    287.34     161.90 2.6628   32.522 2.494e-07 **
```

```
# model AUC
predgamMod1<- predict(modgam1d, type= "response")
head(predgamMod1)

(rocGAM1_mod<-roc(LR1_rs1$ENROLL_CAT,predgamMod1,
            levels = levels(LR1_rs1$ENROLL_CAT)))
#Area under the curve: 0.946
ci.auc(rocGAM1_mod) #95% CI: 0.9217-0.9702 (DeLong)
auc(rocGAM1_mod) #Area under the curve: 0.946

# model AUC
predgamMod1<- predict(modgam1d, type= "response")
head(predgamMod1)

(rocGAM1_mod<-roc(LR1_rs1$ENROLL_CAT,predgamMod1,
            levels = levels(LR1_rs1$ENROLL_CAT)))
#Area under the curve: 0.946
ci.auc(rocGAM1_mod) #95% CI: 0.9217-0.9702 (DeLong)
auc(rocGAM1_mod) #Area under the curve: 0.946

thr<- .5
# convert to labels
predModgam1<- factor(ifelse(predgamMod1 > thr, "PD", "HC"))
head(predModgam1)
confusionMatrix(predModgam1, LR1_rs1$ENROLL_CAT, positive = "PD")
# Sensitivity : 0.9130
# Specificity : 0.8022

coords(rocGAM1_mod, x = "best", best.method = "youden")
# threshold specificity sensitivity
# 0.6818034   0.9230769   0.8502415
library(ROSE)
roc.curve(LR1_rs1$ENROLL_CAT,predgamMod1) #same OK


# prediction_GAM1 valdation/test
colnames(test1[, c(2,5,8,10, 12,15)])

# test GAM on test set
predgam1<- predict(modgam1d, newdata= subset(test1, select=c(2,5,8,10, 12,15) ),
type= "response" )
head(predgam1)

thr<-.5
# convert to labels
```

```
predlabGam1<- factor(ifelse(predgam1 > thr, "PD", "HC"))
head(predlabGam1)
confusionMatrix(predlabGam1, test1$ENROLL_CAT, positive = "PD")

# Reference
# Prediction HC PD
# HC 34  9
# PD  5 79
# Accuracy : 0.8898
# Kappa : 0.7482
# Sensitivity : 0.8977
# Specificity : 0.8718

library(pROC)
(rocGAM1<-roc(test1$ENROLL_CAT,predgam1,
         levels = levels(test1$ENROLL_CAT)))
#Area under the curve: 0.928
library(ROSE)
roc.curve(test1$ENROLL_CAT,predgam1 )#Area under the curve (AUC): 0.928

auc(rocGAM1)# Area under the curve: 0.928
ci.auc(rocGAM1)# 95% CI: 0.8778-0.9783 (DeLong)

coords(rocGAM1, x = "best", best.method = "youden")
# threshold specificity sensitivity
#0.5341743   0.8974359   0.8977273
coords(rocGAM1, x = "best", best.method = "topleft")# same

thr<- 0.5341743  # like RF .5341667
# convert to labels
predlabGam1<- factor(ifelse(predgam1 > thr, "PD", "HC"))
head(predlabGam1)
confusionMatrix(predlabGam1, test1$ENROLL_CAT, positive = "PD")
# Prediction HC PD
# HC 35  9
# PD  4 79

#Accuracy :  0.8976
#Kappa : 0.7678
# Sensitivity : 0.8977
# Specificity : 0.8974

plot.roc(test1$ENROLL_CAT, predgam1,
      levels = levels(test1$ENROLL_CAT),
      main= "GAM",
      ci= TRUE, of = "thresholds", # CI for threshold
```

```
        thresholds = "best", #
        print.thres = "best",
        print.auc= TRUE,
        legacy.axes =TRUE,# highlight threshold in plot
        #print.auc= TRUE,
        #print.thres.pattern = "%.3f (Spec = %.2f, Sens = %.2f)", # % special character.
.2f just print to 2 floating point decimal places
        print.thres.cex = .8,
        cex.axis= .6,
        cex.lab=.8)

dev.off()

# tree_1
### rpart_
library(rpart)
library(rpart.plot)

# Goodness of split and AUC from k-fold resampling in caret
# optimized feature selection.

# Mode-based features resulting in highest AUC
colnames(treeNative6<- train1[, c(1,5,15,2,12,6,8)])#
# 1] "ENROLL_CAT"   "RevUpsit.sum" "MCATOT"      "age"         "rbdSum"
# [6] "Abeta1_42"    "pTau"
#write.csv(treeNative6, file="treeNative6.csv")

tree1<- rpart(ENROLL_CAT~ age +  RevUpsit.sum + rbdSum + MCATOT +
             Abeta1_42  + pTau, data = train1,
           control = rpart.control(minbucket = 3, cp=.001,
                          maxdepth = 5), method = "class")
tree1$cptable
#the complexity parameter value associated with the lowest cross-validation error
corresponding
#to the lowest number of splits selected
min.xerror <- tree1$cptable[which.min(tree1$cptable[,"xerror"]),"CP"]
min.xerror # 0.02197802

tree1$variable.importance
tree1$variable.importance
#RevUpsit.sum      MCATOT       pTau      rbdSum        age    Abeta1_42
#     55.619330    12.119080    8.051632    6.704339    6.488315    6.075371


# caret selection of cp
metric <- "ROC"
```

```
controltree <- trainControl(method="repeatedcv",
                    number=10,
                    repeats=5,
                    classProbs = TRUE,
                    savePredictions = "final",
                    # search="random", # default grid search in caret proved better here
than search = random
                    summaryFunction = twoClassSummary)

set.seed(3450)#
tree2caret6<- train(ENROLL_CAT~ .,
              data = treeNative6, # top
              method= "rpart",
              metric = metric,
              tuneLength= 3,
              trControl = controltree) #
tree2caret6 # same cp as selected by native tree
# cp         ROC       Sens      Spec
# 0.02197802  0.8644630  0.8082222  0.8718571

tree2caret6$finalModel$variable.importance
tree2caret6$finalModel$variable.importance
# RevUpsit.sum      MCATOT        age      rbdSum    Abeta1_42       pTau
# 52.996617    11.399080    4.648826    4.648826    1.859530    1.394648
varImp(tree2caret6)
# Overall
# RevUpsit.sum 100.000
# MCATOT       49.787
# rbdSum       16.303
# pTau         13.487
# Abeta1_42     4.377
# age           0.000


# Mod AUC
predModtree<- predict(tree2caret6, newdata = NULL, type = "prob")
head(predModtree)
library(pROC)
(rocTree1_mod<-roc(treeNative6$ENROLL_CAT,
             predict(tree2caret6, newdat= NULL,
                   type = "prob")[, "PD"], # using group of interest
             levels = levels(treeNative6$ENROLL_CAT)))
#Area under the curve: 0.872
auc(rocTree1_mod)# 0.872
ci.auc(rocTree1_mod) #95% CI: 0.831-0.913 (DeLong)
library(ROSE)
```

```
predVecTree1<- predModtree[, "PD"]
head(predVecTree1)
roc.curve(train1$ENROLL_CAT, predVecTree1) # Area under the curve (AUC): .872
coords(rocTree1_mod, x= "best", best.method ="youden")

# Tree1 validation/test
colnames(treeNative6<- train1[, c(1,5,15,2,12,6,8)])#
colnames(test1[, c(5,15,2,12,6,8)])
predict_caret1 <- predict(tree2caret6$finalModel,
                    newdata = subset(test1,
                                select= c(5,15,2,12,6,8 )),
                    type = "prob")

thr <- .5
# converted to labels
problabs<- factor(ifelse(predict_caret1[, "PD"]> thr, "PD", "HC"))
head(problabs)
confusionMatrix(problabs, test1$ENROLL_CAT, positive = "PD")
# Sensitivity : 0.8182
# Specificity : 0.8974

library(pROC)
(rocTree1<- roc(test1$ENROLL_CAT,
           predict(tree2caret6$finalModel,
                 newdata = subset(test1,
                             select= c(5,15,2,12,6,8) ),
                 type = "prob")[,"PD"],
           levels = c("HC","PD")))
#Area under the curve: 0.8601

auc(rocTree1)# Area under the curve: 0.8601
ci.auc(rocTree1)
vecTree<- predict_caret1[, "PD"]
roc.curve(test1$ENROLL_CAT, vecTree) # .860 same

# Optmized threshold
coords(rocTree1, x = "best", best.method = "youden") #
# threshold specificity sensitivity
# 0.5860331   0.8974359   0.8181818


thr<-  0.5860331

predtreekf <- factor( ifelse(predict_caret1[, "PD"] > thr, "PD", "HC") )
confusionMatrix(predtreekf, test1$ENROLL_CAT, positive = "PD")#
# Sensitivity : 0.8182
```

```
# Specificity : 0.8974
# Kappa : 0.6592

roctreekf<- plot.roc(test1$ENROLL_CAT,
          predict(tree2caret6$finalModel, newdata = subset(test1,
                                          select= c(5,15,2,12,6,8) ),
              type = "prob")[,"PD"],
          levels = c("HC","PD"),
          ci= TRUE, of = "thresholds", # CI for threshold
          thresholds = "best", #
          print.thres = "best",
          print.auc= TRUE,# highlight threshold in plot

          #print.thres.pattern = "%.3f (Spec = %.2f, Sens = %.2f)", # % special
character. .2f just print to 2 floating point decimal places
          print.thres.cex = .8,
          cex.axis= .6,
          cex.lab=.8)
dev.off()

# rf_1 Random Forest1
# control; find best mtry hyperparameter
library(caret)
metric <- "ROC"
ctrl.tb<- trainControl(
  method = "repeatedcv",
  number = 10,
  repeats = 5,
  search = "random",
  classProbs = TRUE, # note class probabilities included
  savePredictions = "final",
  allowParallel = TRUE,
  summaryFunction = twoClassSummary)

# rf1 features: selected by Rf importance and
# features that produced mod with highest AUC
colnames(Natrf1<- train1[, c(1, 5, 15, 12,6, 7, 2)]) #
# "ENROLL_CAT"   "RevUpsit.sum" "MCATOT"      "rbdSum"
# "Abeta1_42"   CSFasyn"      "age"
#  write.csv(Natrf1, file= "Natrf1.csv")

# rf1
library(randomForest)
set.seed(3450)
rf1<- randomForest(ENROLL_CAT~ .,
         data =Natrf1, # top 6 from train1
```

```
              ntree= 3000,
              mtry= 1,
              nodesize =1,
              keep.forest = FALSE,
              importance = TRUE)

randomForest::importance(rf1)
#                   HC        PD MeanDecreaseAccuracy MeanDecreaseGini
# RevUpsit.sum 104.746935 78.439997          109.844659        43.75576
# MCATOT       52.725061 18.535121           47.056811        14.91820
# rbdSum       20.475157 20.284455           27.783661        14.39493
# Abeta1_42     2.721190  3.310753            4.229279        16.02956
# CSFasyn       3.425899  4.230626            5.471082        15.61443
# age          -6.036461  6.990939            1.255458        14.43114

#
# Caret model
set.seed(3450)
rf6_nTb<- train(ENROLL_CAT~ .,
            data =Natrf1 ,
            method= "rf",
            metric = metric,
            trControl = ctrl.tb,
            ntree= 3000,
            nodesize = 3, importance = TRUE)
# default nodesize = 1 but 3 used (marginally less overfitting)

rf6_nTb$finalModel# OOB 14.09
rf6_nTb$finalModel$tuneValue # mtry 1
rf6_nTb$finalModel$ntree #3000
rf6_nTb$finalModel$importance
#                     HC           PD MeanDecreaseAccuracy MeanDecreaseGini
# RevUpsit.sum  0.2061789394 0.0701172820        0.1110491982        41.38246
# MCATOT       0.0776313831 0.0112032058        0.0313013208        13.69592
# rbdSum       0.0300958711 0.0119341716        0.0174868617        12.96029
# Abeta1_42    0.0002994742 0.0004678583        0.0004879846        13.61975
# CSFasyn      0.0028739754 0.0010333425        0.0016244879        13.39878
# age         -0.0060080879 0.0036339267        0.0007455218        12.25267

varImp(rf6_nTb)
# Importance
# RevUpsit.sum   100.0000
# MCATOT          40.8281
# rbdSum          23.8295
# CSFasyn          1.5892
# age              0.2025
```

```
# Abeta1_42       0.0000

predModRF<- predict(rf6_nTb, newdata = NULL, type = "prob")
(rocRFModb<-roc(Natrf1$ENROLL_CAT,
            predict(rf6_nTb,
                  newdat= NULL,
                  type = "prob")[, "PD"], # using group of interest
            levels = levels(train1$ENROLL_CAT))) #.9996
auc(rocRFModb)
ci.auc(rocRFModb) #95% CI: 0.9989-1
head(predModRF)
thr<- .5
predModrf1<- factor(ifelse(predModRF[, "PD"] > thr, "PD", "HC"))
confusionMatrix(predModrf1, train1$ENROLL_CAT, positive = "PD")
# Sensitivity : 1.0000
# Specificity : 0.9341

coords(rocRFModb, x= "best", best.method = "youden")
# threshold specificity sensitivity
# 0.6190000   1.0000000   0.9903382
coords(rocRFModb, x= "best", best.method = "closest.topleft")# same


# Test/validation rf1
colnames(Natrf1[, -1])
colnames(test1[, c(5,15, 12,6, 7,2)])
# Best_ using nodesize = 3 and caret or native
predtest_rf_6b<- predict(rf6_nTb,
                  newdata= subset(test1,
                              select= c(5,15, 12,6, 7,2)), type = "prob")
vecRF1a2<- predtest_rf_6b[, "PD"]
roc.curve(test1$ENROLL_CAT,vecRF1a2)
# Area under the curve (AUC): 0.913

(rocRFMod6b2<-roc(test1$ENROLL_CAT,
            predict(rf6_nTb,
                  newdat= test1, select= c(5,15, 12,6, 7,2) ,
                  type = "prob")[, "PD"], # using group of interest
            levels = levels(test1$ENROLL_CAT)))
#Area under the curve: 0.9126

auc(rocRFMod6b2)
ci.auc(rocRFMod6b2) #95% CI: 0.858-0.9672 (DeLong)


thr<- .50
```

```
# converted to labels
predtestRF2<- factor(ifelse(predtest_rf_6b[, "PD"] > thr, "PD", "HC"))
head(predtestRF2)
confusionMatrix(predtestRF2, test1$ENROLL_CAT, positive="PD")#
# Reference
# Prediction HC PD
# HC 31  5
# PD  8 83

#Accuracy : 0.8976
#Kappa : 0.7542
# Sensitivity : 0.9432
# Specificity : 0.7949

coords(rocRFMod6b2, x = "best", best.method = "youden") #
# threshold specificity sensitivity
# 0.5341667   0.8717949   0.9090909

thr<-  0.5341667
# converted to labels
probRF2_thr<- factor(ifelse(predtest_rf_6b[, "PD"] >thr, "PD", "HC"))
head(probRF2_thr) # class labs
confusionMatrix(probRF2_thr, test1$ENROLL_CAT, positive = "PD")#
#Sensitivity : 0.9091
#Specificity : 0.8718

rocRF1_6<- plot.roc(test1$ENROLL_CAT,
            predict(rf6_nTb,
                  newdat= subset(test1, select= c(5,15, 12,6, 7,2)) ,
                  type = "prob")[, "PD"], # using group of interest
            levels = levels(test1$ENROLL_CAT),

            ci= TRUE, of = "thresholds", # CI for threshold
            thresholds = "best", #
            print.thres = "best",
            print.auc= TRUE,# highlight threshold in plot

            #print.thres.pattern = "%.3f (Spec = %.2f, Sens = %.2f)", # % special
character. .2f just print to 2 floating point decimal places
            print.thres.cex = .8,
            legacy.axes = TRUE,
            cex.axis= .6,
            cex.lab=.8)

dev.off()
```

```
# xgb_1
library(data.table)
library(xgboost)
library(e1071); library(doSNOW)
# doSNOW is for parallel computing and training;
# e1071  impromved predicted modeling

# See variables of importance (12) on lines 966 (native) and 1026 (caret)

# defaults first: not great result just AUC ~ .86
params1 <- list(booster = "gbtree", objective = "binary:logistic",
            eta=0.3, gamma=0,
            max_depth=6,
            min_child_weight=1,
            subsample=1,
            colsample_bytree=1)


# formating data for xgb
xgbTrain2<- data.table(train1[, c(1:6, 7, 10:15)], keep.rownames= F) # all in
data.table format
head(xgbTrain2)#
#write.csv(xgbTrain2, file= "xgbTrain2.csv")
is.factor(xgbTrain2$ENROLL_CAT)
xgbTrain2_Lab<- xgbTrain2$ENROLL_CAT
colnames(xgbTrain2)# ether tau lower mod AUC than CSF so CSF retained
head(xgbTrain2_Lab)
contrasts(xgbTrain2_Lab)
# PD
# HC  0
# PD  1
table(xgbTrain2_Lab)

# must put in matrix form: also required prior to sparse matrix creation for
# xgb.DMatrix (proprietary XBBoost format)

xgbTrain2Mat<- as.matrix(xgbTrain2[, -1]) # all as matrix except DV
#write(xgbTrain2Mat, file= "xgbTrain2Mat.csv")
str(xgbTrain2Mat)
head(xgbTrain2Mat)# dense matrix
# note in below subtracting 1 from alphabetically order contrasts
# which as.numeric are 1, 2
# subtracts 1 so first contrast = 0 and second = 1
head(xgbTrain2_Lab)
# HC PD HC PD HC PD
```

```
head(as.numeric(xgbTrain2_Lab))
# 1  2  1  2  1  2
xgbTrain2_LabNum<- as.numeric(xgbTrain2_Lab)-1
table(xgbTrain2_LabNum)
# 0   1
# 91 207

# test data
colnames(test1)
colnames(test1[, c(1:6, 7, 10:15)]) #
xgbTest2<- data.table(test1[, c(1:6, 7, 10:15)], keep.rownames= F) #
head(xgbTest2)
ncol(xgbTest2[, -1])#  12 OK
xgbTest2_lab<- xgbTest2$ENROLL_CAT
head(xgbTest2_lab)
table(xgbTest2_lab)
# HC PD
# 39 88
contrasts(xgbTest2_lab)
# PD
# HC  0
# PD  1
xgbTest2NumLab<- as.numeric(xgbTest2_lab)-1
is.vector(xgbTest2NumLab)
table(xgbTest2NumLab)
# 0  1
# 39 88

xgbTest2Mat<- as.matrix(xgbTest2[, -1]) # DV removed
colnames(xgbTest2Mat)

#Note Dense matrices store every entry in the matrix.
#Sparse matrices only store the nonzero entries.
#preparing DMatrix xgb.DMatrix constructed from either a dense matrix,
#or a sparse matrix,

dtrain2 <- xgb.DMatrix(data = xgbTrain2Mat,label = xgbTrain2_LabNum) #
print(attributes(dtrain2))
dtest2 <- xgb.DMatrix(data = xgbTest2Mat,label=xgbTest2NumLab)
print(attributes(dtest2))
# xgb1 native
set.seed(3450)
xgbcv2 <- xgb.cv( params1 = params1,
          data = dtrain2,
          nrounds = 500, # back to 100 default
          nfold = 10, showsd = T,
```

```
             stratified = T, print_every.n = 10,
             early_stop_round = 20, maximize = F, best_iteration = T,
             metrics = list("error", "auc"))

# at eta = .3
min(xgbcv2$evaluation_log$test_error_mean) #  0.1782853
max(xgbcv2$evaluation_log$test_auc_mean) #   0.8647737
xgbcv2$params$best_iteration# just says TRue

(found<-  0.8647737 %in% xgbcv2$evaluation_log$test_auc_mean) # TRUE

(ind<- which.max(xgbcv2$evaluation_log$test_auc_mean)) # 11 nrounds

set.seed(3450)
xgb1b <- xgb.train (params = params1, data = dtrain2,
              nrounds =11, watchlist = list(val=dtest2,train=dtrain2),
              print_every_n = 10, early_stop_round = 10, maximize = F ,
              eval_metric =  "auc")
xgb1b$evaluation_log$val_auc
# [1]     val-auc:0.867716    train-auc:0.940569
# [11]  val-auc:0.907051      train-auc:0.997877

#
# Highest AUC occured using 12 variables (all except GDS[depression], and ESS)
#[11]   val-auc:0.907051      train-auc:0.997877 # CSF asyn not pTau. tTau
mat2<- xgb.importance (feature_names = colnames(xgbTrain2Mat), model = xgb1b )
xgb.plot.importance(importance_matrix = mat2)
mat2
#      Feature   Gain       Cover  Frequency  Importance
# 1: RevUpsit.sum 0.543583071 0.292290099 0.13142857
# 2:      MCATOT 0.140359348 0.162598955 0.12571429
# 3:      rbdSum 0.053493884 0.103709012 0.10285714
# 4:    Abeta1_42 0.052262042 0.072165608 0.11428571
# 5:     SumTrait 0.048585893 0.063995237 0.09714286
# 6:         age 0.041651677 0.120618173 0.12571429
# 7:      CSFasyn 0.041447861 0.073930507 0.12571429
# 8:      EDUCYRS 0.030330728 0.023366820 0.05714286
# 9:      NP1CNST 0.019322081 0.024424834 0.03428571
# 10:      gdsSum 0.014678954 0.036234427 0.03428571
# 11:      epwSum 0.011480885 0.021731814 0.04000000
# 12:        gend 0.002803576 0.004934513 0.01142857
# caret tuning
tune_gridb <- expand.grid(eta = c(.075, .1, .2), # eta default=0.3; is the learning rate;
lower increments better
```

```
                    nrounds =c(50, 100, 500), # default = 100; similar to number of trees
or ntree in random forest; eta learning rate must be supported by nrounds; range
100-1000; will be tuned using CV as recommended
                    max_depth= c(4, 5, 6), # default=6: c(4:10) recommended; but 1:6 here
found good range; determines tree depth; larger tree with more depth has greater
chance of overfitting
                    min_child_weight = c(1, 2, 2.25), # default= 1, range: 0 inf; blocks the
potential feature interactions to prevent overfitting; if the leaf node has a minimum
sum of instance weight lower than min_child_weight, the tree splitting stops; should
be CV determined; In classification, if the leaf node has a minimum sum of instance
weight (calculated by second order partial derivative) lower than min_child_weight,
the tree splitting stops
                    colsample_bytree =c(.4,.6, .8), # c(.4,.6, .8) recommended but shorted
here; colsample_bytree[default=1][range: (0,1)]; control the number of features
(variables) supplied to a tree; Typically, its values lie between (0.5,0.9)
                    gamma= c(1, 5), # 0 is default and means no regularization;  higher
value penalizes large coefficiens that don't improve model preformacne
                    subsample = c(0.5, 0.75, 1))

metric <- "ROC"
ctrl.LGxg<- trainControl(
  method = "repeatedcv",
  number = 10,
  repeats = 5,
  #search = "random",
  classProbs = TRUE, # note class probabilities included
  savePredictions = "final",
  allowParallel = TRUE,
  summaryFunction = twoClassSummary)

library(doSNOW)
cl<-makeCluster(3, type= "SOCK") #  doSNOW will open 3 instances of R
registerDoSNOW(cl)

# may take ~ 15-20 minutes on cpu with 3 GHZ or greater processor and 4-cores
set.seed(3450)
xg_hcpdM1A<- train(x = xgbTrain2Mat,
          y = xgbTrain2_Lab, # caret requires factor
          method="xgbTree",
          metric = metric,
          tuneGrid = tune_gridb,
          verbose = TRUE,
          #preProcess =c("scale", "center"),
          trControl= ctrl.LGxg)

xg_hcpdM1A$bestTune$nrounds #500
```

```
xg_hcpdM1A$bestTune$max_depth #5
xg_hcpdM1A$bestTune$eta # 0.2
xg_hcpdM1A$bestTune$min_child_weight # 1#
xg_hcpdM1A$bestTune$colsample_bytree #.4 #
xg_hcpdM1A$bestTune$gamma #5
xg_hcpdM1A$bestTune$subsample #.5

stopCluster(cl)#
varImp(xg_hcpdM1A)
Overall
# RevUpsit.sum 100.000
# MCATOT       27.235
# Abeta1_42    15.246
# CSFasyn      13.431
# rbdSum       12.773
# age          9.983
# SumTrait     9.204
# NP1CNST       8.155
# EDUCYRS       5.971
# gdsSum       2.771
# gend         2.581
# epwSum        0.000

# Mod AUC xgb1
pred_hcpd_Mod2a<- predict(xg_hcpdM1A, newdata= NULL, type= "prob")
head(pred_hcpd_Mod2a)

(rocXg2_a<-roc(xgbTrain2_Lab,pred_hcpd_Mod2a[, "PD"],  #pred2xgb
          # using group of interest
          levels = levels(xgbTrain2_Lab)))
#Area under the curve: 0.9584
ci.auc(rocXg2_a)# 95% CI: 0.9374-0.9795 (DeLong)

#
thr<- .5
#converting to labels
predModxgb1<- factor(ifelse(pred_hcpd_Mod2a[, "PD"] > thr, "PD", "HC"))
head(predModxgb1)
confusionMatrix(predModxgb1, train1$ENROLL_CAT, positive= "PD")
#Sensitivity : 0.9372
#Specificity : 0.8352

coords(rocXg2_a, x= "best", best.method = "youden")
# threshold specificity sensitivity
# 0.6425525   0.9010989   0.8985507
```

```
# Validation/test xgb1
# test set  mod2a xgb_test
pred_xgb2a<- predict(xg_hcpdM1A, newdata= xgbTest2Mat ,
                 type= "prob")

# this is same, so XGBoost model does not need to be tested on matrix format
pred_xgb2a<- predict(xg_hcpdM1A, newdata= xgbTest2[,-1] , type= "prob")
head(pred_xgb2a)
range(pred_xgb2a)

thr<- .5
# converting to labels
pred_hcpd_carLab<- factor(ifelse(pred_xgb2a[, "PD"] > thr, "PD", "HC"))
head(pred_hcpd_carLab)
confusionMatrix(pred_hcpd_carLab, xgbTest2_lab, positive = "PD")
# Reference
# Prediction HC PD
# HC 31  9
# PD  8 79
# Accuracy : 0.8661
# Sensitivity : 0.8977
# Specificity : 0.7949


# ROC_ same as above, as expected
(roc1xgb<-roc(xgbTest2_lab,
            predict(xg_hcpdM1A,
                  newdat=xgbTest2Mat ,
                  type = "prob")[, "PD"], # using group of interest
            levels = levels(xgbTest2_lab)))
# Area under the curve: 0.9234

ci.auc(roc1xgb) #95% CI: 0.8747-0.9721 (DeLong)

#coords(roc1xgb, x = "best", best.method = "youden")
#  threshold specificity sensitivity
# 0.7221842   0.9230769   0.8522727

#coords(roc1xgb, x = "best", best.method = "closest.topleft")
# threshold specificity sensitivity
# 0.6598866   0.8974359   0.8750000

# best trade-off
thr<- 0.6598866#
pred_hcpdthr<- factor(ifelse(pred_xgb2a[, "PD"] > thr, "PD", "HC"))
```

```
head(pred_hcpdthr)
confusionMatrix(pred_hcpdthr, xgbTest2_lab, positive = "PD")
#Sensitivity : 0.8750
#Specificity : 0.8974

rocXGB1<- plot.roc(xgbTest2_lab,
          predict(xg_hcpdM1A,
               newdat=xgbTest2Mat ,
               type = "prob")[, "PD"], # using group of interest
          levels = levels(xgbTest2_lab),
          #ci= TRUE, # CI for threshold
          print.thres.best.method = "topleft", #
          print.thres = "best",
          print.auc= TRUE,
          legacy.axes =TRUE,# highlight threshold in plot
          #print.auc= TRUE,
          #print.thres.pattern = "%.3f (Spec = %.2f, Sens = %.2f)", # % special
character. .2f just print to 2 floating point decimal places
          print.thres.cex = .8,
          cex.axis= .6,
          cex.lab=.8)
dev.off()

# Comparitive tests for correlated AUC
# used_ rocRFMod6b2, rocXg2_test, rocLG2, rocTree1, rocGAM1
roc.test(rocGAM1, rocLG2, paired = T, method = "bootstrap", boot.stratified =
TRUE)# ns
roc.test(rocRFMod6b2, rocLG2, paired = T, method = "delong")# ns
roc.test(rocGAM1 , rocTree1, paired = T, method = "bootstrap") #p-value = 0.009
roc.test(rocXg2_test , rocTree1, paired = T, method = "bootstrap" ) #p-value = 0.004


## TESTING best two PD versus HC integrated models (GAM and Xgb); they are
#  applied to SWEDD versus HC test data
#
hcSWa<- read.csv("hcSWa_pd4SW_July9.csv", header= T)
colnames(hcSWa)
hcSWa$X<- NULL


table(hcSWa$ENROLL_CAT)
# HC SWEDD
# 39   43
table(hcSWa$ENROLL_CAT, hcSWa$gend)
#      0  1
# HC   13 26
```

```
# SWEDD 18 25

# catpd4SW SWEDD assigned to PD cat so model will
# recognize category BUT remember PD=SWEDD here and the SWEDD category
includes several clinial entities
table(hcSWa$catpd4sw)
# HC PD
# 39 43

colnames(hcSWa[, c(3, 6,9, 11,13,16)])
# [1] "age"         "RevUpsit.sum" "pTau"
# [4] "NP1CNST"      "rbdSum"       "MCATOT"
predswConGam1<- predict(modgam1d,
                  newdata = subset(hcSWa, select=c(3, 6,9, 11,13,16)) ,
                  type = "response")

# GAM early PD/control model applied to SWEDD/control validation set
head(predswConGam1)
contrasts(hcSWa$catpd4sw)

thr<- .5
predLab2<- factor(ifelse(predswConGam1 > thr, "PD", "HC"))
head(predLab2)
confusionMatrix(predLab2, hcSWa$catpd4sw, positive = "PD")

library(pROC)
(rocGAM3<-roc(hcSWa$catpd4sw,predswConGam1,
           levels = levels(hcSWa$catpd4sw)))# Area under the curve: 0.8634
ci.auc(rocGAM3) #95% CI: 0.7857-0.9412 (DeLong

# optimized coords
coords(rocGAM3, x = "best", best.method = "youden")
# threshold specificity sensitivity
# 0.3888635   0.8461538   0.8139535

thr<- 0.3888635
# converting to labels
predLab3<- factor(ifelse(predswConGam1 > thr, "PD", "HC"))
head(predLab2)
confusionMatrix(predLab3, hcSWa$catpd4sw, positive = "PD")
# Sensitivity : 0.8140
# Specificity : 0.8462

rocGAMplot<- plot.roc(hcSWa$catpd4sw,predswConGam1,
                levels = levels(hcSWa$catpd4sw),
                ci= TRUE, of = "thresholds", # CI for threshold
```

```
               thresholds = "best", #
               print.thres = "best",
               print.auc= TRUE,
               legacy.axes =TRUE,# highlight threshold in plot
               #print.auc= TRUE,
               #print.thres.pattern = "%.3f (Spec = %.2f, Sens = %.2f)", # % special
character. .2f just print to 2 floating point decimal places
               print.thres.cex = .8,
               cex.axis= .6,
               cex.lab=.8,
               main= "GAM")
dev.off()


#xgb_1 early PD/control model applied to SWEDD/control validation data
colnames(xgbTest2Mat)
# [1] "age"        "gend"        "EDUCYRS"      "RevUpsit.sum" "Abeta1_42"
# [6] "CSFasyn"      "NP1CNST"      "SumTrait"     "rbdSum"      "gdsSum"
# [11] "epwSum"      "MCATOT"

colnames(hcSWa[, c(3:8,11:16)])
# [1] "age"        "gend"        "EDUCYRS"      "RevUpsit.sum" "Abeta1_42"
# [6] "CSFasyn"      "NP1CNST"      "SumTrait"     "rbdSum"      "gdsSum"
#[11] "epwSum"      "MCATOT"
pred_xg1hcSW<- predict(xg_hcpdM1A, newdata= subset(hcSWa, select =
c(3:8,11:16)) , type= "prob")
head(pred_xg1hcSW)
predvecXGBhcSW<- pred_xg1hcSW[, "PD"] #PD= SWEDD here
head(predvecXGBhcSW)

(roc1onhcSW<-roc(hcSWa$catpd4sw,predvecXGBhcSW,
             levels = levels(hcSWa$catpd4sw)))  # Area under the curve: 0.8312
ci.auc(roc1onhcSW) #95% CI: 0.7418-0.9207 (DeLong)

#
thr<- .5
predxgbHcSWLab<- factor(ifelse(pred_xg1hcSW[, "PD"] >thr, "PD", "HC"))
confusionMatrix(predxgbHcSWLab, hcSWa$catpd4sw, positive = "PD") #

coords(roc1onhcSW, x= "best", method ="youden")
# threshold specificity sensitivity
# 0.3781936   0.7692308   0.8372093

thr<-  0.3781936
predxgbHcSWLabthr<- factor(ifelse(pred_xg1hcSW[, "PD"] >thr, "PD", "HC"))
confusionMatrix(predxgbHcSWLabthr, hcSWa$catpd4sw, positive = "PD")
```

```
# Sensitivity   0.8372
# Specificity : 0.7692

rocXGBhcSW<- plot.roc(hcSWa$catpd4sw,
                predict(xg_hcpdM1A, newdata =subset(hcSWa,
                                          select= c(3:8,11:16)),
                      type = "prob")[, "PD"],
                levels = levels(hcSWa$catpd4sw),
                ci= TRUE, of = "thresholds", # CI for threshold
                thresholds = "best", #
                print.thres = "best",
                print.auc= TRUE,
                legacy.axes =TRUE,# highlight threshold in plot
                main = "XGB",
                #print.thres.pattern = "%.3f (Spec = %.2f, Sens = %.2f)", # % special
character. .2f just print to 2 floating point decimal places
                print.thres.cex = .8,
                cex.axis= .6,
                cex.lab=.8)
dev.off()


# 2_pdsw SMOTE

pdsw2<- read.csv("pdsw2.csv", header = T)
pdsw2$X<-NULL

set.seed(9876)
indexPDSW1<- createDataPartition(y = pdsw2$ENROLL_CAT,
                        p = 0.50, # 50%
                        times = 1, #just 1 split
                        list = FALSE)
train50a <- pdsw2[indexPDSW1,]
test50a <- pdsw2[-indexPDSW1,]

table(train50a$ENROLL_CAT)
# PD SWEDD
# 148    22
table(test50a$ENROLL_CAT)
# PD SWEDD
# 147    21

# all similar
prop.table(table(pdsw2$ENROLL_CAT))
prop.table(table(train50a$ENROLL_CAT))
```

```
prop.table(table(test50a$ENROLL_CAT))

# glm_2

# balanced groups achieved by synthetic minority oversampling
sm1<-SMOTE(ENROLL_CAT ~., perc.over = 100, perc.under = 200,
        data = train50a[, -16])# removing PATNO
table(sm1$ENROLL_CAT)
# PD SWEDD
# 44    44

# saved smote data set (same as above)
Sm_dat<- read.csv("smote_pdswJune14.csv", header = T)
table(Sm_dat$ENROLL_CAT)# exactly even, as above
colnames(Sm_dat)# all except PATNO OK
Sm_dat$X<- NULL
table(Sm_dat$ENROLL_CAT)

# used for glm; includes transformed EDUCYRS var (which violated linearity of the
logit
# ); also includes recaled and standarized varsions of variables,
# see Supporting information V accompanying paper for details)

# includes dummy coded years of education variable (Quar25, Quar75, Quar_gp4)
# Note,the column named Ed_Dum is not needed (duplicate of col ED_Q). It can
# be removed, though then a new set of columns numbers designating (features)
# will need to be selected.

sm_dat_glmQ<- read.csv("sm_dat_glmQrsStd.csv", header = T)
sm_dat_glmQ$X<-NULL
testglm<- read.csv("testglmQdumPDSW.csv", header = T)
testglm$X<-NULL

# glm_2 features selected by stepwise logistic reg AIC; a process that
# also resulted in the features with highest with
# highest logistic model AUC (in the early PD/SWEDD classification)

#
colnames(glmDat<- Sm_dat[, c(1:4, 11,12, 15)])
# [1] "ENROLL_CAT"   "age"         "EDUCYRS"      "RevUpsit.sum" "rbdSum"
# [6] "gdsSum"       "gend"


#linearity of logit graph assessment and Box-tidwell test
# Fit the logistic regression model
summary(glm_sm1a<- glm(ENROLL_CAT~ RevUpsit.sum +
```

```
                     age + EDUCYRS + rbdSum +
                     gdsSum + gend,
                   data = Sm_dat,
                   family = binomial(link = 'logit')))
pr1<- predict(glm_sm1a, newdata =NULL,
            type = "response")

library(dplyr)
predClasses <- ifelse(pr1 > 0.5, "SWEDD", "PD")
head(predClasses)

library(dplyr)# only numeric so should remove DV
sm6dat<- glmDat[,-7] %>%
  dplyr::select_if(is.numeric)
head(sm6dat) # correct all but DV,  OK
preds1<- colnames(sm6dat)# get colnames
preds1
# Bind the logit, tidying and convert to long format
sm6dat<- sm6dat %>%
  mutate(logit = log(pr1/ (1-pr1))) %>%
  gather(key = "preds1", value= "predictor.value", -logit) # converts to long format;  -
logit » option will simply ignore the « logit » column when gathering the predictors.
head(sm6dat) # long format

ggplot(sm6dat, aes(logit, predictor.value))+
  geom_point(size = 0.5, alpha = 0.5) +
  geom_smooth(method = "loess") +
  theme_bw() +
  facet_wrap(~preds1, scales = "free_y")
# log odds on x axis; as go from high number of ED yrs to
# lower number, log odd odds of SWEDD increses
# age does not look appear to have linear relationship to logit

# Box-Tidwell
LLps<- glmDat
LLps$RevUpsitInt<- (LLps$RevUpsit.sum * log(LLps$RevUpsit.sum))
LLps$ageInt<- (LLps$age * log(LLps$age))
LLps$EDUCYRSInt<- (LLps$EDUCYRS * log(LLps$EDUCYRS))
LLps$rbdSumInt<- (LLps$rbdSum * log(LLps$rbdSum))
LLps$gdsSumInt<- (LLps$gdsSum * log(LLps$gdsSum))
LLps$gendInt<- (LLps$gend * log(LLps$gend))

summary(LLps_Mod1<- glm(ENROLL_CAT~RevUpsit.sum + RevUpsitInt + age +
ageInt+
                      rbdSum + rbdSumInt + EDUCYRS + EDUCYRSInt,
                    data= LLps, family = binomial))
```

```
# EDUCYRS      -6.80447    2.52420  -2.696  0.00702 **
# EDUCYRSInt    1.73301    0.65981   2.627  0.00863 **

#OK
summary(LLps_Mod2<- glm(ENROLL_CAT~RevUpsit.sum + RevUpsitInt + age +
ageInt+
                    rbdSum + rbdSumInt +gdsSum + gdsSumInt,
                   data= LLps, family = binomial))
# EDUCYRS violated lin of logit
# converted into quartiles (see Supporting information V for details)
# sames as using ED_Q;
# dummies vars Quar25( 25th quartile); Quar75
# 75th quartile; Quar_gp4 refers to 4th quartile; reference was the lowest quartile,
# those with <= 14 yrs education)
# of greatest yrs of education

set.seed(3450)
summary(glmA<- glm(ENROLL_CAT~ RevUpsit.sum +
                age + Quar25 + Quar75+ Quar_gp4 +  rbdSum +
                gdsSum + gend,
              data = sm_dat_glmQ,
              family = binomial(link = 'logit')))
# #            Estimate Std. Error z value Pr(>|z|)
# (Intercept)  -8.10015    3.42671  -2.364  0.01809 *
#   RevUpsit.sum -0.35627    0.09136  -3.899 9.64e-05 ***
#   age         0.12879    0.04108   3.135  0.00172 **
#   Quar25      -1.22455    1.26143  -0.971  0.33167
# Quar75      -2.90275    1.24951  -2.323  0.02017 *
#   Quar_gp4    -4.35679    1.47166  -2.960  0.00307 **
#   rbdSum       0.49130    0.16324   3.010  0.00261 **
#   gdsSum       0.63983    0.32389   1.975  0.04821 *
#   gend        2.54073    1.11280   2.283  0.02242 *
# Null deviance: 121.994  on 87  degrees of freedom
# Residual deviance:  54.091  on 79  degrees of freedom
# AIC: 72.091

# for diagnostic assumption tests see lines 1289-1373 PDSW.R

colnames(glm2dat<- sm_dat_glmQ[, c(1,2,4,11,12,15, 18:20)])
# write.csv(glm2dat, file= "glm2dat.csv")

# Model AUC glm_2
pred_modGLM<- predict(glmA, newdata =NULL,
                type = "response")
head(pred_modGLM)
```

```
library(pROC)
(modGLMpds<- roc(sm_dat_glmQ$ENROLL_CAT,pred_modGLM )) #Area under the
curve: 0.938
(modGLMpds<- roc(sm_dat_glmQ$ENROLL_CAT,
            predict(glmA, newdata = NULL,
                  type = "response"),
            levels = levels(sm_dat_glmQ$ENROLL_CAT))) #same
ci.auc(modGLMpds)# 95% CI: 0.8627-0.972 (DeLong)
#
thr<- .5
# convert to labels
predglmMod2<- factor(ifelse(pred_modGLM > .5, "SWEDD", "PD"))
head(predglmMod2)
confusionMatrix(predglmMod2, sm_dat_glmQ$ENROLL_CAT, positive = "SWEDD")
# Sensitivity : 0.8636
# Specificity : 0.8409

coords(modGLMpds, x= "best", best.method= "closest.topleft")
#coords(modGLMpds, x= "best", best.method= "youden")# same

# threshold specificity sensitivity
#0.4511014   0.8409091   0.9090909

thr<- 0.4511014
# converting to labels
predglmMod2b<- factor(ifelse(pred_modGLM > thr, "SWEDD", "PD"))
head(predglmMod2b)
confusionMatrix(predglmMod2b, sm_dat_glmQ$ENROLL_CAT, positive = "SWEDD")
# Sensitivity : 0.9091
# Specificity : 0.8409

# glm_2 validation/test
colnames(sm_dat_glmQ[, c(2,4, 18:20,11,12,15)])
colnames(testglm[, c(2,4,19:21,11,12,15 )])

pred_glmSm1a<- predict(glmA, newdata = subset(testglm, select=
c(2,4,19:21,11,12,15)),
                type = "response")
head(pred_glmSm1a)
contrasts(sm_dat_glmQ$ENROLL_CAT)

library(pROC)
rocGLM_2<- roc(testglm$ENROLL_CAT,pred_glmSm1a )
#Area under the curve: 0.7787
#not this model glm_sm1a without ED in quatiles a .80 AUC
```

```
ci.auc(rocGLM_2)# 95% CI: 0.6772-0.8803 (DeLong)
auc(rocGLM_2) #Area under the curve: 0.7787

(rocGLM_2<- roc(testglm$ENROLL_CAT,
          predict(glmA, newdata = subset(testglm, select= c(2,4,19:21,11,12,15)),
               type = "response"),
          levels = levels(testglm$ENROLL_CAT))) #same OK

roc.curve(testglm$ENROLL_CAT, pred_glmSm1a )# .779 same OK

thr<- 0.5 #
predlabglm2<- factor(ifelse(pred_glmSm1a > thr, "SWEDD", "PD"))
head(predlabglm2)
confusionMatrix(predlabglm2, testglm$ENROLL_CAT, positive = "SWEDD")

coords(rocGLM_2, x = "best", best.method = "youden")
# threshold specificity sensitivity
# 0.5037343   0.7551020   0.6666667

thr<-  0.5037343  #lowering thrs (e.g. .40) will improve sens at cost of lower spec
#converting to labels
predbestLR2<- factor(ifelse(pred_glmSm1a > thr, "SWEDD", "PD"))
head(predbestLR2)
confusionMatrix(predbestLR2, testglm$ENROLL_CAT, positive = "SWEDD")
# Sensitivity : 0.66667
# Specificity : 0.75510

rocLG_2<- plot.roc(testglm$ENROLL_CAT,pred_glmSm1a,
            levels = levels(testglm$ENROLL_CAT),
            ci= TRUE, of = "thresholds", # CI for threshold
            thresholds = "best", #
            print.thres = "best",
            print.auc= TRUE,
            legacy.axes =TRUE,# highlight threshold in plot
            #print.auc= TRUE,
            #print.thres.pattern = "%.3f (Spec = %.2f, Sens = %.2f)", # % special
character. .2f just print to 2 floating point decimal places
            print.thres.cex = .8,
            cex.axis= .6,
            cex.lab=.8,
            main = "GLM")
dev.off()

#GAM_2

library(nlme)
```

```
library(mgcv)

# same features as in GLM
set.seed(3450)
modGam_pds1<- gam(ENROLL_CAT~ age + RevUpsit.sum +
                  rbdSum + s(EDUCYRS, bs="tp", k=7) +
                  gend + gdsSum,
                 data= sm_dat_glmQ,
                 method = "REML",
                 family= binomial(link= 'logit'))
#
summary(modGam_pds1)
# Parametric coefficients:
#   Estimate Std. Error z value Pr(>|z|)
# (Intercept)  -10.20406    3.43193  -2.973 0.002946 **
#   age           0.13050    0.04095   3.187 0.001438 **
#   RevUpsit.sum  -0.40482    0.09974  -4.059 4.94e-05 ***
#   rbdSum        0.57431    0.16794   3.420 0.000627 ***
#   gend          2.63400    1.13550   2.320 0.020358 *
#   gdsSum        0.59875    0.31026   1.930 0.053623 .

#
# Approximate significance of smooth terms:
#   edf Ref.df Chi.sq p-value
# s(EDUCYRS) 4.119  4.912  14.39  0.0126 *
gam.check(modGam_pds1)
# k'  edf k-index p-value
#s(EDUCYRS) 6.00 4.12    0.74    0.01 **
# Low p-value (k-index<1) may
# but k not close to edf so looks OK

anova(glmA, modGam_pds1, test = "Chisq" )
# Resid. Df Resid. Dev     Df Deviance Pr(>Chi)
# 1    79.000     54.091
# 2    77.881     46.043 1.1187   8.0482 0.005576 **

# Mod AUC GAM2
predgamMod2<- predict.gam(modGam_pds1, type= "response")
head(predgamMod2)

(rocGAM2_mod2<-roc(sm_dat_glmQ$ENROLL_CAT,predgamMod2,
                   levels = levels(sm_dat_glmQ$ENROLL_CAT)))
#Area under the curve: 0.9551
#
ci.auc(rocGAM2_mod2) #95% CI: 0.916-0.9941 (DeLong)
auc(rocGAM2_mod2) #Area under the curve: 0.9551
```

```
library(ROSE)
roc.curve(sm_dat_glmQ$ENROLL_CAT,predgamMod2) #same OK


thr<-.5
# converting to labels
predModGam2<- factor(ifelse(predgamMod2 > thr, "SWEDD", "PD"))
confusionMatrix(predModGam2, Sm_dat$ENROLL_CAT, positive = "SWEDD")
# Sensitivity : 0.8864
# Specificity : 0.9091

#GAM2_ validation/test
colnames(pdsw2[, c(2,3,4,11,12,15)])
colnames(sm_dat_glmQ[, c(2,3,4,11,12,15)]])
colnames(testglm)
colnames(testglm[, c( 2:4,11,12,15 )]])

predgam2<- predict.gam(modGam_pds1,
                newdata= subset(testglm, select=c( 2,3,4,11,12,15)),
                type ="response")
head(predgam2)

thr<-.5
# converting to labels
predlabGam2<- factor(ifelse(predgam2 > thr, "SWEDD", "PD"))
head(predlabGam2)
confusionMatrix(predlabGam2, testglm$ENROLL_CAT, positive = "SWEDD")


library(pROC)
(rocGAM2<-roc(testglm$ENROLL_CAT,predgam2,
         levels = levels(testglm$ENROLL_CAT)))
#Area under the curve: 0.7872


#
library(ROSE)
roc.curve(testglm$ENROLL_CAT,predgam2 )#same

auc(rocGAM2)#
ci.auc(rocGAM2)#  95% CI: 0.6887-0.8856 (DeLong)

coords(rocGAM2, x = "best", best.method = "youden")
# threshold specificity sensitivity
#  0.4373964   0.7619048   0.7142857
```

```
#coords(rocGAM1, x = "best", best.method = "topleft")# same

thr<-0.4373964
predlabGam1<- factor(ifelse(predgam2 > thr, "SWEDD", "PD"))
head(predlabGam1)
confusionMatrix(predlabGam1, testglm$ENROLL_CAT, positive = "SWEDD") #
# Sensitivity : 0.71429
# Specificity : 0.76190

rocLG_2<- plot.roc(testglm$ENROLL_CAT,predgam2,
            levels = levels(testglm$ENROLL_CAT),
            ci= TRUE, of = "thresholds", # CI for threshold
            thresholds = "best", #
            print.thres = "best",
            print.auc= TRUE,
            legacy.axes =TRUE,# highlight threshold in plot
            #print.auc= TRUE,
            #print.thres.pattern = "%.3f (Spec = %.2f, Sens = %.2f)", # % special
character. .2f just print to 2 floating point decimal places
            print.thres.cex = .8,
            cex.axis= .6,
            cex.lab=.8,
            main = "GAM")
dev.off()


#tree2

# features
dat_treeSmote<- read.csv("TreeSub_resampSm88_66.csv", header=T)
dat_treeSmote$X<-NULL
table(dat_treeSmote$ENROLL_CAT)
# PD SWEDD
# 88    66

# Note this SMOTE decsion tree data set was left at the default setting
# from caret resampling; subampling within resampled SMOTE data was ONLY
# availabel for decision tree but not other tree model types, and it resulted
# in a higher AUC than SMOTE outside of caret resampling

# Features had highest AUC
colnames(tree2dat<- dat_treeSmote[, c(1:4,7,9 ,10,11,12,13,15) ])
# [1] "age"         "EDUCYRS"      "RevUpsit.sum" "Abeta1_42"
# [5] "tTau"        "SumTrait"     "rbdSum"       "gdsSum"
# [9] "epwSum"      "MCATOT"       "ENROLL_CAT"
#write.csv(tree2dat, file= "tree2dat.csv")
```

```
ncol(tree2dat[, -11])
# native tree
set.seed(3450)
tree2<- rpart(ENROLL_CAT~.,  data = tree2dat,
          control = rpart.control(minbucket = 3, cp=.001,
                            maxdepth = 5), method = "class")
tree2$cptable
min.xerror <- tree2$cptable[which.min(tree2$cptable[,"xerror"]),"CP"]
min.xerror # 0.01515152

metric <- "ROC"
controltree <- trainControl(method="repeatedcv",
                    number=10,
                    repeats=5,
                    classProbs = TRUE,
                    savePredictions = "final",
                    allowParallel = TRUE,
                    #search="random", # with logistic and random forest control
(includes search = random)
                    summaryFunction = twoClassSummary)

set.seed(3450)
treeSmote10<- train(ENROLL_CAT ~.,
             data = dat_treeSmote[, c(1:4,7,9 ,10,11,12,13,15) ], #
             method="rpart",
             metric = metric,
             tuneLength = 30,
             #preProcess =c("scale", "center"), #"center"
             trControl= controltree)
treeSmote10
# cp          ROC       Sens      Spec
#
#0.008881923  0.8318618  0.8375000  0.7319048
round(treeSmote10$finalModel$variable.importance,2)
# RevUpsit.sum      epwSum      MCATOT       tTau        age      rbdSum
# 24.64             16.20       10.53       10.52       9.67       9.03
# Abeta1_42      EDUCYRS      gdsSum    SumTrait
# 7.22          3.10        2.71        1.83

varImp(treeSmote10)
# RevUpsit.sum 100.000
# epwSum       89.692
# age         87.443
# EDUCYRS      79.757
# tTau        56.777
```

```
# Abeta1_42    51.197
# MCATOT       28.988
# SumTrait      5.590
# rbdSum        3.807
# gdsSum        0.000

treeSmote10$finalModel$call$control$minbucket #7
treeSmote10$finalModel$call$control$minsplit #20
treeSmote10$finalModel$call$control$maxdepth #30

# Mod tree2 AUC

predmodTree2<- predict(treeSmote10, newdata = NULL, type= "prob")

head(predmodTree2)
tail(predmodTree2)

vecTree<- predmodTree2[, "SWEDD"]
1- 0.19512195 #0.8048781 OK

head(vecTree)

library(pROC)
(rocTrModps<-roc(dat_treeSmote$ENROLL_CAT,
            predict(treeSmote10, newdata = NULL,
                  type = "prob")[, "SWEDD"], # using group of interest
            levels = levels(dat_treeSmote$ENROLL_CAT))) #Area under the curve:
0.9324
roc(dat_treeSmote$ENROLL_CAT, vecTree) #Area under the curve: 0.9324

ci.auc(rocTrModps) #95% CI: 0.8936-0.9712 (DeLong)

library(ROSE)
roc.curve(dat_treeSmote$ENROLL_CAT, predmodTree2[, "SWEDD"])# same

thr <- .5
# converting to labels
predModLabTree2<- factor(ifelse(predmodTree2[, "SWEDD"] > thr, "SWEDD",
"PD"))
head(predTrLab)
confusionMatrix(dat_treeSmote$ENROLL_CAT, predModLabTree2, positive =
"SWEDD")
# Sensitivity : 0.8906
# Specificity : 0.9000

# optimized coords
```

```
coords(rocTrModps, x = "best", best.method = "youden")
# threshold specificity sensitivity
#0.4864499   0.9204545   0.8636364

thr<- 0.4864499
predLabTR2<- factor(ifelse(predmodTree2[, "SWEDD"] > thr, "SWEDD", "PD"))

confusionMatrix(predLabTR2,dat_treeSmote$ENROLL_CAT, positive = "SWEDD" )
# Sensitivity : 0.8636
# Specificity : 0.9205

# tree_2 validation/test
colnames(dat_treeSmote[, c(1:4,7,9 ,10,11,12,13) ])
colnames(test50a)
colnames(test50a[, c(2, 3,4,5,8,10,11,12,13,14)])
predTr_swpd<- predict(treeSmote10$finalModel, newdata = subset(test50a,
                select= c(2, 3,4,5,8,10,11,12,13,14)), type= "prob")
head(predTr_swpd)
tail(predTr_swpd)

vecTr_pdsw<- predTr_swpd[, "SWEDD"]

(rocTr_psdw<-roc(test50a$ENROLL_CAT,
            predict(treeSmote10$finalModel, newdata =subset(test50a,
                                            select= c(2, 3,4,5,8,10,11,12,13,14)),
                  type = "prob")[, "SWEDD"], # using group of interest
            levels = levels(test50a$ENROLL_CAT))) #Area under the curve: 0.7428

roc(test50a$ENROLL_CAT, vecTr_pdsw) # same

roc.curve(test50a$ENROLL_CAT, vecTr_pdsw) # same

ci.auc(rocTr_psdw) #95% CI: 0.617-0.8686 (DeLong)

# confusion matrix
thr <- .5
# converting to labels
predTrLab<- factor(ifelse(predTr_swpd[, "SWEDD"] > thr, "SWEDD", "PD"))
head(predTrLab)
confusionMatrix(test50a$ENROLL_CAT, predTrLab, positive = "SWEDD")

# optimized coords
coords(rocTr_psdw, x = "best", best.method = "youden")
# # threshold specificity sensitivity
# 0.4864499   0.8163265   0.6666667
```

```
thr<- 0.4864499
predLabTR2<- factor(ifelse(predTr_swpd[, "SWEDD"] > thr, "SWEDD", "PD"))

confusionMatrix(predLabTR2,test50a$ENROLL_CAT, positive = "SWEDD" )
# Sensitivity : 0.66667
# Specificity : 0.81633


rocTR_pdswSM<- plot.roc(test50a$ENROLL_CAT,
            predict(treeSmote10$finalModel, newdata =subset(test50a,
                                    select= c(2, 3,4,5,8,10,11,12,13,14)),
               type = "prob")[, "SWEDD"],
            levels = levels(test50a$ENROLL_CAT),
            ci= TRUE, of = "thresholds", # CI for threshold
            thresholds = "best", #
            print.thres = "best",
            print.auc= TRUE,
            legacy.axes =TRUE,# highlight threshold in plot
            #print.auc= TRUE,
            #print.thres.pattern = "%.3f (Spec = %.2f, Sens = %.2f)", # % special
character. .2f just print to 2 floating point decimal places
            print.thres.cex = .8,
            cex.axis= .6,
            cex.lab=.8)
dev.off()


# rf_2 rf_pdsw

#rf_2 features
# selected by Rf importance and
# features that produced mod with highest AUC

library(caret)
metric <- "ROC"
ctrl.tb<- trainControl(
  method = "repeatedcv",
  number = 10,
  repeats = 5,
  search = "random",
  classProbs = TRUE, # note class probabilities included
  savePredictions = "final",
  allowParallel = TRUE,
  summaryFunction = twoClassSummary)

colnames(rf2<-Sm_dat[, -c(7,8)])
```

```
# removed pTau and tTau;
# feature elimination determined Alpha-asyn more important

#write.csv(rf2, file= "rf2.csv")

set.seed(3450)
rf_Smote4<- train(ENROLL_CAT ~.,
            data = Sm_dat[, -c(7,8)], # removing taus but retaining CSFasyn
            method="rf",
            metric = metric,
            ntree= 3000,
            nodesize = 1,
            importance = T,
            trControl= ctrl.tb)
rf_Smote4
rf2cols<- rf_Smote4$finalModel$importance
#                   PD     SWEDD MeanDecreaseAccuracy MeanDecreaseGini
# age          0.023359472 0.02460689          0.02407231        3.6107806
# EDUCYRS      0.020135649 0.02610213          0.02286455        3.3651036
# RevUpsit.sum 0.045655719 0.05873869          0.05177872        5.2682377
# Abeta1_42    0.016901167 0.01983513          0.01839591        3.5958043
# CSFasyn      0.006625528 0.02238511          0.01446508        3.1940778
# NP1CNST      0.013780788 0.01523357          0.01443794        1.6038831
# SumTrait     0.027917125 0.02016454          0.02394299        3.5478183
# rbdSum       0.028805035 0.03456324          0.03148131        3.8887961
# gdsSum       0.015284721 0.01379646          0.01435858        2.7571704
# epwSum       0.031455506 0.04154790          0.03627434        4.4061258
# MCATOT       0.019662801 0.02410897          0.02176893        3.1023135
# gend         0.005080654 0.01595500          0.01038553        0.9581928


varImp(rf_Smote4)
# RevUpsit.sum   100.000
# epwSum          74.156
# rbdSum          60.377
# age             45.280
# SumTrait        44.161
# MCATOT          39.461
# EDUCYRS         35.193
# NP1CNST         30.023
# Abeta1_42       20.431
# gdsSum          19.533
# CSFasyn          9.591
# gend             0.000

# Mod AUC rf_2
```

```
predModRF_pds<- predict(rf_Smote4, newdata = NULL, type = "prob")
head(predModRF_pds)
vecRF2<- predModRF_pds[, "SWEDD"]
length(vecRF2)

library(pROC)
rocRF2mod<- roc(Sm_dat$ENROLL_CAT, predict(rf_Smote4, newdata= NULL,
                                type = "prob")[, "SWEDD"],
            levels = levels(Sm_dat$ENROLL_CAT)) #
ci.auc(rocRF2mod) #95% CI: 1-1 (DeLong)
auc(rocRF2mod) #Area under the curve:1

thr<- .5
rfModSMlab<- factor(ifelse(predModRF_pds[, "SWEDD"] > thr, "SWEDD", "PD"))
confusionMatrix(rfModSMlab, Sm_dat$ENROLL_CAT, positive= "SWEDD")

# Sensitivity :1
# Specificity :1

# prediction on validation set test50a
colnames(rfsm4dat<- Sm_dat[, -c(1,7,8)]) # all but taus
colnames(testRF2set<- test50a[, -c( 1, 7,8,16)])
ncol(testRF2set<- test50a[, -c( 1, 7,8,16)])#12

#rf_2 Validation/test
predRF_sm4<- predict(rf_Smote4, newdata = testRF2set,
               type = "prob")
head(predRF_sm4)
#      PD    SWEDD
# 1 0.7293333 0.2706667
# 3 0.4750000 0.5250000
# 4 0.4190000 0.5810000
# 5 0.5450000 0.4550000
# 6 0.5560000 0.4440000
# 7 0.5286667 0.4713333
head(VecpreRFsw<- predRF_sm4[, "SWEDD"])
#0.2706667 0.5250000 0.5810000 0.4550000 0.4440000 0.4713333
mean(VecpreRFsw) #0.4134147
head(VecpreRFpd<- predRF_sm4[, "PD"])
mean(VecpreRFpd)# ] 0.5865853

#0.7293333 0.4750000 0.4190000 0.5450000 0.5560000 0.5286667
VecpreRF[1:10]
tail(predRF_sm4)
#       PD    SWEDD
# 327 0.7993333 0.2006667
```

```
# 329 0.6930000 0.3070000
# 330 0.7716667 0.2283333
# 332 0.4830000 0.5170000
# 334 0.5916667 0.4083333
# 336 0.5670000 0.4330000
tail(VecpreRF) #0.2006667 0.3070000 0.2283333 0.5170000 0.4083333
0.4330000


thr<- .5
#converted to labels
pred_pdswSMlabs<- factor(ifelse(predRF_sm4[, "SWEDD"] > thr, "SWEDD", "PD"))
confusionMatrix(pred_pdswSMlabs, test50a$ENROLL_CAT, positive = "SWEDD")

library(pROC)#
roc_rf_SM<-roc(test50a$ENROLL_CAT,
          predict(rf_Smote4, newdata = testRF2set,
                type= "prob")[, "SWEDD"],
          levels= levels(test50a$ENROLL_CAT)) #
auc(roc_rf_SM)# Area under the curve: 0.8225
ci.auc(roc_rf_SM) #95% CI: 0.746-0.8989 (DeLong)

# coords(roc_rf_SM, x = "best",input="threshold", best.method = "youden")
# threshold specificity sensitivity
# 0.4360000   0.6598639   0.9047619
coords(roc_rf_SM, x = "best",input="threshold", best.method = "topleft")# more
balanced here than Youden
# threshold specificity sensitivity
# 0.4606667   0.7210884   0.8095238

thr<-   0.4606667 #  0.4360000 #  again selecting "SWEDD" if thr for SWEDD > .461
else get PD
pred_pdswSMlabs_th<- factor(ifelse(predRF_sm4[,"SWEDD"]> thr, "SWEDD", "PD"))
confusionMatrix(pred_pdswSMlabs_th, test50a$ENROLL_CAT, positive = "SWEDD")
# Sensitivity : 0.8095
# Specificity : 0.7211

rocbjRF<- plot.roc(test50a$ENROLL_CAT,
            predict(rf_Smote4, newdata = testRF2set,
                  type= "prob")[, "SWEDD"],
            levels = levels(test50a$ENROLL_CAT),
            print.thres = "best",
            print.thres.best.method = "topleft",
            print.auc= TRUE,
            legacy.axes =TRUE,
            print.thres.cex = .8,
```

```
        cex.axis= .6,
        cex.lab=.8)
plot(ci(roc_rf_SM, of= "thresholds", thresholds = "best", best.method="topleft"))
dev.off()

## xgb_2
# xgboost_pdsw

library(data.table)
library(xgboost)
library(e1071); library(doSNOW) #

# data.table and matrix
library(data.table)
xgtrSM<- data.table(Sm_dat, keep.rownames= F) # all in data.table format
#write.csv(xgtrSM, file= "xgtrSM.csv")
head(xgtrSM)#
str(xgtrSM) # data.table
is.factor(xgtrSM$ENROLL_CAT)
xgtrSM_Lab<- xgtrSM$ENROLL_CAT  # extracting DV labels
head(xgtrSM_Lab)
contrasts(xgtrSM_Lab)
# SWEDD
# PD      0
# SWEDD    1
table(xgtrSM_Lab)
#OK
# PD SWEDD
# 44    44

# must put in matrix before converting to xgb.DMatrix, which is sparse
# will will not be used  immediately
xgtrSM_Mat<- as.matrix(xgtrSM[, -1]) # all as (dense) matrix except DV
head(xgtrSM_Mat)
str(xgtrSM_Mat)
# note in below subtracting 1 from alphabetically order contrasts
# which as.numeric are 1, 2
# subtracts 1 so firt contrast = 0 and second = 1
head(xgbtr3a_Lab)
#[1] PD PD PD PD PD PD
#Levels: PD SWEDD
head(as.numeric(xgtrSM_Lab))
tail(as.numeric(xgtrSM_Lab))
table(as.numeric(xgtrSM_Lab))
# 1  2
# 44 44
```

```
xgtrSM_LabNum<- as.numeric(xgtrSM_Lab)-1
table(xgtrSM_LabNum) #OK correct
# 0  1
# 44 44

# test data
colnames(test50a)
xgtest3a<- test50a[, -16]  #less PATNO not in model; PATNO can be returned post
analysis
xgtest3a<- data.table(xgtest3a, keep.rownames= F) # all in data.table formate
head(xgtest3a)#
str(xgtest3a) # data.table
is.factor(xgtest3a$ENROLL_CAT)
xgtest3a_Lab<- xgtest3a$ENROLL_CAT  # extracting DV labels
head(xgtest3a_Lab)
contrasts(xgtest3a_Lab)
# SWEDD
# PD       0
# SWEDD    1
table(xgtest3a_Lab)
#xgtest3a_Lab
# PD SWEDD
# 147   21
# must put in matrix before converting to xgb.DMatrix, which is sparse
# will will not be used  immediately
xgtest3aMat<- as.matrix(xgtest3a[, -1]) # all as matrix except DV
head(xgtest3aMat)
colnames(xgtest3aMat)
# note in below subtracting 1 from alphabetically order contrasts
# which as.numeric are 1, 2
# subtracts 1 so firt contrast = 0 and second = 1
head(xgtest3a_Lab)
#[1] PD PD PD PD PD PD
#Levels: PD SWEDD
head(as.numeric(xgtest3a_Lab))
table(as.numeric(xgtest3a_Lab))
# 1   2
# 147  21 #
xgtest3a_LabNum<- as.numeric(xgtest3a_Lab)-1
table(xgtest3a_LabNum) #OK correct
#   0   1
# 148  21 OK

# defaults
params1 <- list(booster = "gbtree", objective = "binary:logistic",
             eta=0.3, gamma=0,
```

```
            max_depth=6,
            min_child_weight=1,
            subsample=1,
            colsample_bytree=1)

# XGBoost native first
# Note taus should be removed for final model set of features;
# at least if you follow the rather sensitive maxcorrelation level set here
# of .75; taus are highly correlated (rs = 97), and taus are quite highly correlated
# with Alpha-syn
# rs= .83)
dtrain_xgb2<- xgb.DMatrix(data = xgtrSM_Mat,label = xgtrSM_LabNum) # Lab must
be numeric

agMatb<-xgtrSM_Mat[, -c(6,7)]# removes taus
dtrain_xgb2b<- xgb.DMatrix(data = agMatb,label = xgtrSM_LabNum) # Lab must be
numeric
dtest_xgb2<- xgb.DMatrix(data = xgtest3aMat,label=xgtest3a_LabNum)#


set.seed(3450)#
xgbcv_2b<- xgb.cv( params = params1,# new params1 from caret subsampling
             data = dtrain_xgb2b,# all features
             nrounds = 500, # 100 default
             nfold = 10, showsd = T,
             stratified = T, print_every.n = 10,
             early_stop_round = 20, maximize = F, best_iteration = T,
             metrics =  list("error" , "auc"))
# at eta = .3
min(xgbcv_2b$evaluation_log$test_error_mean) # 0.14375
max(xgbcv_2b$evaluation_log$test_auc_mean) #   0.9602183
xgbcv_2b$params$best_iteration#

(found<-   0.9602183 %in% xgbcv_2b$evaluation_log$test_auc_mean) # TRUE

(ind<- which.max(xgbcv_2b$evaluation_log$test_auc_mean)) #309

set.seed(3450) # must use xgb.train to get xgb.importance BUT all so can not use
xgb_2b<- xgb.train (params = params1, data = dtrain_xgb2b,
              nrounds =309, watchlist = list(val=dtest_xgb2,train=dtrain_xgb2b),
              print_every_n = 10, early_stop_round = 10, maximize = F ,
              eval_metric =  "auc") #val-auc:0.695821 low without caret resampling
matxgb2<- xgb.importance (feature_names = colnames(dtrain_xgb2b), model =
xgb_2b )
xgb.plot.importance(importance_matrix =matxgb2)
matxgb2 #
```

```
#        Feature       Gain      Cover  Frequency
# 1: RevUpsit.sum 0.207053387 0.15055582 0.10344828
# 2:       epwSum 0.160236556 0.16217506 0.13793103
# 3:      EDUCYRS 0.139566835 0.10828640 0.10114943
# 4:      CSFasyn 0.101668786 0.10795016 0.14022989
# 5:          age 0.100248637 0.08847830 0.09425287
# 6:     SumTrait 0.082899161 0.04305370 0.04827586
# 7:       rbdSum 0.066671673 0.06512179 0.07356322
# 8:       MCATOT 0.064022396 0.08238519 0.05977011
# 9:    Abeta1_42 0.049285911 0.11158518 0.13563218
# 10:      gdsSum 0.021665061 0.06365214 0.08735632
# 11:        gend 0.006681598 0.01675626 0.01839080

# BUT using caret (k-fold) resampling, the highest AUC was obtained with Alpha-
syn, constipation and gender removed
library(caret)
metric <- "ROC"
ctrl.LGxg<- trainControl(
  method = "repeatedcv",
  number = 10,
  repeats = 5,
  #search = "random",
  classProbs = TRUE, # note class probabilities included
  savePredictions = "final",
  allowParallel = TRUE,
  summaryFunction = twoClassSummary)

tune_gridb3 <- expand.grid(eta = c(.075, .1, .2), # eta default=0.3; is the learning
rate;  lower increments better
                      nrounds =c(50, 100, 500), # default = 100; similar to number of trees
or ntree in random forest; eta learning rate must be supported by nrounds; range
100-1000; will be tuned using CV as recommended
                      max_depth= c(4, 5, 6), # default=6: c(4:10) recommended; but 1:6
here found good range; determines tree depth; larger tree with more depth has
greater chance of overfitting
                      min_child_weight = c(1, 2, 2.25), # default= 1, range: 0 inf; blocks the
potential feature interactions to prevent overfitting; if the leaf node has a minimum
sum of instance weight lower than min_child_weight, the tree splitting stops; should
be CV determined; In classification, if the leaf node has a minimum sum of instance
weight (calculated by second order partial derivative) lower than min_child_weight,
the tree splitting stops
                      colsample_bytree =c(.4,.6, .8), # c(.4,.6, .8) recommended but shorted
here; colsample_bytree[default=1][range: (0,1)]; control the number of features
(variables) supplied to a tree; Typically, its values lie between (0.5,0.9)
                      gamma= 3, # 0 is default and means no regularization;  higher value
penalizes large coefficiens that don't improve model preformacne
```

```
                    subsample = c(0.5, 0.75, 1)) # number of observations supplied to a
tree

# features resulting in highest AUC from resampling (k-fold: 10 folds * 5 repeats)
colnames(xgtrSM_Mat[, -c(5:7, 14)])
#  "age"        "EDUCYRS"     "RevUpsit.sum"
# "Abeta1_42"   "NP1CNST"     "SumTrait"
# "rbdSum"      "gdsSum"      "epwSum"
# "MCATOT"


#original without gender, taus and csf, had highest AUC
library(doSNOW)
cl<-makeCluster(3, type= "SOCK") # tells doSNOW to open 3 instances of R to make
registerDoSNOW(cl)
set.seed(3450)#  with gamma as 3 to reduce overfitting
xgbsmPDSW_4<- train(x = xgtrSM_Mat[, -c(5:7, 14)], #
              y = xgtrSM_Lab, # caret requires factor
              method="xgbTree",
              metric = metric,
              tuneGrid = tune_gridb3,  # gamma changed from 1 t 3
              verbose = TRUE,
              #preProcess =c("scale", "center"),
              trControl= ctrl.LGxg)

xgbsmPDSW_4$bestTune$nrounds #500
xgbsmPDSW_4$bestTune$max_depth #4
xgbsmPDSW_4$bestTune$eta # 0.2
xgbsmPDSW_4$bestTune$min_child_weight # 1#
xgbsmPDSW_4$bestTune$colsample_bytree #0.8
xgbsmPDSW_4$bestTune$gamma #3
xgbsmPDSW_4$bestTune$subsample #.75
# #
varImp(xgbsmPDSW_4)
# Overall
# RevUpsit.sum  100.00
# epwSum        95.35
# EDUCYRS       63.75
# MCATOT        42.37
# rbdSum        37.98
# Abeta1_42     28.08
# age           23.71
# gdsSum        12.57
# SumTrait      12.23
# NP1CNST        0.00
```

```
# Mod AUC xgb2
predModxgps4<- predict(xgbsmPDSW_4, newdata = NULL, type = "prob")
# head(predModxgps4)
#
vecXGBmod4<- predModxgps4[, "SWEDD"]
rocModxg4<- roc(xgtrSM_Lab,vecXGBmod4)#
auc(rocModxg4) # Area under the curve: 0.9974       Area under the curve:9.38 with CSFasyn taus CNST removed;  0.9948 low without CNST; Area under the curve: 0.9933 wwithout CSF
ci.auc(rocModxg4) #95% CI: 0.9927-1 (DeLong)
rocModxgb4<- roc(xgtrSM_Lab,
          predict(xgbsmPDSW_4,
               type= "prob")[,"SWEDD"],
          levels = levels(xgtrSM_Lab)) #Area under the curve: 0.9974

thr<- .5
# converting to labels
xgb2ModLab4<- factor(ifelse(predModxgps4[, "SWEDD"] > thr, "SWEDD", "PD"))
confusionMatrix(xgb2ModLab4, xgtrSM_Lab, positive= "SWEDD")
# Sensitivity : 0.9773
# Specificity : 0.9545


#Validation/test xgb_2
colnames(xgtest3aMat[,-c(5:7, 14)])

predXGB4<- predict(xgbsmPDSW_4, newdata = xgtest3aMat[,-c(5:7, 14)], type ="prob")
rocXGB4<- roc(xgtest3a_Lab, predict(xgbsmPDSW_4,
                    newdata =xgtest3aMat[,-c(5:7, 14)], type ="prob")[,"SWEDD"],
        levels= levels(xgtest3a_Lab))
#
auc(rocXGB4)# Area under the curve: 0.8626
ci.auc(rocXGB4)# 95% CI: 0.7774-0.9479 (DeLong)


thr<- .5
#converting to labels
xgb4predlab<- factor(ifelse(predXGB4[, "SWEDD"] > thr, "SWEDD", "PD"))
confusionMatrix(xgb4predlab, test50a$ENROLL_CAT, positive= "SWEDD")

coords(rocXGB4, x = "best", best.method = "youden")
# threshold specificity sensitivity
# 0.5420807   0.7482993   0.9047619

#coords(rocXGB4,x = "best",input="threshold", best.method = "topleft") #same
```

```
# labs
thr<-  0.5420807
xgb4Labthr<- factor(ifelse(predXGB4[, "SWEDD"] > thr, "SWEDD", "PD"))
head(xgb4Labthr)
confusionMatrix(xgb4Labthr, xgtest3a_Lab, positive = "SWEDD")
# Sensitivity : 0.9048
# Specificity : 0.7483

colnames(testXG4dat<-xgtest3aMat[,-c(5:7, 14)])
rocbjXG<- plot.roc(xgtest3a_Lab,
            predict(xgbsmPDSW_4,testXG4dat,
                  type ="prob")[,"SWEDD"],
            levels= levels(xgtest3a_Lab),
            print.thres = "best",
            print.thres.best.method = "youden",
            print.auc= TRUE,
            legacy.axes =TRUE,
            print.thres.cex = .8,
            cex.axis= .6,
            cex.lab=.8)
plot(ci(rocbjXG, of= "thresholds", thresholds = "best"))
dev.off()

stopCluster(cl)
```
