# Supplementary material for: Non-motor Clinical and Biomarker Predictors Enable High Cross-Validated Accuracy Detection of Early PD but Lesser Cross-Validated Accuracy Detection of Scans Without Evidence of Dopaminergic Deficit
Source: Front Neurol. 2020 May 11;11:364. doi: 10.3389/fneur.2020.00364 (PMC7232850; doi:10.3389/fneur.2020.00364)
Supplement: Data Sheet 1 — Supporting Information I-V. [file Data_Sheet_1.ZIP › Appendices/Supporting information 1.docx]

*Supporting information 1 (S1)*

Figure Legends

FIG S1-1: early PD and control MoCA scores (histogram)

FIG S1-2: Linearity of logit plot, early PD/controls predictors

FIG S1-3: Composite: leverage (hat-values), discrepancy (studentized residuals), Cook’s Distance

FIG S1-4: Leverage values; early PD/control

FIG S1-5: Discrepancy values; early PD/control

FIG S1-6: Cook’s Distance; early PD/control

Expression 1: caret trainControl() settings

FIG S1-7: Linearity of logit plot, early PD/SWEDD continuous predictors

FIG S1-8: early PD/ SWEDD diagnostic plots

FIG S1-9: early PD/SWEDD leverage

FIG S1-10: early PD/SWEDD discrepancy

FIG S1-11: early PD/SWEDD Cook’s Distance

FIG S1-12: Decision tree, early PD/SWEDD ROC AUC, non-sampled vs. SMOTE data models

FIG S1-13: Random forest, early PD/SWEDD ROC AUC, non-sampled vs. SMOTE data

FIG S1-14: GLM (logistic) early PD/SWEDD ROC AUC, non-subsampled vs. SMOTE model

FIG S1-15: XGBoost early PD/SWEDD ROC AUC, non-sampled vs. SMOTE data models

FIG S1-16: early PD/SWEDD years of education (histogram)

FIG S1-17 early PD/SWEDD linearity of logit plot

FIG S1-18: Composite: leverage (hat-values), discrepancy (studentized residuals), Cook’s Distance values.

FIG S1-19: Leverage values; early PD/SWEDD

FIG S1-20 Discrepancy values; early PD/SWEDD

FIG S1-21 Cook’s Distance; early PD/SWEDD

FIG S1-22 Logistic regression plots

FIG S1-23 early PD/SWEDD Epworth sleepiness scale (histogram)

**FIG S1-1: early PD and HC (controls) MoCA**

**FIG S1-2 Linearity of the logit scatterplots with smoothers:, early PD/controls** MCATOT = Montreal cognitive assessment test;NP1CNST = constipation; rbdSum = rapid eye movement behaviour disorder questionnaire; RevUpsit.sum = University of Pennsylvania smell test

FIG S1-3: early PD/ control diagnostic plots

FIG S1-4 Leverage: 4% (13/298) with > .06 leverage

FIG S1- 5	Discrepancy: 4 of studentized residuals exceeded > ± 2.5

FIG S1-6	Cook’s Distance: no cases approached 1

**Expression 1**

**trainControl(method="repeatedcv", number=10, repeats=5,**

**savePredictions = TRUE, classProbs = TRUE,**

**search = "random", # reduce tree accuracy and ROC**

**summaryFunction = twoClassSummary)**

**FIG S1-7 Linearity of logit scatterplots with smoothers**, **early PD/ SWEDD**: gdsSum = Geriatric depression scale; MCATOT = Montreal cognitive assessment test; rbdSum = rapid eye movement behaviour disorder questionnaire; RevUpsit.sum = University of Pennsylvania smell test

FIG S1-8: early PD/ SWEDD diagnostic plots

FIG S1-9: Leverage early PD/SWEDD: 7% (15/220) with > .082

FIG S1-10: Discrepancy early PD/SWEDD

FIG S1-11: Cook’s Distance early PD/SWEDD

**FIG S1-12: Decsion tree: E**arly PD/SWEDD ROC AUC trained tree model original vs. subsampled data

**FIG S1-13 Random forest:** Rndom forest models:, early PD/SWEDD., original vs. subsampled (SMOTE) data

**FIG S1-14 GLM:** early PD/SWEDD ROC original vs. subsampled (SMOTE) data

**FIG S1-15: XGBoost**: early PD/SWEDD ROC AUC XGBoost , original non-sampled vs. subsampled (SMOTE) data models

**FIG S1-16:** Early PD/SWEDD, years of education; EDUCYRS = years of education

**FIG S1-17 Linearity of the logit scatterplots with smoothers (early PD/SWEDD).** rbdSum = rapid eye movement behaviour disorder questionnaire; RevUpsit.sum = University of Pennsylvania smell test; gdsSum = Geriatric depression scale; EDUCYRS = years of education

FIG S1-18: Composite: leverage (hat-values), discrepancy (studentized residuals), Cook’s Distance values.

FIG S1-19: Leverage values; early PD/SWEDD

FIG S1-20 Discrepancy values; early PD/SWEDD

FIG S1-21 Cook’s Distance; early PD/SWEDD

**FIG S1-22** Logistic regression plots**: (A)** x-axis is DAT scan mean putamen uptake, y-axis proportion of cases who are early PD relative to controls; (**B)** x-axis is is DAT scan mean putamen uptake, y-axis is proportion of cases who are early PD relative to scanswithout dopaminergic deficit (SWEDD); (C) x-axis is olfactory acuity based on reverse scaled UPSIT, y-axis is proportion of cases who are early PD relative to controls; (D) x-axis is olfactory acuity based on reverse scaled UPSIT, y-axis is proportion of cases who are early PD relative to SWEDD.

**FIG S1-23** early PD/SWEDD Epworth sleepiness scale; ESS = Epworth sleepiness scale
