# Supplementary material for: Non-motor Clinical and Biomarker Predictors Enable High Cross-Validated Accuracy Detection of Early PD but Lesser Cross-Validated Accuracy Detection of Scans Without Evidence of Dopaminergic Deficit
Source: Front Neurol. 2020 May 11;11:364. doi: 10.3389/fneur.2020.00364 (PMC7232850; doi:10.3389/fneur.2020.00364)
Supplement: Data Sheet 1 — Supporting Information I-V. [file Data_Sheet_1.ZIP › Appendices/Supporting information V.docx]

***Supporting information V***

*Features*

Biological predictors, the biomarkers, were cerebral spinal fluid (CSF) levels of beta-amyloid 1-42 (A*β*_1-42_), α-synuclein, tau phosphorylated at threonine 181 (pTau), and total tau (tTau). With respect to biomarkers, because hemoglobin contamination can influence the biologic measures, exclusion of samples with > 200 ng/ml has been recommended ^(45)^; this screening recommendation was adopted in the current work. The non-motor clinical measures were trait anxiety scores based on the 20-item trait anxiety (max 80 points or 4 points per question) scale from the State-Trait Anxiety Inventory ^(58)^; depression score based on the 15-item (max 15) Geriatric Depression Scale (GDS) ^(59)^; cognition based on the 30 point Montreal Cognitive Assessment Inventory (MoCA) ^(60)^, which has a maximum score of 30; olfactory acuity based on the 40-item (maximum score of 40) University of Pennsylvania Smell Identification Test (UPSIT) ^(61)^, which was reverse scaled (higher is proportional to lesser olfactory acuity); the 8-item (max score of 24) Epworth Sleepiness Scale (ESS) ^(62)^; the 10-item (max score of 13) rapid eye movement sleep behavior disorder questionnaire (RBDQ) ^(63)^; and constipation, ranging from 0 to 4 (MDS-UPDRS-I, ^(64)^. Sociodemographic measures also included under the rubric of non-motor clinical measures were age, gender and years of education. The biomarkers are continuous variables, and the clinical variables are continuous or semi-continuous scales. Note, for all clinical measures except MoCA, and given UPSIT was reverse scaled, higher scores are generally suggestive of pathology while the reverse typically holds for the biologics; higher CSF biologic values suggest a more normal state.

*Characterizing data symmetry*

Fig SV-1 density plots convey a lack of normal distribution symmetry typical of the predictor variables: 3 of the clinical and 3 of the biological variables are shown.

**FIG SV-1: Density plots.** UPSIT = University of Pennsylvania Smell Inventory Test, reverse-scaled;

RBSQ = rapid eye- movement Behaviour disorder questionnaire; MoCA = Montreal Cognitive

Assessment; HC = healthy controls; PD = early Parkinson’s disease; SWEDD= scans without

Evidence of dopamine deficit

***Classification result presentation format***

Classification results are presented in a two-table format: a confusion matrix Table (e.g. Table Xa) as well as a performance metric Table (e.g. Table Xb), where X is just a proxy in the context of this explanation for the actual table number. The performance metrics, and the confusion matrices are provided at the default .50-classification threshold and optimized classification threshold. As noted in methods (see Methods, *2.7 Classification performance metrics*)*,* and with one exception, the optimized model threshold was selected by the pROC package ^(1)^ utilizing a modified ^(2)^ version of the Youden Index ^(3)^.The confusion matrix is the basis of the performance metrics and is provided to support the performance metric findings. If there is no interest in recalculation of performance metrics from the confusion matrix, then the confusion matrix tables can be by-passed.

**SV-1. Model results, Early PD versus Control classification**

*SV-1.1 Decision tree classification analysis: early PD versus controls*

This section reviews the decision tree (early PD/control) classification. Model-specific results on training data are followed by performance classification probability estimates on the validation data-set. Of the original 14 predictors, built-in feature elimination (goodness of split) determined 6 variables or predictors of importance. The goodness of split ranking of features was largely in agreement with the generic feature elimination function in caret ^(4)^, though rank of predictors 4-6 differed (see Table SV-1).There are two column headings in Table SV-1: Scale 0-100% refers features/predictors of importance ranked from 0 to 100% based on their importance to the model classification ^(4)^. Goodness of split also ranks variables on a 0-100% scale of importance but is the *rpart* built-in variable of importance selection function. For further details on *rpart* Goodness of split and *rpar*t in general see Supporting information IV (*The models:* *Logistic regression, general additive, decision tree, random forest and XGBoost*).

**TABLE SV-1 Predictor importance: decision tree model early PD/control**

| *Predictors* | *Scaled 0-100%* | *Predictors* | *Goodness of split* |
| --- | --- | --- | --- |
| Upsit-rev | 100.00 | Upsit-rev | 53.00 |
| MoCA | 49.78 | MoCA | 11.40 |
| RBDQ | 16.30 | RBDQ | 4.65 |
| pTau | 13.49 | Age | 4,65 |
| A*β* _1-42_ | 4.38 | A*β* _1-42_ | 1.86 |
| Age | 0.00 | pTau | 1.39 |

A*β* _1-42_ = beta amyloid _1-42_; Upsit-rev = reverse scaled Upsit = University

of Pennsylvania Smell Identification Test ; MoCA = Montreal Cognitive

Assessment; pTau =tau phosphorylated at Thr181 at threonine 181 (p-Tau_181_)

_;_ RBDQ = rapid eye movement behavior disorder questionnaire; Goodness

of split: number of times a variable or its surrogate is used to split the data;

Scaled 0-100% = predictor importance to classification from

0-100%

Fig SV-2 provides the decision tree graphic of tree that used Table SV-1 predictors determined as important by the model to early PD versus control membership classification (tree depth was restricted to reduce image size). The single most important predictor is situated at the top of the tree (the root node) and predictors deemed of lesser importance by the algorithm are lower in the tree.

**FIG SV-2: Tree plot, early PD versus controls**. AB = beta-amyloid1-42 (A*β* _1-42_); MoCA = Montreal Cognitive Assessment ; Upsit = reverse-scaled University of Pennsylvania Smell Inventory Test; pTau = tau phosphorylated at Thr181 at threonine 181 (p-Tau_181_); RBDQ = rapid eye movement behaviour disorder. The top of each node displays the classification (and the very top number corresponds to the branch number), the mid-node shows the probability of the class at that node, and the lower node value is the percentage % of observations at the node. PD = early Parkinson’s disease, HC = healthy controls

The decision tree (*rpart*) default Gini index splitting rule was retained for model training. The pruned final tree model that utilized these same top 6 predictors listed in Table SV-1 was based on a complexity parameter (*cp)* overfitting corrective measure of *cp*= 0.0219 (and minsplit = 10, minbucket = 10); the *cp* parameter (the amount by which splitting a given node reduced the relative error) was initially derived from the lowest cross-validated error (xerror) associated with the lowest number of splits in the tree model. Subsequently, and as with the other tree-based models (random forest and XGBoost) the ROC AUC was the criterion used to select the optimal *cp* value of the final model during k-fold (10 folds, 5 repeats) resampling. The resulting final model, with a k-fold tuned *cp* parmemter, also selected a *cp* = 0.0219.

The model ROC AUC was .872 (95% CI: 0.831-0.913, DeLong; sensitivity .879; specificity .857). Table SV-2a confusion matrices specify the classification frequency predicted by the model applied to the validation data-set. Table SV-2b provides performance metrics for the model applied to the validation data-set. Results at the default .50 and optimal classification thresholds are provided in Tables SV-2a and 2b. The optimal classification threshold was .586; it was selected by the pROC package ^(1)^ utilizing a modified ^(2)^ version of the Youden Index ^(3)^.

**TABLE SV-2a: Decision tree confusion matrices, early PD/control**

| **Class** | **OBS** | **Predicted, K-fold model, thr = .50** | | **Predicted, K-fold model, thr = .586** | |
| --- | --- | --- | --- | --- | --- |
| PD | 88 | TN = 35 | FN = 16 | TN = 35 | FN = 16 |
| HC | 39 | FP = 4 | TP = 72 | FP = 4 | TP = 72 |

**Note:** *N* = 127 (validation data); K-fold, where k =10 (repeated 5 times for 50 models); HC = healthy controls;

OBS= observations; PD = early Parkinson’s disease; Predicted; K-fold model = predictions from k-fold model

applied to validation data-set; thr = threshold, e.g. thr of .50 refers trained model class prediction on the validation

data-set at the default threshold of .50; FN = false negative**;** FP = false positive**;** TN = true negative**;** TP = true positive

**TABLE SV-2b: Decision tree performance metrics, early PD/control**

| **Threshold** | **Model** | **Resampling** |  | **Cross-validated performance metrics** | | | | | |
| --- | --- | --- | --- | --- | --- | --- | --- | --- | --- |
|  |  |  |  |  | *ROC AUC (95% CI, DeLong)* | *ACC* | *Kappa* | *SN* | *SP* |
| .50 | Tree | 10-fold, 5 repeats |  |  | .860 (95% CI: 0.799-0.922) | .842 | .659 | .818 | .897 |
| .586 | Tree | 10-fold, 5 repeats |  |  | .860 (95% CI: 0.799-0.922) | .842 | .659 | . 818 | .897 |

**Note:** Predicted results on the validation data-set.ACC = accuracy; Tree = decision tree; Kappa = Cohen’s Kappa; ROC = receiver

operating characteristic; SP = specificity; SN = Sensitivity; Resampling = 10-fold, 5 repeats resampling of the model tuning

parameter (the complexity parameter), whereby the optimal hyper-parameter setting was determined by the AUC

The ROC AUC metric was .860 (95% CI .79, .92), indicating an ~ 86% chance the model would correctly distinguish between early PD and controls. At the optimized classification threshold of .586 performance metrics remained unaltered. The ROC curve with the optimized classification threshold (.586) is provided in Fig SV-3.

**Fig SV-3 Decsion tree ROC AUC:** .860; specificity .897, sensitivity .818;

error bars reflect variation in specificity and sensitivity threshold.

***1.2 Logistic regression and general additive model classification analyses, early PD versus controls***

This section reviews the logistic regression (GLM) and general additive (GAM) (early PD/control) model-specific results, which includes feature elimination, model assumption diagnostics, and the tabulated logistic and GAM model results. This is followed by predictive performance results on the validation data-set. The logistic regression GAM was used both to supplement the logistic regression GLM and as a distinct predictive classification model.

There was agreement between generic ^(4)^ and logistic regression stepwise feature elimination. The final 6 predictors ranked in order of greatest importance are in Table SV-3. The Table 5 predictors are ranked on a 0-100% scale ^(4)^, with the predictor of least importance being attributed zero value based for contribution to the model classification; the predictors are also ranked by coefficient z-scores. The coefficient z-values are simply the coefficient z-values of the model with all 6 predictors included.

**TABLE SV-3 Predictor importance: logistic model**

| *Predictors* | *Scaled 0-100%* | *Coefficient z-values* |
| --- | --- | --- |
| Upsit-rev | 100 | 7.60 |
| RBDQ | 34.36 | 2.96 |
| pTau | 26.08 | 2.36 |
| Age | 20.43 | 1.96 |
| MoCA median | 20.42 | 1.96 |
| CNST | 15.40 | 1.60 |
| MoCA high | 0.00 | 0.51 |

Upsit-rev = reverse scaled UPSIT = University of Pennsylvania

Smell Identification Test; pTau = CSF phosphorylated Tau;

MoCA = the Montreal Cognitive Assessment; RBDQ = rapid

eye movement behavior disorder questionnaire; Scaled = predictor

importance to classification on a 0-100% scale (higher values

indicate greater predictor importance)

Note that in Table SV-3 MoCA is a categorical variable. It was originally a semi-continuous variable but violated linearity of the logit and was converted to a 3-level factor: the reference is the MoCA low range of scores (≤ 26). MoCA mid-range has a median score of 28, and MoCA high has a range of 29-30. While MoCA proved to be a variable of lesser importance it nevertheless contributed to the model and its conversion from continuous to categorical warrants brief explanation. The semi-contiguous MoCA (0-30 integer scale) violated linearity of the logit ^(5)^. The remedial option used was to initially convert it to quartiles (e.g. https://rpubs.com/kaz_yos/logistic-linearity); quartiles were labeled Q1-Q4 constituted a 4-level categorical version of MoCA. There were only 3 cases equal to the base (Q1) score of 21 but there were 71 cases (all early PD) less than 27, which was the score marking the first quartile (.25). To reasonably represent the lower scores, the first two quantiles, Q1 and Q2, were merged, leaving a single low range for all instances with a score < 27. This resulted in a 3-level factor representing the MoCA low (21-26), midrange or median (28) and upper level scores (29-30).

The logistic regression analysis specifics of the predictors in Table 5 are provided in Table SV-4. Table SV-4 in addition to the coefficients ($\hat{\boldsymbol{\beta}}$**)**, standard errors ($\boldsymbol{SE}\hat{\hat{\boldsymbol{\beta}}}$**),** z-values and odds ratios, includes standardized predictor coefficients as well as rescaled coefficients (i.e. with score range binarized) (see 2.5 Statistics). Standardized and rescaled coefficients have been recommended ^(6)^ to lend an unbiased perspective to differentially scaled predicators (e.g. olfactory deficit based on the University of Pennsylvania Smell test has 0-40 scale, constipation based on the UPDRS I has a 0-4 scale), making such differentially scaled predictors comparable. Standardized predictor coefficients are provided for all predictors (except the categorical variable MoCA); rescaled coefficients are provided for Likert-type scales Upsit, rapid eye movement behaviour disorder (RBDQ), and constipation (CNST).

| **TABLE SV-4. Logistic regression early PD/controls** | | | |  |  | **Standardized** | | ***Rescaled*** |  |
| --- | --- | --- | --- | --- | --- | --- | --- | --- | --- |
| Variable | $\hat{\boldsymbol{\beta}}\boldsymbol{(SE)}$ | *z* | ***95% CI*** | ***Odds ratio*** | ***95% CI (Odds) (Odds) ratio)*** | $\hat{\boldsymbol{\beta}}$******* | ***Odds**** | $\hat{\boldsymbol{b}}$ | ***p*** |
| Intercept | -0.55(1.11) | -0.49 |  |  |  |  |  |  |  |
| Upsit-rev | 0.27(0.03) | 7.60 | (.21, .35) | 1.31 | (1.23, 1.41) | 2.48 | 11.95 | 10.82 | < .0001*** |
| RBDQ | 0.23(0.07) | 2.96 | (.08, .40) | 1.26 | (1.09, 1.49) | 0.66 | 1.93 | 3.05 | = .003** |
| pTau | -0.08(0.03) | -2.36 | (-.15, -.02) | .92 | (0.86, .98) | -0.54 | 0.58 | - | = .018* |
| Age | -0.03(0.01) | -1.96 | (-.07, .00) | .97 | (.93, 1.00) | -0.36 | 0.70 | - | = .051 |
| MoCA median | -0.93(0.47) | -1.96 | (-1.87, -1.01) | .40 | (.15, .99) | - | - | - | = .051 |
| CNST | 0.59(0.37) | 1.60 | (-.11, 1.36) | 1.80 | (.90, 3.91) | 0.37 | 1.45 | 1.77 | = .109 |
| MoCA high | -0.22(0.43) | -0.51 | (-1.06, .62) | .81 | (.35, 1.86) | - | - | - | = .612 |

*Note.* *N* = 298. CNST = MDS-UPDRS I NP1CNST; MoCA = Montreal Cognitive Assessment; Odds = odds ratio; Odds* = standardized odds ratio;

pTau = CSF phosphorylated Tau _181_; RBDQ = rapid eye movement behaviour disorder questionnaire; Upsit-rev = reverse-scaled University of Pennsylvania Smell Identification Test; $\hat{\boldsymbol{\beta}}$**** =*** standardized coefficient; $\hat{\boldsymbol{b}}$ = rescaled coefficient (scaled variables have same 0 to 1 range); McFadden pseudo *R*^2^ (*pR*^2^) = .47; Residual deviance: 194.42 on 290 df; AIC= 210..42. P-values are rounded 3 decimal places; other values are rounded two decimal places.

The biological predictors CSF α-synuclein, pTau, and tTau where highly correlated (*r_s_* > .75). To avoid violation of assumption of multicollinearity these variables were not entered concurrently into the same model. Table SV-4 specifies the final logistic model results derived from stepwise (backward, employing AIC) logistic regression, which controlled for age, years of education and gender. The pseudo *R^2^* ^(7)^ of this final logistic model was. 47, which suggests a very good model fit to the data (https://stats.stackexchange.com/questions/82105/mcfaddens-pseudo-r2-interpretation).

Elaborating briefly on Table SV-4 standardized significant results, holding other variables constant, a one standard deviation increase in reduced olfactory acuity (Upsit-rev in Table SV-4 and Fig SV-4) explains a 2.48 standard deviation increase in the log odds of early PD, an increase that significantly differed from zero, *z* = 7.60, *p* < .0001. For every standard deviation unit increase in reduced olfactory acuity the odds of early PD increased by a multiplicative factor of 11.95. Holding other variables constant, a one standard deviation increase in RDBQ was associated with a .66 standard deviation increase in the log odds of early PD, an increase that significantly differed from zero, *z* = 2.96, *p* = .003. Every standard deviation unit increase in RBDQ multiplies the odds of early PD by 1.93. Holding other variables constant, a one standard deviation increase in pTau was associated with a .54 standard deviation reduction in the log odds of early PD, a difference that significantly differed from zero, *z* = -2.36, *p* = .02. Every standard deviation unit increase in pTau reduces the odds of early PD by about half (.58). The two most potent predictors, olfaction (reverse scaled University of Pennsylvania Smell Test) and rapid eye-movement behaviour disorder (RBDQ), are depicted in effects plots in Fig SV-4, where the predicted probability of early PD group membership rises with higher predictor values.

**FIG SV-4. Effects plots**: the probability of early PD membership (y-axis) increases with higher predictor

values (x-axis); the y-axis is on the probability scale, shading around the lines reflects the 95% confidence

interval. RBDQ = rapid eye movement behaviour disorder questionnaire; Upsit-rev = University of

Pennsylvania Smell Identification Test (reverse-scaled).

An insignificant (*p* = .55) Hosmer and Lemeshow Goodness of Fit test suggested that the model was an effective classifier; there was not a significant difference between observed and predicted values, *Χ*^2^(4) = 2.3, *p* = .68. A final collinearity check using the variance inflation factor demonstrated no indications of high collinearity among predictors (the highest value was age at 1.24), and the Durbin-Watson test returned a value of 2.13, (*p* =. 22) indicating errors were not correlated. Logistic regression case-wise index plots of leverage, discrepancy and Cook’s Distance, did not reveal any cases sufficiently extreme to bias model results (see Supporting information I case-wise plots Figs S2-S6). While 4% of cases (13/298) had leverage beyond the cut-off (.06 here) and 4 cases exceeded a discrepancy of > ± 2.5, there were no cases that exceeded both these leverage and discrepancy criteria. In short, none of the cases attained a Cook’s Distance of 1 (the highest Cooks Distance value was .09). In addition, other than the MoCA variable already discussed, none of the other five predictors violated linearity of the logit.

The logistic regression GAM (referred to hereafter as just GAM) model specifics are provided in Supporting Information II, Table S2-1. The GLM included conversion of the semi-continuous MoCA in to quartiles. The MoCA variable violated the assumption of linearity of logit ^(5)^ and was converted in to quartiles. Although such conversion is common practice, it can result in a loss of information ^(8)^. The GAM model, which can retain all information, helped to further convey the relation of the MoCA variable to the probability of early PD classification (see *2.5 Statistical analyses*)*.* The Box-Tidwell ^(5)^ test for olfactory deficit (Upsit-rev), age, pTau, RDBQ and constipation all had insignificant Box-Tidwell *p*-values, though judging purely from the S1-2 plots these variables also had a degree of non-linear association with outcome. In short, although the Box-Tidwell test identified only MoCA was in violation of linearity of the logit, other predictors (notably age, pTau and olfactory deficit) had, arguably, a less than linear relationship with outcome on the logit scale. The utility of the logistic regression GAM to capture the non-linear characteristics of these variables was apparent; residual deviance was lowest and explained deviance (pseudo *R^2^*) were highest when all predictors, not simply MoCa, were transformed into thin plate smoother splines. The GAM model deviance (161.90) was significantly lower than logistic model deviance (194.43), *Χ^2^* (2.66) = 32.52, *p* < .0001. GAM pseudo *R^2^* (55.9%) was also higher relative to logistic regression pseudo *R*^2^ (47.0%). The GAM model output is provided in Supporting Information II, Table, S2-1. Interestingly, and as shown in Table S2-1, the MoCA variable was insignificant. In addition, of the six predictors, MoCA made the smallest contribution to the GAM model. The predictor ranking in descending order of importance was as follows based on p-values: Upsit-rev, RBDQ, age, pTau, constipation, and MoCA. With respect to GAM diagnostics, the estimated degrees of freedom (edf) did not closely approach predictor *k*-value. The default *k* = 10 (*k*-1= 9 maximum) produced good results but the following *k* values proved optimal to lower (residual) deviance and increase deviance explained (analogous to pseudo *R^2^*): age, *k*= 3; olfaction, *k*= 6; RDBQ, *k* = 4; MoCa, *k* = 5; and pTau, *k*=4; constipation, *k*=4. The residuals in the (quantile-quantile) qq.gam plot (see Supporting Information II, Fig S2-1) adhered quite closely to the diagonal, suggesting the data set quantiles were relatively well behaved and approximated the theoretical quantiles. Scatterplots of all predictors are provided in Supporting Information II, Fig S2-2.

The logistic regression (GLM) model ROC AUC was .920 (95% CI: 0.8877-0.9533, DeLong; sensitivity .934, specificity .797). Single predictor models using the top two predictors Upsit-rev (reverse-scaled University of Pennsylvania Smell Test) and RBDQ had poor (RDBQ, AUC .674) to good (Upsit-rev AUC .889) AUC outcomes.

The GAM model ROC AUC was .946 (95% CI: 0.9217-0.9702, DeLong; sensitivity .913, specificity .807). The GLM and GAM model predictive results are summarized in Tables SV-5 and SV-5b. The Table SV-5a confusion matrices specify the classification frequency predicted by the model applied to the validation data-set. Table SV-5b provides performance metrics for the model applied to the validation data-set. Results at the default .50 and optimal classification thresholds are provided.

The optimized threshold for the GLM was .462; the optimized threshold for the GAM was .534. The optimized model thresholds were selected by the pROC package ^(1)^ utilizing a modified ^(2)^ version of the Youden Index ^(3)^. The cross-validated GLM ROC AUC metric was .907 (95% CI: 0.8493-0.9642 (DeLong)), indicating an ~ 91% chance the model would correctly distinguish between early PD and controls. The cross-validated GAM ROC AUC metric was .928 (95% CI: 0.8778-0.9783 (DeLong)), indicating an ~ 93% chance the model would correctly distinguish between early PD and controls. The GLM and GAM model ROC curves are shown in Fig SV-5. With the exception of specificity, performance metrics were higher in the GLM and GAM models than in the decision tree model.

**TABLE SV-5a: Logistic regression GLM and GAM confusion matrices**

| **Model** | **Class** | **OBS** | **Predicted; thr = 0.5** | | **Predicted; opt. * thr.** | |
| --- | --- | --- | --- | --- | --- | --- |
| GLM | PD | 88 | TN = 34 | FN = 9 | TN = 34 | FN = 8 |
| GLM | HC | 39 | FP = 5 | TP = 79 | FP = 5 | TP = 80 |
| GAM | PD | 88 | TN = 34 | FN = 9 | TN = 35 | FN = 9 |
| GAM | HC | 39 | FP = 5 | TP = 79 | FP = 4 | TP = 79 |

**Note:** *N*= 127 (validation data-set); OBS= observations; PD = early PD; opt.

= optimized threshold (Youden Index); thr = threshold, e.g. thr of .50 refers

to trained model class predictions on validation data-set at the default thre-

shold of .50.; HC = healthy control; FN = false negative**;** FP = false positive**;**

TN = true negative**;** TP = true positive; * see TABLE 8b for optimized thresholds

**TABLE SV-5b: Logistic regression GLM and GAM performance metrics**

| **Threshold** | **Model** | **Resampling** |  | **Performance metrics: validation data-set** | | | | | |
| --- | --- | --- | --- | --- | --- | --- | --- | --- | --- |
|  |  |  |  |  | *ROC (95% CI)* | *ACC* | *Kappa* | *SN* | *SP* |
| .50 | GLM | None |  |  | .907 (95% CI: 0.849-0.964, DeLong) | .890 | .748 | .898 | **.**872 |
| .462 | GLM | None |  |  | .907 (95% CI: 0.849-0.964, DeLong) | .898 | .764 | .909 | .872 |
| .50 | GAM | None |  |  | .928 (95% CI: 0.878-0.978, DeLong) | .890 | .748 | .898 | .872 |
| .534 | GAM | None |  |  | .928 (95% CI: 0.878-0.978, DeLong) | .898 | .768 | .898 | .897 |

**Note:** Predicted results on validation data-set. ACC = accuracy; Kappa = Cohen’s Kappa; ROC = receiver operating

characteristic; SP = specificity; SN = sensitivity**;** Kappa = Cohen’s Kappa; ROC = receiver operating characteristic.

**FIG SV-5 Logistic regression GLM and GAM AUCs:** GLM ROC AUC**:** .907 (95% CI: 0.849-0.964, DeLong)**,** specificity .872, sensitivity .909; GAM ROC AUC .928 (95% CI: 0.878-0.978, DeLong), specificity .897, sensitivity .898; error bars reflect variation in specificity and sensitivity threshold

***1.3 Random forest classification analysis, early PD versus controls***

This section reviews the random forest (early PD/control) training data, model-specific results followed by predictive performance results on the validation data-set. From the 14 original predictors, the six most important predictors determined by feature elimination are listed in descending order of importance in Table SV-6. The predictors values listed under Scaled 0-100% in Table SV-6 are ranked on a 0-100% scale^(4)^ based on contribution to the model**.** Predictor values in Table SV-6 listed under Mean Gini decrease are ranked by the extent to which they contributed to the mean decrease in the Gini index (the decrease in impurity), across all trees. The Mean Gini decrease is the built-in random forest measure of predictor importance.

**TABLE SV-6: Predictor importance, random forest**

| Predictors | *Scaled 0-100%* |  | Predictors | *Mean Gini decrease* |
| --- | --- | --- | --- | --- |
| Upsit-rev | 100 |  | Upsit-rev | 41.38 |
| MoCA | 40.82 |  | MoCA | 13.70 |
| RBDQ | 22.83 |  | A*β* _1-42_ | 13.62 |
| CSF α-syn | 1.59 |  | CSF α-syn | 13.40 |
| Age | 0.20 |  | RBDQ | 12.96 |
| A*β* _1-42_ | 0.00 |  | Age | 12.25 |

A*β* _1-42_ = beta amyloid _1-42_; CSF α-syn = CSF alpha synuclein; Mean

Gini decrease = variable importance to average Gini (impurity) decrease;

Upsit-rev= reverse scaled University of Pennsylvania Smell Identification;

RBDQ = rapid eye movement behaviour disorder questionnaire; Vars Imp

Scaled = predictor importance to classification on a 0-100% scale

The ROC AUC criterion, used to select the optimal tuning parameters during the k-fold (10-fold x 5 repetitions) model training procedure, determined an optimal mtry = 1 hyper-parameter. The optimal model, based on the predictors in Table 8, also had an ntree (the number of trees grown) value of 3000 trees and a node size of 3. The out of bag (OOB) error rate was 14.09%. The OOB error is graphed in Fig SV-6. See Supporting information IV for an explanation of OOB error in random forest.

**FIG SV-6 Out of bag error analysis:** HC = healthy controls;

OOB = out of bag error; PD = early Parkinson’s disease

The model ROC AUC was .999 (95% CI: 0.9989-1, DeLong; sensitivity 1.00, specificity .9341). Model predictive results (from applying the model to the validation/test data-set) are summarized in Tables SV-7a and SV-7b. The Table SV-7a confusion matrices specify the classification frequency predicted by the model applied to the validation data-set. Table SV-7b provides performance metrics for the model applied to the validation data-set. Results at the default .50 and optimal classification thresholds are provided. The optimized threshold (.534) was selected by the pROC package ^(1)^ utilizing a modified ^(2)^ version of the Youden Index ^(3)^.

The predicted cross-validated ROC AUC was of .913 (95% CI: 0.8585-0.9672, DeLong) indicated an approximate 91% probability the model would correctly distinguish between early PD and controls. This was a higher AUC than the cross-validated (CV) .860 achieved by the decision tree model, comparable to the CV AUC of logistic model (rounded to two decimal places), and a little lower than the GAM .928 CV AUC.

**TABLE SV-7a: Random forest confusion matrices**

| **Class** | **OBS** | **Predicted, K-fold model, thr = 0.5** | | **Predicted, K-fold model, thr = .541** | |
| --- | --- | --- | --- | --- | --- |
| PD | 88 | TN = 31 | FN = 5 | TN = 34 | FN = 8 |
| HC | 39 | FP = 8 | TP = 83 | FP = 5 | TP = 80 |

**Note:** *N*= 127 (validation data-set); K-fold, where k =10 (repeated 5 times for 50 models); OBS=

observations; K-fold model= predictions from k-fold model applied to validation data-set; thr =

threshold, e.g. thr of .50 refers trainedmodel class prediction on validation data-set at the default

threshold of .50FN = false negative**;** FP = false positive**;** TN = true negative**;** TP = true positive

**TABLE SV-7b: Random forest performance metrics**

| **Threshold** | **Model** | **Resampling** |  | **Cross-validated performance metrics** | | | | | |
| --- | --- | --- | --- | --- | --- | --- | --- | --- | --- |
|  |  |  |  |  | *ROC (95% CI, DeLong)* | *ACC* | *Kappa* | *SN* | *SP* |
| .50 | RF | 10-fold, 5 repeats |  |  | . 913 (95% CI: 0.858-0.968) | .898 | .754 | .943 | .795 |
| .534 | RF | 10-fold, 5 repeats |  |  | . 913 (95% CI: 0.858-0.968) | .898 | .764 | .909 | .872 |

**Note:** Predicted results on validation data-set. ACC = accuracy; Kappa = Cohen’s Kappa; ROC = receiver operating

characteristic;SP = specificity; SN = sensitivity**;** Kappa = Cohen’s Kappa; RF = random forest; ROC = receiver operating

characteristic; Resampling = 10-fold, 5 repeats resampling of the model tuning parameters (i.e. mtry, but ntree, nodesize

were also assessed), whereby the optimal hyper-parameter setting was determined by the AUC

A plot of the ROC AUC, with sensitivity and specificity at the optimized threshold, is provided in Fig SV-7.

**FIG SV-7 Random Forest:** ROC AUC: .913**;** specificity .872, sensitivity

.909; error bars reflect variation in specificity and sensitivity threshold

*1.4 XGBoost classification analysis, early PD versus controls*

This section reviews the XGBoost (early PD/control) classification analysis. Feature selection results are followed by review of XGBoost optimized tuning parameters determined by resampling. Subsequently, the predictive performance outcome on the validation data-set is reviewed. From the 14 original predictors, both XGBoost built-in and caret ^(4)^ generic feature elimination were in agreement with respect the features most important to classification, though after the 2 most important predictors ranking order differed. The SV-8 most important predictors are listed in descending order of importance in Table SV-8.The predictors values listed under Scaled 0-100% in Table 10 are ranked on a 0-100% scale ^(4)^ based on contribution to the model**.** Under the Table 10 heading Gain, predictors are ranked by the extent to which they contributed to the XGBoost gain index. Gain is a predictor importance metric built-in to XGBoost that measures a feature’s contribution to each tree in the model and hence overall prediction; higher values are better.

**TABLE SV-8: Predictor importance, XGBoost**

| Predictors | Scaled 0-100% |  | Predictors | Gain |
| --- | --- | --- | --- | --- |
| Upsit-rev | 100 |  | Upsit-rev | 0.5436 |
| MoCA | 27.23 |  | MoCA | 0.1404 |
| A*β* _1-42_ | 15.25 |  | RBDQ | 0.0535 |
| α-syn | 13.43 |  | A*β* _1-42_ | 0.0523 |
| RBDQ | 12.77 |  | Anxiety | 0.0486 |
| Age | 9.98 |  | Age | 0.0416 |
| Anxiety | 9.20 |  | α-syn | 0.0414 |
| CNST | 8.15 |  | EDYRS | 0.0303 |
| EDYRS | 5.97 |  | CNST | 0.0193 |
| Depression | 2.77 |  | Depression | 0.01932 |
| Sex | 2.58 |  | ESS | 0.0114 |
| ESS | 0.00 |  | Sex | 0.002 |

A*β* _1-42_ = CSF beta amyloid _1-42_; Anxiety = trait anxiety from the

State-trait anxiety inventory; CNST = constipation based

on the UPDRS I scale; α-syn = CSF alpha synuclein;

EDYS = years of eduction; ESS = Epworth daytime sleepiness

scale; Gain = variable importance to prediction, higher is better;

GDS = Geriatric depression scale; MoCA = Montreal cognitive

Assessment test; Upsit-rev= reverse scaled University of

Pennsylvania Smell Identification; RBDQ = rapid eye

movement behaviour disorder questionnaire; Vars Imp Scaled

= predictor importance to classification on a 0-100% scale

A range of tuning parameter settings were assessed during resampling (10 fold, repeated 5 times) using the ROC AUC as the optimal parameter selection criterion. Tuning parameter guidelines adopted (<https://www.slideshare.net/OwenZhang2/tips-for-data-science-competitions>) were endorsed by the creators of XGBoost ^(9)^. The optimal tuning parameters found were as follows: eta .02; nrounds 500; max_depth 5; min_child_weight 1; colsample_bytree .4; gamma 5; subsample setting of .5. For further XGBoost hyper-parameter information Supporting information IV (*The models:* *Logistic regression, general additive, decision tree, random forest and XGBoost*).

The model ROC AUC was .958 (95% CI: 0.9374-0.9795, DeLong; sensitivity .937; specificity .835). Model predictive results are summarized in Tables SV-9a and SV-9b. The Table SV-9a confusion matrices specify the classification frequency predicted by the model applied to the validation data-set. Table SV-9b provides performance metrics for the model applied to the validation data-set. Results at the default .50 and optimal classification thresholds are provided. The optimized model threshold (.660) was selected by the pROC package ^(1)^ where the optimal threshold was the point nearest to the top-left point of perfect sensitivity/specificity. A plot of the XGBoost ROC AUC is provided in Fig SV-8.

The ROC AUC statistic on the (external) validation data set was .923 (95% CI: 0.8747-0.9721, DeLong), indicating an approximate 92% chance the model would correctly distinguish between early PD and controls. This was a higher ROC AUC than the .860 achieved by the decision tree, similar to the random forest (.911) and logistic model (.907) AUCs but marginally less than the .928 AUC achieved by the GAM model.

**TABLE SV-9a: XGBoost confusion matrices**

| **Class** | **OBS** | **Predicted, K-fold model, thr = 0.5** | | **Predicted, K-fold model, thr = .660** | |
| --- | --- | --- | --- | --- | --- |
| PD | 88 | TN = 31 | FN = 9 | TN = 35 | FN = 11 |
| HC | 39 | FP = 8 | TP = 79 | FP = 4 | TP = 77 |

**Note:** *N*= 127 (validation data-set); K-fold, where k =10 (repeated 5 times for 50 models); OBS=

observations; K-fold model = predictions based on k-fold model applied to validation data-set;

thr = threshold, e.g. thr of .50 refers trainedmodel class prediction on validation data-set at the

default threshold of .50.; FN = false negative**;** FP = false positive**;** TN = true negative**;** TP = true

positive

**TABLE SV-9b: XGBoost performance metrics**

| **Threshold** | **Model** | **Resampling (k-fold)** |  | **Performance metrics: validation data-set** | | | | | |
| --- | --- | --- | --- | --- | --- | --- | --- | --- | --- |
|  |  |  |  |  | *ROC (95% CI, DeLong)* | *ACC* | *Kappa* | *SN* | *SP* |
| .50 | XGB | 10-fold, 5 repeats |  |  | .923 (0.875-0.972) | .866 | .708 | .898 | .795 |
| .660 | XGB | 10-fold, 5 repeats |  |  | .923 (0.875-0.972) | .882 | .736 | .875 | .897 |

**Note:** K-fold model predicted results on validation data-set. ACC = accuracy; Kappa = Cohen’s Kappa; ROC = receiver operating

characteristic; SP = specificity; SN = sensitivity**;** Kappa = Cohen’s Kappa; RF = random forest; ROC = receiver operating characteristic;

Higher performance outcomes are in bold type; XGB = XGBoost model. Resampling = 10-fold, 5 repeats resampling of the model

tuning parameters (e.g. eta, nrounds, etc), whereby the optimal hyper-parameter setting was determined by the AUC

**FIG SV-8 XGBoost ROC AUC**: .923 (95% CI: 0.875-0.972, DeLong)**;**

specificity is .897, sensitivity is .875; error bars reflect variation in

specificity and sensitivity; optimized threshold was .660 determinded

the point nearest o the top-left point of perfect sensitivity/specificity

selected by the pROC package (Robin et al 2011)

***1.5 Early PD versus control model applied to SWEDD; long-term* *conversion of SWEDD to PD***

In the preceding early PD/control classification analyses the GAM model achieved the highest AUC of .928 (sensitivity .898, specificity .897) when applied to the (early PD/control) validation data. The XGBoost model, when applied to the validation data, had a similar result (AUC .923, sensitivity .875, specificity .897). The GAM model (using features selected by stepwise regression), was more parsimonious, and used far fewer predictors than the XGBoost model. This section reports the results of these two higher performing early PD versus HC classifiers applied to the SWEDD data. Model performance on validation data is reviewed as is model utility predicting conversion of SWEDD to PD or PD-like at 12-36 months post baseline.

The (early PD/control) classifiers map to SWEDD versus control. The results are an indicator of model predicted classification of SWEDD as early PD and hence model predicted conversion of SWEDD to early PD. Note that the early PD/control classifiers are being applied to SWEDD/control test data. As such, the diverse mix of PD-like (e.g. essential tremor, peripheral neuropathy, psychogenic illness, apparently normal etc.) as well as PD neuropathology that makes-up SWEDD is being shoehorned to fit early PD. Consequently, what the models classify as PD will be a mix of conditions, with the actual diagnosis being revealed in the longitudinal data (see below, *Longitudinal diagnosis vs. model predicted SWEDD to PD conversion*). The control data is the same control validation data used to validate the early PD/control models. The validation data set comprised of SWEDD (*n*= 43, 25 male) and control validation sets (*n* = 39 controls, 26 male) was isolated from the models during training.

Confusion matrices resulting from the application of these two models to the SWEDD validation data is provided in Supporting information III. Table SV-10 contains the performance metrics for the two early PD/control classifiers applied to the SWEDD/control validation data. Table SV-10 includes results at the default .50 and optimal classification thresholds. The optimized model threshold was selected by the pROC package ^(1)^ utilizing a modified ^(2)^ version of the Youden Index ^(3)^. The ROC AUC plots of the early PD versus control models applied to SWEDD versus control validation data are provided in Fig SV-9.

**TABLE SV-10: XGBoost and GAM early PD/control models applied to SWEDD versus control validation data**

| **Threshold** | **Model** | **Resampling (k-fold)** |  | **Performance metrics: validation data-set** | | | | | |
| --- | --- | --- | --- | --- | --- | --- | --- | --- | --- |
|  |  |  |  |  | *ROC AUC (95% CI, DeLong)* | *ACC* | *Kappa* | *SN* | *SP* |
| .50 | XGB | 10-fold, 5 repeats |  |  | .831 (0.742-0.921) | .732 | .466 | .674 | .795 |
| .378 | XGB | 10-fold, 5 repeats |  |  | .831 (0.742-0.921) | .805 | .608 | .837 | .769 |
| .50 | GAM | None |  |  | .863 (0.786-0.941) | .780 | .564 | .698 | .872 |
| .389 | **GAM** | None |  |  | .863 (0.786-0.941) | .829 | .658 | .814 | .846 |

**Note:** K-fold model predicted results on validation data-set. ACC = accuracy; Kappa = Cohen’s Kappa; ROC = receiver operating

characteristic; SP = specificity; SN = sensitivity**;** Kappa = Cohen’s Kappa; RF = random forest; ROC = receiver operating characteristic;

XGB = XGBoost model. Resampling = 10-fold, 5 repeats resampling of the model tuning parameters (e.g. eta, nrounds, etc),

whereby the optimal hyper-parameter setting was determined by the AUC

**Fig SV-9: ROC AUC graphs, GAM and XGBoost early PD vs. control models applied to SWEDD vs. control validation data.** Left**,** GAM = general additive model, ROC AUC .863 (95% CI: 0.786-0.941, DeLong), at the optimized threshold of .389 sensitivity = .814, specificity = .846); Right, XGBoost model, ROC AUC .831 (95% CI: 742-0.921, DeLong)**,** at the optimized threshold of .378 specificity =.769, sensitivity =.837. Error bars reflect variation in specificity and sensitivity threshold

GAM and XGBoost model performance metrics were very similar (see Table SV-10). Not surprisingly, 39 of the validation data samples classified by the GAM model as PD or PD-like (true positive plus false positives) matched the 39/45 (87%) validation sample cases classified by the XGBoost model as PD or PD-like. In keeping with the similar model results, a modified ^(1)^ bootstrap (*n* = 2000) test ^(10)^ for correlated ROC curves indicated there was not a significant ROC AUC difference between the GAM and XGBoost models, *D* = 1.27, *p* = .21.

Reiterating the sensitivity and specificity results, at the optimized classification threshold (.389), the GAM model differentiated 35/43 (81.4%, sensitivity) SWEDD as PD-like or early PD. There were 33/39 (84.6%, specificity) subjects correctly classified as controls. For the XGBoost model, the optimized classification threshold (.378) differentiated 36/43 (83.7%, sensitivity) SWEDD as PD-like or early PD. There were 30/39 (76.9 %, specificity) subjects correctly classified as controls. Next, the PPMI longitudinal diagnoses were compared with model predicted classification to determine the percentage of actual SWEDD to PD conversions relative to cases that proved to be PD-like but not PD pathology.

***1.6 Longitudinal diagnosis vs. model predicted SWEDD to PD conversion***

The (likely) longitudinal primary diagnosis used was the diagnosis at the longitudinal time point furthest from baseline (i.e. the 36 month diagnosis was used if available rather than 12 or 24 month value). Likewise, DAT scan (not modelled) mean putamen values referenced used values at the longitudinal time point furthest from baseline (e.g. the 36 month value was used if available rather than 12 or 24 month value).

For the GAM model, and at its optimum classification threshold (.389), 12/38 (32%) baseline SWEDD predicted by the GAM model to be PD-like were diagnosed as idiopathic PD by 12-36 months in the PPMI available longitudinal data. However, the majority of these data instances, 26/38 (68%), were not classified in the 12-36 month longitudinal data as idiopathic PD but diagnosed as another PD-like category or apparently normal. This broad category was made-up of 1 Alzheimer’s disease case, 1 possible dementia, 1 polyneuropathy, 1 dystonia tremor, 1 parkinsonism, 1 vascular parkinsonism, 1 lateral sclerosis, 1 peripheral neuropathy, 4 essential tremor, 3 psychogenic illness and 11 apparently normal cases. Four of those predicted by the model to convert to idiopathic PD (4/12 model true positives) and re-diagnosed by 24 months as PD, had DAT scan evidence of dopaminergic dysfunction suggested by relatively low mean putamen DAT scan values (*M* = 1.24, *SD*= .73). The mean 12-36 month putamen value averaged across all 12 cases predicted by the model to convert from SWEDD to PD (including the above mentioned four) was 1.49 (*SD* = .68). The mean putamen value of 26/38 predicted by the model to convert from SWEDD to PD but that converted to status other than PD was 2.01 (*SD*= .47). The mean putamen value of the model predicted control cohort was 2.14 (*SD* = .57). The 24-month time point diagnosis data also indicated that none of the 33 model predicted controls converted to PD.

The XGBoost early PD/control model applied to the SWEDD versus control validation set had similar results. At the optimum classification threshold (.378), the available longitudinal 12-36 month PPMI data indicated 13/43 (30%) baseline SWEDD predicted by the XGBoost model to be PD-like had converted from SWEDD to idiopathic PD. As occurred for the GAM model, the majority of these data instances, 34/43 (79%), were not classified in the 12-36 month longitudinal data as idiopathic PD but diagnosed as a collective of diverse disorders similar to those noted for the GAM model. The XGBoost model predicted conversions also included the same four SWEDD subjects found by the GAM model with lower DAT scan values suggestive of dopaminergic dysfunction. The mean putamen value of the remaining 20/34 (59%) cases predicted by the model to convert to PD but that converted to status other than control or PD was 2.05 (*SD*= .49). The mean putamen value of the controls was 2.09 (*SD* = .57). As with the GAM model, none of the model predicted controls was diagnosed at 12-24 months as PD.

**2.0: *Model results, Early PD versus SWEDD classification SMOTE-based models***

This section reviews results for the early PD/SWEDD classification. Consistent with the early PD/control analyses, the early PD versus SWEDD model-specific results (e.g. logistic regression coefficients and odds ratios; tree model optimal tuning parameter settings, etc.) are followed by predictive performance results. Subsampling (i.e.. Synthetic Minority Oversampling Technique [SMOTE]) was used to mitigate the SWEDD 13% minority class rate in the training data*.* As noted previously, resampling (10-fold, 5 repeats, distinct from subsampling) was applied to tree-based models (decision tree, random forest, XGBoost) but not to the GLM or GAM models. The GLM does not have tuning parameters to resample (though the parameters or coefficients can be resampled but were not here), and at the time of this writing the resampling interface used ^(4)^ did not permit specification of GAM formula particulars deemed important to model optimization.

The ROC AUC was used to assess overall model classification performance. Subsampling methods applied were ROSE, and SMOTE. However, the ROSE model introduced negative synthetic values with each implementation; an occurrence reported elsewhere (<https://stats.stackexchange.com/questions/166458/rose-and-smote-oversampling-methods>). Given all values in the data were positive, the injection of synthetic negative values was deemed unrealistic and the ROSE method was not employed in the analyses. Model results are presented in the following order: decision tree, logistic regression and general additive models, random forest and XGBoost.

SMOTE subsampling was used on training data in this analysis. SMOTE altered data instances from an imbalanced training set of 148 PD and 22 SWEDD to 44 instances of each class. It warrants mention, that while the SMOTE derived training data had symmetrical class balance (44= early PD and 44 SWEDD), the validation data set was left in its original class-asymmetric state: 147 early PD and just 21 SWEDD (with a gender division of 10 females to 11 males).

***2.1 Decision tree early PD versus SWEDD classification results (SMOTE-based model)***

This section reviews the decision tree (early PD/SWEDD) training data model-specific results followed by predictive performance results of the decision tree classification probability estimates on the internal validation data-set of hold-out samples. The AUC of the SMOTE model was higher than the AUC of the model based on non-subsampled data. Consequently the SMOTE-based model was used. Fig S1-12 conveys the ROC AUC, sensitivity and specificity outcomes of the original non-subsampled as well as the SMOTE-based models. Note, the ROSE model outcome is also included in S1-12; it had marginally better results than the SMOTE based model but, as already noted, introduced negative values in the tree and other models, and was not employed in the analyses.

An additional subsampling related finding occurred only with the decision tree model. Unlike the other tree models, decision tree SMOTE data derived from subsampled during (10 fold x 5 repeats) resampling could be readily extracted. A modified ^(1)^ bootstrap (n = 2000) test ^(10)^ for unpaired ROC curves indicated the model ROC AUC from the SMOTE subsampled data, where the SMOTE data was obtained during resampling had a significantly higher ROC AUC (.932) compared to the ROC AUC (.799) of the model based on SMOTE data that was not subsampled during k-fold resampling, *D* = 2.66,p = .008. An explanation and example of subsampling during resampling is readily available (<https://topepo.github.io/caret/subsampling-for-class-imbalances.html#resampling>). Consequently, the SMOTE data derived from subsampling during resampling was used (*n* = 88 early PD (63 male); *n* = 66 SWEDD (41 male). Again, this applied only to the decision tree model.

Of the original 14 predictors, built-in feature elimination (goodness of split) determined 10 predictors of importance from SMOTE subsampled training data that resulted in the model with the highest AUC. Table SV-11 lists the 10 predictors of greatest importance to classification. Scale 0-100%, a generic predictor ranking method used throughout this work, ranks features of importance ranked from 0 to 100% based on their importance to the model classification ^(4)^. Goodness of split also ranks variables on a 0-100% scale of importance but is the *rpart* built-in variable of importance selection function. Note that the descending order of predictor rank differs between the generic (Kuhn 2007) and built-in (Goodness of split) variable of importance methods after the top two predictors.

**TABLE SV-11 Predictor importance: decision tree model, early PD/SWEDD**

| *Predictors* | *Scaled 0-100%* | *Predictors* | *Goodness of split* |
| --- | --- | --- | --- |
| Upsit-rev | 100.00 | Upsit-rev | 24.64 |
| ESS | 89.69 | ESS | 16.20 |
| Age | 87.44 | MoCA | 10.53 |
| EDYRS | 79.76 | tTau | 10.52 |
| tTau | 56.77 | Age | 9.67 |
| A*β* _1-42_ | 51.20 | RDBQ | 9.03 |
| MoCA | 29.98 | A*β* _1-42_ | 7.22 |
| Anxiety | 5.59 | EDYRS | 3.10 |
| RBDQ | 3.81 | Depression | 2.71 |
| Depression | 0.00 | Anxiety | 1.83 |

A*β* _1-42_ = beta amyloid _1-42_; Depression = Geriatric depression scale; Upsit-rev = reverse scaled

UPSIT = University of Pennsylvania Smell Identification Test ; MoCA =Montreal Cognitive

Assessment; tau phosphorylated at Thr181 at threonine 181(p-Tau_181_RBDQ = rapid eye move-

ment behavior disorder questionnaire; Goodness of split: number of times a variable or its surr-

ogate is used to split the data; Scaled = predictor importance to classification on a 0-100%

Fig SV-10 is a tree plot utilizing Table 14 predictors. The single most important predictor to classification of group membership is situated at the top of the tree (the root node). The predictors deemed of lesser importance by the algorithm are lower in the tree. The decision tree (*rpart*) default Gini index splitting rule was retained for model training. For the pruned final model, the predictors listed in Table SV-11 were based on a complexity parameter (*cp)* overfitting corrective measure of *cp*= .0180 (and default minsplit = 20, minbucket = 7). The ROC AUC was the criterion used to select the optimal *cp* value of the final model during a k-fold model training procedure.

The model ROC AUC was .932 (95% CI: 0.8936-0.9712, DeLong; sensitivity .891, specificity .900). Model (cross-validated) predictive results are summarized in Tables 12a and 12b. The Table 12a confusion matrices specify the classification frequency predicted by the model applied to the validation data-set.

**Fig SV-11: Tree plot, Early PD vs. SWEDD:** MoCA = Montreal Cognitive Assessment; Olfaction = reverse-scaled University of Pennsylvania

Smell Inventory Test; (Upsit-rev) tTau = total Tau; ESS = Epworth Daytime Sleepiness scale. The top of each node displays the

classification (and the very top number corresponds to the branch number), the mid-node shows the probability of the class at that

node, and the lower node value is the percentage (% ) of observations at the node. PD = early Parkinson’s disease, SWEDD = scans

without dopamine deficit

Table 12b provides performance metrics for the model applied to the validation data-set. Results at the default .50 and optimal classification thresholds are provided. The optimized model threshold (.486) was selected by the pROC package ^(1)^ utilizing a modified ^(2)^ version of the Youden Index ^(3)^.

**TABLE 12a: Decision tree confusion matrices, early PD/SWEDD (SMOTE-based model)**

| **Class** | **OBS** | **Predicted, K-fold model, thr = 0.5** | | **Predicted, K-fold model; opt. thr. = .486** | |
| --- | --- | --- | --- | --- | --- |
| PD | 147 | TN = 120 | FN = 27 | TN = 120 | FN = 7 |
| SWEDD | 21 | FP = 7 | TP = 14 | FP = 27 | TP = 14 |

**Note:** *N*= 168 (validation data-set); K-fold, where k =10 (repeated 5 times for 50 models); OBS= observations;

K-fold model = predictions based on k-fold model applied to validation data-set; thr = threshold, e.g.

thr of .50 refers trainedmodel prediction on the validation data-set at the default threshold of .50.; FN =

false negative**;** FP = false positive**;** TN = true negative**;** TP = true positive

**TABLE 12b: Decision tree performance metrics, early PD/SWEDD (SMOTE-based model)**

| **Threshold** | **Model** | **Subsampling** | **Resampling** |  | **Performance metrics: validation data-set** | | | | | |
| --- | --- | --- | --- | --- | --- | --- | --- | --- | --- | --- |
|  |  |  |  |  |  | *ROC (95% CI, Delong)* | *ACC* | *Kappa* | *SN* | *SP* |
| .50 | Tree | SMOTE | 10-fold, 5 repeats |  |  | .743 (95% CI: 0.617-0.869) | .798 | .343 | .341 | .945 |
| .486 | Tree | SMOTE | 10-fold, 5 repeats |  |  | .743 (95% CI: 0.617-0.869) | .798 | .343 | .667 | .816 |

**Note:** K-fold model predicted results on validation data-set. ACC = accuracy; Kappa = Cohen’s Kappa; ROC = receiver operating characteristic; SP = specificity; SN = sensitivity**;** Kappa = Cohen’s Kappa; RF = random forest; ROC = receiver operating characteristic; Resampling = 10-fold, 5 repeats resampling of the model tuning parameter(s), whereby the optimal hyper-parameter setting was determined by the AUC; SMOTE = synthetic minority oversampling technique; Tree = decision tree.

The ROC AUC metric of .743 (95% CI: 0.617-0.867, DeLong), indicated an approximate 74% chance the model would correctly distinguish between early PD and SWEDD. The ROC curve with the optimized classification threshold (.486) is provided in Fig SV-12.

**FIG SV-12 Decision tree ROC AUC:** specificity .816; sensitivity .667.

Error bars reflect variation in specificity and sensitivity threshold

***2.2 Logistic regression and general additive classification analysis, early PD vs. SWEDD (SMOTE-based model)***

This section reviews the logistic regression model (GLM) and general additive model GAM results; feature elimination, model assumption diagnostics, and tabulated GLM results are provided. This is followed by predictive performance results for both the GLM and GAM models on the validation data-set. As in the early PD/control analysis, the logistic regression GAM was used here both to supplement the logistic regression GLM and as a distinct predictive classification model. The GLM results will be reviewed first followed by the GAM results.

Fig S1-14 (see *Supporting Information 1*) conveys that the SMOTE subsampled model had a higher AUC compared to the AUC of model that used original, non-subsampled data. As in the early PD versus control analysis, feature elimination used stepwise (backward) regression employing AIC to select predictors of greatest import to classification from a SMOTE subsampled training data-set. Table SV-13 lists the 6 predictors ranked in descending order of importance that made the greatest contribution to the GLM model. Table SV-13 ranks predictors on a 0-100% scale ^(4)^, with the predictor of least importance being attributed zero. The predictors are also ranked by coefficient z-scores - the coefficient z-values of the model with all 6 predictors included. As explained below, one of the predictors was converted to quartiles, which increased the number of model variables to 8. This complied with the minimum of 5 variables per predictor ^(11)^, given there were 44 cases for each class (*N*= 88). However, the variable gender was under represented, with each class having 14 females and 30 males.

The variable number of years of education (EDYRS in Table SV-13) was converted from an integer to a categorical variable comprised of 4 levels (EDYRS Q1-Q4). As occurred in the early PD/control GLM analysis but with a different variable, the variable years of education violated linearity of the logit ^(5)^. Scatterplots (with smoothers) of all predictor values against the logit is provided in Fig S1-17. Remedially, the years of education variable was converted to (categorical) quartiles and (*k*-1, = 3) dummy variables. The reference was the lowest quartile, and the dummy variables are the lower quartile (EDYRS Q2), the upper quartile (EDYRS Q3), and those with years of education exceeding the upper quartile (EDYRS Q4). Of note, while 17 early PD exceeded the upper quartile of 18 or more years of education just 7 SWEDD exceeded 18 years of education. The SWEDD cohort had significantly fewer years of education relative to the early PD cohort (see Table 2).

**TABLE SV-13 Predictor importance, logistic regression**

| Predictors | *Scaled 0-100%* | *Coefficient z-values* |
| --- | --- | --- |
| Upsit-rev | 100 | 3.90 |
| Age | 73.90 | 3.15 |
| RBDQ | 69.62 | 3.01 |
| EDYRS Q4 | 67.94 | 2.96 |
| EDYRS Q3 | 46.18 | 2.32 |
| Sex | 44.81 | 2.28 |
| Depression | 34.31 | 1.97 |
| EDYRS Q2 | 0.00 | .971 |

Coefficient z-scores = absolute value of coefficient z-scores; Depression =

Geriatric depression scale; Upsit-rev= (UPSIT) University of

Pennsylvania Smell Identification Test, reverse-scaled; Scaled = predictor

importance to classification on a 0-100% scale; EDYRS Q2

= years of education below the lower quartile (25% ); EDYRS Q3 =

years of education below the upper quartile (75%); EDYRS Q4 = years

of education above Q3; RBDQ = rapid eye movement behavior disorder

questionnaire

The logistic regression analysis specifics are provided in Table SV-14. Coefficients ($\hat{\boldsymbol{\beta}}$**)**, standard errors ($\boldsymbol{SE}\hat{\hat{\boldsymbol{\beta}}}$**),** z-values and odds ratios, standardized predictor coefficients as well as rescaled coefficients (i.e. with score range binarized) (see 2.5 Statistics) are included. As noted previously, standardized and rescaled coefficients have been recommended ^(6)^ to lend an unbiased perspective to differentially scaled predicators. Here, olfaction based on the University of Pennsylvania Smell Test (UPSIT; reverse-scaled in the current work [Upsit-rev]), has 0-40 scale, gender contrast is binary (0,1). Coefficients rescaled to a binary scale were (Likert-type variables) Upsit-rev, rapid eye movement behaviour disorder, and the geriatric depression scale. Standardized predictor coefficients are provided for all predictors (except for the categorical variable years of education).

| **TABLE SV-14. Logistic regression early PD/SWEDD** | | | |  |  | **Standardized** | | ***Rescaled*** |  |
| --- | --- | --- | --- | --- | --- | --- | --- | --- | --- |
| Variable | $\hat{\boldsymbol{\beta}}\boldsymbol{(SE)}$ | *z* | ***95% CI*** | ***Odds ratio*** | ***95% CI (Odds) (Odds) ratio)*** | $\hat{\boldsymbol{\beta}}$******* | ***Odds**** | $\hat{\boldsymbol{b}}$ | ***p*** |
| Intercept | -0.8(3.43) | -2.40 |  |  |  |  |  |  |  |
| Upsit-rev | -0.36(0.09) ((0.03) | -3.90 | (-.57, -.21) | .70 | (.59, .84) | -2.79 | 0.06 | -14.25 | < .0001*** |
| Age | 0.13(0.04) | 3.13 | (.06, .22) | 1.14 | (1.05, 1.23) | 1.34 | 3.81 | - | = .002** |
| RBDQ | 0.49(.16) | 3.01 | (.21, .86) | 1.63 | (1.19, 2.25) | 1.50 | 4.50 | 6.39 | = .003** |
| EDYRS Q4 | -4.36(1.47) | -2.96 | (-7.68, -1.82) | .01 | (.00, .23) | - | - | - | = 003** |
| EDYRS Q3 | -2.90(1.25) | -2.32 | (-5.68, -0.65) | .05 | (.00, .64) | - | - | - | = .020* |
| Sex (male) | 2.54(1.11) | 2.28 | (.57, 5.06) | 2.69 | (1.43, 112.37) | 1.18 | 3.26 | - | = .022* |
| Depression | 0.64(0.32) | 1.97 | (.07, 1.38) | 1.90 | (1.01, 3.58) | 0.97 | 2.64 | 1.77 | = .048* |
| EDYRS Q2 | -1.22(1.26) | -0.97 | (-3.88, 1.17) | .29 | (.02, .3.48) | - | - | - | = .332 |

*Note.* *N* = 88. EDYRS Q2= lower quartile for number of education years; EDYRS Q3 = upper quartile for number of education years; EDYRS Q4=number of education years of those exceeding the upper quartile. RBDQ = rapid eye movement behaviour disorder questionnaire; Upsit-rev = University of Pennsylvania Smell Identification Test (reverse-scaled); $\hat{\boldsymbol{\beta}}$**** =*** standardized coefficient; $\hat{\boldsymbol{b}}$ = rescaled coefficient (scaled variables have same 0 to 1 range); McFadden pseudo *R*^2^ (*pR*^2^) = .56; Odds = odds ratio; Odds* = standardized odds ratio; Residual deviance: 54.09 on 79 df; AIC= 72.09. P-values are rounded 3 decimal places; other values are rounded two decimal places.

A likelihood ratio test indicated the model was a significantly improved classifier relative to the null (no-predictor) model, *χ*^2^ (8) = 67.9, *p* = < .000. The McFadden pseudo *R^2^* value of .56 suggests the model provided a good fit to the data and hence good predictive power to explain classification (https://eml.berkeley.edu/~mcfadden/travel.html). The Hosmer-Lemeshow Goodness of Fit test was insignificant, *Χ*^2^ (8) = 1.59, *p* = .95, indicating there was not a significant difference between observed and predicted values. This suggests the model was an effective classifier.

It warrants reiterating again that olfaction was reverse-scaled (Upsit-rev), making higher values indicative of reduced olfactory acuity (e.g. in Table 2 the SWEDD group has a much lower olfactory impairment score). With greater precision and stability accorded to variables with narrow confidence intervals ^(12)^, olfaction, age and RDBQ were the predictors of note. Interpretation of the results will be confined to the results for olfaction, RBDQ, and years of education variable (EDYRS Q4) representing those exceeding the upper quartile. Holding other predictors constant, a one standard deviation increase in reduced olfactory acuity explained a 2.79 standard deviation decrease in the log odds of SWEDD, a decrease that significantly differed from zero, *z* = -3.90, *p* < .0001. With other predictors partialed out, the odds of reduced olfactory acuity in SWEDD was 6% of the odds of reduced olfactory acuity in early PD. Holding other predictors constant, a one standard deviation increase in RBDQ explains a 1.50 standard deviation increase in the log odds of SWEDD, an increase that significantly differed from zero, *z* = 3.01 *p* = .003. With other predictors partialed out, a one standard deviation increase in RBDQ multiplies the odds of SWEDD by 4.50. Finally, holding other predictors constant, and relative to the base lowest level of years of education (first quartile), each year increase in education beyond the upper quartile reduced the log odds of SWEDD by 4.36, a reduction that significantly differed from zero, z = -2.96, *p* = .003. Relative to the lowest level of years of education, the odds of SWEDD having greater than 18 years of education (above the third quartile here) was 1% of the odds of early PD having greater than 18 years of education.

Effects plots for three highest ranked logistic regression GLM predictors, olfaction, age and RBDQ, are provided in Fig SV-13, where the predicted probability (y-axis) of SWEDD group membership decreases with higher olfactory deficit (increasing x-axis values) but increases with higher RBDQ score and age.

**FIG SV-13 Effects plots**: predicted probability (y-axis) of SWEDD group membership decreased with higher olfaction deficit (left) but increased with higher RBDQ scores and age (right). Upsit-rev = University of Pennsylvania Smell Inventory Test; (reverse-scaled); RBDQ = rapid eye movement behaviour disorder questionnaire; SWEDD = scans without evidence of dopamine deficit

In a final check on collinearity, the variance inflation factor for logistic regression determined an absence of explanatory variable concern for collinearity, and the Durbin-Watson test (2.23, *p* = .27) indicated errors were not correlated. Case-wise diagnostics included index plots of leverage, discrepancy and Cook’s Distance. There was no indication of cases sufficiently extreme to bias model results (see Supporting information 1 case-wise plots Figs S1 18-21). Approximately 3% of cases (3/88) had leverage beyond the cut-off (.31) and there were no cases that exceeded a discrepancy of > ± 2.5. None of the cases attained a Cook’s Distance of 1 (the highest Cooks Distance value was .42, and second highest was .12).

The GLM included conversion of years of education variable (which violated the assumption of linearity of logit) to quartiles. Because this conversion can result in a loss of some data ^(8, 13, 14)^ the GAM model helped to further convey the relation of the years of education variable to the probability of SWEDD classification. A GAM model treating the years of education variable as a thin plate spline had significantly lower residual deviance (46.04) compared to the GLM (54.09), *χ*^2^ (1.12)= 8.05, *p* = .006. GAM pseudo *R^2^* (62.3%) was also higher relative to logistic regression pseudo *R^2^* (56.0%). The GAM model output is provided in Supporting information II, Table, S2-2. The predictor ranking in descending order of importance was as follows based on *p*-values: Upsit-rev, RBDQ, age, years of education, gender, and depression. With respect to GAM diagnostics, the estimated degrees of freedom (edf) did not closely approach predictor *k*-value. The optimal k-value of the lone smoothed term, years of education, was *k*= 7. The residuals in the (quantile-quantile) qq.gam plot (see Supporting information II, Fig S2-3) adhered quite closely to the diagonal, suggesting the data set quantiles approximated the theoretical quantiles. A scatterplot of years of education (smoothed using a thin plat spline) is provided in Supporting information II, Fig S2-4.

The logistic regression (GLM) model ROC AUC was .938 (95% CI: 0.8877-0.9533, DeLong; sensitivity .864; specificity .841). Single predictor AUC values were calculated only with logistic regression. Single logistic regression predictor models using the top 3 predictors had poor to moderate AUC values: age AUC .543; RBDQ AUC .701; hyposmia AUC .760. Based on modified ^(1)^ bootstrap (*n* = 2000) test ^(10)^ for correlated ROC curves, the 6-predictor integrated model (actually an 8-predictor model counting the actual education quartiles modeled) had significantly improved classification compared to the top single predictor hyposmia model, *D* = 3.62, *p* < .001. The GAM model ROC AUC was .955 (95% CI: 0.916-0.9941, DeLong; sensitivity .886; specificity .909).

The GLM and GAM model predictive results are summarized in Tables SV-15a and SV-15b. The Table SV-15a confusion matrices specify the classification frequency predicted by the model applied to the validation data-set. Table SV-15b provides performance metrics for the model applied to the validation data-set. Results at the default .50 and optimal classification thresholds are provided. The optimized GLM threshold was .504; only marginally different from the default .50 threshold. As such, confusion matrices and performance metrics for the default and optimized threshold were identical. The optimized GAM threshold was .437. Optimized model thresholds were selected by the pROC package ^(1)^ utilizing a modified ^(2)^ version of the Youden Index ^(3)^. ROC AUC GLM graphs for the GLM and GAM cross-validated models are provided in Fig SV-14.

**TABLE SV-15a: Logistic regression GLM and GAM confusion matrices, early PD/SWEDD**

|  | **Class** | **OBS** | **Predicted; thr = 0.5** | | **Predicted; opt. thr.** | |
| --- | --- | --- | --- | --- | --- | --- |
| GLM | PD | 147 | TN = 111 | FN = 7 | TN = 111 | FN = 7 |
| GLM | SWEDD | 21 | FP = 36 | TP = 14 | FP = 36 | TP = 14 |
| GAM | PD | 147 | TN = 117 | FN = 8 | TN = 112 | FN = 6 |
| GAM | SWEDD | 21 | FP = 30 | TP = 13 | FP = 35 | TP = 15 |

**Note:** *N*= 168 (validation data-set); OBS= observations; GAM = general additive

model; GLM = generalized linear model (logistic regression here); PD = early PD;

opt. = optimized threshold (Youden Index); thr = threshold, e.g. thr of .50 refers

to trained model class predictions on validation data-set at the default threshold of .50.;

SWEDD= scans without evidence of dopamine deficit; FN = false negative**;** FP = false

positive**;** TN = true negative**;** TP = true positive

**TABLE SV-15b: Logistic regression GLM and GAM performance metrics, early PD/SWEDD**

| **Threshold** | **Model** | **Subsampling** | **Resampling** |  | **Performance metrics: validation data-set** | | | | | |
| --- | --- | --- | --- | --- | --- | --- | --- | --- | --- | --- |
|  |  |  |  |  |  | *ROC (95% CI, Delong)* | *ACC* | *Kappa* | *SN* | *SP* |
| .50 | GLM | SMOTE | None |  |  | .779 (95% CI: 0.677-0.880) | .744 | .265 | .667 | **.**755 |
| .504 | GLM | SMOTE | None |  |  | .779 (95% CI: 0.677-0.880) | .744 | .265 | .667 | **.**755 |
| .50 | GAM | SMOTE | None |  |  | .787 (95% CI: 0.689-0.886) | .774 | .286 | .619 | .796 |
| .437 | GAM | SMOTE | None |  |  | .787 (95% CI: 0.689-0.886) | .756 | .299 | .714 | .762 |

**Note:** Predicted results on validation data-set. ACC = accuracy; GAM = general additive model; GLM = generalized linear model (logistic

regression here); ROC = receiver operating characteristic; SP = specificity; SN = sensitivity**;** Kappa = Cohen’s Kappa; ROC = receiver opera-

ting characteristic; SMOTE = synthetic minority oversampling technique.

**FIG SV-14 Logistic regression GLM and GAM AUCs:** GLM ROC AUC**:** .779 (95% CI: 0.677-0.880, DeLong)**,** specificity .667, sensitivity .755; GAM ROC AUC .787 (95% CI: 0.689-0.886, DeLong), specificity .762, sensitivity .714; error bars reflect variation in specificity and sensitivity threshold

***2.3 Random forest early PD versus SWEDD classification (SMOTE-based model)***

This section reviews the random forest (early PD/SWEDD) analysis; feature elimination, and model specific findings are followed by model predictive performance results from application of the model to the validation data set. Fig S1-13 (see *Supporting Information 1*) conveys that the SMOTE subsampled model had a higher AUC compared to the AUC of model that used original, non-subsampled data. The SMOTE subsampled data, as in the GLM and GAM analyses, altered data instances from an imbalanced training set of 148 PD and 22 SWEDD to 44 instances of each class (30 male in each class).

From the original 14 predictors of the SMOTE augmented training data, the mean Gini decrease ranking of predictor importance (built-in to random forest) and the generic variable of importance function ^(4)^ agreed generally on feature elimination: the 12 of 14 predictors were retained are listed in Table SV-16 (this set of features resulted in a model with the highest AUC; see Table SV-17b). However, as is evident in Table SV-16, the ranking of predictors though identical up to and including the forth-ranked predictor age, differed thereafter. In Table SV-16 under Scaled 0-100%, predictors are ranked on a 0-100% scale ^(4)^ based on contribution to the model**;** under the heading Mean Gini decrease, predictors are ranked by the extent to which they contributed to the mean decrease in the Gini index (the decrease in impurity) across all trees.

The ROC AUC criterion, used during select the optimal tuning parameters during the k-fold (10-fold x 5 repetitions) model training procedure, determined an optimal mtry = 1 hyper-parameter. The optimal model had an ntree (the number of trees grown) value of 3000 trees and a node size of 1. Fig SV-15 includes two random forest out-of-bag (OOB) error plots. Plot (A) shows the OOB error rate of the original model, without subsampling. Plot (B) shows the OOB error rate for the SMOTE based model yielded substantive improvement in OOB error rate, particularly for SWEDD.

**TABLE SV-16: Predictor importance, random forest**

| Predictors | Scaled 0-100% |  | Predictors | Mean Gini decrease |
| --- | --- | --- | --- | --- |
| Upsit-rev | 100 |  | Upsit-rev | 5.27 |
| ESS | 74.16 |  | ESS | 4.41 |
| RBDQ | 60.37 |  | RBDQ | 3.89 |
| Age | 45.28 |  | Age | 3.61 |
| Anxiety | 44,16 |  | A*β* _1-42_ | 3.60 |
| MoCA | 39.46 |  | Anxiety | 3.55 |
| EDYRS | 35.19 |  | EDYRS | 3.36 |
| CNST | 30.02 |  | CSF α-syn | 3.19 |
| A*β* _1-42_ | 20.43 |  | MoCA | 3.10 |
| Depression | 19.53 |  | Depression | 2.76 |
| CSF α-syn | 9.59 |  | CNST | 1.60 |
| Sex | 0.00 |  | Sex | .960 |

A*β* _1-42_ = beta amyloid _1-42_; Anxiety = State trait anxiety inventory; CNST =

Constipation based on the UPDRS I; CSF α-syn = CSF alpha synuclein;

ESS = Epworth daytime sleepiness scale; MeanGini decrease = variable

importance to average Gini (impurity) decrease; MoCA = Montreal

cognitive assessment; Upsit-rev= (UPSIT) University of Pennsylvania

Smell Identification Test, reverse-scaled; RBDQ = rapid eye movement

behaviour disorder questionnaire; Scaled = predictor importance to

classification ona 0-100% scale.

**FIG SV-16**: (A) Out-of-bag (OOB) error rate original non-subsampled data; (B) OOB rate

Synthetic Minority Over-sampling Technique (SMOTE) data

The model ROC AUC was 1.00 (95% CI: 1-1, DeLong; sensitivity 1.00; specificity 1.00), which is inevitable by design when the optimal nodesize =1, as occurred here. Model (cross-validated) predictive results are summarized in Tables SV-17a and SV-17b. The Table SV-17a confusion matrices specify the classification frequency predicted by the model applied to the validation data-set. Table SV-17b provides performance metrics for the model applied to the validation data-set. Results at the default .50 and optimal classification thresholds are provided. The optimized threshold (.461) was selected by the pROC package ^(1)^ where the optimal threshold was the point nearest to the top-left point of perfect sensitivity/specificity. The ROC AUC graph with the optimized threshold is provided in Fig SV-17.

**TABLE SV-17a: Random forest confusion matrices, early PD/SWEDD**

| **Class** | **OBS** | **Predicted, K-fold model; thr = 0.5** | | **Predicted;, K-fold model, opt. thr. = .461** | |
| --- | --- | --- | --- | --- | --- |
| PD | 142 | TN = 116 | FN = 7 | TN = 106 | FN = 4 |
| SWEDD | 21 | FP = 31 | TP = 14 | FP = 41 | TP = 17 |

**Note:** *N*= 168 (validation data-set); OBS= observations; PD = early PD; opt. = optimized threshold (closest t

op-left); thr = threshold, e.g. thr of .50 refers to trained model class predictions on validation data-set at

the default threshold of .50.; SWEDD= scans without evidence of dopamine deficit; FN = false negative**;** FP =

false positive**;** TN = true negative**;** TP = true positive; K-fold, where

k =10 (repeated 5 times for 50 models)

**TABLE SV-17b: Random forest performance metrics, early PD/SWEDD**

| **Threshold** | **Model** | **Subsampling** | **Resampling** | **Performance metrics: validation data-set** | | | | | |
| --- | --- | --- | --- | --- | --- | --- | --- | --- | --- |
|  |  |  |  |  | *ROC (95% CI, Delong)* | *ACC* | *Kappa* | *SN* | *SP* |
| .50 | RF | SMOTE | 10-fold x 5 |  | .822 (95% CI: 0.746-0.899) | .774 | .306 | .667 | .789 |
| .461 | RF | SMOTE | 10-fold x 5 |  | .822 (95% CI: 0.746-0.899) | .732 | .302 | .809 | **.**721 |

**Note:** K-fold model predicted results on validation data-set. ACC = accuracy; Kappa = Cohen’s Kappa; ROC = receiver operating char-acteristic; SP = specificity; SN = sensitivity**;** RF = random forest; ROC = receiver operating characteristic; SMOTE = Synthetic Minority Oversampling Technique

**FIG SV-17** ROC AUC early PD/SWEDD: AUC = .822; sensitivity =

.810, specificity = .721. Optimized threshold based on point closest

to top-left plot region of perfect sensitivity/specificity; error bars reflect

variation in specificity and sensitivity threshold

*2.4 XGBoost early PD versus SWEDD classification (SMOTE-based model)*

This section reviews the XGBoost (early PD/SWEDD) classification analysis. Feature selection results are followed by review of XGBoost optimized tuning parameters determined by resampling. Subsequently, the predictive performance results on the validation data-set is reviewed. Fig S1-15 (see *Supporting Information 1*) conveys that the SMOTE subsampled model had a higher AUC compared to the AUC of model that used original, non-subsampled data. The SMOTE subsampled data used in this analysis, and in the other analyses (GLM, GAM, random forest) altered data instances from an imbalanced training set of 148 PD and 22 SWEDD to 44 instances of each class (30 male in each class).

From the original 14 predictors of the SMOTE augmented training data, the gain index of predictor importance (built-in to XGBoost) and the generic variable of importance function ^(4)^ selected a similar set of predictors of greatest importance, but the gain index excluded gender indicating it did not contribute to the model. The final model 10 most important predictors, resulting in the highest AUC (see Table 19b), are listed by descending order of importance in Table SV-18. The predictors listed under scaled 0-100% in Table 20 are ranked on 0-100% scale ^(4)^ based on contribution to the model**;** predictors listed under Gain are ranked by extent of contribution to the XGBoost Gain index. Gain, an XGBoost built-in index of predictor importance, measures a feature’s contribution to each tree in the model and hence overall prediction; higher values are better. Note that the built-in (gain-based) and generic based ranking of predictor importance was the same for the first 3 predictors but differed thereafter. The k-fold resampling determined α-synuclein (absent from the Scaled 0-100 list in Table SV-18) was not important to XGBoost differentiation of SWEDD from early PD.

**TABLE SV-18: Predictor importance, XGBoost**

| Predictors | Scaled 0-100% |  | Predictors | Gain |
| --- | --- | --- | --- | --- |
| Upsit-rev | 100.00 |  | Upsit-rev | 0.2070 |
| ESS | 91.55 |  | ESS | 0.1602 |
| EDYRS | 63.75 |  | EDYRS | 0.1396 |
| MoCA | 42.37 |  | CSF α-syn | 0.1017 |
| RBDQ | 37.98 |  | Age | 0.0719 |
| A*β* _1-42_ | 28.08 |  | Anxiety | 0.0828 |
| Age | 23.71 |  | RBDQ | 0.0667 |
| Depression | 12.57 |  | MoCA | 0.0640 |
| Anxiety | 12.23 |  | A*β* _1-42_ | 0.0493 |
| CNST | 0.00 |  | Depression | 0.0216 |

A*β* _1-42_ = beta amyloid _1-42_; Anxiety = trait anxiety from the

State-trait anxiety inventory; EDYS = years of eduction; ESS

= Epworth daytime sleepiness scale;Gain = variable importance

to prediction, higher is better;Depression = Geriatric depression

scale; MoCA = Montreal Cognitive Assessment test; Upsit-rev

= reverse-scaled University of Pennsylvania Smell Identification;

RBDQ = rapid eye movement behaviour disorder questionnaire;

Scaled 0-100 %= predictor importance to classification on a 0-

100% scale; Gain = predictors ranked by the extent of contrib-

ution to the XGBoost gain index

As in the XGBoost early PD/control analysis, a range of tuning parameter settings were assessed during resampling (10 fold, repeated 5 times), using the ROC AUC as the optimal parameter selection criterion. Tuning parameter guidelines adopted (<https://www.slideshare.net/OwenZhang2/tips-for-data-science-competitions>) were endorsed by the creators of XGBoost ^(9)^. The optimal tuning parameters found were as follows: eta 0.2; nrounds 500; max_depth 4; min_child_weight 1; colsample_bytree 0.8; gamma 3; subsample setting of .75. See *Supporting information IV* (*The models:* *Logistic regression, general additive, decision tree, random forest and XGBoost Logistic regression)* for an overview of XGBoost tuning parameters.

The model ROC AUC was .997 (95% CI: 0.9927-1; sensitivity .977; specificity .954). Model predictive results are summarized in Tables SV-19a and SV-19b. The Table SV-19a confusion matrices specify the classification frequency predicted by the model applied to the validation data-set. Table SV-19b provides performance metrics for the model applied to the validation data-set. Results at the default .50 and optimal classification thresholds are provided. The optimized model threshold (.542) was selected by the pROC package ^(1)^ utilizing a modified ^(2)^ version of the Youden Index ^(3)^.

**TABLE SV-19a: XGBoost confusion matrices, early PD/SWEDD (*SMOTE-based model*)**

| **Class** | **OBS** | **Predicted, K-fold model; thr = 0.5** | | **Predicted;, K-fold model, opt. thr. = .542** | |
| --- | --- | --- | --- | --- | --- |
| PD | 142 | TN = 104 | FN = 2 | TN = 110 | FN = 2 |
| SWEDD | 21 | FP = 43 | TP = 19 | FP = 37 | TP = 19 |

**Note:** *N*= 168 (validation data-set); OBS= observations; PD = early PD; opt. = optimized threshold based a

modified version of the Youden Index; thr = threshold, e.g. thr of .50 refers to trained model class predi-

ctions on validation data-set at the default threshold of .50.; SWEDD= scans without evidence of dopamine

deficit; FN = false negative**;** FP = false positive**;** TN = true negative**;** TP = true positive; K-fold, where k =10

(repeated 5 times for 50 models)

**TABLE SV-19b: XGBoost performance metrics, early PD/SWEDD (*SMOTE-based model*)**

| **Threshold** | **Model** | **Subsampling method** | **Resampling** | **Performance metrics: validation data-set** | | | | | |
| --- | --- | --- | --- | --- | --- | --- | --- | --- | --- |
|  |  |  |  |  | *ROC (95% CI, DeLong)* | *ACC* | *Kappa* | *SN* | *SP* |
| .50 | XGB | SMOTE | 10-fold x 5 |  | .863 (0.777-0.948) | .732 | .333 | .905 | .707 |
| .542 | XGB | SMOTE | 10-fold x 5 |  | .863 (0.777-0.948) | .768 | .381 | .905 | **.**748 |

**Note:** K-fold model predicted results on validation data-set. ACC = accuracy; Kappa = Cohen’s Kappa; ROC = receiver operating

characteristic; SP = specificity; SN = sensitivity**;** RF = random forest; ROC = receiver operating characteristic; SMOTE = Synthetic

Minority Oversampling Technique

The ROC AUC cross-validated statistic (on the validation data set) was .863 (95% CI: 0.7774-0.9479, DeLong), indicating an approximate 86% chance the model would correctly distinguish between SWEDD and early PD. This was the highest ROC AUC achieved in the early PD/SWEDD classification analyses. The ROC AUC of the XGBoost model applied to the validation data-set is plotted in Fig SV-19.

**FIG SV-19 XGBoost ROC AUC**: .863 (0.777-0.948, DeLong**;** sensitivity

.905 specificity .748; error bars reflect variation in specificity and

sensitivity; threshold .542 determinded by the Youden Index (Youden

1950) modified by (Perkins and Schisterman 2006) and selected by the

pROC package (Robin et al 2011)

***2.5 Early PD versus SWEDD (SMOTE) model longitudinal fidelity***

As in section *4.5,* PPMI longitudinal data (12-36 months) was again used to assess long-term predicative utility of the models. However, section *4.5* was largely concerned with the early PD versus control model utility to identify SWEDD to PD conversions, and how model predictions faired against longitudinal diagnosis. This section describes early PD versus SWEDD model longitudinal accuracy in terms of sensitivity and specificity measures. Specifically, the interest was to determine the extent to which early PD versus SWEDD model cross-validated sensitivity and specificity was maintained at 12-36 months from baseline. Given the heterogeneous nature of SWEDD, evident from section *4.5* and reported elsewhere ^(15)^, case instances of model identified SWEDD persisting as virtually any non-PD category in the longitudinal data were interpreted as persistence of model SWEDD classification fidelity. As specified previously, the training data for both classifiers consisted of SMOTE resampled data (*N*= 88: 44 early PD, 30 male; 44 SWEDD, 30 male); the validation test data set was also previously specified (*N*= 168, 147 early PD, 92 male; 21 SWEDD, 11 male).

The two top performing early PD versus SWEDD SMOTE-based models, random forest and XGBoost were assessed. Cross-validated AUC, sensitivity and specificity performance results of the two models are reiterated here for convenience. This is followed by a simple assessment of model long-term classification utility: case-wise model sensitivity, specificity are compared with longitudinal case-wise diagnoses. DAT scan mean putamen values, not modelled but used as in indicator of disease, were also included as a diagnostic measure. Models, as noted in methods, utilized baseline data only. As specified in section *4.5*, the (likely) longitudinal primary diagnosis used was the diagnosis at the longitudinal time point furthest from baseline: i.e. 36 months from baseline at the time of this writing but few records were available beyond 24 months. Likewise, DAT scan mean putamen values referenced used values at the longitudinal time point furthest from baseline (e.g. the 48 month value, if available, was used rather than 12 or 24 month value).

The early PD versus SWEDD XGBoost model applied to the early PD versus SWEDD validation data achieved an AUC of .863, with sensitivity of .905 and specificity of .743 at the optimum threshold (.542). The early PD versus SWEDD random forest model applied to the early PD versus SWEDD valuation data achieved an AUC of .822, with sensitivity of .809 and specificity of .721 at the optimum threshold (.461). The sensitivity measure was comprised of 19/21 (90.47%) cases common between models. The specificity measure consisted of 143/151 (94.71%) cases common between models.

In the 12-36 month PPMI longitudinal data, identical case data was available and used by both models for assessing model sensitivity and specificity conformity to long-term diagnosis; 16/21 sensitivity measure cases and 128/147 specificity cases were available in the 12-36 month longitudinal data. How model sensitivity (true positives and false negatives) and specificity (true negatives and false positives) projected in the long-term data differed modestly between models. Sensitivity true positives refer to agreement between model classified SWEDD and the actual observation class at baseline. An objective was to determine the extent model predicted SWEDD true positives persisted with a non-PD diagnosis longitudinally. Also of interest was extent of long-term diagnosis agreement with model predicted early PD false negatives (model predicted PD contrary to baseline categorization of SWEDD), part of the sensitivity measure. With respect to specificity, model true negatives (early PD here) refer to case classification agreement between the model predicted class and actual observation class at baseline. Model false positives (model prediction of SWEDD class contrary to actual baseline observation PD classification) the other part of specificity, were also assessed for agreement with longitudinal data.

At its optimal cutoff (.461), random forest long-term sensitivity amounted to 12/16 (75%) correct class predictions, predictions that matched the 12-24 month diagnoses records available. This approximation of model long-term sensitivity was calculated from baseline model prediction matches with 12-24 month diagnoses as follows: the random forest model misclassified 4/16 (25%) cases as SWEDD that were diagnosed as PD at 12-24 months; two of three model false negatives classified as early PD by the model proved to be correct predictions and were diagnosed as PD at 12-24 months; the remaining false negative case classified as early PD by the model was diagnosed as essential tremor not PD in the longitudinal data; 10/13 (76.9%) model true positive SWEDD cases matched the long-term 12-24 months SWEDD diagnosis. The latter SWEDD group of 10, all cases without evidence of PD-pathology, consisted of 2 essential tremor, 3 other neurological disorders, 4 cases that were without evident disease, and 1 case of Alzheimer’s disease. In sum, the approximation of model long-term sensitivity amounted to 12/16 (75%) correct class predictions. The mean putamen DAT scan value averaged across all SWEDD/non-PD at 12-36 months was 2.06 (*SD* = .50). With respect to model-approximated specificity, 92/128 (71.87%) cases (true negatives) were correctly classified by the model as PD at 12-24 months. There were 36/128 (28.12%) cases (model false positives) incorrectly classified by the model as SWEDD/non-PD. The mean putamen DAT scan value of the PD classified cases, averaged across all PD classified cases at 12-36 months, was .69 (*SD* = .27). Overall, 104/144 (72%) random forest model predictions matched 12-24 month diagnoses.

At its optimal cutoff (.542), XGBoost long-term model sensitivity approximation amounted to 13/16 (81.25%) correct class predictions that matched 12-24 month diagnoses. The long-term approximation of model sensitivity was calculated from baseline model prediction matches with 12-24 month diagnoses as follows: the XGBoost model misclassified 3/16 (18.75%) cases as SWEDD, which were diagnosed as PD at 12-24 months; two model false classified as early PD by the model proved to be correct predictions and were diagnosed as PD at 12-24 months; 11/14 (78.57%) model true positives, SWEDD predicted cases, were consistent with SWEDD diagnosis at 12-24 months. The latter SWEDD group of 11 non-PD pathology cases was a mix of 3 essential tremor, 3 other neurological disorders, 4 cases that were without evident disease, and 1 case of Alzheimer’s disease. The sensitivity metric, again, amounted to 13/16 (81.25%) correct long-term class predictions. The mean putamen DAT scan value averaged across all SWEDD/non-PD at 12-36 months was 2.0 (*SD* = .50). As concerned specificity, the 12-24 month diagnoses verified 97/128 (75.78%) cases (model true negatives) were correctly classified by as PD. There were 31/128 (24.22%) cases (model false positives) misclassified by the model as SWEDD/non-PD. The mean putamen DAT scan value of the PD classified segment, averaged across all PD diagnosed cases at 12-36 months, was .70 (*SD* = .31). Overall, 110/144 (76.39%) XGBoost model predictions matched 12-24 month diagnoses.

| **TABLE** | **SV-20** |  |  |  |  |  |  |  |  |  |  |  |  |  |  |  |  |  |  |
| --- | --- | --- | --- | --- | --- | --- | --- | --- | --- | --- | --- | --- | --- | --- | --- | --- | --- | --- | --- |
|  | Age | Yrs ed | Upsit-rev | AB 1-42 | CSF asyn | pTau | tTau | UPDSR3 | CNST | Anxiety | RBDQ | GDS | ESS | MoCA | CaudR | CaudL | PutR | PutL | AveCaud |
| Age | 1 |  |  |  |  |  |  |  |  |  |  |  |  |  |  |  |  |  |  |
| Yrs ed | 0.03 | 1 |  |  |  |  |  |  |  |  |  |  |  |  |  |  |  |  |  |
| Upsit-rev | 0.22 | -0.09 | 1 |  |  |  |  |  |  |  |  |  |  |  |  |  |  |  |  |
| AB 1-42 | -0.03 | 0.02 | -0.15 | 1 |  |  |  |  |  |  |  |  |  |  |  |  |  |  |  |
| CSF asyn | 0.18 | 0 | -0.07 | 0.59 | 1 |  |  |  |  |  |  |  |  |  |  |  |  |  |  |
| pTau | 0.27 | 0.07 | -0.08 | 0.58 | 0.82 | 1 |  |  |  |  |  |  |  |  |  |  |  |  |  |
| tTau | 0.26 | 0.05 | -0.07 | 0.61 | 0.81 | 0.98 | 1 |  |  |  |  |  |  |  |  |  |  |  |  |
| UPDSR3 | 0.1 | -0.02 | 0.54 | -0.13 | -0.11 | -0.14 | -0.12 | 1 |  |  |  |  |  |  |  |  |  |  |  |
| CNST | 0.11 | -0.02 | 0.2 | -0.06 | -0.08 | -0.05 | -0.05 | 0.29 | 1 |  |  |  |  |  |  |  |  |  |  |
| Anxiety | -0.09 | -0.11 | 0.17 | -0.06 | -0.12 | -0.12 | -0.11 | 0.24 | 0.23 | 1 |  |  |  |  |  |  |  |  |  |
| RBDQ | -0.02 | -0.05 | 0.18 | -0.08 | -0.09 | -0.06 | -0.04 | 0.29 | 0.21 | 0.24 | 1 |  |  |  |  |  |  |  |  |
| GDS | -0.1 | -0.13 | 0.1 | -0.07 | -0.03 | -0.05 | -0.05 | 0.04 | -0.01 | 0.16 | 0.04 | 1 |  |  |  |  |  |  |  |
| ESS | -0.03 | -0.09 | 0.04 | -0.02 | -0.01 | -0.02 | -0.02 | 0.08 | 0.13 | 0.18 | 0.23 | 0.11 | 1 |  |  |  |  |  |  |
| MoCA | -0.21 | 0.04 | -0.24 | -0.05 | -0.09 | -0.07 | -0.08 | -0.24 | -0.12 | -0.06 | -0.08 | -0.05 | 0.01 | 1 |  |  |  |  |  |
| CaudR | -0.15 | -0.01 | -0.48 | 0.11 | 0.12 | 0.09 | 0.06 | -0.53 | -0.13 | -0.13 | -0.17 | 0.01 | -0.01 | 0.15 | 1 |  |  |  |  |
| CaudL | -0.15 | -0.02 | -0.5 | 0.15 | 0.13 | 0.12 | 0.1 | -0.48 | -0.12 | -0.11 | -0.16 | -0.01 | -0.04 | 0.11 | 0.85 | 1 |  |  |  |
| PutR | -0.1 | -0.03 | -0.55 | 0.14 | 0.15 | 0.14 | 0.12 | -0.65 | -0.15 | -0.13 | -0.17 | 0.01 | 0.03 | 0.19 | 0.85 | 0.73 | 1 |  |  |
| PutL | -0.1 | -0.05 | -0.58 | 0.16 | 0.15 | 0.16 | 0.15 | -0.59 | -0.14 | -0.1 | -0.16 | 0 | -0.03 | 0.11 | 0.73 | 0.86 | 0.79 | 1 |  |
| AveCaud | -0.16 | -0.02 | -0.51 | 0.14 | 0.12 | 0.11 | 0.08 | -0.53 | -0.13 | -0.12 | -0.18 | 0 | -0.02 | 0.13 | 0.96 | 0.96 | 0.82 | 0.82 | 1 |
| AvePut | -0.12 | -0.03 | -0.59 | 0.16 | 0.15 | 0.16 | 0.14 | -0.64 | -0.16 | -0.13 | -0.18 | 0.01 | -0.01 | 0.16 | 0.84 | 0.84 | 0.94 | 0.94 | 0.87 |
|  |  |  |  |  |  |  |  |  |  |  |  |  |  |  |  |  |  |  |  |

References

1.	Robin X, Turck N, Hainard A, Tiberti N, Lisacek F, Sanchez JC, et al. pROC: an open-source package for R and S plus to analyze and compare ROC curves. Bmc Bioinformatics. 2011;12.

2.	Perkins NJ, Schisterman EF. The inconsistency of "optimal" cutpoints obtained using two criteria based on the receiver operating characteristic curve. American Journal of Epidemiology. 2006;163(7):670-5.

3.	Youden WJ. INDEX FOR RATING DIAGNOSTIC TESTS. Biometrics. 1950;6(2):172-3.

4.	Kuhn M. Package caret. In: Kuhn M, editor. Classification and regression training. CRAN2019, March, 3.

5.	Box GEP, Tidwell PW. Transformation of the Independent Variables. <http://www.jstor.org/stable/1266288>: Technometrics; 1962. p. 531-50.

6.	Cohen J, Cohen P, West SG, Aiken LS. Applied Multiple Regression Correlation Analysis for the Behavioral Sciences. 3r ed. ed. New Jersey: Lawrence Erlbaum Associates, Inc.; 2003.

7.	McFadden D. Conditional logit analysis of qualitative choice behavior. Frontiers in Econometrics. 1974:105-42.

8.	Bennette C, Vickers A. Against quantiles: categorization of continuous variables in epidemiologic research, and its discontents. Bmc Medical Research Methodology. 2012;12.

9.	Chen T, Guestrin, C. XGBoost: A Scalable Tree Boosting System. SIGKDD. 2016:785-94.

10.	Hanley JA, McNeil BJ. THE MEANING AND USE OF THE AREA UNDER A RECEIVER OPERATING CHARACTERISTIC (ROC) CURVE. Radiology. 1982;143(1):29-36.

11.	Vittinghoff E, McCulloch CE. Relaxing the rule of ten events per variable in logistic and Cox regression. American Journal of Epidemiology. 2007;165(6):710-8.

12.	Poole C. Low P-values or narrow confidence intervals: Which are more durable? Epidemiology. 2001;12(3):291-4.

13.	Greenland S. DOSE-RESPONSE AND TREND ANALYSIS IN EPIDEMIOLOGY - ALTERNATIVES TO CATEGORICAL ANALYSIS. Epidemiology. 1995;6(4):356-65.

14.	Royston P. A strategy for modelling the effect of a continuous covariate in medicine and epidemiology. Statistics in Medicine. 2000;19(14):1831-47.

15.	Erro R, Schneider SA, Stamelou M, Quinn NP, Bhatia KP. What do patients with scans without evidence of dopaminergic deficit (SWEDD) have? New evidence and continuing controversies. Journal of Neurology Neurosurgery and Psychiatry. 2016;87(3):319-23.
